# α7 integrin co-regulates chromatin accessibility and gene expression in HPV-negative head and neck cancer

**DOI:** 10.1038/s41392-026-02995-9

**Published:** 2026-10-07

**Authors:** Olegs Borodins, Anne Vehlow, Katrin Sameith, Andreas Dahl, Jennifer M. Spangle, Daniela E. Aust, Cornelia Brunner, Outi Monni, Inga Lange, Jovan Mircetic, Elisa Thomas, Nils Cordes

**Affiliations:** 1 OncoRay-National Center for Radiation Research in Oncology, Faculty of Medicine Carl Gustav Carus, Technische Universität Dresden, Dresden, Germany; 2 DRESDEN-concept Genome Center, Technology Platform at the Center for Molecular and Cellular Bioengineering (CMCB) Technology Platform, Technische Universität Dresden, Dresden, Germany; 3 Department of Radiation Oncology, Winship Cancer Institute of Emory University, Atlanta, GA USA; 4 Department for Pathology and Tumour and Normal Tissue Bank of the National Center for Tumor Diseases/University Cancer Center (NCT/UCC), University Hospital Carl Gustav Carus, Technische Universität Dresden, Dresden, Germany; 5 Department of Otorhinolaryngology, Ulm University Medical Center, Ulm, Germany; 6 Applied Tumor Genomics Research Program, Faculty of Medicine, University of Helsinki, Helsinki, Finland; 7 ICAN Digital Precision Cancer Medicine Flagship, Helsinki, Finland; 8 Laboratory of Genetics, Diagnostic Center, Helsinki University Hospital, Helsinki, Finland; 9 Helmholtz-Zentrum Dresden-Rossendorf (HZDR), Institute of Radiooncology - OncoRay, Dresden, Germany; 10 German Cancer Consortium (DKTK), Partner Site Dresden, and German Cancer Research Center (DKFZ), Heidelberg, Germany; 11 Department of Radiotherapy and Radiation Oncology, University Hospital Carl Gustav Carus, Technische Universität Dresden, Dresden, Germany

**Keywords:** Head and neck cancer, Cancer microenvironment, Gene expression analysis

## Abstract

Epigenetic regulation and gene transcription are dynamically modulated by extracellular signals, including focal adhesions; however, the role of integrins in these processes remains poorly understood. In this study in HPV-negative head and neck squamous cell carcinoma, (HNSCC), we combined investigations in physiological three-dimensional, matrix-embedded cell models and patient-derived organoids with multi-omics analyses, bioinformatics and artificial intelligence, to unmask the integrin effector signaling network that reshapes chromatin to drive cancer. Among the 12 α/β1 integrin receptors, α7 integrin (ITGA7) emerged as the most potent modulator of histone post-translational modifications, associated with significant changes in gene transcription and chromatin accessibility. Depletion of ITGA7, achieved independently by CRISPR/Cas9 knockout and RNA interference, reduced chromatin accessibility at the AURKA locus and downregulated an AURKA-centered transcriptional program. Mechanistically, phosphoproteomics revealed significant inhibition of MAPK- and PI3K/AKT-dependent signaling with concomitant functional impairment of cell cycling and cell survival upon ITGA7 depletion. Across all analyses, machine-learning-based feature selection consistently nominated AURKA as the dominant downstream node, and tumor ITGA7 expression stratified patient overall survival in a non-linear manner. In summary, these findings establish α7 integrin as a key co-regulator of chromatin accessibility and gene transcription in HPV-negative HNSCC, acting through an AURKA-centered axis that couples adhesion signaling to cell cycle control and patient survival, and nominate AURKA, potentially targetable with clinical Aurora-A inhibitors such as alisertib, as an actionable therapeutic node downstream of the currently non-druggable ITGA7.

## Introduction

Although epigenetic regulation and gene transcription occur in the nucleus, they are dynamically controlled through complex feedback interactions with extracellular signals in all living organisms.^1^ Finely tuned in healthy tissue, genetic mutations and epigenetic dysregulations in human malignancies lead to intra- and intertumoral, highly heterogeneous autonomy towards these signals.^2^ Be it pathological deregulation of, for example, growth factors, oxygen, metabolism or extracellular matrix (ECM) remodeling,^3–7^ extracellular signals adaptively translate into the required phosphoproteomic repertoire to maintain survival via epigenetic regulation and gene transcription. Because extensive ECM remodeling coincides with altered expression of cell adhesion molecules (CAM) such as integrin receptors and their downstream signaling,^8,9^ this raises the question of to what extent integrins regulate tumor survival through participation in epigenetic regulation and gene transcription.

Integrins are a family of transmembrane receptors coalescing with receptor tyrosine kinases (RTK), adapter proteins and protein kinases at large adhesion and signaling hubs termed focal adhesions (FA).^8,10,11^ Twenty-four integrins have been identified composed from 18 α and 8 β subunits, with β1 integrin being central to 12 out of the 24 integrin receptors.^12^ The promitogenic and prosurvival functions of integrins via FAK/Src, PI3K/AKT and MAPK signaling pathways as well as in local invasion, metastasis and therapy resistance are well documented.^8,13^ Intervention studies have identified integrins as therapeutically actionable targets and key regulators of DNA repair and transcription.^14–19^

Cellular function depends on tightly regulated gene expression and protein biosynthesis, which are driven by extracellular signals and mediated through epigenetic and chromatin-related mechanisms. Chromatin is organized into highly condensed, transcriptionally inactive heterochromatin and more open, transcriptionally active euchromatin.^2^ The smallest functional unit is the nucleosome, defined as a region of histone-wrapped DNA with specific DNA and histone modifications.^2,20,21^ All DNA-based processes such as transcription, DNA repair and replication are highly dynamic and tightly controlled by chromatin-modifying enzymes. These modifications can alter chromatin structure and enable the recruitment of chromatin remodeling enzymes.^22^ In cancer, abnormal expression patterns or genomic modifications in chromatin regulators are associated with oncogenesis and cancer progression.^2,23,24^ Histones, particularly histone H3 and H4, undergo a range of chemical changes known as histone posttranslational modifications (HPM).^22^ These modifications play a central role in regulating chromatin accessibility thereby controlling processes such as transcription and DNA replication. An exemplary modification is acetylation of lysine residues (e.g., H3K27ac and H3K9ac).^22^ This process neutralizes the positive charge on histones weakening their interaction with negatively charged DNA. As a result, the chromatin structure becomes more relaxed typically associated with active gene transcription. This is co-regulated by methylation of histones such as H3K4me1, H3K4me3, and H3K36me3.^22^ In contrast, other HPM like H3K9me3 and H3K27me3 are associated with gene repression and more compact chromatin. Phosphorylation of histones, particularly during cellular signaling and different stages of the cell cycle, can also promote chromatin relaxation.^22^ This facilitates access to DNA for transcription or replication machinery. Together, these modifications determine whether chromatin adopts an open, transcriptionally active state or a condensed, repressed configuration.^22^ Such HPM patterns and epigenetic regulation have been investigated for a number of integrins and in the context of mechanotransduction.^25–27^ For gene transcription, outside-in signaling via β2 integrin family members promote the induction of anti-inflammatory cascades and is mediated by transcription factors (TF) such as AP-1, Mac-1 or IEX-1.^28,29^ But how integrins affect epigenetic regulation and transcription in general remains to be shown.

To address this question, we examined HPV-negative head and neck squamous carcinoma (HNSCC) models and patient-derived organoids (PDO) grown under physiological three-dimensional (3D), matrix-embedded conditions as an exemplary solid cancer type. We combined a knockdown screen for 12 α/β1 integrins with computational analyses on RNA-sequencing (RNA-seq), Assay for Transposase-Accessible Chromatin using sequencing (ATAC-seq) and phosphoproteome data sets. Among all integrins, knockdown of α7 integrin (ITGA7) showed the strongest impact on HPM with significant changes in transcription and chromatin accessibility. These were mainly associated with perturbations in heterochromatin organization, cell cycle progression and cell viability by multi-omics analyses, an observation that was supported by functional assays. Overall, our results suggest that signaling mediated by α7 integrin regulates chromatin accessibility and gene expression profiles, supporting the paradigm that chromatin integrates upstream cues from cell adhesion signaling, to regulate transcriptional output.

## Results

### Integrins are predicted to act as co-regulators of epigenetic mechanisms in HNSCC

**Fig. 1:**
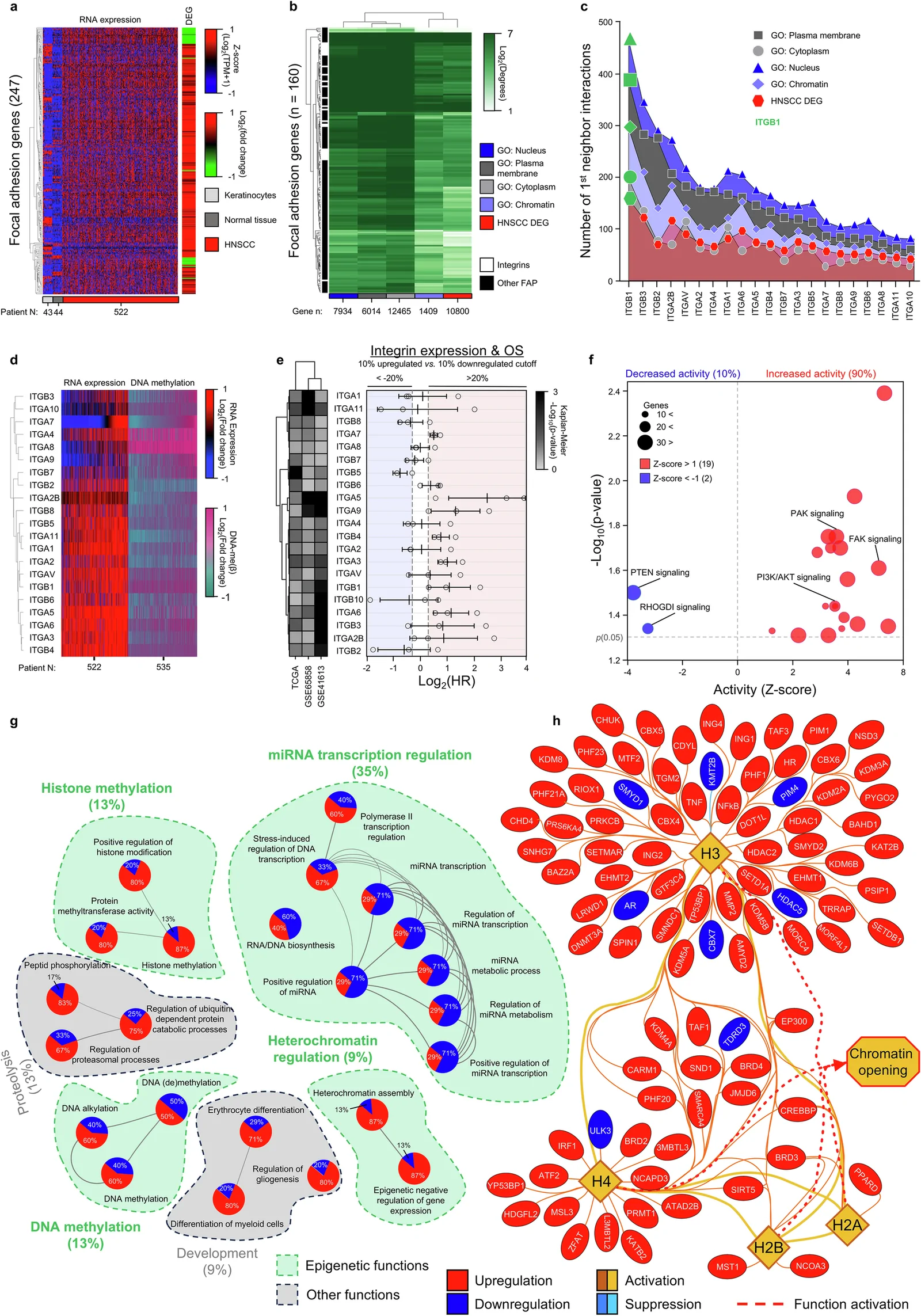
HNSCC show focal adhesion gene overexpression with an emerging potential for integrins as novel epigenetic co-regulators**. a** Heatmap illustrating the differential expression of genes associated with focal adhesions in HNSCC. Log_2_ normalized transcript per million (TPM + 1) gene expression levels are compared with corresponding normal tissues (TCGA) and normal keratinocytes (GEO). Differentially expressed genes (DEG; 247) in HNSCC (sourced from TCGA) are highlighted (right column). **b** Heatmap showing the degree of interaction between adhesome (GO:0005925), nucleus (GO:0005634), plasma membrane (GO:0005886), cytoplasm (GO:0099568), and chromatin (GO:0000785) genes or HNSCC DEG within the gene interaction network constructed using STRING with a 75% confidence score. **c** Hierarchical order of all integrins based on number of first neighbor interactions with genes in indicated groups. **d** Comparative analysis of differentially expressed integrin genes from TCGA HNSCC patients and corresponding fold change in DNA methylation beta values (relative to normal tissue). **e** p-values from Kaplan–Meier-based overall survival (OS) considering 10% upper and lower integrin expression (left) and corresponding hazard ratios (HR; right) in the HNSCC patient cohort (3 independent studies are included). **f** Functional enrichment analysis of integrin-associated DEG from Fig. 1b conducted with IPA. **g** ClueGO- and IPA-based analyses on the predicted chromatin regulatory activities of upstream and master regulators in HNSCC for DEG associated with β1 integrin and its 12 α subunits. **h** Integrin associated DEG interactive network from IPA specific for a chromatin opening signaling pathway

The dual functionality of FA proteins in adhesion and downstream signaling, as well as physical connections between FA and the nuclear membrane, suggests a potential role of FA proteins in epigenetic regulation.^8,11,13^ Particularly in cancer such as HNSCC, FA signaling and chromatin organization are rewired to propel survival and therapy resistance.^30,31^ To determine the function of FA components as co-determinants of epigenetic regulation, we began by analyzing 474 focal adhesion (FA)-associated genes from Gene Ontology (GO) database. By comparing matched healthy tissues and keratinocytes from various publicly available databases, we identified 247 differentially expressed genes (DEG) associated with FA in HNSCC (Fig. 1a and Supplementary Data S1). As 89% of these FA-related DEG were upregulated relative to healthy tissue (Supplementary Fig. 1a and Supplementary Data S1), we subsequently constructed an interaction network between these FA genes and all genes associated with a specific cellular compartment, *i.e*. plasma membrane, cytoplasm, nucleus, and chromatin, as well as all DEG in the HNSCC The Cancer Genome Atlas (TCGA) dataset (Supplementary Fig. 1b and Supplementary Data S1). A Cytoscape protein-protein interaction, STRING-based network was built with a 75% confidence score, considering only genes that share at least one interconnection across all analyzed groups (Fig. 1b and Supplementary Data S1). Analysis of first neighbor interactions revealed 160 FA genes with integrins as major interactors of chromatin regulatory genes and the HNSCC DEG groups (Supplementary Fig. 1c-g). As expected, integrins were strongly represented in plasma membrane and cytoplasmic interactions; notably, they also showed extensive connectivity with nuclear and chromatin-associated genes (Fig. 1c). Among the 21 integrins analyzed, ITGB1 exhibited the highest degree of connectivity across all groups (Fig. 1c). We next examined DNA methylation data from TCGA HNSCC samples and found distinct patterns among integrins. Some integrins were highly expressed despite showing relatively high levels of methylation across their gene-associated regions (e.g., ITGB1, ITGB4, ITGA4, ITGA6, ITGA8). In contrast, others were also overexpressed but had comparatively low methylation levels (e.g., ITGB2, ITGB6, ITGB8, ITGA2, ITGA2B, ITGA11; Fig. 1d). Importantly, several integrins were associated with poorer patient outcomes regardless of their expression or methylation status. These included ITGA7, ITGB6, ITGA5, ITGA9, ITGB4, ITGA3, ITGAV, ITGB1, ITGA6, ITGB3, and ITGA2B, each associated with an approximately 20% increased risk of death (Fig. 1e and Supplementary Figs. 2 and 3). Next, we confined our analysis to all subunits of the 12 α/β1 integrin heterodimers and merged them with 196 associated HNSCC DEG (Supplementary Fig. 4a and Supplementary Data S1) in a subsequent Ingenuity Pathway Analysis (IPA) for functional enrichment with a focus on epigenetic regulatory pathways. In addition, 8 chromatin regulatory genes and a significant activation of key canonical, survival-advantaging pathways, including FAK, PAK, PI3K/AKT signaling, as well as inhibition of PTEN and RHOGDI signaling were predicted (Fig. 1f; Supplementary Data S1; and Supplementary Fig. 4b). Subsequent IPA of upstream gene regulators of integrin-associated DEG identified 13 regulatory genes, of which 11 were predicted to be more activated (activation Z-score > 1) and 2 more suppressed (-1 > activation Z-score), including 11 epigenetic regulatory genes (Supplementary Fig. 4c). We also found 237 significantly more activated and 69 more inhibited master regulators, including 42 chromatin regulatory genes (Supplementary Fig. 4d and Supplementary Data S1). Functionally, these epigenetic regulators act in positive regulation of miRNA transcription (reduced activity; 35%), histone modification (increased activity; 13%) and heterochromatin regulation (increased activity; 9%) (Fig. 1g). A general activation of chromatin openness mechanisms appears to exist in HNSCC via canonical histone modification pathways (Fig. 1h). These findings highlight the relevance of FA proteins, particularly integrins, in HNSCC progression and suggest their involvement in chromatin regulation.

### Chromatin marker profiles are altered integrin-dependently

**Fig. 2:**
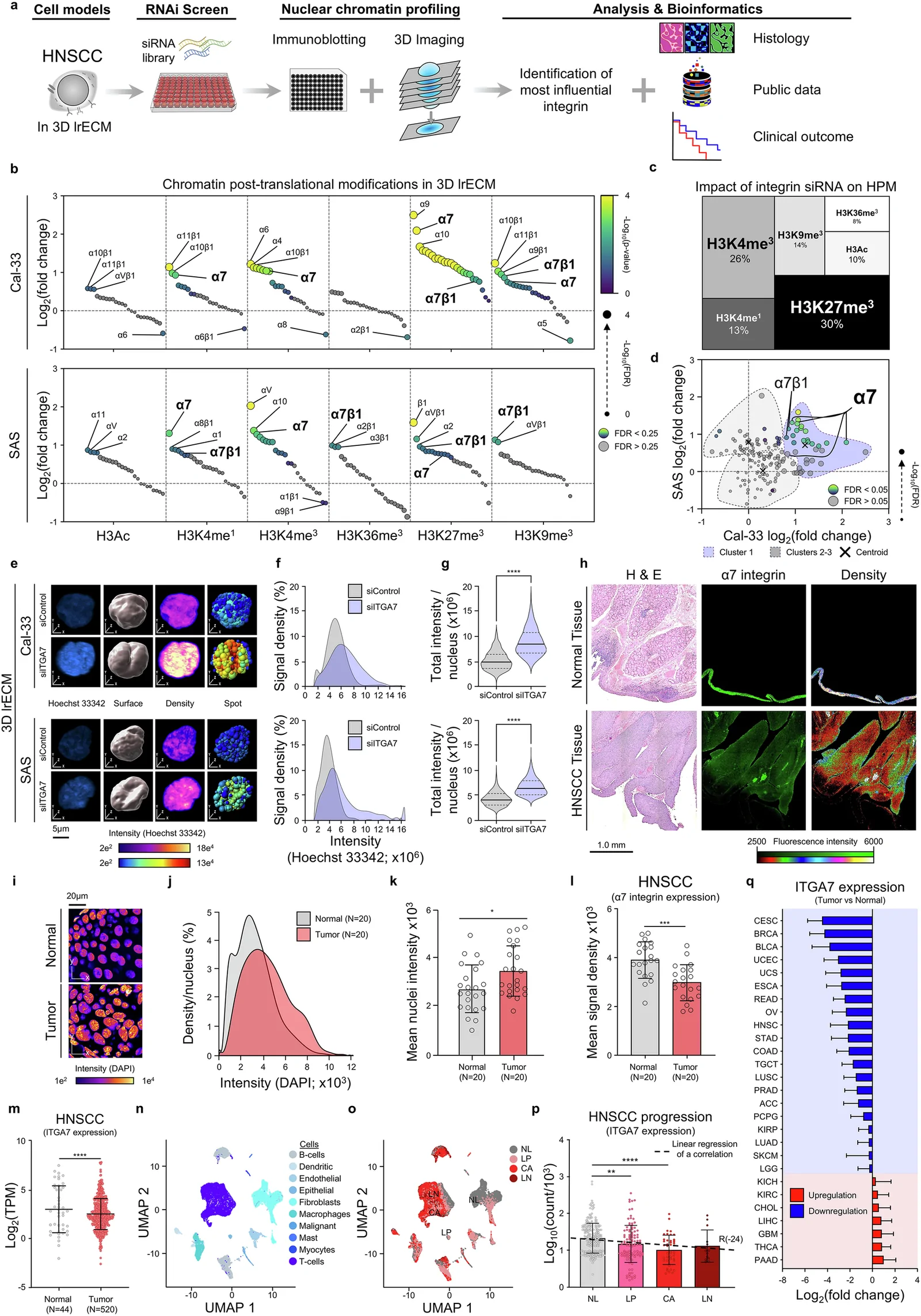
Integrin depletion modifies histone post-translational modifications and targeting of α7 integrin elicits a more heterochromatic DNA state. **a** Schematic of RNAi screen workflow. **b** Fold change of histone posttranslational modifications (HPM) following siRNA-mediated single or combined silencing of β1 integrin and its 12 α subunits compared to non-specific siRNA controls (minimum *N* ≥ 3; two-way ANOVA; two-stage linear step-up procedure of Benjamini, Krieger and Yekutieli; *p* < 0.05; FDR < 0.25). Dot blot analyses of whole cell lysates from 3D lrECM Cal-33 and SAS cell models. **c** Total percentage impa**c**t on specific HPM after integrin knockdown. **d** K-means clustering analysis from Fig. 2b categorizes HPM into 3 groups with similar changes detected in Cal-33 and SAS cell models after integrin silencing (Cluster 1: strongest impact; groups 2-3: lower impact). **e** High-resolution Z-stack images of Hoechst 33342-stained nuclei of ITGA7-silenced cells relative to controls for 3D segmentation analyses. **f** Quantitative distribution of signal density per nucleus from Fig. 2e (63x resolution; *N* = 4; ≥25 nuclei images per biological replicate per group). **g** Comparison of total Hoechst 33342 intensity per nuclear volume from Fig. 2e. Data are presented as mean ± standard deviation with a sample size of *N* = 4 (25x resolution; ≥ 1000 nuclei images per biological replicate per group; unpaired *t*-test; ****p < 0.0001). **h** Staining with hematoxylin and eosin (H&E) and for α7 integrin in HNSCC compared to normal tissue. Intensity heatmaps show signal density of α7 integrin staining. Images were segmented (10x resolution; unsegmented images are shown in Supplementary Fig. 6b). **i** Intensity heatmaps of DAPI-stained nuclei in HNSCC and normal tissue. **j** Comparative quantification of DAPI signal density distributions per nucleus from (**i**). **k** Comparison of total DAPI intensity per nuclear volume from (**i**). Data are presented as mean ± standard deviation with a sample size of *N* = 20 (analyses based on 20 HNSCC and 20 corresponding normal tissues; 25x resolution images, ≥ 100 nuclei images per biological replicate per group unpaired *t*-test; *p < 0.05). **l** Quantitative analysis of α7 integrin intensity in HNSCC relative to normal tissue. Data are presented as mean ± standard deviation with a sample size of *N* ≥ 20 (20 HNSCC, 20 corresponding normal tissues; 25x resolution images; unpaired *t*-test; ***p < 0.001). **m** ITGA7 mRNA expression data from TCGA HNSCC patient cohort, unpaired t-test, ****p < 0.0001. **n**, **o** Single-cell sequencing analysis of mRNA expression at different stages of HNSCC development (NL - normal; LP - leukoplakia; CA - cancer; LN - lymph nodal metastasis). UMAP clustering based on their cell type characteristic (**n**). UMAP clustering based on the tumor development stages (**o**). **p** Single-cell sequencing analysis of ITGA7 expression at different stages of HNSCC from Fig. 2o. Data are presented as mean ± standard deviation with a sample size of *N* = 9 (NL), *N* = 4 (LP), *N* = 20 (CA), *N* = 4 (LN) (one-way ANOVA; Dunn Test with Bonferroni correction; **p_adj_ < 0.01; ****p_adj_ < 0.0001; all conditions vs NL). **q** ITGA7 mRNA expression fold change in 27 cancer types (TCGA database) compare to healthy control. Cancer types are cervical squamous cell carcinoma and endocervical adenocarcinoma (CESC); breast invasive carcinoma (BRCA); bladder urothelial carcinoma (BLCA); uterine corpus endometrial carcinoma (UCEC); uterine carcinosarcoma (UCS); esophageal carcinoma (ESCA); Rectum adenocarcinoma (READ); ovarian serous cystadenocarcinoma (OV); head and neck squamous cell carcinoma (HNSC); stomach adenocarcinoma (STAD); colon adenocarcinoma (COAD); testicular germ cell tumors (TGCT); lung squamous cell carcinoma (LUSC); prostate adenocarcinoma (PRAD); adrenocortical carcinoma (ACC); pheochromocytoma and paraganglioma (PCPG); kidney renal papillary cell carcinoma (KIRP); lung adenocarcinoma (LUAD); skin cutaneous melanoma (SKCM); lower-grade glioma (LGG); kidney chromophobe (KICH); kidney renal clear cell carcinoma (KIRC); cholangiocarcinoma (CHOL); liver hepatocellular carcinoma (LIHC); glioblastoma (GBM); thyroid carcinoma (THCA); pancreatic adenocarcinoma (PAAD). Data are presented as mean ± standard deviation

The central role and proximal location of integrins connecting FA and the nucleus prompted us to perform an RNAi screen targeting all subunits of the 12 α/β1 integrin heterodimers and investigate their impact on 6 key HPM (Fig. 2a). Initially, we characterized a panel of 8 HNSCC cell models cultured in a physiological 3D laminin-rich extracellular matrix (lrECM). Super-resolution laser scanning microscopy allowed us to determine nuclear DNA segmentation patterns by Hoechst 33342 staining and to categorize our cell model panel based on this surrogate marker. We observed a spectrum of nuclear DNA organization ranging from homogeneous (Cal-33) to highly heterogeneous (SAS) (Supplementary Fig. 5a and Supplementary Videos S1–S8). The subsequent HPM analysis provided clearly diverging profiles (Supplementary Fig. 5b). For further investigation, H3K9me3 was selected, a marker for dense, stably organized heterochromatin, which exhibited the lowest expression in Cal-33 and the highest expression in SAS models (Supplementary Fig. 5b, c; leftmost group of columns). Next, we analyzed publicly available TCGA chromatin immunoprecipitation (ChIP)seq data and observed a similar distribution in euchromatic and heterochromatic markers relative to our data (Supplementary Fig. 5d). Reviewing previously published whole exome sequencing data of our 8 HNSCC cell models revealed 60 protein-altering mutations in chromatin regulatory genes known for their roles in post-translational modifications (Reactome).^32^ Thirty-five of these 60 genes were uniquely mutated in our cell models (Supplementary Fig. 5e and Supplementary Data S2), found in the Genome Aggregation Database (gnomAD) and Exome Aggregation Consortium (ExAC), with two-thirds of the 60 genes being rarely mutated in various human populations (Supplementary Fig. 5f and Supplementary Data S2). A ClueGO-based biological functional network analysis revealed that 31% of these mutated genes are involved in peptidyl-lysine modification, 19% in histone modification, 12% in epigenetic regulation, and 38% in other chromatin organization processes (Supplementary Fig. 5g).

Based on these observations and our computational analysis of FA proteins, particularly integrins, we silenced 12 α/β1 integrin receptors to assess their impact on nuclear HPM. To maximize the identification of conserved molecular patterns, these experiments were conducted in two HNSCC cell models, Cal-33 and SAS, which exhibit divergent nuclear characteristics. Our data demonstrate that euchromatic (pan-H3Ac, H3K4me1, H3K4me3, H3K36me3), constitutive heterochromatic (H3K9me3), and facultative heterochromatic markers (H3K27me3) were differentially modified following specific integrin depletion (Fig. 2a–c). K-means clustering analysis identified that α7, and α7β1 integrins were the most influential determinants of HPM, with α7 integrin showing the most pronounced effect (Fig. 2d; FDR < 0.05). The significant increase in the euchromatic markers H3K4me1/me3 and heterochromatic markers H3K27me3/H3K9me3 in both ITGA7-depleted cell models indicated altered transcriptional regulation. Super-resolution laser scanning microscopy of Hoechst 33342-stained, ITGA7-depleted 3D lrECM Cal-33 and SAS cell models uncovered significantly increased spatial distribution and aggregation of nuclear DNA consistent with increased heterochromatin formation compared to controls (Fig. 2e–g). In addition, staining for pan-H3Ac and H3K4me3 confirmed colocalization with low Hoechst 33342 intensity, indicating euchromatic DNA. In contrast, H3K27me3 partially localized to highly dense nuclear regions representing facultative heterochromatin (Supplementary Fig. 6a).

We proceeded by characterizing nuclear density and α7 integrin expression in HNSCC tissue samples (Fig. 2h; right, Supplementary Fig. 6b) alongside corresponding normal tissue (Fig. 2h; left; Supplementary Fig. 6b). Consistent with the elevated area of high-density nuclear regions in ITGA7-depleted HNSCC models, HNSCC samples showed a similar, significant increase in DNA spatial distribution and aggregation in the nucleus when compared to normal tissue (Fig. 2i–k). Intriguingly, α7 integrin expression (mRNA, protein) was clearly reduced in all HNSCC specimens relative to normal tissue (Fig. 2l, m). Of note, high α7 integrin expression correlates with reduced OS in HNSCC patients (Supplementary Fig. 6c, d). Analysis of publicly available single-cell transcriptomic datasets revealed a progressive decrease in mean ITGA7 expression during HNSCC development from normal tissue (NL) to leukoplakia (LP), primary tumors (CA), and lymph node metastases (LN).^33^ These data also enabled us to examine the cell type-specific distribution of ITGA7 expression within HNSCC tumor biopsies (Fig. 2n–p and Supplementary Fig. 6e, f). Beyond HNSCC, ITGA7 mRNA was found to be downregulated in 19 of 27 major cancer types (Fig. 2q). Together, these findings support a regulatory role for integrins, particularly α7 integrin, in chromatin organization in HNSCC.

### ITGA7 co-controls gene transcription in HNSCC models

**Fig. 3:**
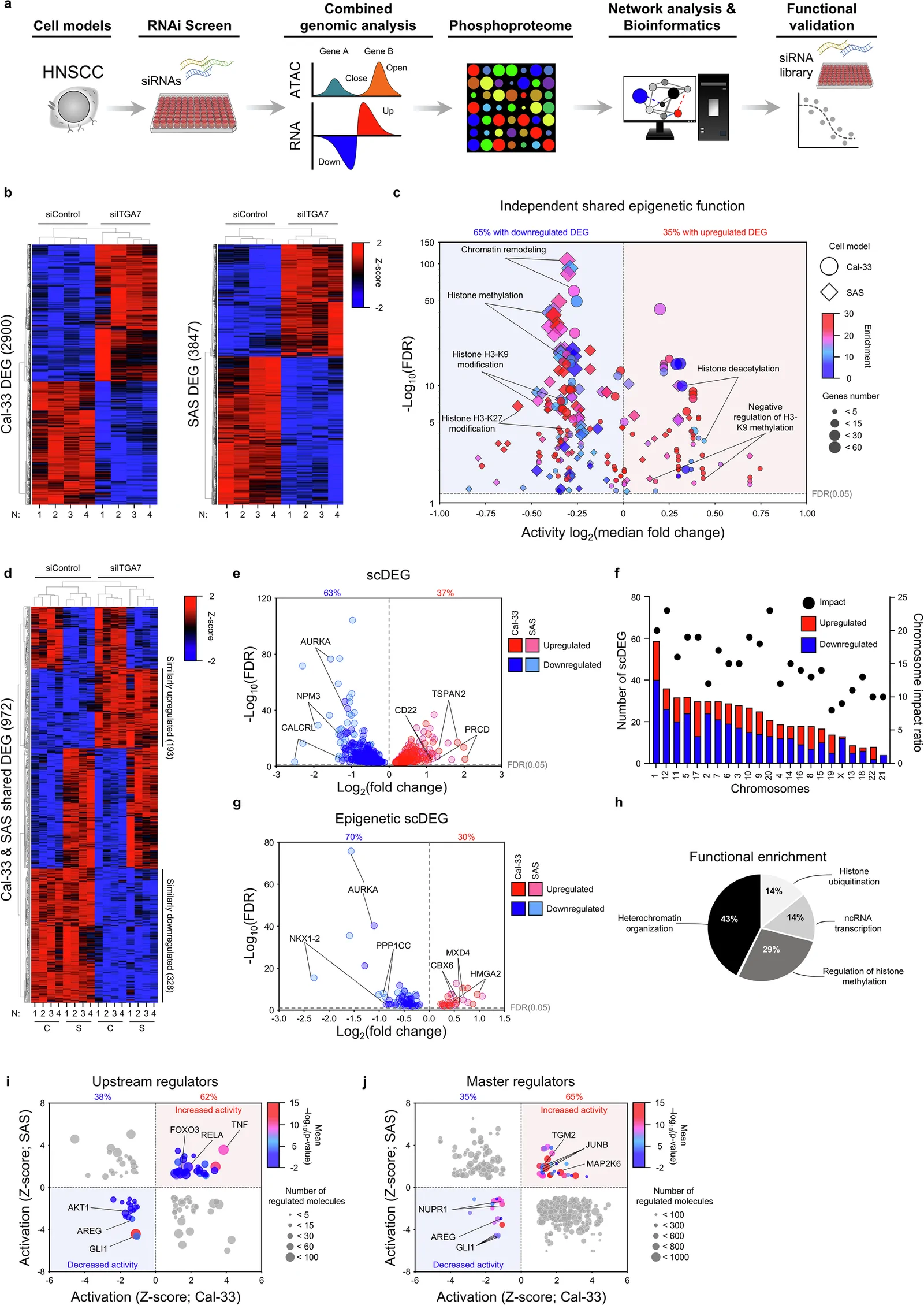
ITGA7 depletion induces profound transcriptomic alterations in 3D lrECM HNSCC cell models. **a** Schematic of the omics workflow. **b** Cell model-specific hierarchically clustered transcriptome changes upon ITGA7 silencing (*N* = 4; DESeq2; *p* < 0.05; FDR < 0.05). **c** GO-based analysis for epigenetically enriched biological processes shared between the two ITGA7-depleted cell models. **d** Hierarchical clustering of shared differentially expressed genes (DEG) in 3D lrECM Cal-33 and SAS cultures upon ITGA7 silencing (*N* = 4; DESeq2; *p* < 0.05; FDR < 0.05). **e** Plotting of similarly changed DEG (scDEG) from **d** (all analyses considered FDR < 0.05). **f** Chromosomal occupancy of scDEG with chromosome enrichment scores of genes from (**d**). **g** Plotting of epigenetically associated scDEG from (**d**). **h** GO term-based hierarchical clustering for functional commonality based on ClueGO network analysis for scDEG from (**g**). **i** IPA-based analysis of similarly changed upstream regulatory enrichment analysis for DEG from Cal-33 and SAS cell models. **j** IPA-based analysis of similarly changed master regulatory enrichment analysis for DEG from Cal-33 and SAS cell models

Hypothesizing that α7 integrin acts as an epigenetic co-regulator, we anticipated that ITGA7 depletion would alter both the transcriptome and chromatin accessibility. First, we conducted a comparative analysis of the transcriptomic response in ITGA7-depleted 3D lrECM Cal-33 and SAS models (Fig. 3a and Supplementary Fig. 7a, b). We detected 2900 DEG in Cal-33 and 3847 DEG in SAS models (*p* ≤ 0.05; FDR < 0.05) using DESeq2 (Fig. 3b; Supplementary Fig. 7c, d; and Supplementary Data S3). IPA of canonical pathways shared between the two ITGA7-depleted cell models predicted significant activation of MAPK, PTEN, and ILK pathways (Supplementary Figs. 7e and 8a, b) as well as significant inhibition of mitotic prophase, EMT, pyrimidine deoxyribonucleotide biosynthesis, and histone modification, which prompted us to further focus on epigenetic pathways. ShinyGO analysis revealed shared, strongly impacted pathways between the two HNSCC models associated with histone methylation/demethylation and heterochromatin organization, with over 60 DEG related to histone modification (Fig. 3c and Supplementary Data S3). We next focused on shared DEG) and identified 521 genes that were consistently altered across both Cal-33 and SAS models. These similarly changed DEG (scDEG) were defined as genes that were significantly differentially expressed and showed the same direction of change (either upregulation or downregulation) across datasets or experimental conditions. Among these, 63% were downregulated, and 37% were upregulated compared to controls (Fig. 3d, e and Supplementary Data S3). Genes such as AURKA, NPM3, and CALCRL were among the most strongly repressed, whereas WNT4, TSPAN2, and CD22 were among the most strongly induced (Fig. 3e). Chromosome enrichment analysis revealed a pronounced accumulation of scDEG on chromosomes 12 (which harbors ITGA7) and 20 (Fig. 3f). In addition, 50 of the 521 scDEG were linked to epigenetic regulation. Of these, 70% including AURKA, NPM3, and NKX1-2 were downregulated following ITGA7 depletion (Fig. 3g). The most strongly induced epigenetic regulators were SDR16C5, HMGA2 and MXD4 (Fig. 3g). ClueGO analysis further indicated that these identified genes are involved in heterochromatin regulation (Fig. 3h). We next performed IPA upstream regulator analysis on ITGA7-dependent transcriptional changes and identified a set of shared regulators that were either predicted to be activated (e.g., TNF, FOXO3, RELA) or inhibited (e.g., AREG, GLI1, AKT1). These findings suggest that such transcriptional regulators may play a role in mediating the epigenetic effects observed following ITGA7 knockdown (Fig. 3i and Supplementary Fig. 8c, d). Subsequently, the predicted master regulatory network identified increased activity in TGM2, JUNB, and MAP2K6, and a decrease in NUPR1, GLI1, and AREG (Fig. 3j and Supplementary Fig. 8e, f). Taken together, these findings support a role for α7 integrin in regulating chromatin organization through coordinated transcriptional networks in HNSCC models.

### ITGA7 depletion elicits profound changes in chromatin accessibility

**Fig. 4:**
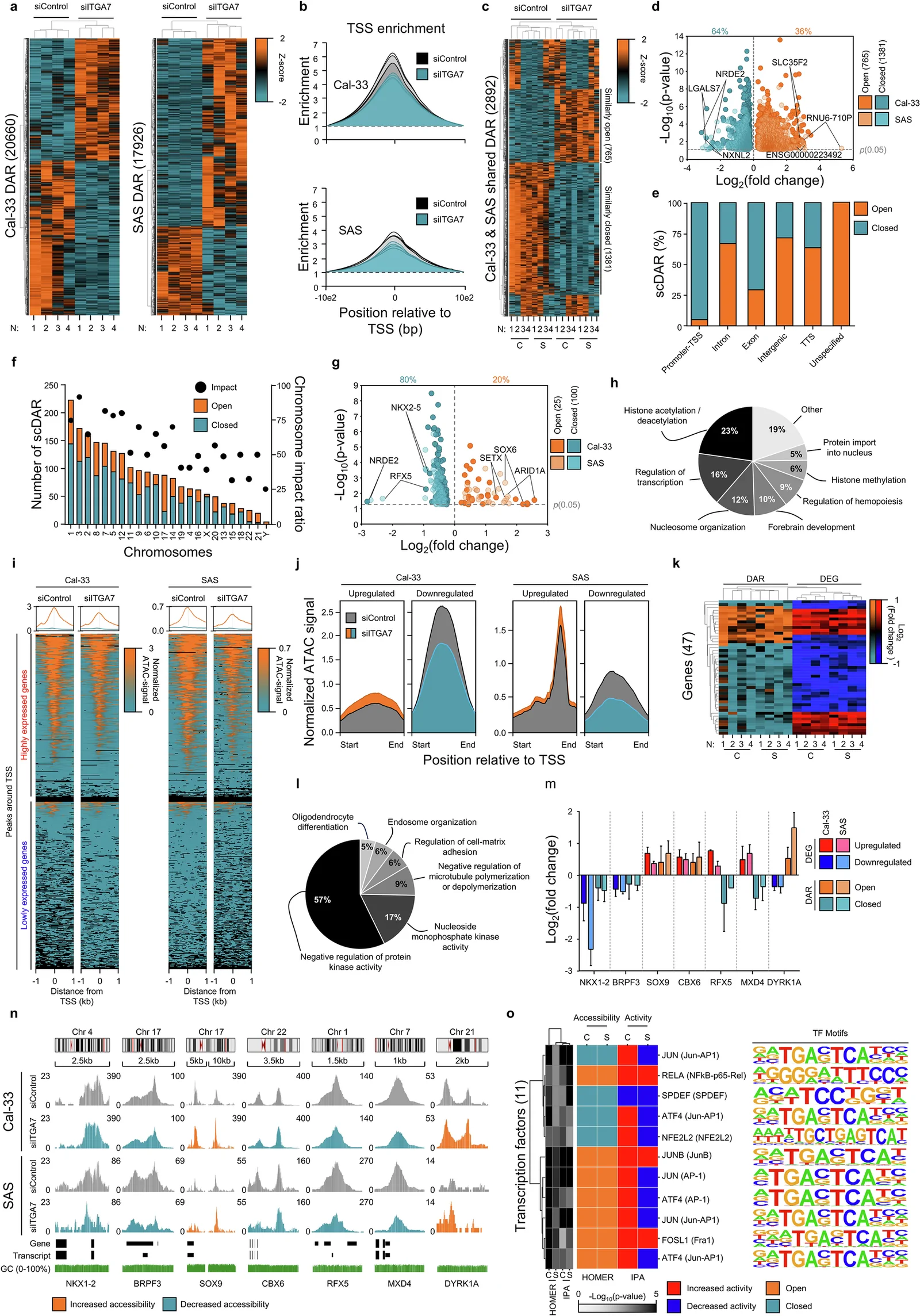
Chromatin accessibility is strongly modified in ITGA7-depleted 3D lrECM HNSCC cell models. **a** Cell model-specific hierarchically clustered chromatin accessibility regions (DAR) changes upon ITGA7 depletion (*N* = 4; DESeq2; *p* < 0.05). **b** ATAC-normalized signal enrichment in global transcription starting sites (TSS) in both ITGA7-silenced cell models (*N* = 4). **c** Hierarchi**c**al clustering of shared differentially DAR upon ITGA7 silencing (*N* = 4; DESeq2; *p* < 0.05, FDR < 0.25). **d** Plotting of similarly changed differentially accessible regions (scDAR) between the two cell models from (**c**) (all analyses considered *p* < 0.05). **e** Distribution of all scDAR peaks across mapped genomic components. **f** Chromosomal occupancy of scDAR shared between Cal-33 and SAS models, showing chromosome enrichment scores of genes from (**c**). Stacked bars indicate the fraction of all scDAR located on each chromosome. Orange and turquoise segments represent regions with increased (open) and decreased (closed) chromatin accessibility, respectively, illustrating the directionality of ITGA7-dependent chromatin accessibility changes across chromosomes. **g** Plotting of epigenetically associated scDAR from (**c**). **h** ClueGO-based network analysis for epigenetically associated scDAR functions. **i** Tornado heatmaps showing ATAC-normalized s**i**gnals around TSS of the top 300 highest and lowest expressed genes upon ITGA7 depletion. **j** ATAC-normalized signals around TSS of differentially expressed up- and downregulated genes after ITGA7 knockdown compare to controls. **k** Hierarchical clustering of genes with shared relationships in ATAC- and RNA-seq datasets. **l** ClueGO-based network analysis for functional commonality between shared scDAR and scDEG. **m** Comparative depiction of expression and accessibility of epigenetically associated scDEG from (**k**). Data are presented as mean ± standard deviation with a sample size of *N* = 4. **n** Genome view of peaks for genes depicted in (**m**). **o** Combined analysis of transcription factors associated with transcription (from Fig. 3) and ATAC datasets with significantly (*p* < 0.05) predicted increased or decreased activity and expression, including their recognition motifs in DAR identified in Cal-33 (C) and SAS (S) cell models

To directly link transcriptomic changes to modifications in chromatin accessibility, we performed ATAC-seq in tandem with the above RNA-seq (see workflow in Fig. 3a). Our analysis revealed 20,660 differentially accessible chromatin regions (DAR) in Cal-33 and 17,926 DAR in SAS models after ITGA7 depletion relative to controls (*p* ≤ 0.05; Fig. 4a; Supplementary Fig. 9a, b; and Supplementary Data S4). The overall distribution of ATAC-seq peaks under control conditions was similar for both cell models, with over 70% of peaks located in non-coding regions, such as introns and intergenic regions, while the remaining 29% were found in promoters, transcription start sites (TSS), and exons (Supplementary Fig. 9c). Upon ITGA7 knockdown, we observed a general increase in peak numbers across all regions in the Cal-33 model, whereas the SAS model showed a global decrease in peaks (Supplementary Fig. 9d). Interestingly, we detected reduced DNA accessibility at TSS in both cell models, further indicating a general and corresponding loss of accessibility following ITGA7 knockdown (Fig. 4b). Comparable to our transcriptome analysis, we focused on shared DAR to identify regions of consistent chromatin change. We found 2892 shared DAR, of which 2146 (74%) represent similarly changed DAR (scDAR) in the two cell models (Fig. 4c, d and Supplementary Data S4). The vast majority of these scDAR localized to intronic regions (27%) and promoter TSS (49%) (Supplementary Fig. 9e), with 64% showing decreased promoter TSS accessibility (Fig. 4d). In contrast, introns and transcription termination sites (TTS), exhibited increased accessibility (Fig. 4e and Supplementary Fig. 9e). Consistent with the accumulation of scDEG to chromosomes 12 and 20, chromosomal enrichment of scDAR demonstrated similar and significant clustering on chromosomes 3, 12, and 20 (Fig. 4f). To further underline the functional involvement of α7 integrin, we searched for scDAR involved in epigenetic regulation and found peaks associated with 125 genes (Fig. 4g and Supplementary Data S4), 80% of these genes showed reduced accessibility, for example for NRDE2, RFX5 and NKX2-5, in contrast to those with increased accessibility such as ARID1A, SOX6 and SETX. ClueGO-based functional analysis further emphasized that 29% of these genes had functions in histone modification, 16% in transcription, and 12% in nucleosome regulation (Fig. 4h).

Next, we asked whether there is a mechanistic link between shared DEG and DAR by comparing ATAC-seq peaks and transcription (Fig. 4i). We observed a clear enrichment of ATAC-seq signals in TSS regions of the top 300 highly expressed genes, while the top 300 lowly expressed genes showed a loss of peak signals under all conditions and cell models. Likewise, an increased accessibility in upregulated genes and decreased accessibility in downregulated genes was found after ITGA7 depletion, indicating a direct relationship between chromatin accessibility and transcriptional activity (Fig. 4j). We identified 47 peaks in shared DEG and DAR, including 34 mirroring genes (genes that show the same vector of change across different conditions or models), of which 9 were upregulated with increased accessibility and 23 downregulated with reduced accessibility (Fig. 4k). Given the consistent impact of promoter accessibility on gene expression, we employed functional enrichment analysis on all shared DEG and DAR, which revealed 57% of these genes to be engaged in negative regulation of protein kinase activity, primarily through the regulation of protein phosphorylation (Fig. 4l). Narrowing down to shared DEG and DAR in epigenetic regulation provided 7 genes (NKX1-2, BRPF3, SOX9, CBX6, RFX5, MXD4, DYRK1A), of which 28% function in histone modification, 9% in chromatin organization and 5% in chromatin remodeling (Fig. 4m, n and Supplementary Fig. 9f). To search for potential TF that bind to these differentially accessible chromatin regions, we conducted HOMER de novo motif discovery and known motif scanning in more and less accessible regions across both cell models. Fourteen common TF were identified in regions with increased accessibility, including FOSL2, BATF, and FRA2, while 20 TF were associated with decreased accessibility, including ELF4, KLF15, and NFY (Supplementary Fig. 9g, h and Supplementary Data S4). By merging predicted transcriptome regulatory gene datasets deduced from IPA with ATAC-seq motif analyses, we discovered 11 overlapping TF, with JUNB, FOSL1, and RELA being activated and only SPDEF being inhibited (Fig. 4o). Overall, integration of transcriptomic and ATAC-seq data indicates that ITGA7 knockdown leads to increased heterochromatin formation paralleled by reduced gene expression based on alterations in DNA accessibility and epigenetic regulation.

### ITGA7 depletion modulates protein phosphorylation and expression, particularly of cell cycle- and epigenetic-associated signaling mediators

**Fig. 5:**
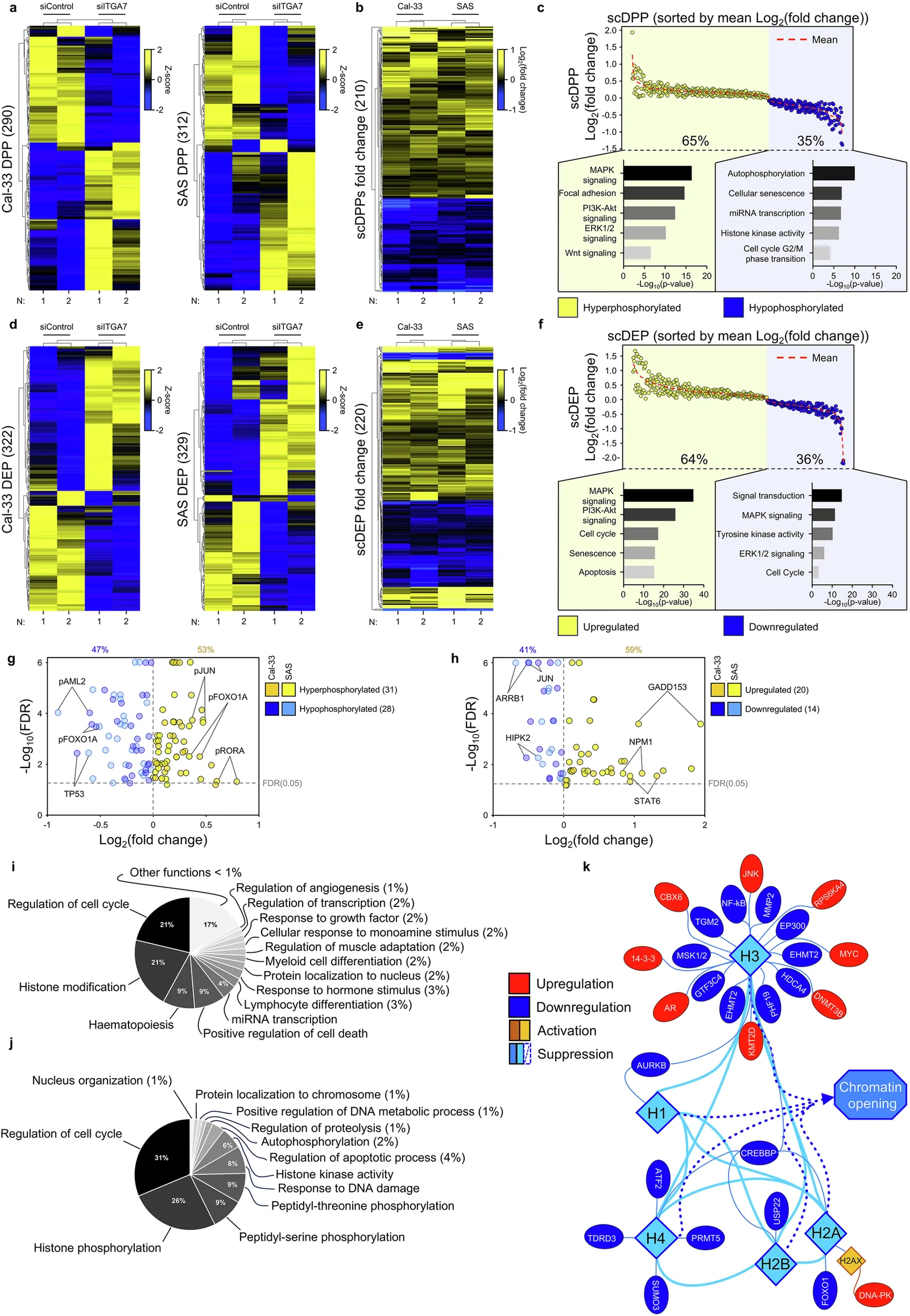
ITGA7 depletion modulates protein phosphorylation and expression in 3D HNSCC models. **a** Hierarchical clustering of changes of differentially phosphorylated proteins (DPP) specific to each cell model following ITGA7 depletion (*N* = 2; *p* < 0.05). **b** Hierarchical clustering of similarly changed differentially phosphorylated proteins (scDPP; *N* = 2; *p* < 0.05). **c** Waterfall of scDPP and associated ClueGO-based functional enrichment analysis on hyper- and hypophosphorylated proteins. **d** Hierarchical clustering of changes of differentially expressed proteins (DEP) specific to each cell model following ITGA7 depletion (*N* = 2; *p* < 0.05). **e** Hierarchical clustering of similarly changed differentially expressed proteins (scDEP; *N* = 2; *p* < 0.05). **f** Waterfall of scDEP and associated ClueGO-based functional enrichment analysis on up- and downregulated proteins. **g** Plotting of epigenetically associated scDPP from (**b**). **h** Plotting of epigenetically associated scDEP from (**e**). **i** ClueGO-based functional enrichment analysis for epigenetically associated scDPP from (**g**). **j** ClueGO-based functional enrichment analysis for epigenetically associated scDEP from (**h**). **k** scDEG, scDPP and scDEP interactive network from IPA specific for chromatin opening signaling pathway

Next, we investigated how α7 integrin mechanistically regulates the observed regulation of transcriptome and chromatin accessibility and examined 2010 proteins and their phosphorylation events by Kinex™ antibody microarray in whole cell lysates from 3D lrECM, ITGA7-depleted Cal-33 and SAS models (Fig. 3a and Supplementary Fig. 10a). We found 290 significantly altered, differentially phosphorylated proteins (DPP) upon ITGA7 depletion relative to controls (*p* ≤ 0.05; Fig. 5a; Supplementary Fig. 10b, c; and Supplementary Data S5). Canonical pathway analysis revealed similar activation of mTOR, EGF, STAT, and telomerase signaling pathways in the two cell models (Supplementary Fig. 10d and Supplementary Data S5). Decreased activities were seen for PPAR, ATM, PTEN, and CLEAR pathways. Shared upstream and master regulators included TP53, VEGFA, MUS81, and the mTORC1 complex (Supplementary Fig. 10e, f). Among the 210 similar changed DPP (scDPP) of both cell models, 136 (65%) showed increased and 74 (35%) decreased phosphorylation (Fig. 5b, c and Supplementary Data S5). Functional enrichment analyses identified hyperphosphorylated scDPP to predominantly associate with MAPK, FA, PI3K/AKT, ERK1/2 and Wnt signaling pathways, whereas hypophosphorylated scDPP were associated with autophosphorylation, cellular senescence, miRNA transcription, histone kinase activity and cell cycle pathways (Fig. 5c; Supplementary Fig. 10g–i; and Supplementary Data S5).

We next examined differentially expressed proteins (DEP) and found 322 DEP in Cal-33 and 329 in SAS models upon ITGA7 depletion compared to controls (*p* ≤ 0.05; Fig. 5d, Supplementary Fig. 10j, k; and Supplementary Data S5). IPA revealed similar changes in both cell models in activated (PI3K, RAC, cell cycle) and inhibited (BMP, PXR, GDNF) signaling axes (Supplementary Fig. 10l and Supplementary Data S5). Additionally, shared upstream and master regulators included SALL4, OGA, HDAC (family), FGFR3/4, ELF3, and CDC37 (Supplementary Fig. 10m, n and Supplementary Data S5). Upon identification of 220 similarly changed DEP (scDEP), 140 (64%) were upregulated and 80 (36%) downregulated (Fig. 5e, f and Supplementary Data S5). In line with the analysis of the phosphorylated forms, further bioinformatics revealed PPAR, PPARα/RXRα, JAK/STAT, PI3K/AKT, cell cycle, and apoptosis to be altered (Fig. 5f, Supplementary Fig. 10o; and Supplementary Data S5). Among more than 40 master regulators from scDEP, SALL4 and CDC37 were the only upstream regulators predicted to be significantly relevant (Supplementary Fig. 10p, q and Supplementary Data S5).

Building on our earlier findings that ITGA7 influences chromatin accessibility, we next examined epigenetic regulators within the (phospho)proteomic datasets. Analysis of scDPP identified 59 proteins linked to epigenetic regulation. Of these, 31 (53%) showed increased phosphorylation, while 28 (47%) showed decreased phosphorylation, with prominent examples including JUN, FOXO1A, and RORA (Fig. 5g and Supplementary Data S5). Overall, phosphorylation changes affected several transcriptional and chromatin-associated regulators, such as JUN, FOXO1A, RORA, AML2, and TP53. In parallel, analysis of scDEP identified 34 epigenetic regulators. Among these, 20 (59%; top examples: GADD153, STAT6, NPM1) were upregulated, while 14 (41%; top examples: JUN, ARRB1, HIPK2) were downregulated (Fig. 5h and Supplementary Data S5).

Pathway analysis of both scDPP and scDEP revealed strong enrichment for cell cycle regulation and histone modification processes (Fig. 5i, j). Notably, several chromatin-regulating proteins exhibited differential protein phosphorylation, indicating direct modulation of both chromatin-activating and chromatin-repressive mechanisms following ITGA7 depletion. For example, phosphorylation of the histone acetyltransferase CREBBP/CBP was reduced at S121/S124 by 24%, and phosphorylation of the MAPK-dependent kinase MSK1 (RPS6KA5) was reduced at S376 by 33%. CREBBP acts as a key transcriptional co-activator that acetylates proteins including histones, depositing acetylation marks such as H3K27ac, while MSK1 phosphorylates histone H3 (e.g., H3S10 and H3S28) during transcriptional activation. Reduced phosphorylation of these proteins indicates impaired enhancer activation and diminished histone modification.

Conversely, components of the chromatin-repressive machinery were also affected. Phosphorylation of HDAC4 at S632 was reduced by 19%, together with a 15% increase in phosphorylation of the SIN3A scaffold protein at S832, pointing toward enhanced HDAC-mediated chromatin silencing. Additional phosphorylation changes were observed in epigenetic enzymes such as KMT2C (MLL3) at S4267 and PRMT5 at T634. Further alterations were detected in chromatin-associated signaling pathways. A 25% decreased phosphorylation of FOXO1 at the canonical AKT target sites S256 and a 32% decrease at S319 indicates perturbed PI3K/AKT signaling downstream of ITGA7. Additional phosphorylation changes were observed in AMPKα1 (T183/S184) and WEE1 (S642). In contrast, expression of the histone chaperone NPM1 was significantly upregulated by 51%, while its phosphorylation changed (11% increase at S4; 16% reduction at T199), suggesting altered functional activity.

Integrating transcriptomic and (phospho)proteomic data revealed an overall suppression of chromatin-opening mechanisms in HNSCC following ITGA7 silencing, particularly through canonical histone modification pathways (Fig. 5k). Together, these findings identify chromatin-associated signaling mediators and epigenetic regulators that may underlie ITGA7-dependent transcriptional programs and changes in chromatin accessibility.

### Integrative multi-omics analysis reveals ITGA7 depletion impairs prosurvival and promitogenic pathways

**Fig. 6:**
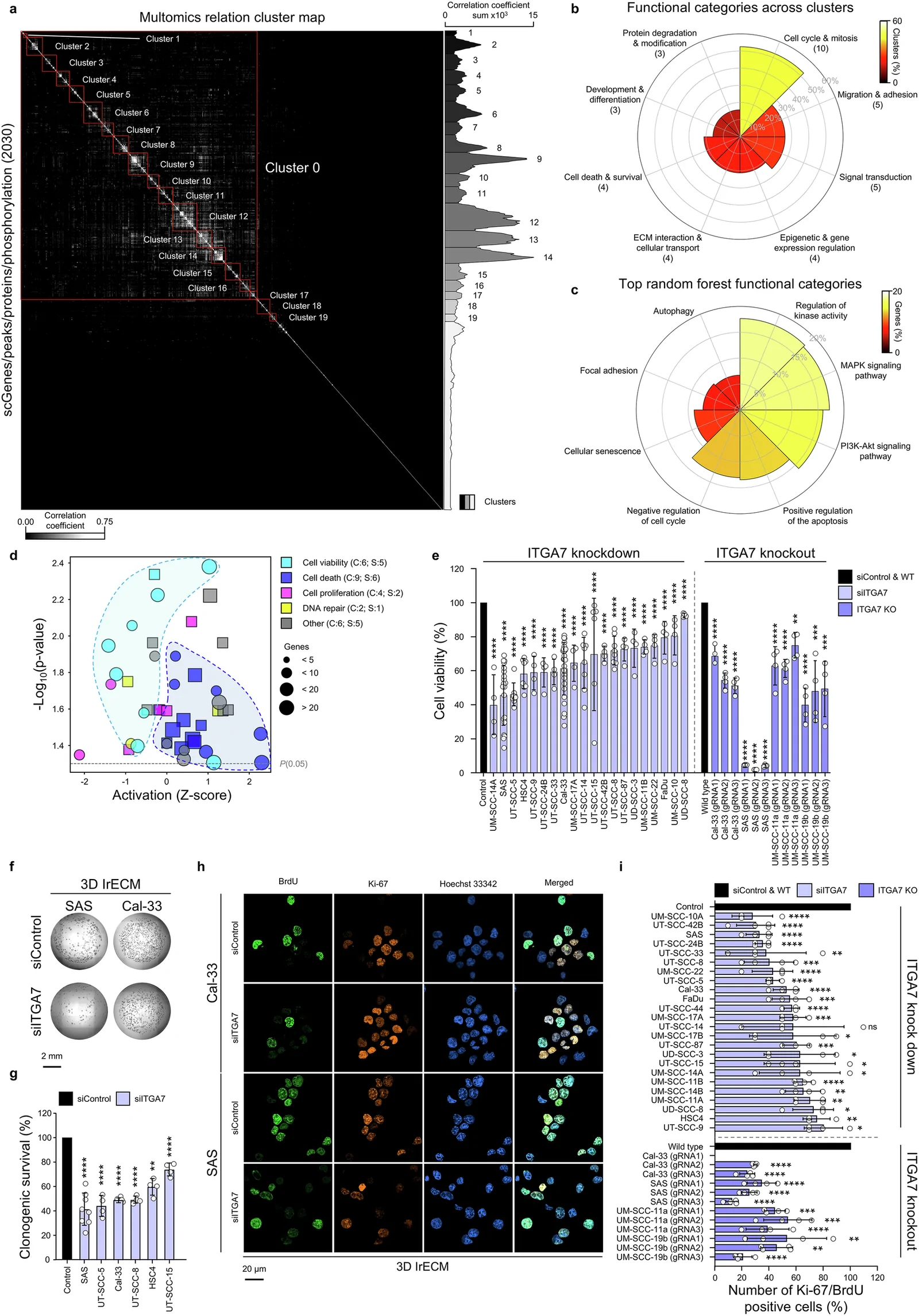
ITGA7 depletion molecularly and functionally affects cell viability and clonogenic survival of 3D lrECM HNSCC models. **a** Nexus correlation clustering analysis of similarly changed data (RNA-seq; ATAC-seq; protein phosphorylation/expression) interconnected via STRING. **b** ClueGO-based enrichment analysis of shared signatures across clusters from (**a**), grouped into eight main functional categories as indicated. **c** ClueGO-based enrichment analysis of all features from random forest analysis (Supplementary Data S6). **d** IPA-based analysis on scDPP from ITGA7-depleted cell models for disease and functional pathway identification. **e** Cell viability of 3D lrECM-grown HNSCC models following ITGA7 siRNA knockdown or CRISPR-Cas9 KO. Data are mean ± SD; *N* ≥ 4 independent biological replicates (each with ≥ 3 technical replicates). Statistical analysis: unpaired two-tailed *t*-test (knockdown; ***p < 0.001, ****p < 0.0001) and one-way ANOVA with Dunnett’s multiple-comparison test (knockout; **p_adj_ < 0.01, ***p_adj_ < 0.001, ****p_adj_ < 0.0001; all conditions versus relative wild type/control). **f** and **g** Clonogenicity of 3D lrECM HNSCC models upon ITGA7 depletion. Data are presented as mean ± standard deviation with a minimum sample size of *N* ≥ 4 (each independent biological replicate had *N* ≥ 3 technical replicates, unpaired *t* test; ****p < 0.0001). **h** Representative segmented images of Ki-67/BrdU-positive cells upon ITGA7 silencing. **i** Number of Ki-67/BrdU-positive cells after either ITGA7 knockdown or ITGA7 KO relative to controls (from h and Supplementary Fig. 12a). Data are mean ± standard deviation with *N* ≥ 3 independent biological replicates (×25 magnification; ≥ 100 nuclei analyzed per biological replicate per condition). Statistical analysis: unpaired two-tailed t-test (knockdown; ns *p* > 0.05, *p < 0.05, **p < 0.01, ***p < 0.001, ****p < 0.0001) and one-way ANOVA with Dunnett’s multiple-comparison test (knockout; **p_adj_ < 0.01, ***p_adj_ < 0.001, ****p_adj_ < 0.0001; all conditions versus relative wild type/control)

To explore whether the epigenetic, survival and cell cycle regulatory mechanisms consistently identified in our analyses could be robustly found in an integrative approach of all our omics datasets, we constructed a STRING-based, protein-protein interaction network with a confidence threshold of 75%. Shared interactors enabled a correlation cluster analysis that revealed clusters of similarly altered data with strong positive correlations, potentially serving as a functional multi-omics fusion analysis (Fig. 6a). This clustering resulted in a megacluster consisting of 17 subclusters plus two smaller clusters (Supplementary Data S6). Subsequent ClueGO enrichment analysis of shared signatures across clusters grouped them into 8 main functional categories (pathways) (Fig. 6b and Supplementary Data S6).

We extended this analysis for a validation approach by performing multi-omics random forest (RF) analyses. To ensure consistency, we focused on common data sets that exclusively contained significant and common data variables. After data normalization by log transformation and standard scaling, we trained an RF classifier to distinguish between ITGA7 knockdown and controls. The model achieved 100% classification accuracy across all datasets (precision, recall, f1 score, macro and weighted averages were all 1), demonstrating robust discrimination between control and ITGA7 knockdown profiles. To address the potential problem of overfitting due to high accuracy, we performed hyperparameter optimization using randomized search combined with stratified 4-fold cross-validation, optimizing parameters such as the number of estimators, maximum depth, feature selection thresholds, and class weights. The best model consistently achieved 100% cross-validation accuracy, indicating high robustness to parameter variations. Additionally, nested cross-validation was employed to obtain an unbiased estimate of the model’s performance (mean nested CV accuracy = 1.0 for all models). This included an inner loop for hyperparameter optimization and an outer loop for model evaluation, and the nested cross-validation accuracy remained at 100%. We also constructed learning curves to examine the model’s performance as a function of training set size (Supplementary Fig. 11a–d). The curves demonstrated that both training and cross-validation accuracies plateaued at 100% even on smaller subsets of the data, suggesting that the model did not benefit from memorization but captured true underlying patterns. Despite these measures, the model maintained high accuracy throughout, suggesting that the strong confidence in the separation between control and knockdown samples may not be an artifact of overfitting. To further validate the identified features in each dataset, we performed principal component analysis (PCA), which uncovered the first two principal components to effectively separate the samples based on their treatment conditions. The top genes contributing to these principal components overlapped with those identified by the RF model (Supplementary Fig. 11e–l), reinforcing their biological significance.

After all levels of random forest model optimization, we identified the top 100 DEG for the transcriptome dataset, including CAMKK2, SECISBP2, MMP14, RELA, TP53, and several MAPK members (Supplementary Fig. 11m and Supplementary Data S6). For chromatin accessibility data, some of the top features were GPRC5C, BTD, PTPRJ, IKBKB, CDK16, and MET (Supplementary Fig. 11n and Supplementary Data S6). In the phosphoproteomic data, RUNX3, TBXA2R, and SIN3A emerged as the three most important features, followed by TP53BP1, HDAC4, STAG2, BUB1B, FOXO3, MYC, YAP1 and CDK12 (Supplementary Fig. 11o and Supplementary Data S6). Finally, DEP were identified, with FGFR, PAK6, and CHUK representing the three most important features, followed by TP53, CDKN1B, CHEK2, CDK4, CENPA, TTK, MTOR, LATS2 and ATR (Supplementary Fig. 11p and Supplementary Data S6). Subsequent ClueGO analysis on combined features confirmed that α7 integrin appears to play a crucial role in the regulation of kinase activity (18%), MAPK (17%) and PI3K/AKT (16%) signaling pathways, positive regulation of apoptosis (13%), and negative regulation of cell cycle pathways (13%) (Fig. 6c). To estimate the consequences for HNSCC cell models after ITGA7 knockdown, we took the scDPP for IPA and found two main trends: (i) decrease in cell viability activity; (ii) increase in cell death pathways (Fig. 6d and Supplementary Data S6).

**Fig. 7:**
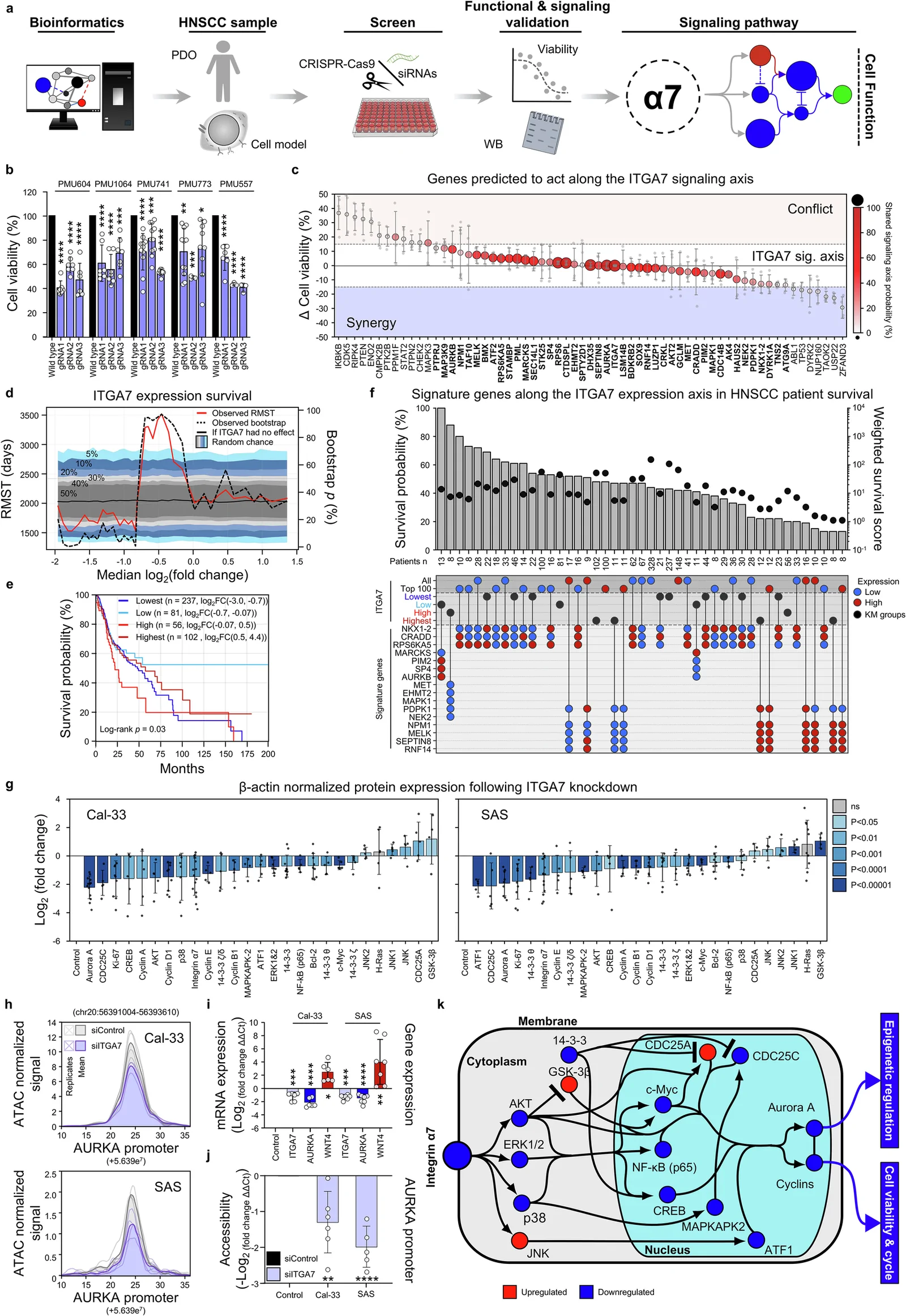
Loss of integrin α7 affects cell viability and survival via MAPK/AKT-AURKA pathway. **a** Schematic of the workflow. **b** Cell viability of 3D lrECM grown ITGA7 KO PDO with 3 different guide RNA (gRNAs). Data are presented as mean ± standard deviation with a minimum sample size of *N* ≥ 3 (each independent biological replicate had *N* ≥ 5 technical replicates, one-way ANOVA; Dunnett; *p_adj_ < 0.05; **p_adj_ < 0.01; ****p_adj_ < 0.0001; ns *p*_adj_ > 0.05; all conditions vs. relative wild type control). **c** Differential cell viability changes upon single and double knockdown relative to ITGA7 depletion, ranked by mean Δ cell viability. The dashed line indicates the ±15% Δ cell viability threshold of the single ITGA7 single-knockdown effect (genes selected marked bold). Data are presented as mean ± standard deviation (*N* ≥ 4 biological replicates; grey dots). The color of each point (shades of red) represents the genes shared signaling axis probability with ITGA7 (ΔPathway Score; sigmoidal transformation of the epistasis score; *T* = 0.05, *k* = 3). **d** Sliding-window RMST across ITGA7 expression (left axis; red line). Patients ordered by ITGA7 log_2_ (fold change tumor vs healthy control). Survival time (*τ*) per window size/step size (e.g., 80 patients, step 10) was set to the maximum observed time within the window (or equivalently the local maximum used for integration of the Kaplan–Meier survival function). No association between ITGA7 and survival while preserving the survival data distribution indicated with solid black line. Shaded bands denote central percentile ranges of the null distribution, indicating random chance probability. The dashed black line (right *y*-axis) represents the bootstrap dominance probability, defined as the proportion of bootstrap resamples in which the observed RMST in a given window exceeded the corresponding RMST under the permutation null (ΔRMST > 0). **e** Kaplan-Meier survival curves for ITGA7 expression-bas**e**d grouping. Patients were ordered by tumor ITGA7 log2 fold change and partitioned into four bins using scan-optimized cutpoints (log_2_ fold lowest (−3.0, −0.7); low (−0.7, −0.07), high (−0.07, 0.5), and highest (0.5, 4.4) expression ITGA7). Survival differences were assessed using the multivariate log-rank test (overall *p* = 0.03). **f** Kaplan–Meier survival and weighted survival score across significant (*p* < 0.05) ITGA7-defined subgroup combinations. Bars indicate Kaplan-Meier survival probability at the indicated time point (left *y*-axis). Dots indicate a weighted survival score, defined as KM survival probability multiplied by number of patients in subgroup, shown on a logarithmic scale (right y-axis). Numbers between panels denote number of patients per subgroup. The lower matrix summarizes subgroup composition: each column represents a group, and rows indicate ITGA7 selection factor (All patients divided by ITGA7 expression to 0 > and < 0, “top 100” takes top 100 low/high extreme expression patients), ITGA7 expression bins (from e, black dots), and signature gene from c. For gene rows, dot color encodes state (red, high; blue, low). **g** Densitometry of western blot shown in Supplementary Fig. 13a. β-actin normalized protein expression after ITGA7 knockdown relative to control in cell models. Data are presented as mean ± standard deviation with a minimum sample size of *N* ≥ 4 (unpaired *t*-test; ns > 0.05, *p* < 0.5–0.0001). **h** ATAC-normalized signal enrichment in the AURKA promoter region in ITGA7-silenced cell models (*N* = 4; Cal-33, top; SAS, bottom). **i** RNA expression fold change upon ITGA7 silencing relative to control. Data are presented as mean ± standard deviation (*N* ≥ 4; one-way ANOVA; Dunnett’s test; *p_adj_ < 0.05; **p_adj_ < 0.01; ***p_adj_ < 0.001; ****p_adj_ < 0.0001; all conditions vs. respective control). **j** AURKA promoter region chromatin accessibility fold change upon ITGA7 silencing relative to control. Data are presented as mean ± standard deviation (*N* ≥ 4; unpaired *t*-test; **p_adj_ < 0.01; ****p_adj_ < 0.0001). **k** Schematic of how ITGA7 signaling regulates chromatin accessibility and transcription through an AURKA-centered axis integrating adhesion cues with cell cycle control

These findings cumulatively prompted us to investigate clonogenicity, proliferation, and cell viability in a large panel of 3D, lrECM-based HNSCC cell models with a depletion or knockout of ITGA7 (Supplementary Table S1). Intriguingly, we observed a significant decrease in all of the functional readouts (Fig. 6e–i). Twenty-five cell models and HNSCC PDO showed a significant reduction in cell viability by 20–60% and 30–90% upon ITGA7 knockdown and knockout, respectively, which was confirmed by clonogenic survival (for cell models) and Ki67/BrdU proliferation assays (Figs. 6f–i and 7a, b; Supplementary Fig. 11q and Supplementary Fig. 12a, b). Taken together, these results demonstrate a crucial functional role of ITGA7 in mitogenesis and cell survival, thus confirming our predictive computational analyses.

### α7 integrin defines a clinically relevant signaling axis controlling HNSCC cell viability

To define the signaling network underlying the observed functional effects of ITGA7 depletion, we combined random forest (RF) feature selection with network-based correlation clustering (Fig. 6), identifying 63 candidate genes associated with the ITGA7 signaling axis (Supplementary Data S7). Experimental validation using a targeted RNAi screen in 3D-grown Cal-33 models (Fig. 7a) assessed single and combinatorial knockdowns with ITGA7 based on delta (Δ) cell viability scores, reflecting similarity to ITGA7 depletion. Using a <15% similarity threshold, 42 of 63 candidates phenocopied ITGA7 knockdown. Among these, seven genes (AURKA, EHMT2, SEPTIN8, SPTY2D1, LSM14B, BDKRB2, SOX9) showed no additional effect upon co-silencing, indicating their involvement in a shared signaling axis. To further refine pathway contribution, we calculated a ΔPathScore (Supplementary Methods), identifying 15 genes with ~50% probability of belonging to ITGA7 axis. Across all analytical approaches, AURKA consistently ranked highest, showing 90–99% similarity to ITGA7 depletion (Fig. 7c and Supplementary Data S7). Independent linear regression analysis identified 24 genes closely aligned (<15% deviation) with ITGA7-dependent effects, further supporting AURKA as a central node in α7 integrin signaling (Supplementary Fig. 12c).

We next examined the clinical relevance of this signaling axis. Given the complex distribution of ITGA7 expression in tumors (Fig. 2), we avoided predefined high/low cutoffs and instead applied a non-linear rank-based survival analysis. TCGA patient data were processed and ranked according to tumor ITGA7 log_2_ fold change (tumor vs normal), and overall survival (OS) was evaluated using overlapping sliding windows (80 patients per window, step size 10; Fig. 7d, Supplementary Fig. 12d). Within each window, the restricted mean survival time (RMST) was calculated up to a window-specific maximum follow-up time (*τ*). RMST showed a pronounced non-linear distribution across ITGA7 expression, with patients in the intermediate low expression range (~−0.7 to −0.07) showing significantly improved survival compared to adjacent groups and permutation-derived null models (2000 permutations). The observed RMST peak exceeded the upper bounds of the permutation null bands, indicating that this localized survival enrichment is unlikely to arise by random chance. To evaluate stability, we performed bootstrap-based dominance probability analysis (2000 resamples). For each window, ΔRMST (RMST_window_ - RMST_rest_) was recalculated across bootstrap iterations. The bootstrap-based dominance probability analysis confirmed the robustness of this survival advantage, with probabilities approaching 100%, whereas both expression extremes showed no consistent benefit.

To translate this continuous survival pattern into discrete patient groups, we performed systematic Kaplan-Meier analyses for all possible 4-bin splits (Supplementary Fig. 12e, f). This revealed non-random clustering of optimal cut points at specific ITGA7 expression, confirming stable survival transition zones (Supplementary Fig. 12e, f). The majority of hits recapitulated the non-linear survival structure observed in Fig. 7d. Most significant combinations of Kaplan–Meier survival probability demonstrated that survival is not increased steadily with decreasing or increasing ITGA7 levels. Notably, the intermediate low ITGA7 group (−0.7 to −0.07) exhibited the most favorable survival, whereas both lower (−3.0 to −0.7) and higher (0.5–4.4) expression extremes were associated with poorer outcomes. Pairwise log-rank testing confirmed significant separation (*p* = 0.03; strongest *p* = 0.01), indicating that survival differences are confined to defined expression intervals.

Given this interval-specific survival pattern, we next tested whether predicted ITGA7-axis genes (Fig. 7c) support survival differences within ITGA7-defined patient groups. Patients were subdivided within each ITGA7-defined cohort according to the median expression of individual signature genes, and 5 year Kaplan–Meier OS probabilities were calculated. Genes reaching statistical significance (*p* < 0.05) were clustered to evaluate combinatorial survival effects (Fig. 7f). To account for subgroup size and avoid overinterpreting small subsets with high survival probabilities, we implemented a weighted survival score (KM probability × subgroup size). Reduced ITGA7 expression consistently associated with improved survival. Within the low ITGA7 cohort, the gene signatures with MARCKS, PIM2, SP4, and AURKB showed the highest survival (100%), whereas in the high ITGA7 group, MET, EHMT2, MAPK1, PDPK1, and NEK2 were associated with >80% survival. NKX1-2, CRADD, and RPS6KA5 demonstrated consistent survival associations across groups, suggesting context-independent effects.

To mechanistically validate this clinically supported axis, we profiled AKT/MAPK, cell cycle, and Aurora signaling in Cal-33 and SAS models following ITGA7 knockdown (Fig. 7g, Supplementary Fig. 13a-b and Supplementary Data S7). Both models showed concordant reductions in Aurora A, CDC25C, Ki-67, cyclins, and α7 integrin, along with decreased AKT and ERK1/2 signaling. Phosphorylation of AKT (Ser473), FOXO1 (Ser256), and Aurora A (Thr288) was markedly reduced. In contrast, stress-response pathways were activated, including increased JNK1/2 expression, enhanced c-JUN and ATF2 phosphorylation, and activation of p38 MAPK signaling. Moreover, GSK-3β activity increased, consistent with a shift toward anti- mitogenic signaling. Across all datasets, AURKA emerged as the most consistent and dominant node within the ITGA7-dependent network.

Consistent with this, ATAC-seq revealed reduced chromatin accessibility at the AURKA promoter following ITGA7 silencing (−23% in Cal-33; −39% in SAS; Fig. 7h), with independent assays showing stronger reductions (−60% and −70%, respectively; Fig. 7j). Correspondingly, AURKA mRNA expression decreased by 54% (Cal-33) and 74% (SAS; Fig. 7i). In contrast, WNT4 expression increased markedly (~400% in Cal-33; ~900% in SAS), confirming selective regulatory effects.

Finally, integration of transcriptomic, epigenetic, proteomic, phosphoproteomic and functional data enabled reconstruction of a coherent α7 integrin signaling axis (Fig. 7a, k and Supplementary Fig. 14a). This integrated model links adhesion signaling to coordinated regulation of AKT/MAPK, cell cycle, and Aurora kinase pathways, positioning AURKA as a central mediator connecting ITGA7 to chromatin state and cell viability. Notably, genes functionally aligned with ITGA7 depletion also stratified patient survival, demonstrating direct convergence between mechanistic biology and clinical outcome. Together, these findings establish α7 integrin as a determinant of biologically distinct tumor states and identify the ITGA7-AURKA axis as a clinically relevant and potentially targetable vulnerability in HNSCC.

## Discussion

Understanding how extracellular signals directly influence chromatin organization, accessibility, and transcriptional competence remains a central challenge in cancer biology. Importantly, despite their known autonomous properties, cancer cells strongly depend on environmental cues such as extracellular matrix, nutrients, hormones, and hypoxia, regulating processes including cancer stem cell niche organization, immune cell infiltration, and sustained angiogenesis during tumor progression.^7,9,13,34^ While integrins are well-known determinants of cell survival, mechanotransduction, and therapy resistance, their role in epigenetic control has remained largely indirect and to be defined. Here, we demonstrate α7 integrin (ITGA7) as a previously unrecognized regulator of chromatin accessibility and transcription, establishing a direct mechanistic link between cell adhesion signaling and epigenetic regulation in HPV-negative HNSCC. These findings position cell adhesion receptors not merely as upstream signaling components but as active determinants of the epigenetic landscape.

A central advance of our study is the demonstration that ITGA7 functionally drives chromatin state transitions. Multi-omics analyses revealed that ITGA7 depletion induces a general shift toward a repressive chromatin configuration, characterized by increased heterochromatin organization, reduced promoter accessibility, and transcriptional downregulation. These molecular changes translated into consistent phenotypic effects, including decreased viability, reduced clonogenic survival, and impaired proliferation across multiple 3D HNSCC models and patient-derived organoids, establishing ITGA7 as a regulator of tumor cell fitness in a physiologically relevant microenvironment. Importantly, ITGA7 was perturbed by two mechanistically independent modalities, i.e., RNA interference and CRISPR/Cas9 knockout, which produced convergent chromatin, transcriptional, and phenotypic outcomes across multiple genetically, epigenetically, and phenotypically diverse models and patient-derived organoids. This concordance across orthogonal reagents controls for reagent-specific off-target effects and indicates that the observed phenotypes reflect on-target loss of ITGA7.^35^

Genetic and pharmacological perturbation of transmembrane receptors has demonstrated that growth factor receptors and their downstream signaling mediators, mechanotransduction-associated cytoskeletal components, and ion channels contribute to genome organization, chromatin accessibility, and gene transcription.^26,34,36,37^ In line with this, extracellular cues are integrated through signaling networks that converge on chromatin regulation. In addition to transcription factors, histone post-translational modifications, and chromatin-modifying enzymes, survival, proliferation, differentiation, and migration critically depend on the integration of extra- and intracellular biochemical as well as mechanical signals. Epigenetic plasticity makes dynamic adaptation of transcriptional programs in response to these inputs feasible, underpinning the fact that genetic and epigenetic alterations cannot be fully understood in isolation but must take into account the environmental context. Prominent examples include cell-ECM interactions and growth in three-dimensional extracellular matrix environments, both of which profoundly influence chromatin organization, gene expression, DNA repair, and responsiveness to anticancer therapies.^7,19,30,38–41^ However, a direct epigenetic regulatory function of cell adhesion molecules remains largely to be clarified. Our findings extend the existing body of literature by identifying α7 integrin as a key upstream regulator linking adhesion signaling to epigenetic regulation and gene transcription.

To mechanistically resolve these observations, we identified a signaling-chromatin axis centered on AURKA, a master regulator of mitotic progression integrating signals controlling centrosome dynamics, spindle assembly, and cell proliferation.^42,43^ ITGA7 depletion reduced chromatin accessibility at the AURKA locus, resulting in decreased transcription and protein expression paralleled by diminished cell cycling and survival. This multi-layer validation uncovered a direct link between adhesion signaling, chromatin remodeling, and cellular phenotype, positioning AURKA as a key downstream effector of integrin-dependent epigenetic regulation.

At the molecular level, ITGA7 signaling converges on the chromatin-modifying machinery. Multiple epigenetic regulators were altered upon ITGA7 silencing, including HMGA2 and MXD4,^44,45^ as well as chromatin-associated enzymes such as EHMT2, CBX6, PHC1, BRPF3, and DYRK1A^46–54^. The global reduction in chromatin accessibility at transcription start sites and concordant transcriptional repression indicate a shift toward a more compact chromatin status.

In parallel, transcription factor networks were affected showing an enrichment of RELA, SPDEF, ATF4, and AP-1 components (JUN, FOSL1, JUNB),^28,55^ thereby suggesting disrupted transcription factor-mediated chromatin regulation. Both cell models investigated demonstrated consistently reduced phosphorylation of CREBBP and MSK1, indicating confined activation of chromatin-opening mechanisms, whereas altered phosphorylation of HDAC4 and SIN3A collectively supports enhanced chromatin-repressive activity.^56–61^ In addition, decreased FOXO1 phosphorylation points to attenuated PI3K/AKT signaling, further linking adhesion signaling to epigenetic pathways. Together, these findings indicate that ITGA7 signaling coordinates multiple chromatin-regulatory complexes including histone methyltransferases, Polycomb components, and acetylation regulators to control the balance between chromatin accessibility and compaction.

Notably, ITGA7 exhibits a context-dependent relationship with tumor progression and patient outcome. Although its expression decreases during the dedifferentiation of normal tissue to carcinoma, functional analyses demonstrate that ITGA7 is inevitable for tumor cell survival and proliferation in certain contexts, which need to be defined in detail. Consequently, we speculate that ITGA7 acts as a tumor suppressor in HPV-negative HNSCC. ITGA7 expression progressively decreases with increasing pressure of malignant transformation, similar to a Red Queen-like dynamic, with higher malignancy associated with lower ITGA7 levels. Future studies are needed to investigate this hypothesis in detail. The fact that ITGA7 expression and patient survival are in a non-linear relationship is also reflected in the patient data. These data demonstrate a non-linear relationship between ITGA7 expression and overall survival. Intermediate expression levels suggest that these are associated with a more favorable prognosis, while both low and high levels correlate with a poorer prognosis. Our findings suggest that ITGA7 defines different tumor stages rather than functioning as a linear biomarker.

The present study was deliberately restricted to HPV-negative HNSCC. In HPV-positive disease, the viral oncoproteins E6 and E7 dominate epigenetic regulation through upregulating DNA methyltransferases and driving a globally distinct, hypermethylated landscape, and their expression is sufficient to reprogram the DNA-methylation profile of HPV-negative cells.^31,62^ Including HPV-positive models in the present study would therefore superimpose a strong, virus-driven epigenetic program that would likely mask the more specific chromatin adhesion signal under investigation; thus, isolating the HPV-negative subtype is a prerequisite for specifically attributing chromatin changes to ITGA7. HPV-negative HNSCC is, moreover, the subtype of greatest unmet clinical need, being more therapy-refractory and carrying a worse prognosis than HPV-positive oropharyngeal carcinoma.^63^ Whether HPV-driven tumors retain or bypass ITGA7-dependent chromatin regulation is an important question that we will address in a dedicated follow-up study.

From a translational perspective, the ITGA7-AURKA axis may present a strategically elegant, therapeutically actionable signaling module as it integrates adhesion cues, cell cycling, and chromatin regulation. The convergence of our multi-omics data, functional validation, and patient analyses underscores its biological and clinical relevance. These results provide an excellent basis for therapeutic strategies targeting the disruption of adhesion-dependent chromatin regulation. Given the complexity, feedback mechanisms, and evasion possibilities of ITGA7-AURKA signaling, future studies must elucidate in detail the functional effectiveness and hierarchical vulnerability of the numerous potential targets of this signaling cascade using genetic and pharmacological intervention approaches. In this regard, no selective pharmacological inhibitor of α7β1 integrin currently exists, and clinically available integrin-directed agents target distinct heterodimers; pan-integrin inhibition would engage multiple receptors simultaneously and preclude clean attribution to ITGA7. The most immediately actionable node emerging from this mechanism is therefore the downstream effector AURKA, for which Aurora-A inhibitors such as alisertib are in clinical development, whereas selective targeting of the ITGA7 pathway itself remains contingent on the development of selective compounds.

Although in vivo validation and modulation of upstream mechanical cues remain important next steps, the use of 3D matrix-based models and patient-derived organoids provides a physiologically highly relevant and clinically translatable system capturing key aspects of tumor microenvironment signaling.^64,65^ According to our observations, ITGA7 depletion alone is sufficient to reprogram chromatin accessibility and tumor cell behavior, demonstrating a direct causal link between adhesion signaling and epigenetic regulation. Integrin-ECM engagement in three-dimensional laminin-rich matrix has been shown to govern tumor cell survival and the malignant phenotype as well as to predict outcomes subsequently confirmed in vivo. Hence, this system was historically validated precisely based on its capacity to recapitulate integrin biology in tissue.^66–68^ In HNSCC specifically, patient-derived organoid drug and radiotherapy responses mirror the clinical outcomes of the matched patients.^64,69,70^ We nonetheless acknowledge, as a limitation, the absence of orthotopic and immune-competent in vivo models. These represent the logical next step toward therapeutic development but are not required to support the mechanistic conclusions established here across three complementary human systems and corroborated by patient survival data.

In summary, this study establishes α7 integrin (ITGA7) as a causal co-regulator of chromatin accessibility and gene expression in HPV-negative HNSCC. Loss of ITGA7, achieved independently by CRISPR/Cas9 knockout and RNA interference across multiple 3D matrix-embedded HNSCC models and patient-derived organoids, suppresses MAPK- and PI3K/AKT-dependent signaling, reduces chromatin accessibility at the AURKA locus, downregulates the AURKA-centered transcriptional program, and impairs cell cycle progression and survival. Because tumor ITGA7 expression stratifies patient overall survival, yet ITGA7 itself is not currently druggable, its downstream effector AURKA, targetable with clinical Aurora-A inhibitors such as alisertib, constitutes an immediately actionable therapeutic entry point in this therapy-refractory subtype. More broadly, these findings demonstrate that integrin-mediated adhesion directly co-determines the epigenetic and transcriptional landscape of cancer cells.

## Materials and methods

The data, methods, and study materials are available from the corresponding author upon reasonable request. Expanded Methods are provided in the Supplemental Materials.

### Data collection and processing

This section encompasses public data collection and preprocessing, methylation data, HNSCC population mutation, and single-cell omics data, etc. Comprehensive details of these procedures are provided in the Supplemental Materials.

### Bioinformatics and computational methods

Included methods describe network and reconstruction, methylation data, gene ontology and functional, patient survival, image, K-means clustering, HNSCC population mutation, single-cell omics data, RNA sequencing analyses, TF motif identification, multi-omics clustering, machine learning and feature selection, and diverse programming languages. Further details can be found in the Supplemental Materials.

### Human studies and patient survival analysis

This category covers human studies, patient survival analysis, and H&E, immunohistochemistry, and immunofluorescence staining of patient material. Detailed methodologies can be found in the Supplemental Materials.

### Cell models, 3D cell culture and patient-derived HNSCC organoids

Methods in this section include the use of cell models, patient-derived HNSCC organoids, 3D cell culture techniques, 3D cell viability, colony formation, and proliferation assays. Additional details are provided in the Supplemental Materials.

### Experimental procedures

This section details RNAi, histone extraction and dot blot, immunofluorescence staining and microscopy, RNA extraction, RT-qPCR, nuclei isolation, RNA/ATAC sequencing, Kinex™ antibody microarray and chromatin accessibility assay. Detailed protocols are provided in the Supplemental Materials.

### Statistical analysis

All statistical analysis procedures utilized in this study are described in detail within the Supplemental Materials.

## Supplementary information

Supplemental Material - clean

Supplementary_Data_S8

Supplementary_Data_S1

Supplementary_Data_S2

Supplementary_Data_S3

Supplementary_Data_S4

Supplementary_Data_S5

Supplementary_Data_S6

Supplementary_Data_S7

Supplementary Tables 1

Suppl. Video 1

Suppl. Video 2

Suppl. Video 3

Suppl. Video 4

Suppl. Video 5

Suppl. Video 6

Suppl. Video 7

Suppl. Video 8

## Data Availability

The data supporting the findings of this study are available within the Article and its Supplementary Information files. The raw RNA-seq and ATAC-seq data generated in this study have been deposited in the Gene Expression Omnibus (GEO) under accession numbers GSE338805 and GSE338979, respectively, and are publicly available. Any additional information required to interpret the data is available from the corresponding author upon reasonable request.

## References

[1] Karin, M. Signal transduction and gene control. *Curr. Opin. Cell Biol.* **3**, 467–473 (1991). 10.1016/0955-0674(91)90075-a 1832548

[2] Esteller, M. et al. The epigenetic hallmarks of cancer. *Cancer Discov.* **14**, 1783–1809 (2024). 10.1158/2159-8290.CD-24-0296 39363741

[3] Swanton, C. et al. Embracing cancer complexity: hallmarks of systemic disease. *Cell* **187**, 1589–1616 (2024). 10.1016/j.cell.2024.02.009 PMC12077170 38552609

[4] Jin, M.-Z. & Jin, W.-L. The updated landscape of tumor microenvironment and drug repurposing. *Signal. Transduct. Target Ther.* **5**, 166 (2020). 10.1038/s41392-020-00280-x PMC7447642 32843638

[5] Hayes, J. D., Dinkova-Kostova, A. T. & Tew, K. D. Oxidative stress in cancer. *Cancer Cell* **38**, 167–197 (2020). 10.1016/j.ccell.2020.06.001 PMC7439808 32649885

[6] Spangle, J. M., Roberts, T. M. & Zhao, J. J. The emerging role of PI3K/AKT-mediated epigenetic regulation in cancer. *Biochim. Biophys. Acta Rev. Cancer* **1868**, 123–131 (2017). 10.1016/j.bbcan.2017.03.002 PMC5548615 28315368

[7] Hanahan, D. & Weinberg, R. A. Hallmarks of cancer: the next generation. *Cell* **144**, 646–674 (2011). 10.1016/j.cell.2011.02.013 21376230

[8] Chastney, M. R., Kaivola, J., Leppänen, V.-M. & Ivaska, J. The role and regulation of integrins in cell migration and invasion. *Nat. Rev. Mol. Cell Biol.* 10.1038/s41580-024-00777-1 (2024). 39349749

[9] Cox, T. R. The matrix in cancer. *Nat. Rev. Cancer* **21**, 217–238 (2021). 10.1038/s41568-020-00329-7 33589810

[10] Haake, S. M., Rios, B. L., Pozzi, A. & Zent, R. Integrating integrins with the hallmarks of cancer. *Matrix Biol.* **130**, 20–35 (2024). 10.1016/j.matbio.2024.04.003 PMC13078519 38677444

[11] Eke, I. & Cordes, N. Focal adhesion signaling and therapy resistance in cancer. *Semin. Cancer Biol.* **31**, 65–75 (2015). 10.1016/j.semcancer.2014.07.009 25117005

[12] Sun, Z., Costell, M. & Fässler, R. Integrin activation by talin, kindlin and mechanical forces. *Nat. Cell Biol.* **21**, 25–31 (2019). 10.1038/s41556-018-0234-9 30602766

[13] Cooper, J. & Giancotti, F. G. Integrin signaling in cancer: mechanotransduction, stemness, epithelial plasticity, and therapeutic resistance. *Cancer Cell* **35**, 347–367 (2019). 10.1016/j.ccell.2019.01.007 PMC6684107 30889378

[14] Eke, I. et al. β₁ Integrin/FAK/cortactin signaling is essential for human head and neck cancer resistance to radiotherapy. *J. Clin. Investig.* **122**, 1529–1540 (2012). 10.1172/JCI61350 PMC3314473 22378044

[15] Dickreuter, E. et al. Targeting of β1 integrins impairs DNA repair for radiosensitization of head and neck cancer cells. *Oncogene* **35**, 1353–1362 (2016). 10.1038/onc.2015.212 26073085

[16] Broghammer, F. et al. Resistance of HNSCC cell models to pan-FGFR inhibition depends on the EMT phenotype associating with clinical outcome. *Mol. Cancer* **23**, 39 (2024). 10.1186/s12943-024-01954-8 PMC10880239 38378518

[17] Franovic, A. et al. Glioblastomas require integrin αvβ3/PAK4 signaling to escape senescence. *Cancer Res.* **75**, 4466–4473 (2015). 10.1158/0008-5472.CAN-15-0988 PMC4631634 26297735

[18] Tang, Z. et al. LncRNA SSTR5-AS1 promotes esophageal carcinoma through regulating ITGB6/JAK1/STAT3 signaling. *Epigenomics* **16**, 1133–1148 (2024). 10.1080/17501911.2024.2388018 PMC11457597 39234955

[19] Christmann, M. et al. Integrin αVβ3 silencing sensitizes malignant glioma cells to temozolomide by suppression of homologous recombination repair. *Oncotarget* **8**, 27754–27771 (2017). 10.18632/oncotarget.10897 PMC5438606 27487141

[20] Baylin, S. B. & Jones, P. A. A decade of exploring the cancer epigenome - biological and translational implications. *Nat. Rev. Cancer* **11**, 726–734 (2011). 10.1038/nrc3130 PMC3307543 21941284

[21] Kouzarides, T. Chromatin modifications and their function. *Cell* **128**, 693–705 (2007). 10.1016/j.cell.2007.02.005 17320507

[22] Tessarz, P. & Kouzarides, T. Histone core modifications regulating nucleosome structure and dynamics. *Nat. Rev. Mol. Cell Biol.* **15**, 703–708 (2014). 10.1038/nrm3890 25315270

[23] Alexandrov, L. B. et al. The repertoire of mutational signatures in human cancer. *Nature* **578**, 94–101 (2020). 10.1038/s41586-020-1943-3 PMC7054213 32025018

[24] van Haaften, G. et al. Somatic mutations of the histone H3K27 demethylase gene UTX in human cancer. *Nat. Genet.* **41**, 521–523 (2009). 10.1038/ng.349 PMC2873835 19330029

[25] Deb, M., Sengupta, D. & Patra, S. K. Integrin-epigenetics: a system with imperative impact on cancer. *Cancer Metastasis Rev.* **31**, 221–234 (2012). 10.1007/s10555-011-9341-9 22160755

[26] Vivo, M., Rosti, V., Cervone, S. & Lanzuolo, C. Chromatin plasticity in mechanotransduction. *Curr. Opin. Cell Biol.* **88**, 102376 (2024). 10.1016/j.ceb.2024.102376 38810318

[27] Park, S.-M. et al. Cyclic stretch promotes cellular reprogramming process through cytoskeletal-nuclear mechano-coupling and epigenetic modification. *Adv. Sci.* **10**, e2303395 (2023). 10.1002/advs.202303395 PMC10646259 37727069

[28] Bianchi, E. et al. Integrin LFA-1 interacts with the transcriptional co-activator JAB1 to modulate AP-1 activity. *Nature* **404**, 617–621 (2000). 10.1038/35007098 10766246

[29] Shi, C., Zhang, X., Chen, Z., Robinson, M. K. & Simon, D. I. Leukocyte integrin Mac-1 recruits toll/interleukin-1 receptor superfamily signaling intermediates to modulate NF-kappaB activity. *Circ. Res.* **89**, 859–865 (2001). 10.1161/hh2201.099166 11701612

[30] Storch, K. et al. Three-dimensional cell growth confers radioresistance by chromatin density modification. *Cancer Res.* **70**, 3925–3934 (2010). 10.1158/0008-5472.CAN-09-3848 20442295

[31] Cosper, P. F., Bradley, S., Luo, L. & Kimple, R. J. Biology of HPV mediated carcinogenesis and tumor progression. *Semin. Radiat. Oncol.* **31**, 265–273 (2021). 10.1016/j.semradonc.2021.02.006 PMC8409095 34455982

[32] Klapproth, E. et al. Whole exome sequencing identifies mTOR and KEAP1 as potential targets for radiosensitization of HNSCC cells refractory to EGFR and β1 integrin inhibition. *Oncotarget* **9**, 18099–18114 (2018). 10.18632/oncotarget.24266 PMC5915060 29719593

[33] Choi, J.-H. et al. Single-cell transcriptome profiling of the stepwise progression of head and neck cancer. *Nat. Commun.* **14**, 1055 (2023). 10.1038/s41467-023-36691-x PMC9958029 36828832

[34] Seguin, L., Desgrosellier, J. S., Weis, S. M. & Cheresh, D. A. Integrins and cancer: regulators of cancer stemness, metastasis, and drug resistance. *Trends Cell Biol.* **25**, 234–240 (2015). 10.1016/j.tcb.2014.12.006 PMC4380531 25572304

[35] Hart, T., Brown, K. R., Sircoulomb, F., Rottapel, R. & Moffat, J. Measuring error rates in genomic perturbation screens: gold standards for human functional genomics. *Mol. Syst. Biol.* **10**, 733 (2014). 10.15252/msb.20145216 PMC4299491 24987113

[36] Mahajan, K. & Mahajan, N. P. Cross talk of tyrosine kinases with the DNA damage signaling pathways. *Nucleic Acids Res.* **43**, 10588–10601 (2015). 10.1093/nar/gkv1166 PMC4678820 26546517

[37] Raynal, N. J.-M. et al. Targeting calcium signaling induces epigenetic reactivation of tumor suppressor genes in cancer. *Cancer Res.* **76**, 1494–1505 (2016). 10.1158/0008-5472.CAN-14-2391 PMC4794357 26719529

[38] Stowers, R. S. et al. Matrix stiffness induces a tumorigenic phenotype in mammary epithelium through changes in chromatin accessibility. *Nat. Biomed. Eng.* **3**, 1009–1019 (2019). 10.1038/s41551-019-0420-5 PMC6899165 31285581

[39] Pfeifer, C. R., Alvey, C. M., Irianto, J. & Discher, D. E. Genome variation across cancers scales with tissue stiffness - an invasion-mutation mechanism and implications for immune cell infiltration. *Curr. Opin. Syst. Biol.* **2**, 103–114 (2017). 10.1016/j.coisb.2017.04.005 PMC5654613 29082336

[40] Le Beyec, J. et al. Cell shape regulates global histone acetylation in human mammary epithelial cells. *Exp. Cell Res.* **313**, 3066–3075 (2007). 10.1016/j.yexcr.2007.04.022 PMC2040058 17524393

[41] Zschenker, O., Streichert, T., Hehlgans, S. & Cordes, N. Genome-wide gene expression analysis in cancer cells reveals 3D growth to affect ECM and processes associated with cell adhesion but not DNA repair. *PLoS ONE* **7**, e34279 (2012). 10.1371/journal.pone.0034279 PMC3324525 22509286

[42] Dutertre, S. et al. Phosphorylation of CDC25B by Aurora-A at the centrosome contributes to the G2-M transition. *J. Cell Sci.* **117**, 2523–2531 (2004). 10.1242/jcs.01108 15128871

[43] Macůrek, L. et al. Polo-like kinase-1 is activated by aurora A to promote checkpoint recovery. *Nature* **455**, 119–123 (2008). 10.1038/nature07185 18615013

[44] Hurlin, P. J. et al. Mad3 and Mad4: novel Max-interacting transcriptional repressors that suppress c-myc dependent transformation and are expressed during neural and epidermal differentiation. *EMBO J.* **14**, 5646–5659 (1995). 10.1002/j.1460-2075.1995.tb00252.x PMC394680 8521822

[45] Coutier, J. et al. MXD4/MAD4 regulates human keratinocyte precursor fate. *J. Investig. Dermatol* **143**, 105–114.e12 (2023). 10.1016/j.jid.2022.07.020 36007550

[46] Ikegami, K. et al. Genome-wide and locus-specific DNA hypomethylation in G9a deficient mouse embryonic stem cells. *Genes Cells* **12**, 1–11 (2007). 10.1111/j.1365-2443.2006.01029.x 17212651

[47] Flora, P., Dalal, G., Cohen, I. & Ezhkova, E. Polycomb repressive complex(es) and their role in adult stem cells. *Genes* **12**, 1485 (2021). 10.3390/genes12101485 PMC8535826 34680880

[48] Chen, L. et al. PHC1 maintains pluripotency by organizing genome-wide chromatin interactions of the Nanog locus. *Nat. Commun.* **12**, 2829 (2021). 10.1038/s41467-021-22871-0 PMC8121881 33990559

[49] Feng, Y. et al. BRPF3-HBO1 regulates replication origin activation and histone H3K14 acetylation. *EMBO J.* **35**, 176–192 (2016). 10.15252/embj.201591293 PMC4718456 26620551

[50] Menon, V. R. et al. DYRK1A regulates the recruitment of 53BP1 to the sites of DNA damage in part through interaction with RNF169. *Cell Cycle* **18**, 531–551 (2019). 10.1080/15384101.2019.1577525 PMC6464593 30773093

[51] Di Vona, C. et al. Chromatin-wide profiling of DYRK1A reveals a role as a gene-specific RNA polymerase II CTD kinase. *Mol. Cell* **57**, 506–520 (2015). 10.1016/j.molcel.2014.12.026 25620562

[52] Lu, H. et al. Phase-separation mechanism for C-terminal hyperphosphorylation of RNA polymerase II. *Nature* **558**, 318–323 (2018). 10.1038/s41586-018-0174-3 PMC6475116 29849146

[53] Zou, Y., Lim, S., Lee, K., Deng, X. & Friedman, E. Serine/threonine kinase Mirk/Dyrk1B is an inhibitor of epithelial cell migration and is negatively regulated by the Met adaptor Ran-binding protein M. *J. Biol. Chem.* **278**, 49573–49581 (2003). 10.1074/jbc.M307556200 14500717

[54] Jang, S. M., Azebi, S., Soubigou, G. & Muchardt, C. DYRK1A phoshorylates histone H3 to differentially regulate the binding of HP1 isoforms and antagonize HP1-mediated transcriptional repression. *EMBO Rep.* **15**, 686–694 (2014). 10.15252/embr.201338356 PMC4197879 24820035

[55] Eferl, R. & Wagner, E. F. AP-1: a double-edged sword in tumorigenesis. *Nat. Rev. Cancer* **3**, 859–868 (2003). 10.1038/nrc1209 14668816

[56] Kang, H., Park, Y.-K., Lee, J.-Y. & Bae, M. Roles of histone deacetylase 4 in the inflammatory and metabolic processes. *Diab. Metab. J.* **48**, 340–353 (2024). 10.4093/dmj.2023.0174 PMC11140402 38514922

[57] Yang, X., Zhang, F. & Kudlow, J. E. Recruitment of O-GlcNAc transferase to promoters by corepressor mSin3A: coupling protein O-GlcNAcylation to transcriptional repression. *Cell* **110**, 69–80 (2002). 10.1016/s0092-8674(02)00810-3 12150998

[58] Bannister, A. J. & Kouzarides, T. Regulation of chromatin by histone modifications. *Cell Res* **21**, 381–395 (2011). 10.1038/cr.2011.22 PMC3193420 21321607

[59] Verdin, E. & Ott, M. 50 years of protein acetylation: from gene regulation to epigenetics, metabolism and beyond. *Nat. Rev. Mol. Cell Biol.* **16**, 258–264 (2015). 10.1038/nrm3931 25549891

[60] Glozak, M. A. & Seto, E. Histone deacetylases and cancer. *Oncogene* **26**, 5420–5432 (2007). 10.1038/sj.onc.1210610 17694083

[61] Saunders, A. et al. The SIN3A/HDAC corepressor complex functionally cooperates with NANOG to promote pluripotency. *Cell Rep.* **18**, 1713–1726 (2017). 10.1016/j.celrep.2017.01.055 PMC5328122 28199843

[62] Lechner, M. et al. Identification and functional validation of HPV-mediated hypermethylation in head and neck squamous cell carcinoma. *Genome Med.* **5**, 15 (2013). 10.1186/gm419 PMC3706778 23419152

[63] Ang, K. K. et al. Human papillomavirus and survival of patients with oropharyngeal cancer. *N. Engl. J. Med.* **363**, 24–35 (2010). 10.1056/NEJMoa0912217 PMC2943767 20530316

[64] Vlachogiannis, G. et al. Patient-derived organoids model treatment response of metastatic gastrointestinal cancers. *Science* **359**, 920–926 (2018). 10.1126/science.aao2774 PMC6112415 29472484

[65] Drost, J. & Clevers, H. Organoids in cancer research. *Nat. Rev. Cancer* **18**, 407–418 (2018). 10.1038/s41568-018-0007-6 29692415

[66] Weaver, V. M. et al. Reversion of the malignant phenotype of human breast cells in three-dimensional culture and in vivo by integrin blocking antibodies. *J. Cell Biol.* **137**, 231–245 (1997). 10.1083/jcb.137.1.231 PMC2139858 9105051

[67] Nelson, C. M. & Bissell, M. J. Of extracellular matrix, scaffolds, and signaling: tissue architecture regulates development, homeostasis, and cancer. *Annu. Rev. Cell Dev. Biol.* **22**, 287–309 (2006). 10.1146/annurev.cellbio.22.010305.104315 PMC2933192 16824016

[68] Eke, I. et al. EGFR/JIP-4/JNK2 signaling attenuates cetuximab-mediated radiosensitization of squamous cell carcinoma cells. *Cancer Res.* **73**, 297–306 (2013). 10.1158/0008-5472.CAN-12-2021 23074283

[69] Driehuis, E. et al. Oral mucosal organoids as a potential platform for personalized cancer therapy. *Cancer Discov.* **9**, 852–871 (2019). 10.1158/2159-8290.CD-18-1522 31053628

[70] Wensink, G. E. et al. Patient-derived organoids as a predictive biomarker for treatment response in cancer patients. *npj Precis Oncol.* **5**, 30 (2021). 10.1038/s41698-021-00168-1 PMC8042051 33846504

