# Supplementary material for: α7 integrin co-regulates chromatin accessibility and gene expression in HPV-negative head and neck cancer
Source: Signal Transduct Target Ther. 2026 Oct 7;11:429. doi: 10.1038/s41392-026-02995-9 (PMC13642342; doi:10.1038/s41392-026-02995-9)
Supplement: Supplementary file 1 [file 41392_2026_2995_MOESM2_ESM.pdf]

Supplementary western blot images for

α7 integrin co-regulates chromatin accessibility and gene expression in HPV-negative head and neck cancer

Olegs Borodins [1], Anne Vehlow [1], Katrin Sameith [2], Andreas Dahl [2], Jennifer M. Spangle [3], Daniela E. Aust [4], Cornelia Brunner [5], Outi Monni [6,7,8], Inga Lange [1,9], Jovan Mircetic [10], Elisa Thomas [1,10,11], Nils Cordes [1,9,10,11,*]

Correspondence to: Nils.Cordes@OncoRay.de

This PDF file includes:

Raw western blot images

Please note that the immunoblot images were acquired using a series of different exposure times. Therefore, the white-light image is provided only to indicate the position of the bands and should not be used to assess immunoblot signal density. Images here are uncropped and unmodified.

Integrin α7

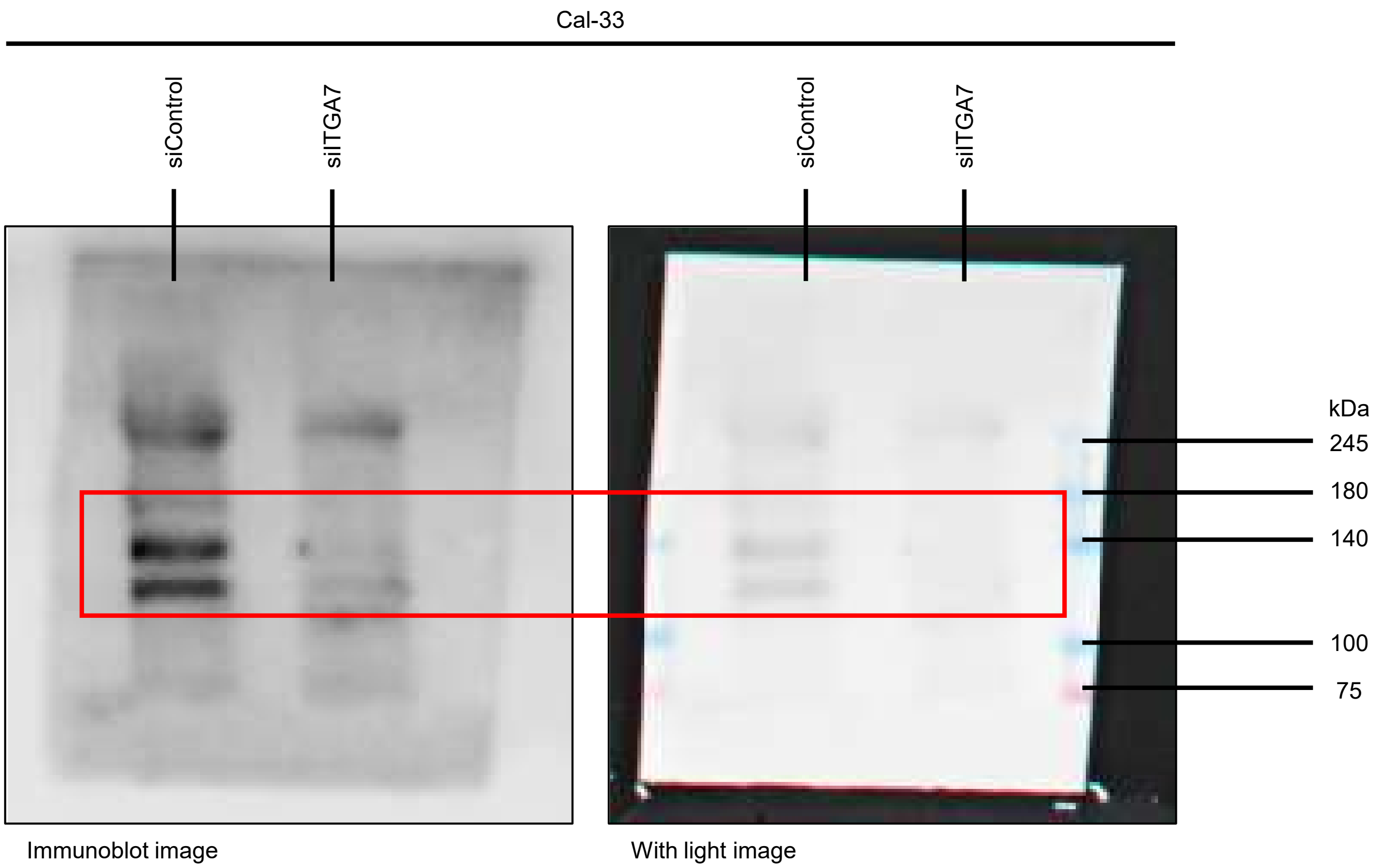

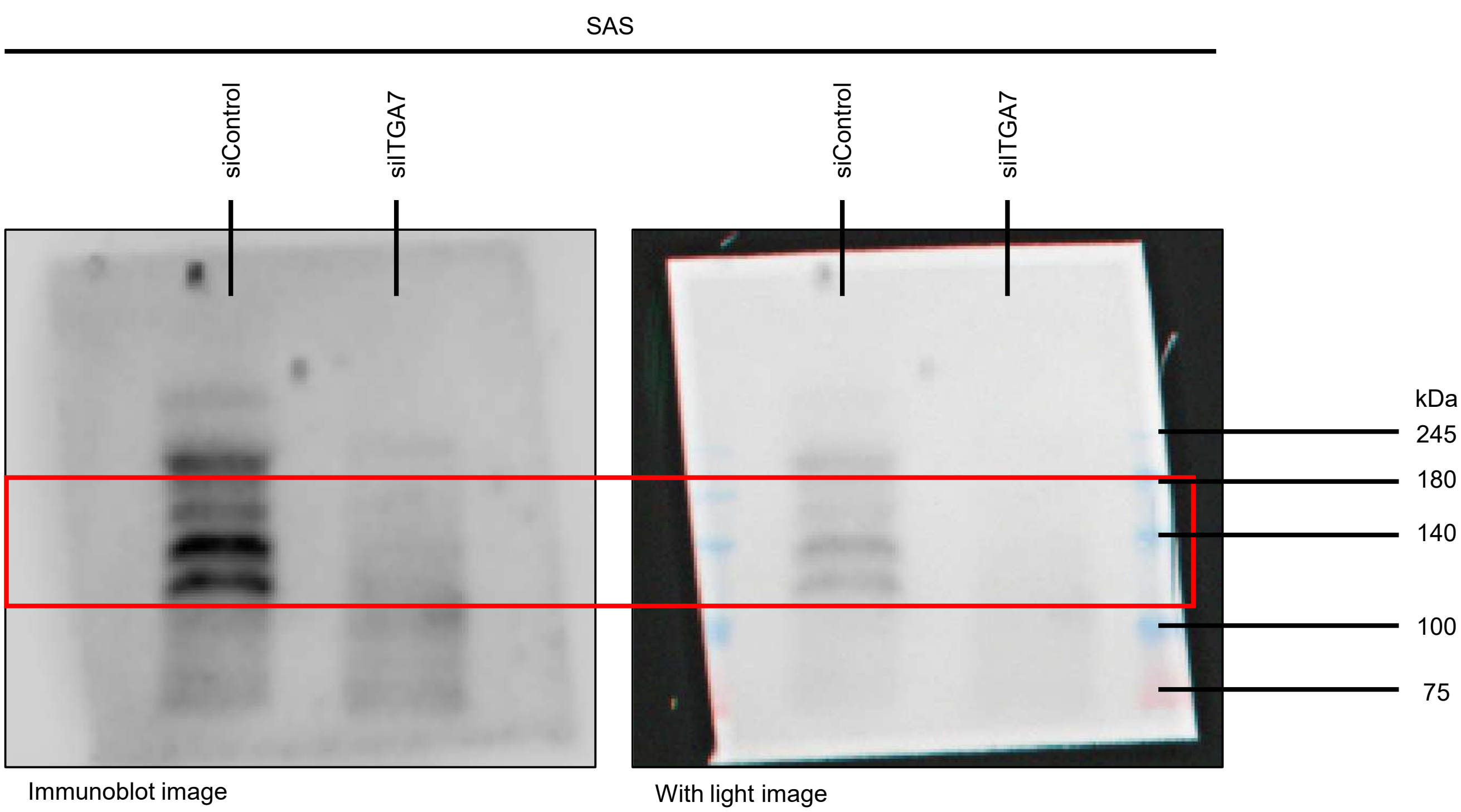

Area cropped on the article figure

p38 MAPK (pan)

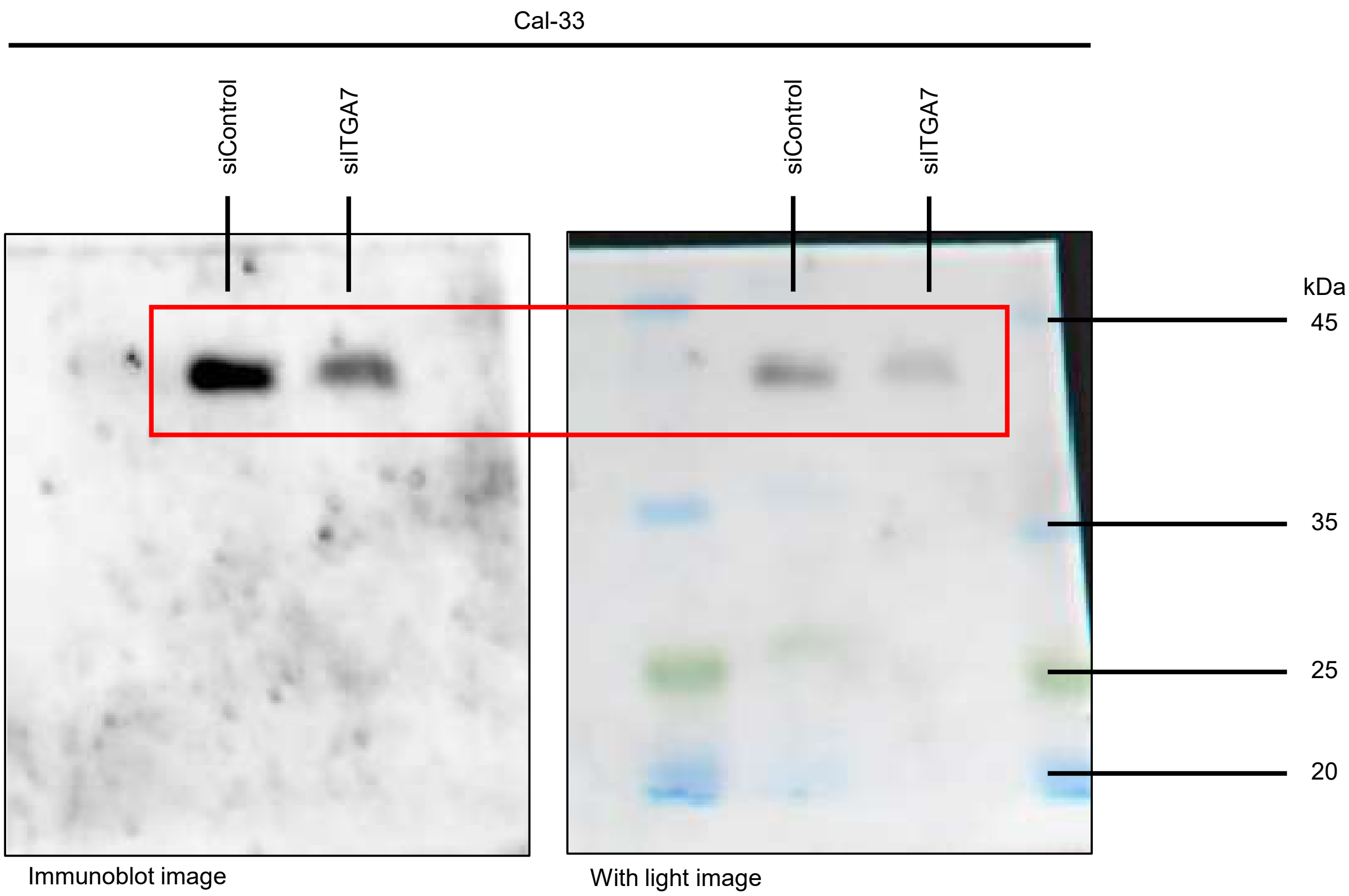

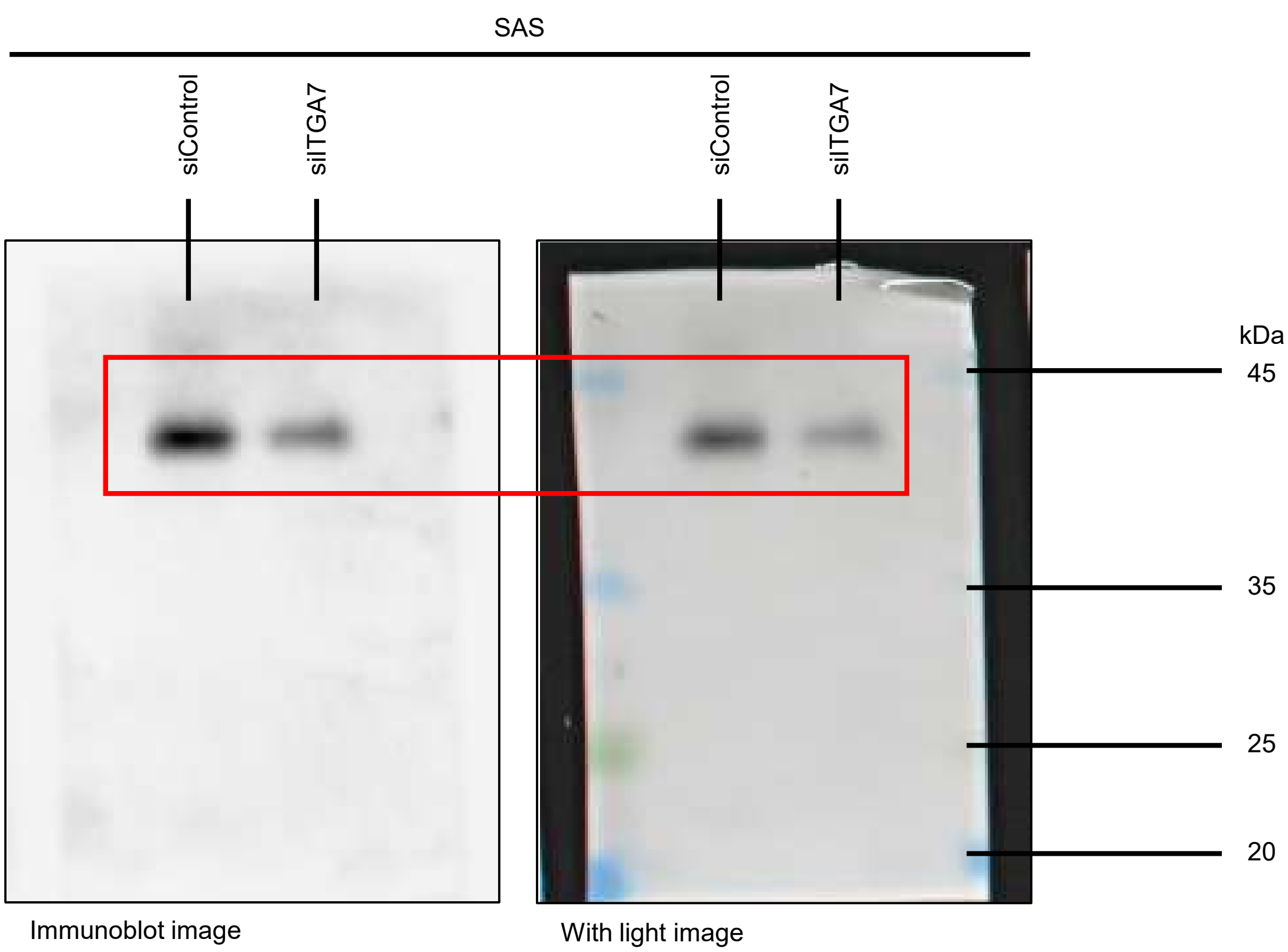

Area cropped on the article figure

p-p38 (Thr180/Tyr182)

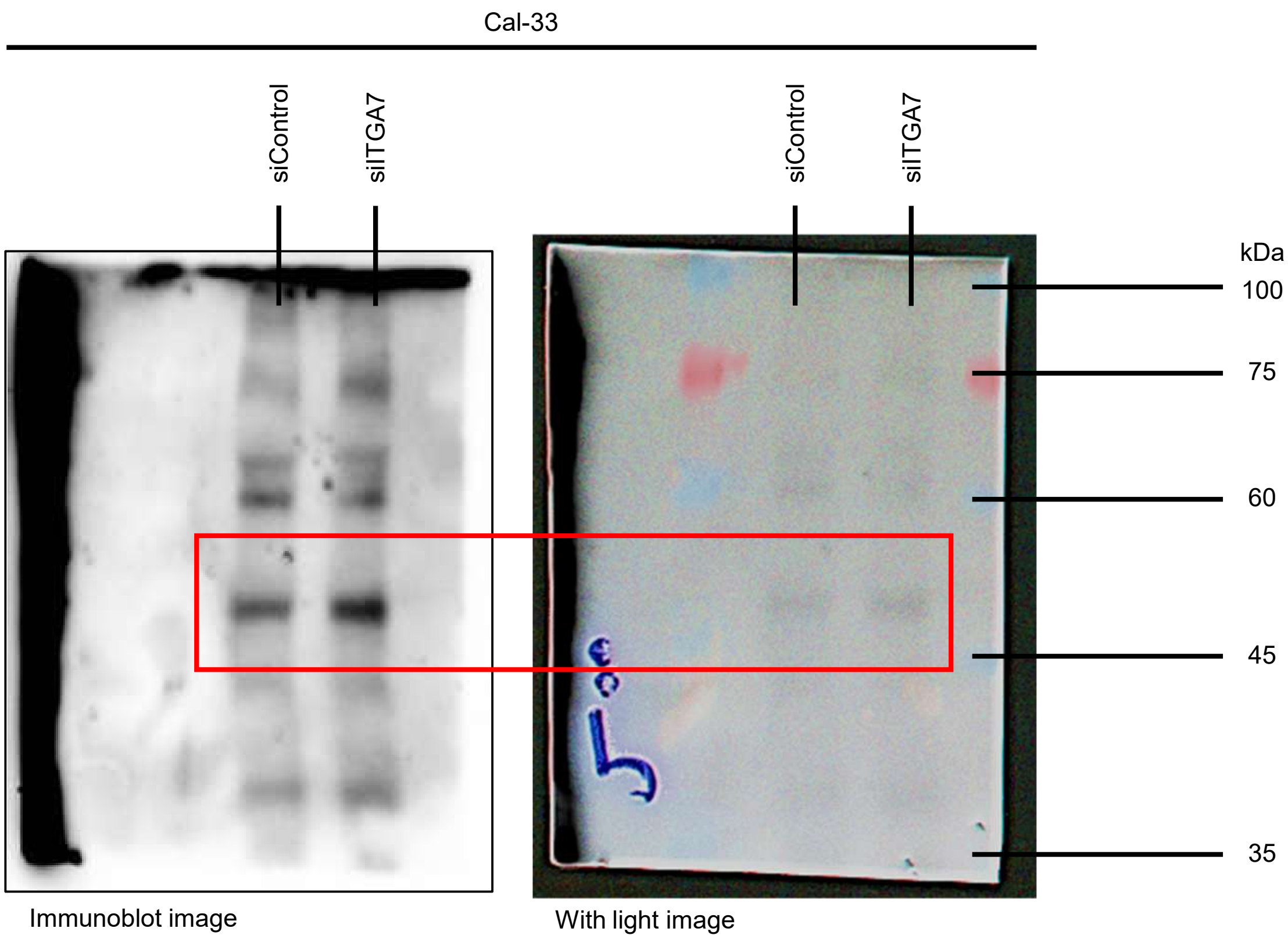

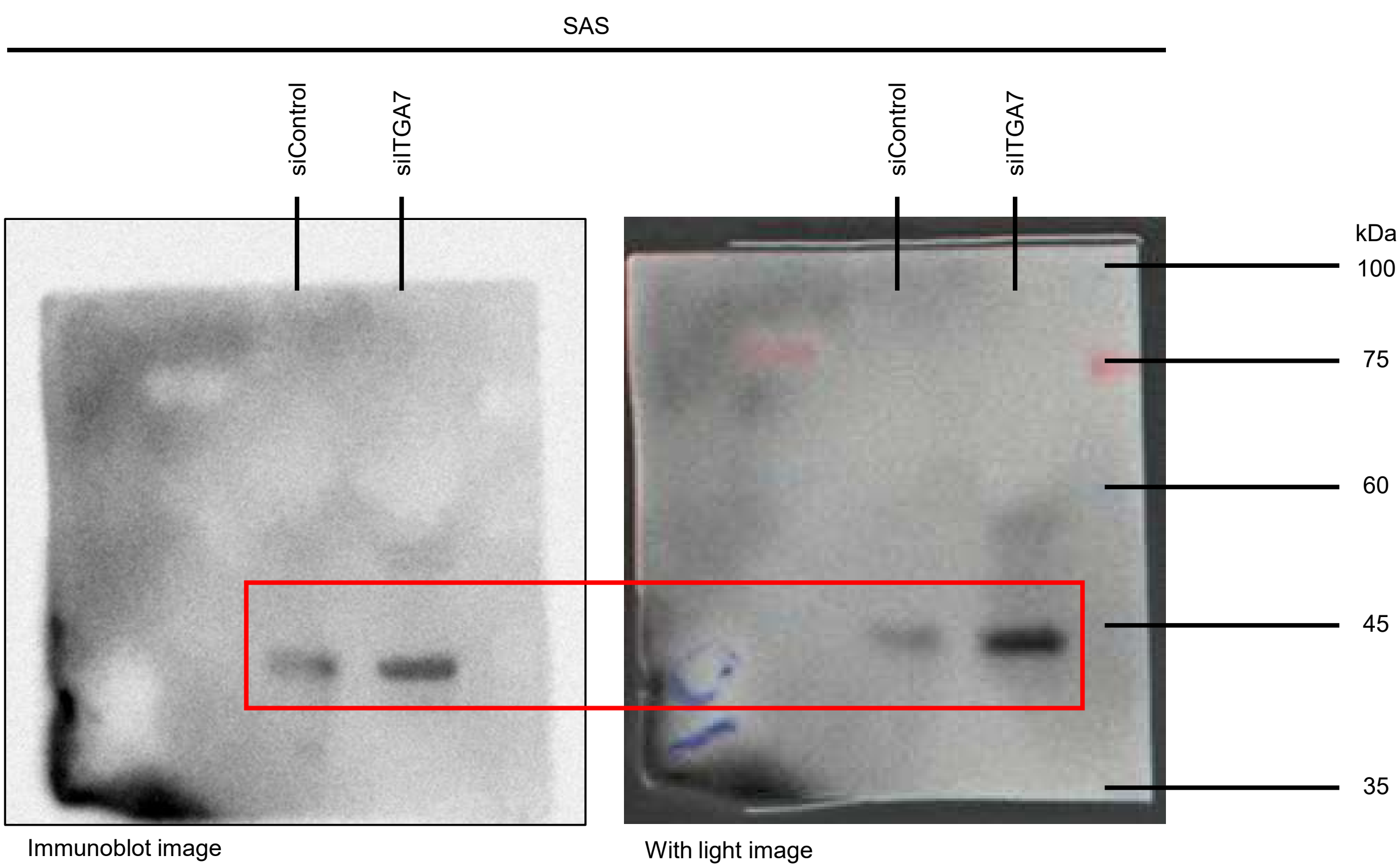

Area cropped on the article figure

MAPKAPK-2

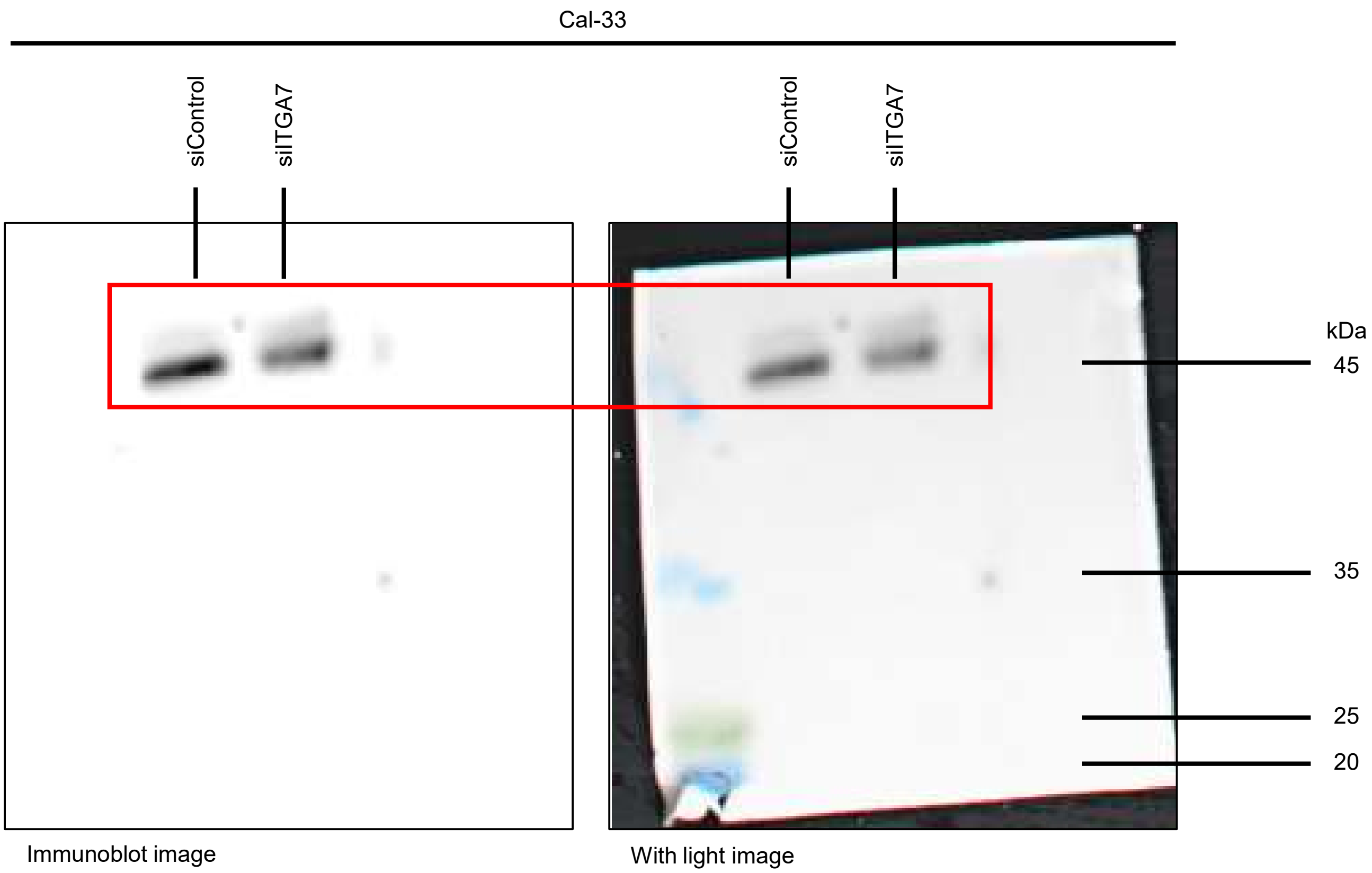

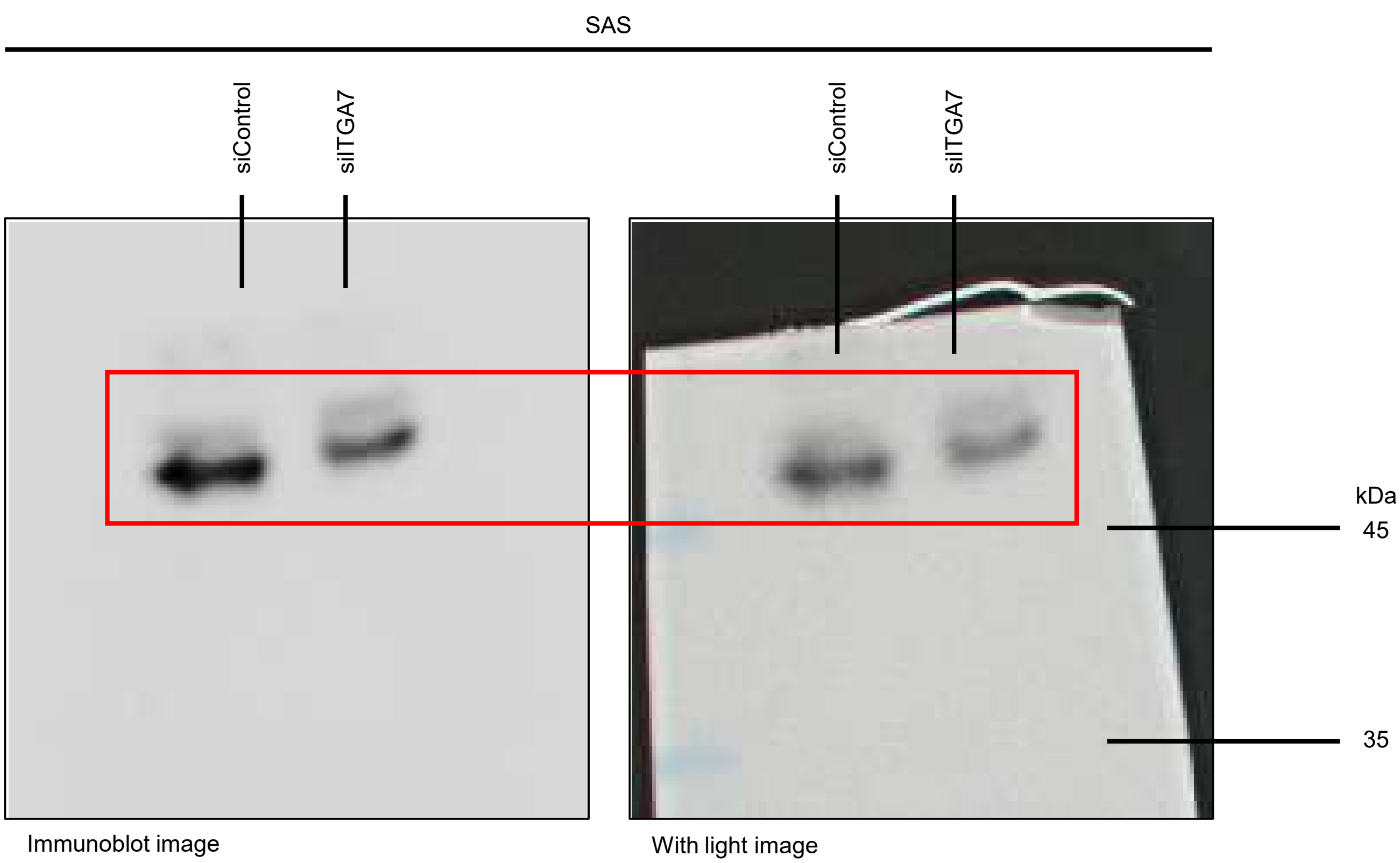

Area cropped on the article figure

ERK1 and ERK2

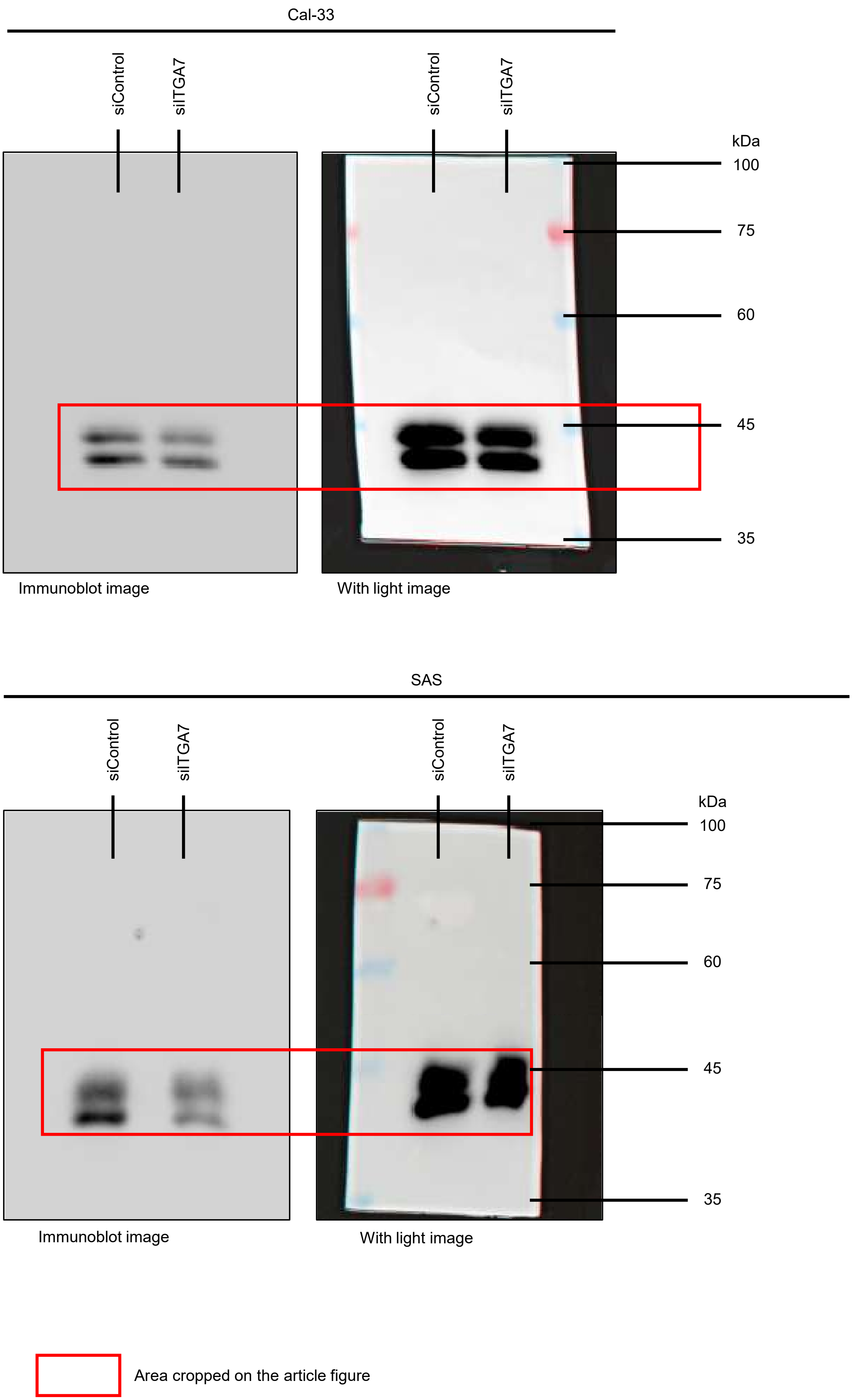

p-ERK1 (Thr202/Tyr204) and p-ERK2 (Thr202/Tyr204)

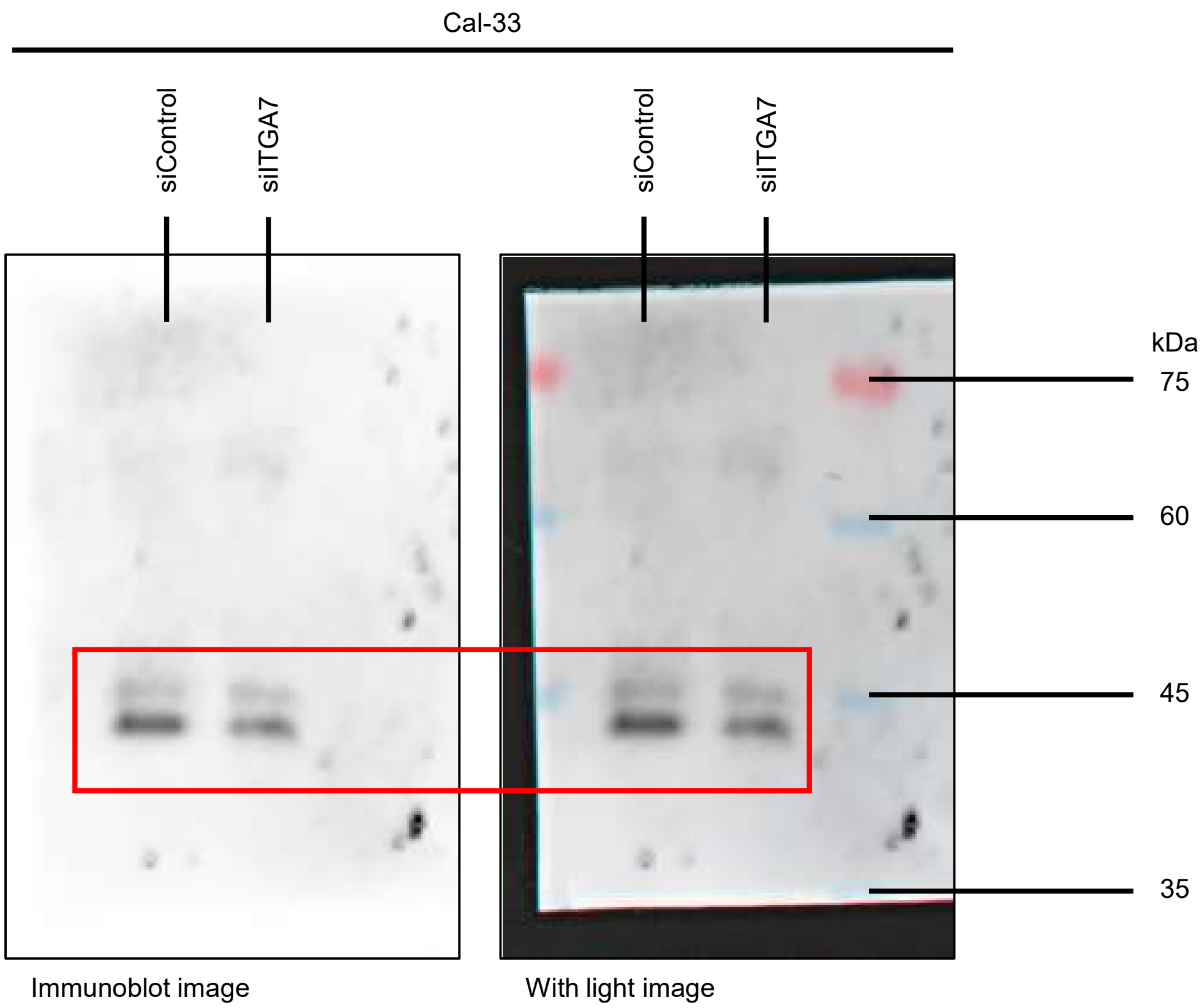

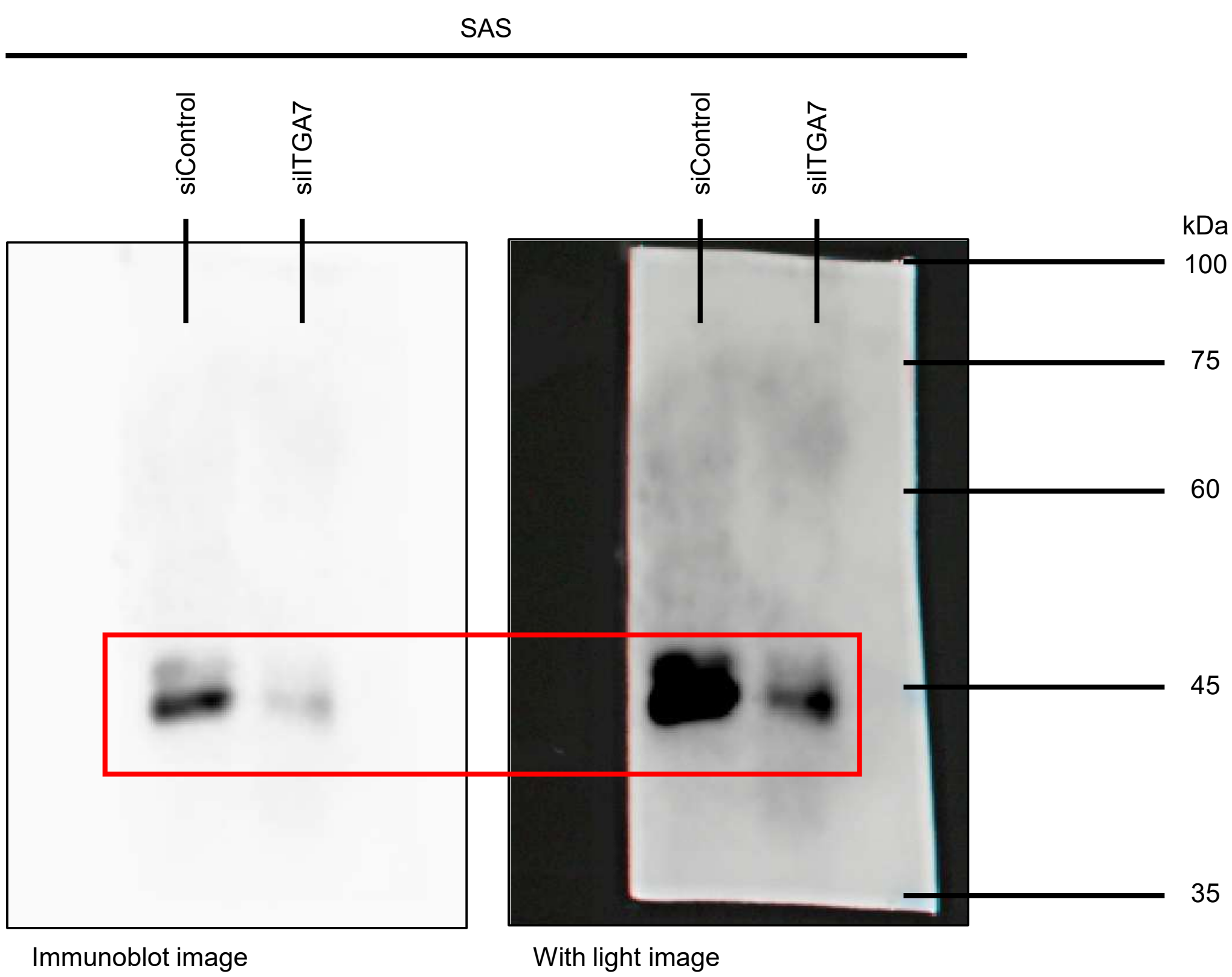

Area cropped on the article figure

JNK (pan)

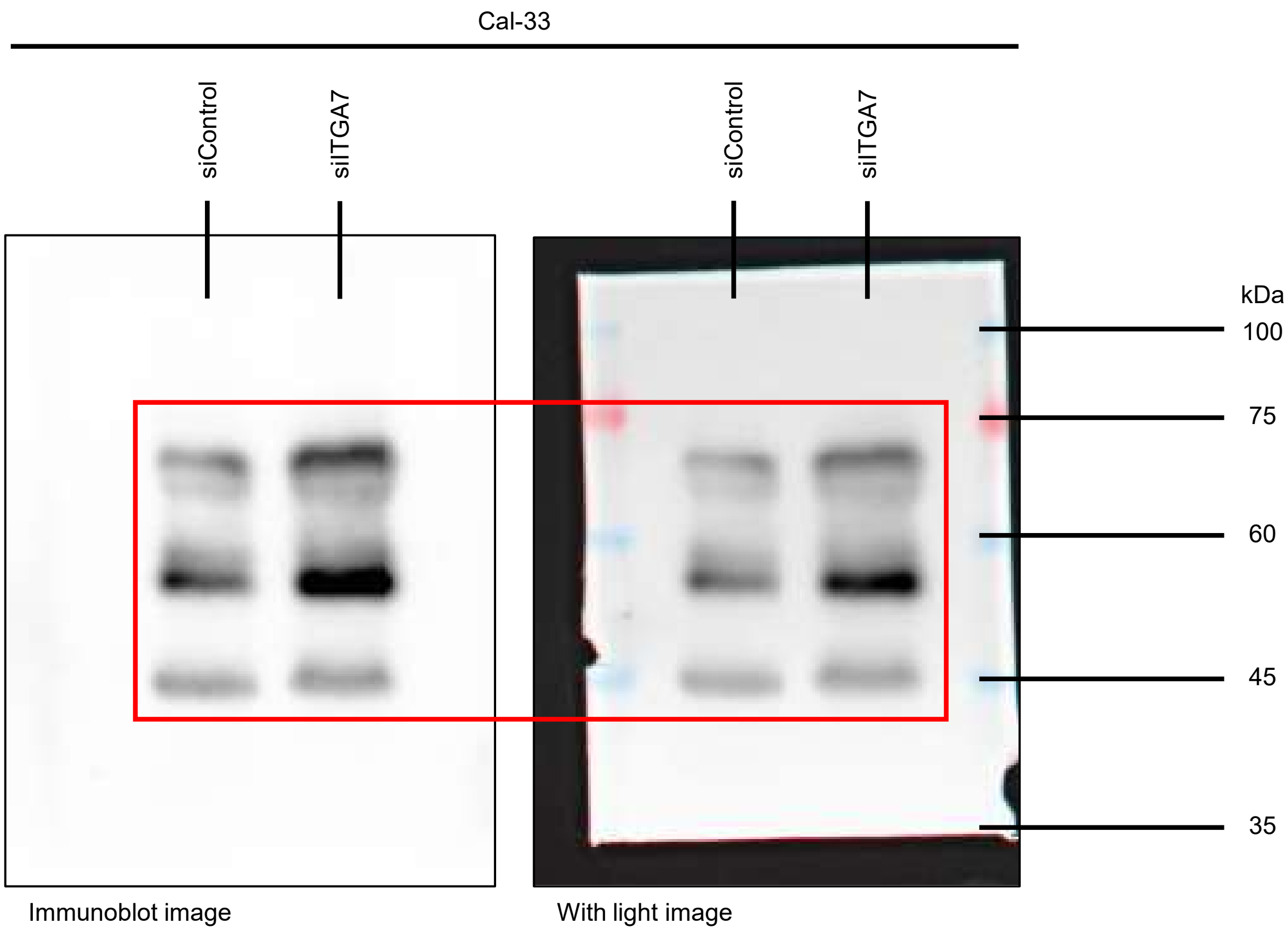

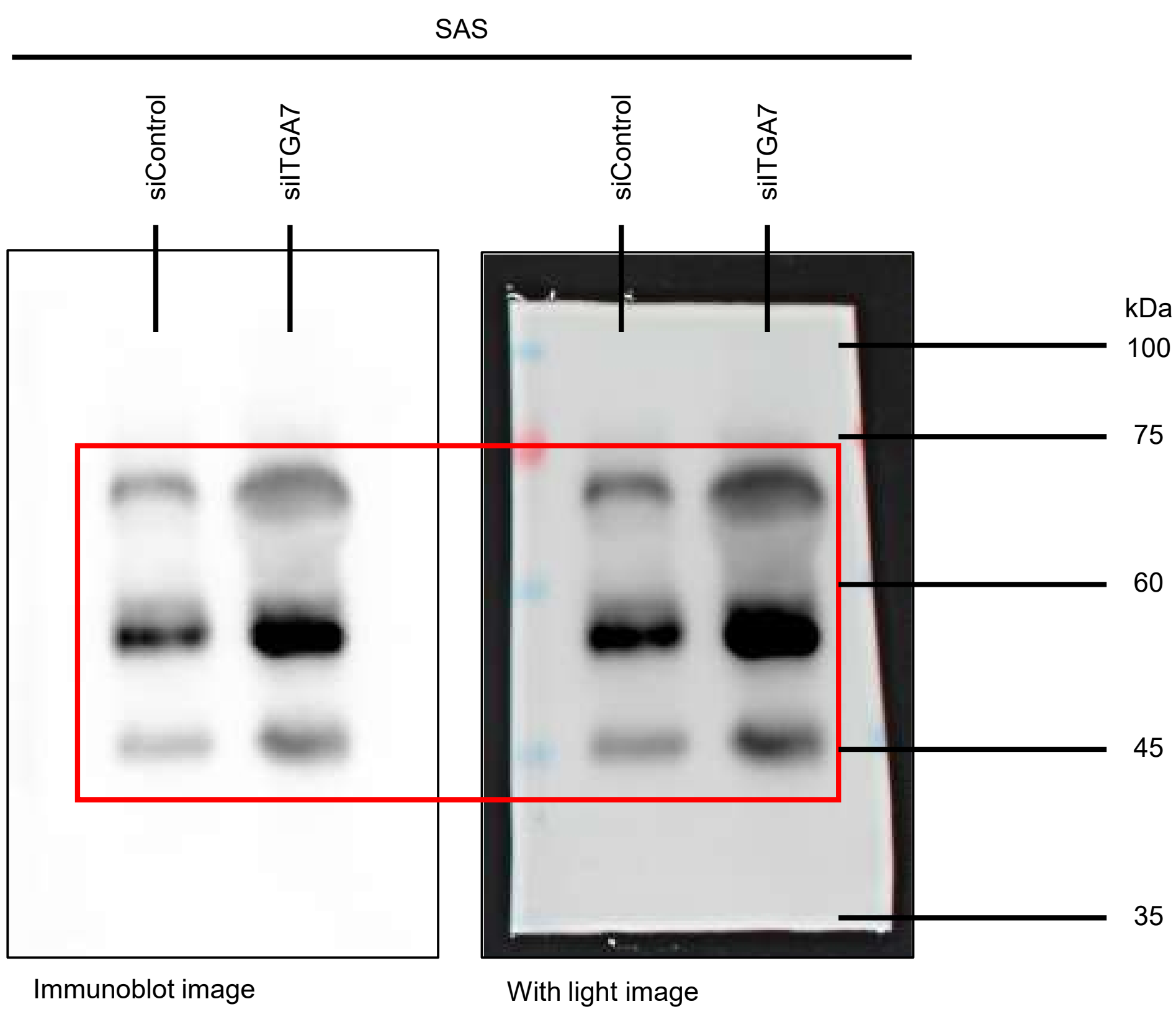

Area cropped on the article figure

JNK1

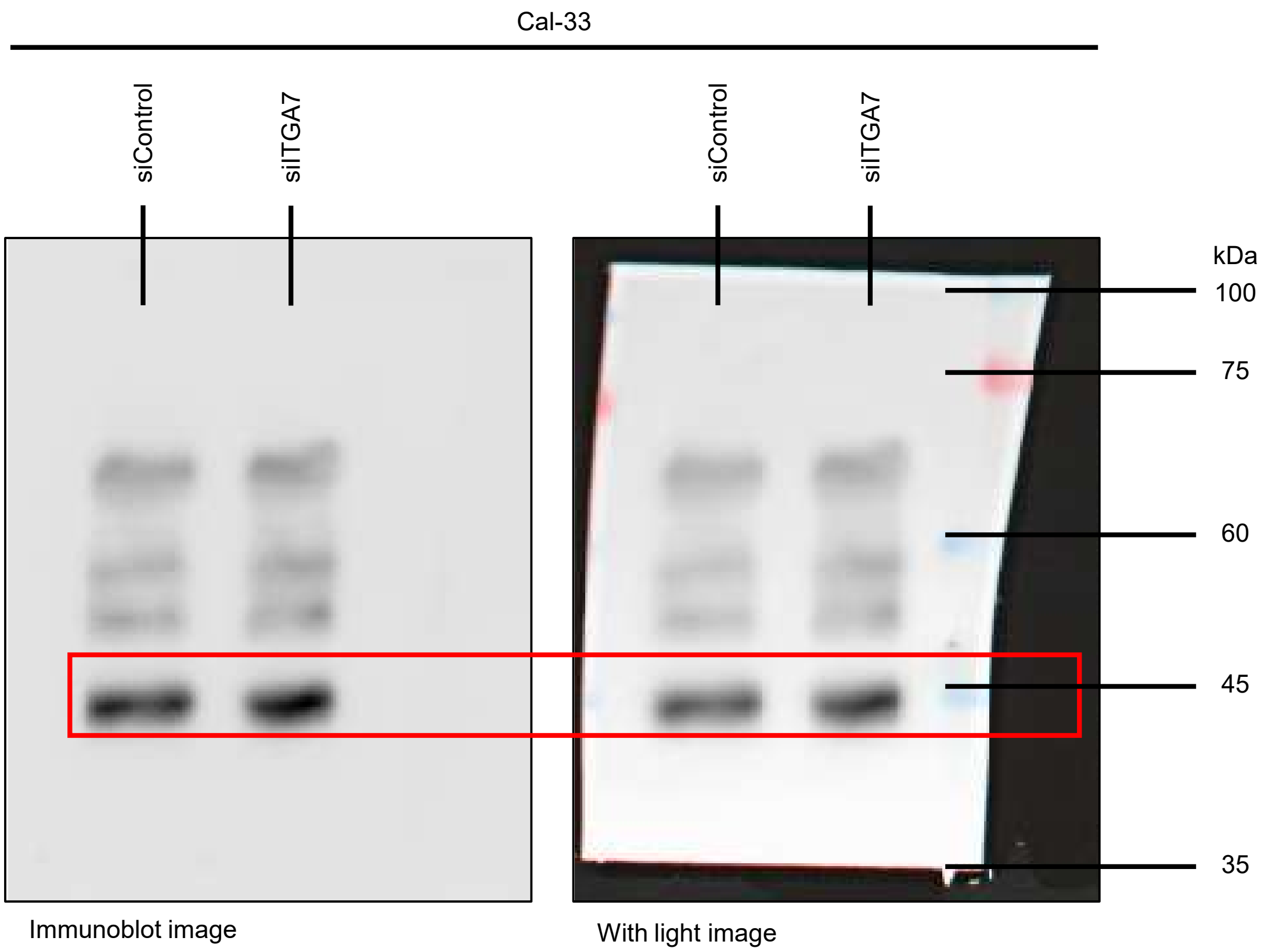

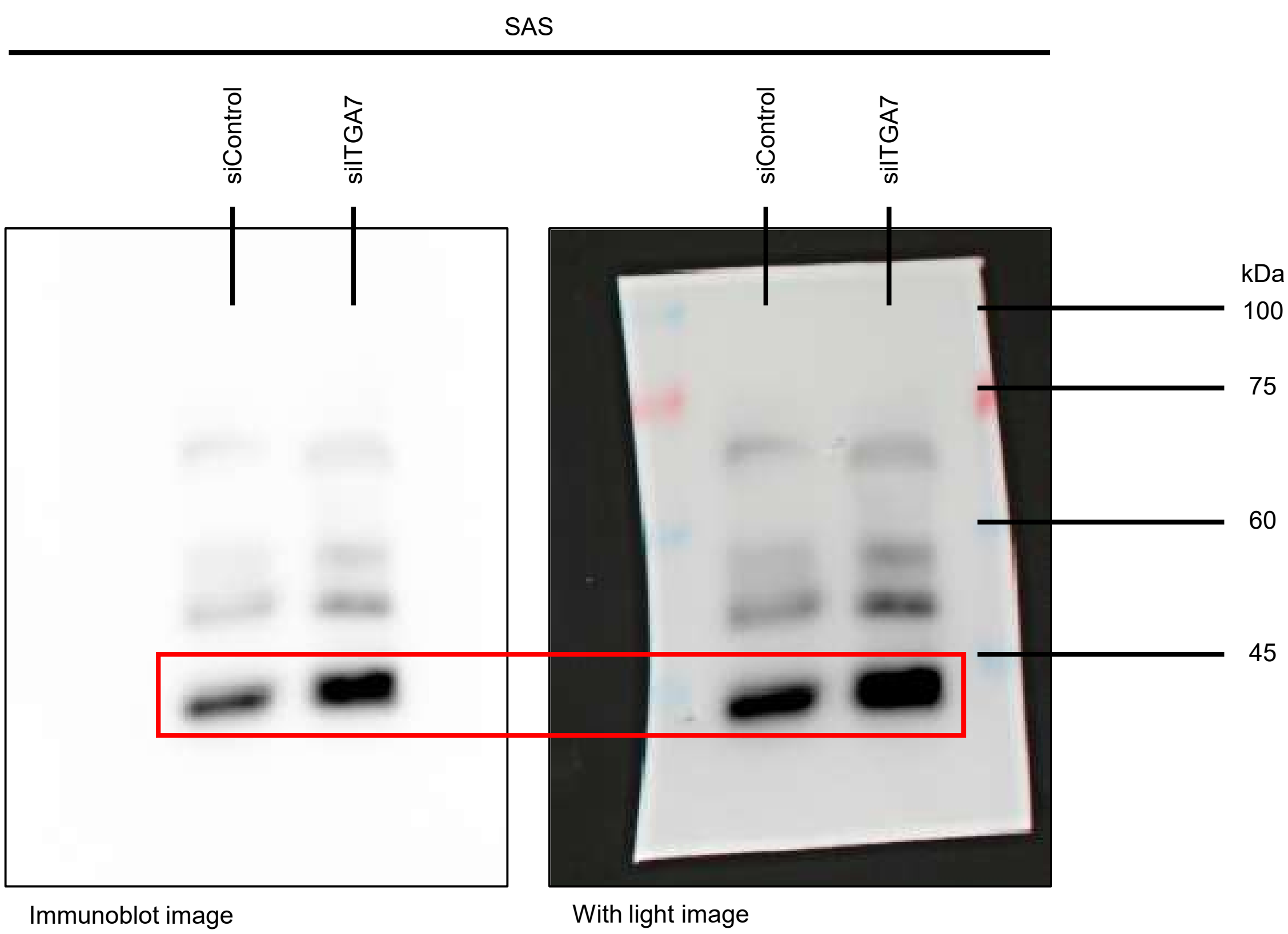

Area cropped on the article figure

JNK2

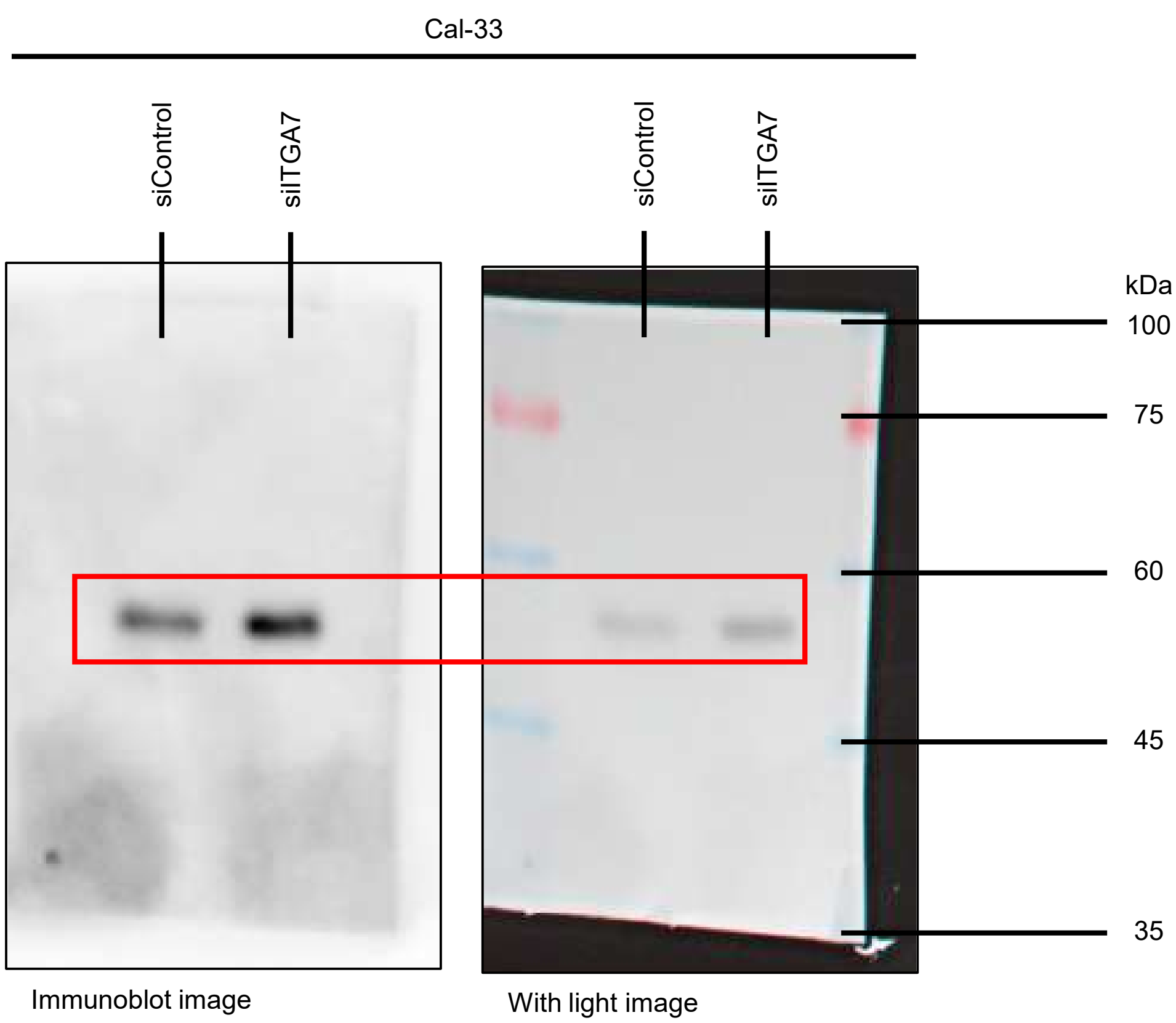

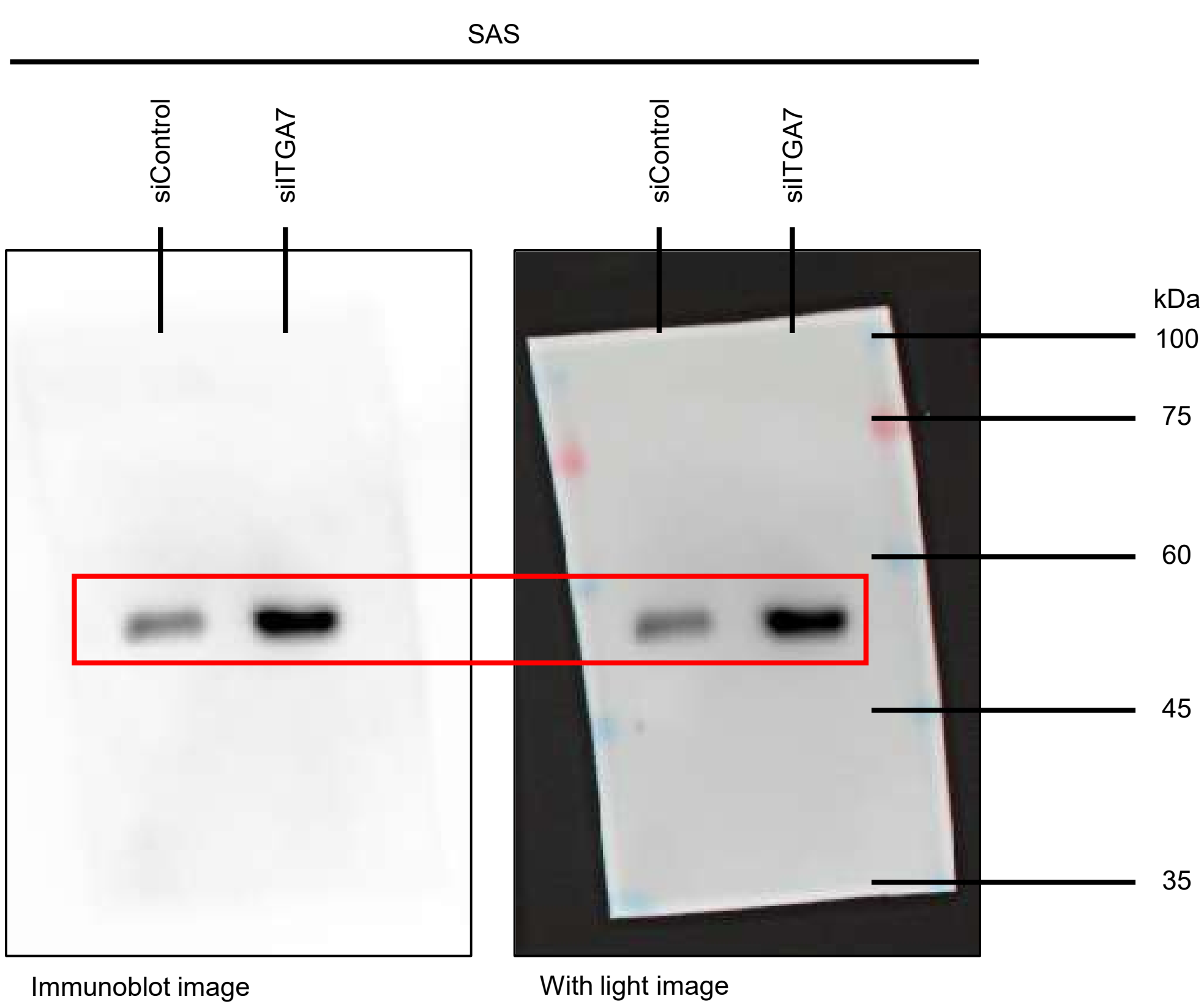

Area cropped on the article figure

AKT (pan)

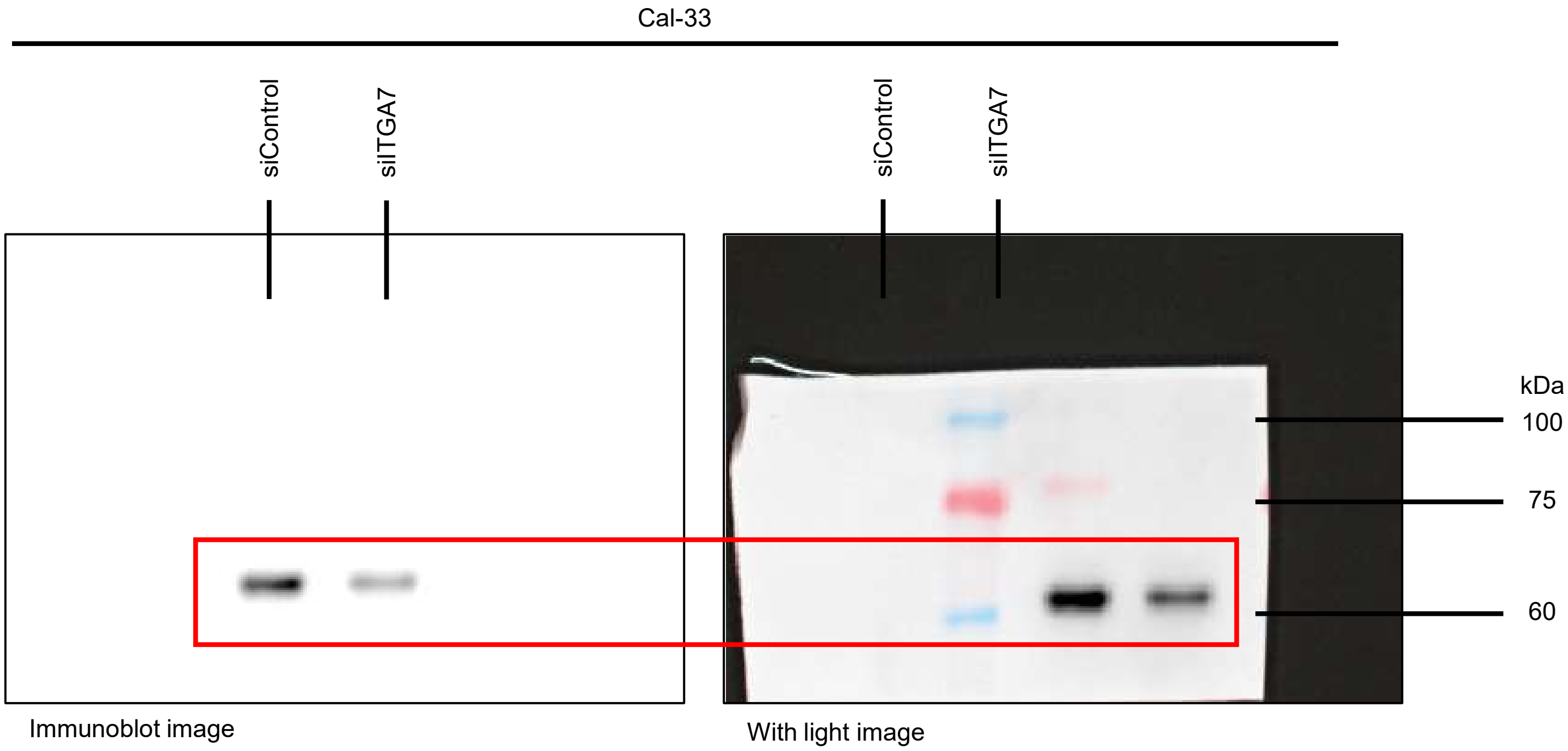

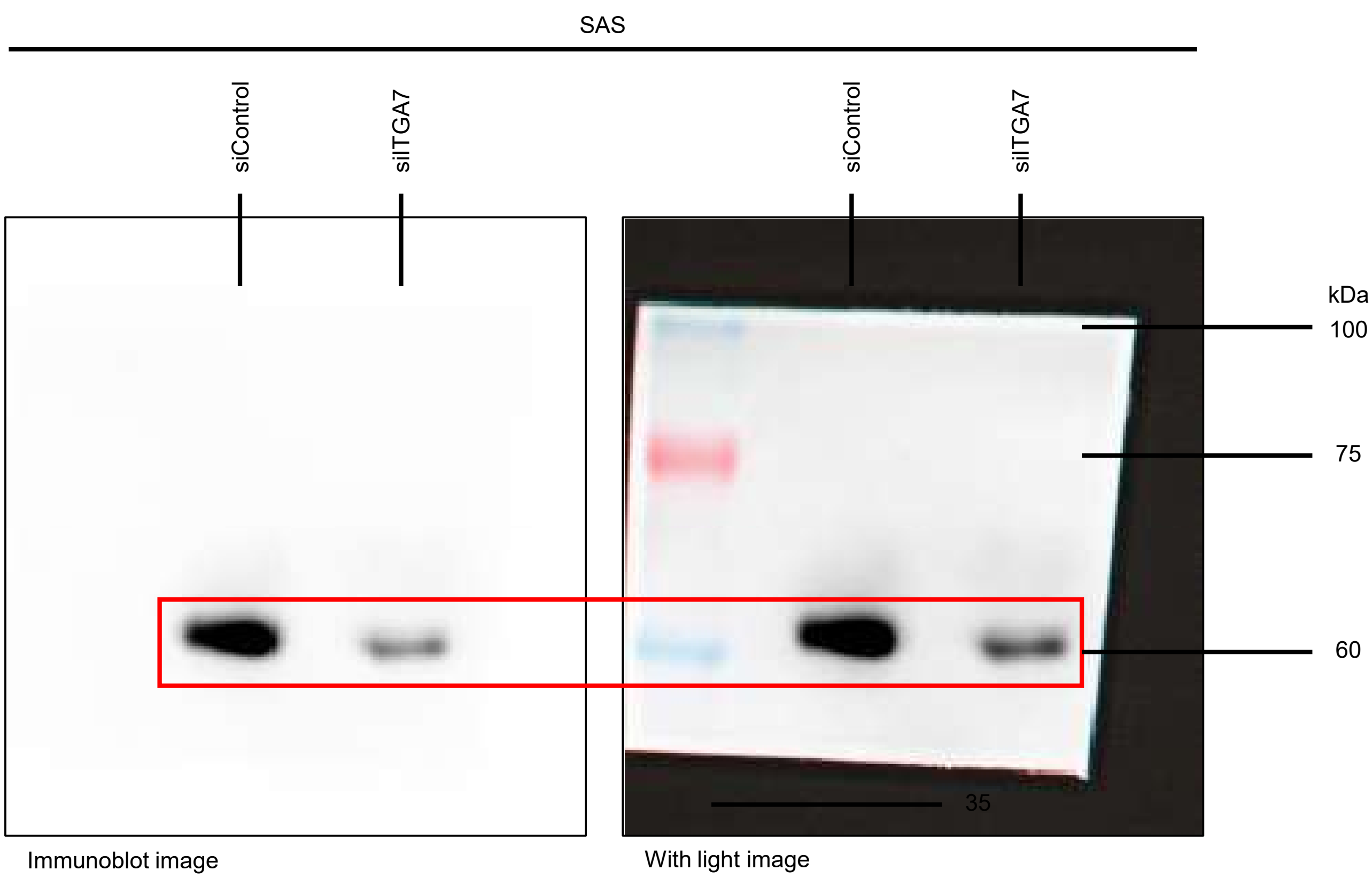

Area cropped on the article figure

p-AKT (Ser473)

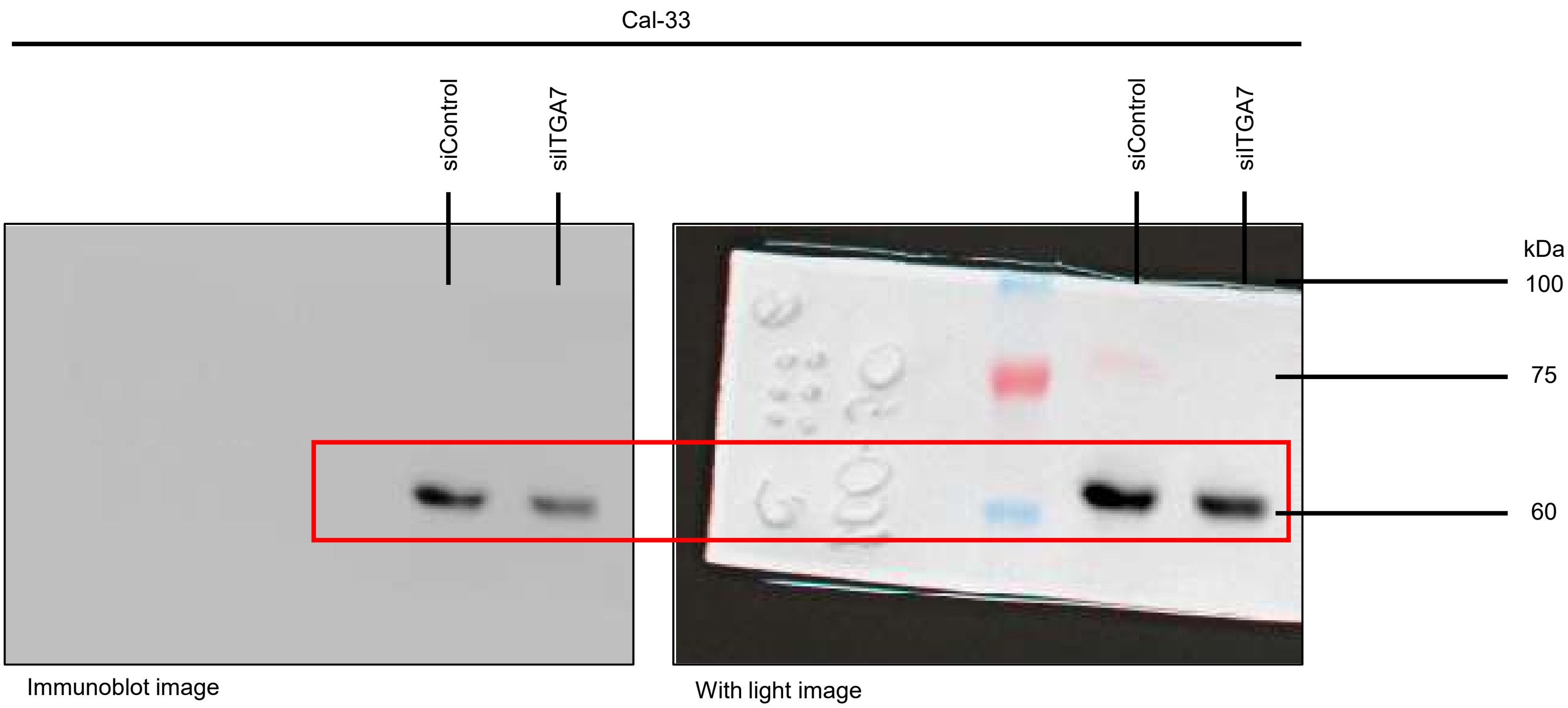
Cal-33
siControl
siITGA7
siControl
siITGA7
kDa
100
75
60
Immunoblot image
With light image

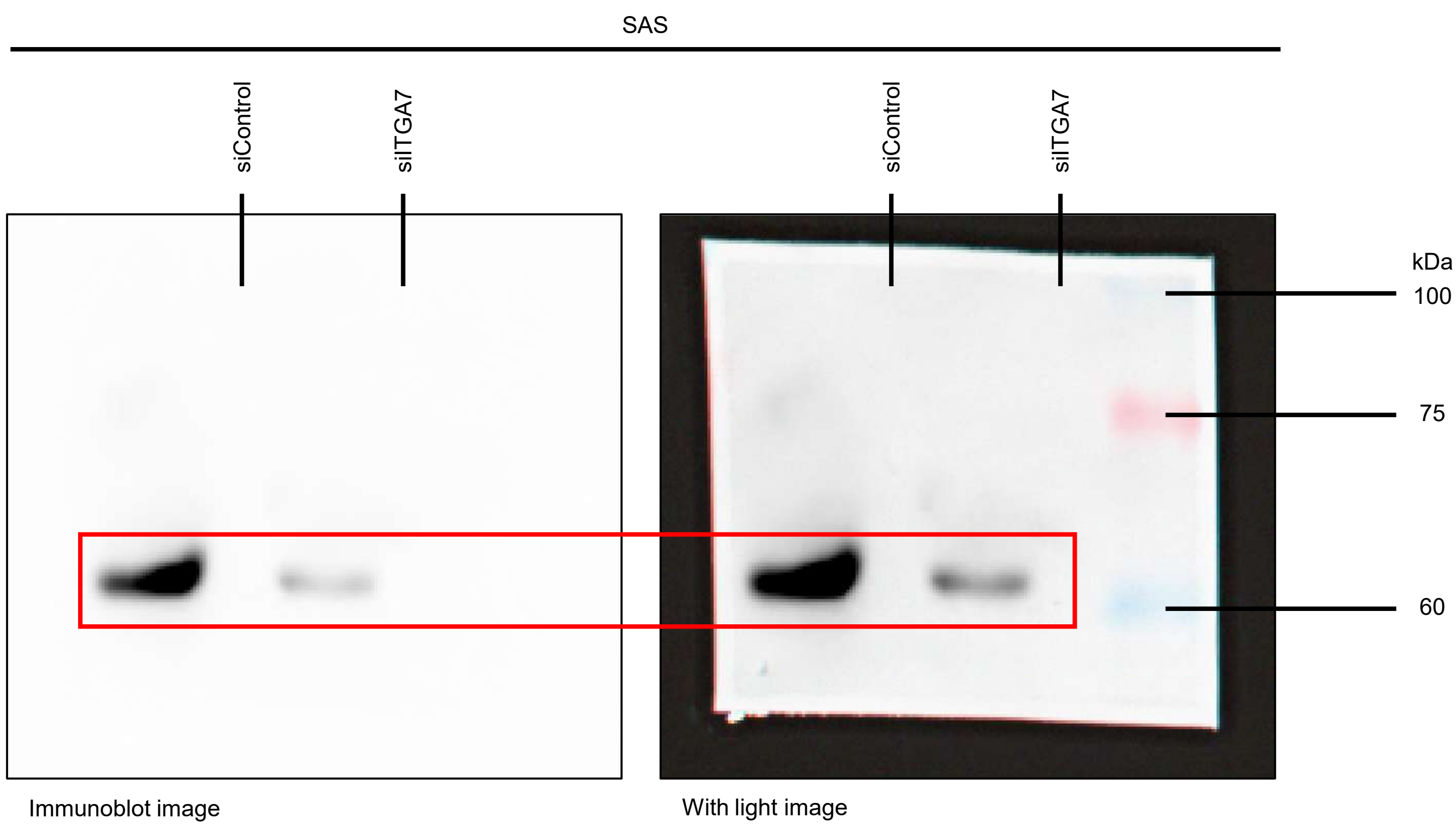
SAS
siControl
siITGA7
siControl
siITGA7
kDa
100
75
60
Immunoblot image
With light image

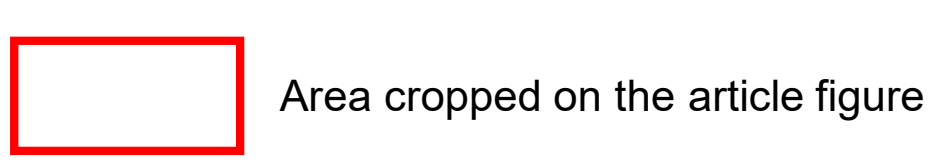
Area cropped on the article figure

GSK-3β

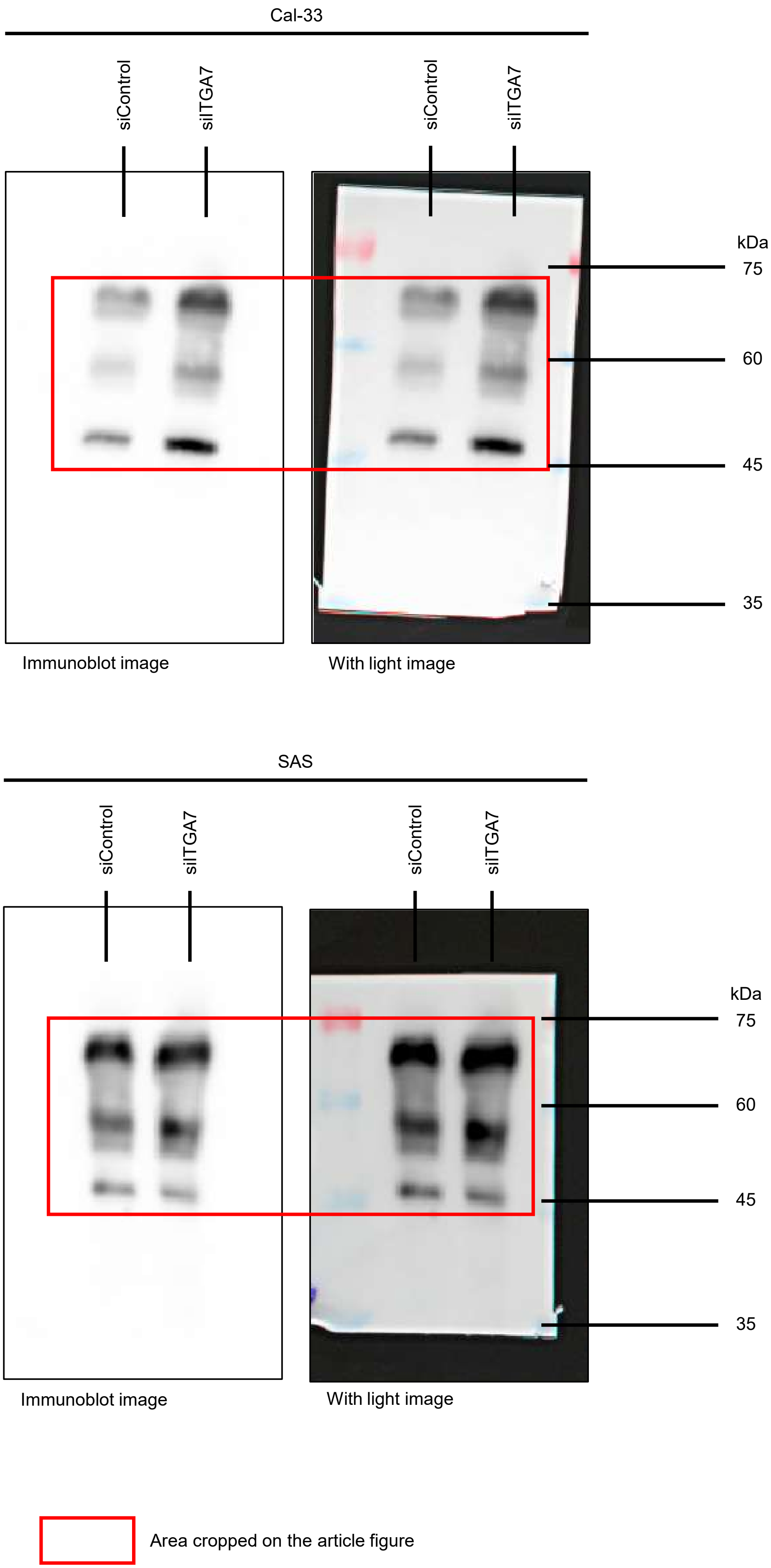

Cal-33
siControl
siITGA7
siControl
siITGA7
kDa
75
60
45
35
Immunoblot image
With light image
SAS
siControl
siITGA7
siControl
siITGA7
kDa
75
60
45
35
Immunoblot image
With light image
Area cropped on the article figure

p-GSK-3β (Ser9)

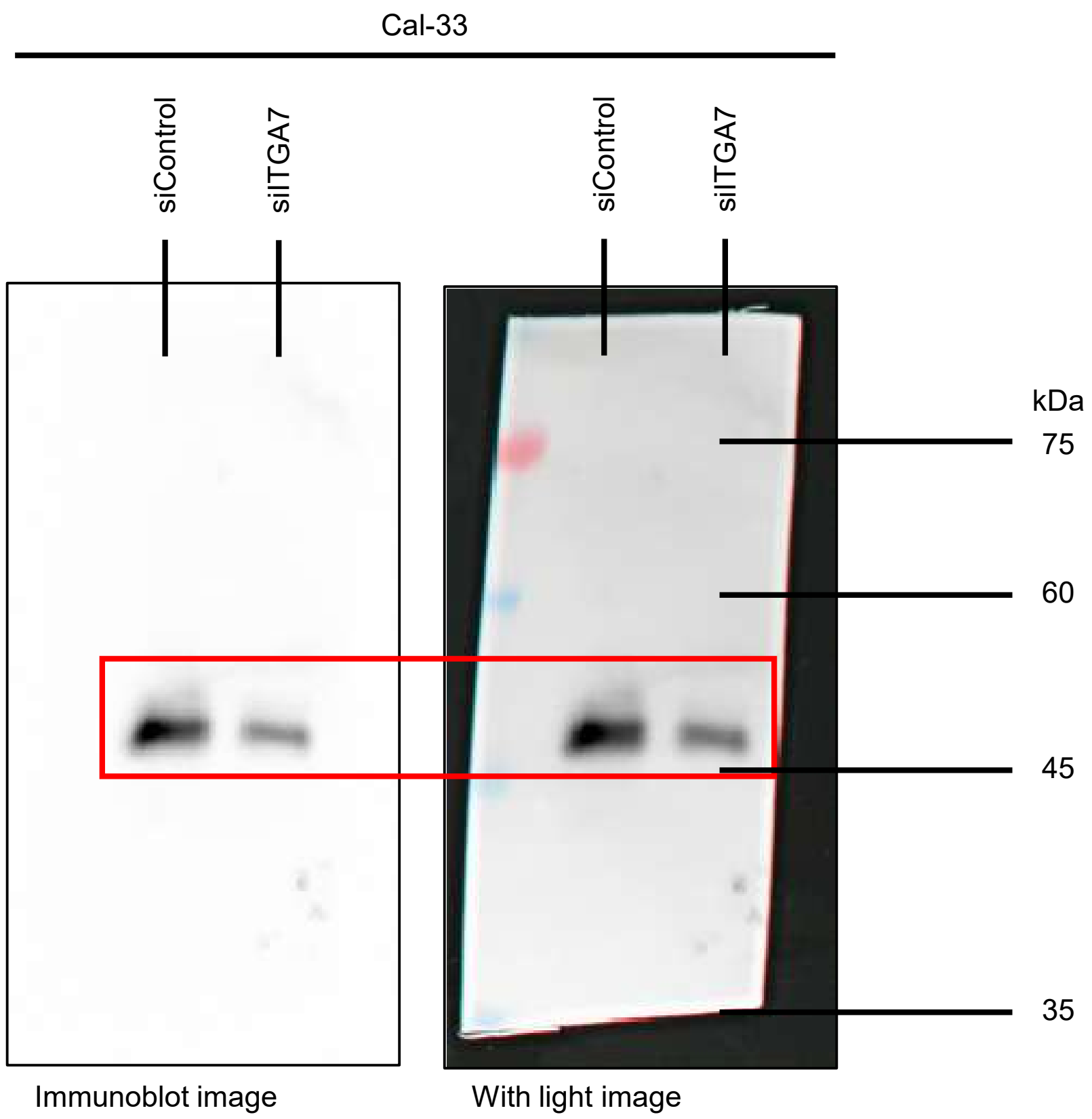

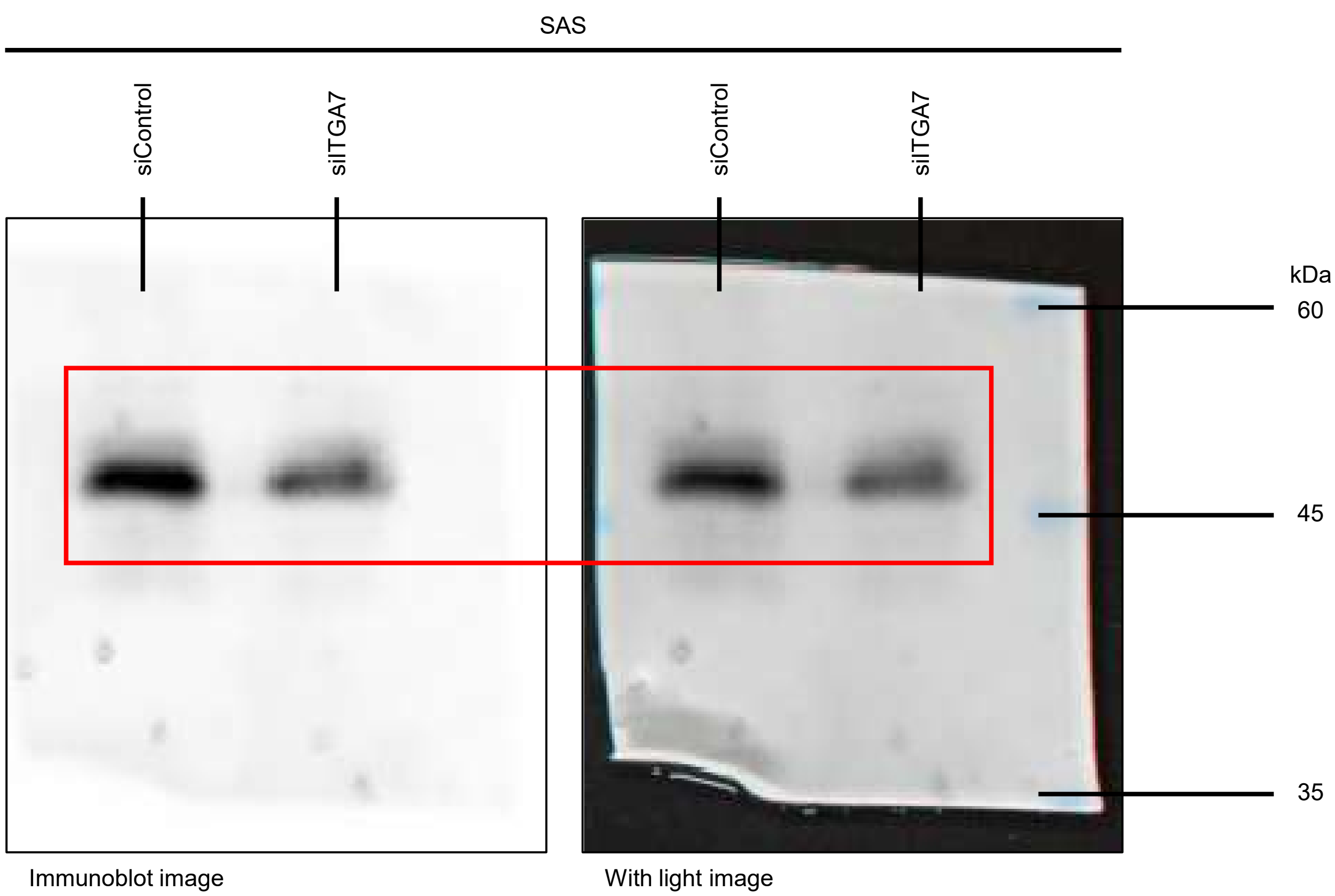

Area cropped on the article figure

p-FOXO1 (Ser256)

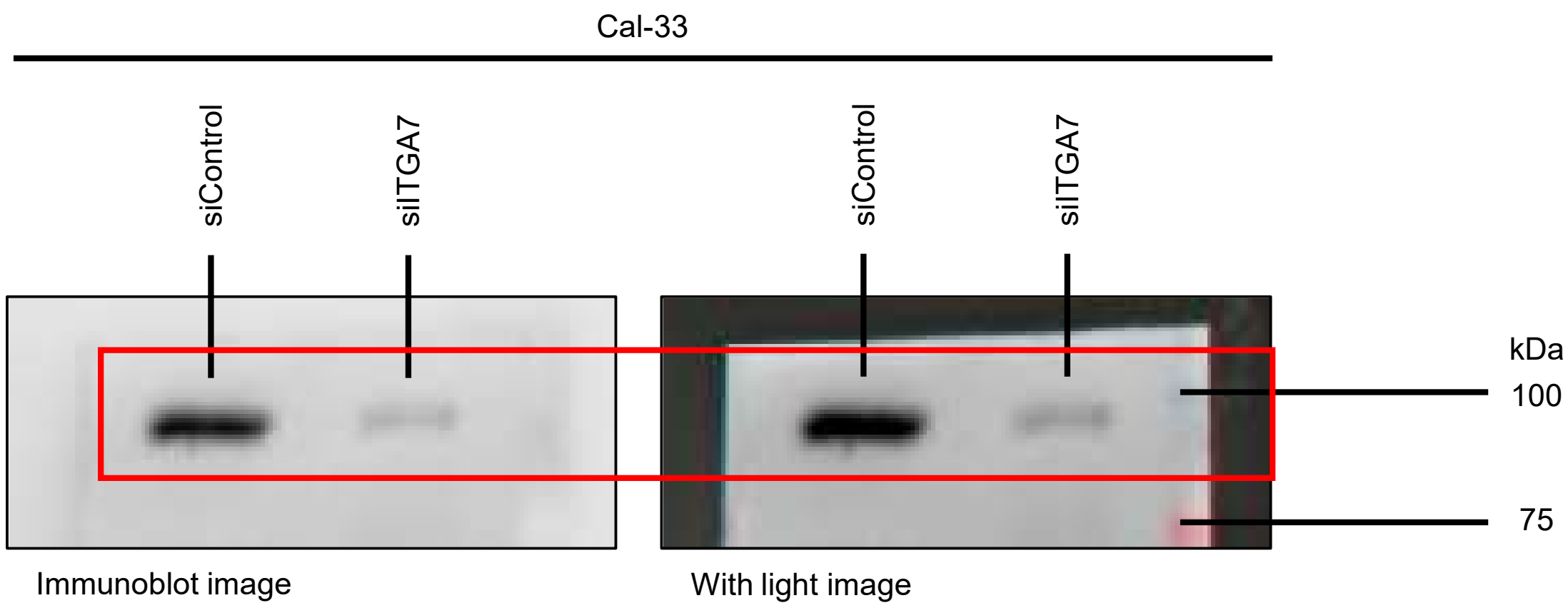

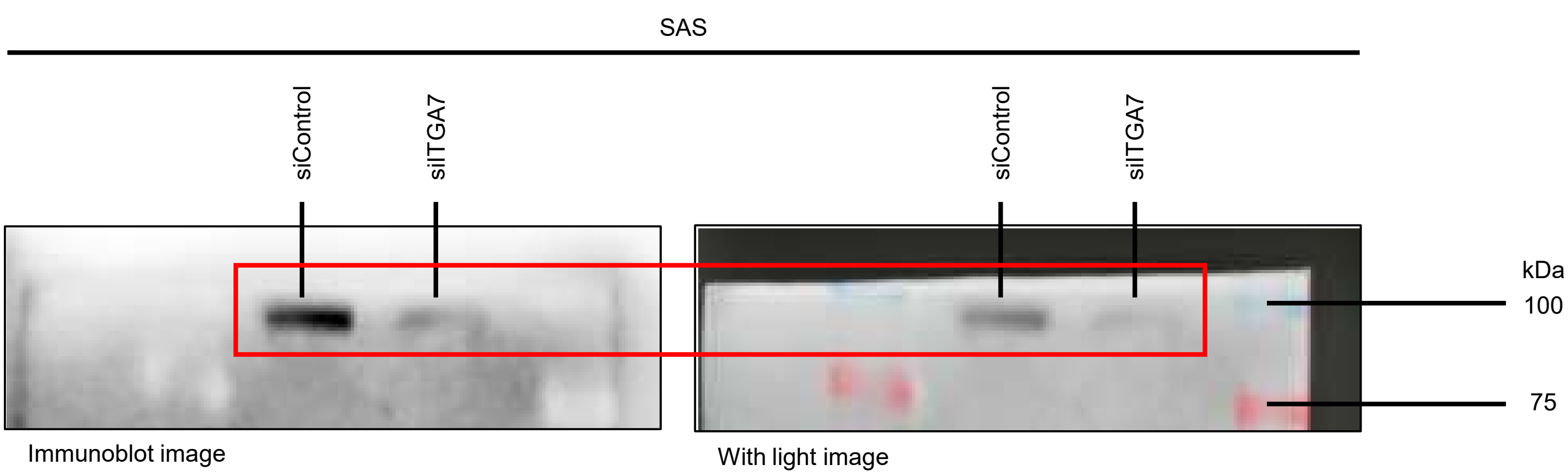

Area cropped on the article figure

Cyclin D1

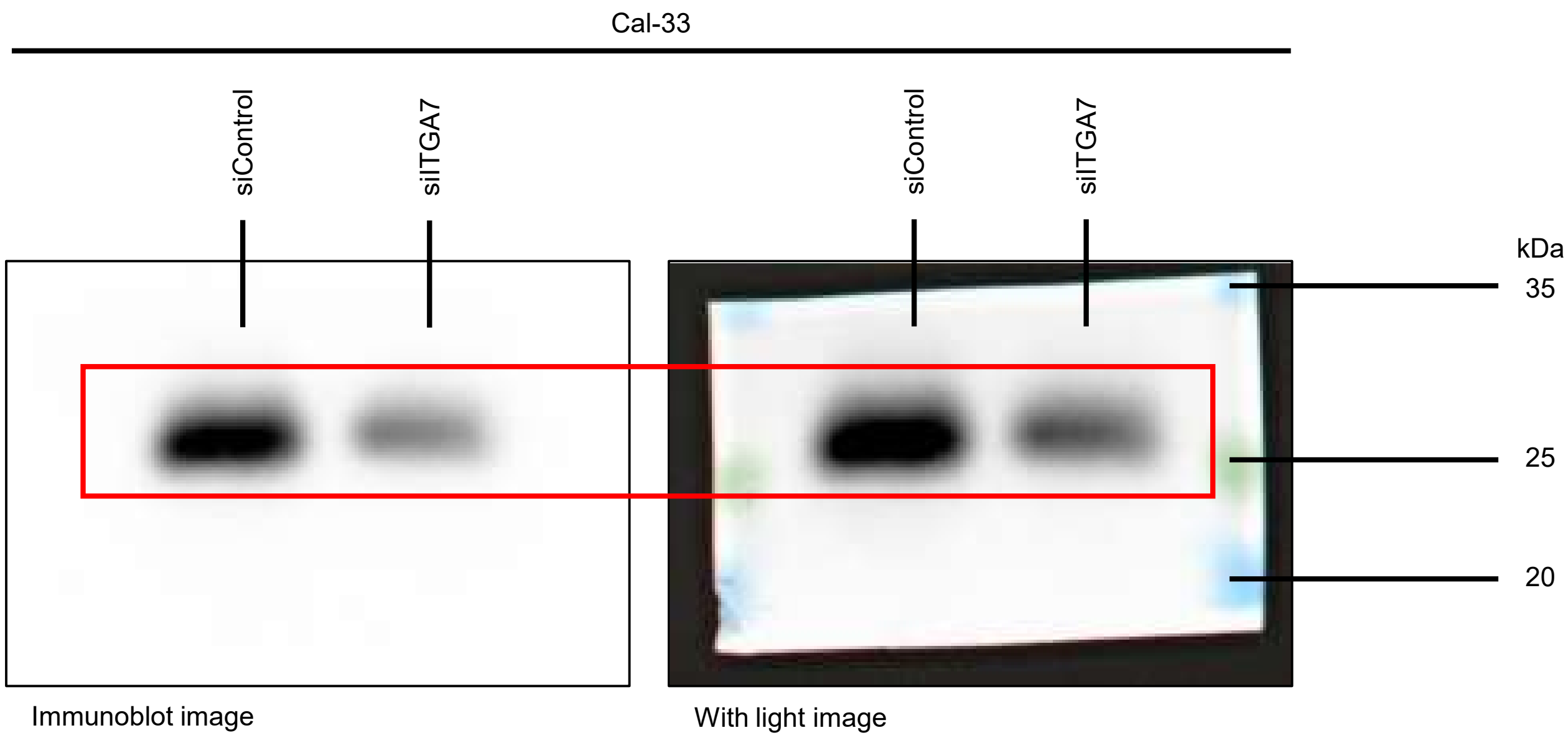

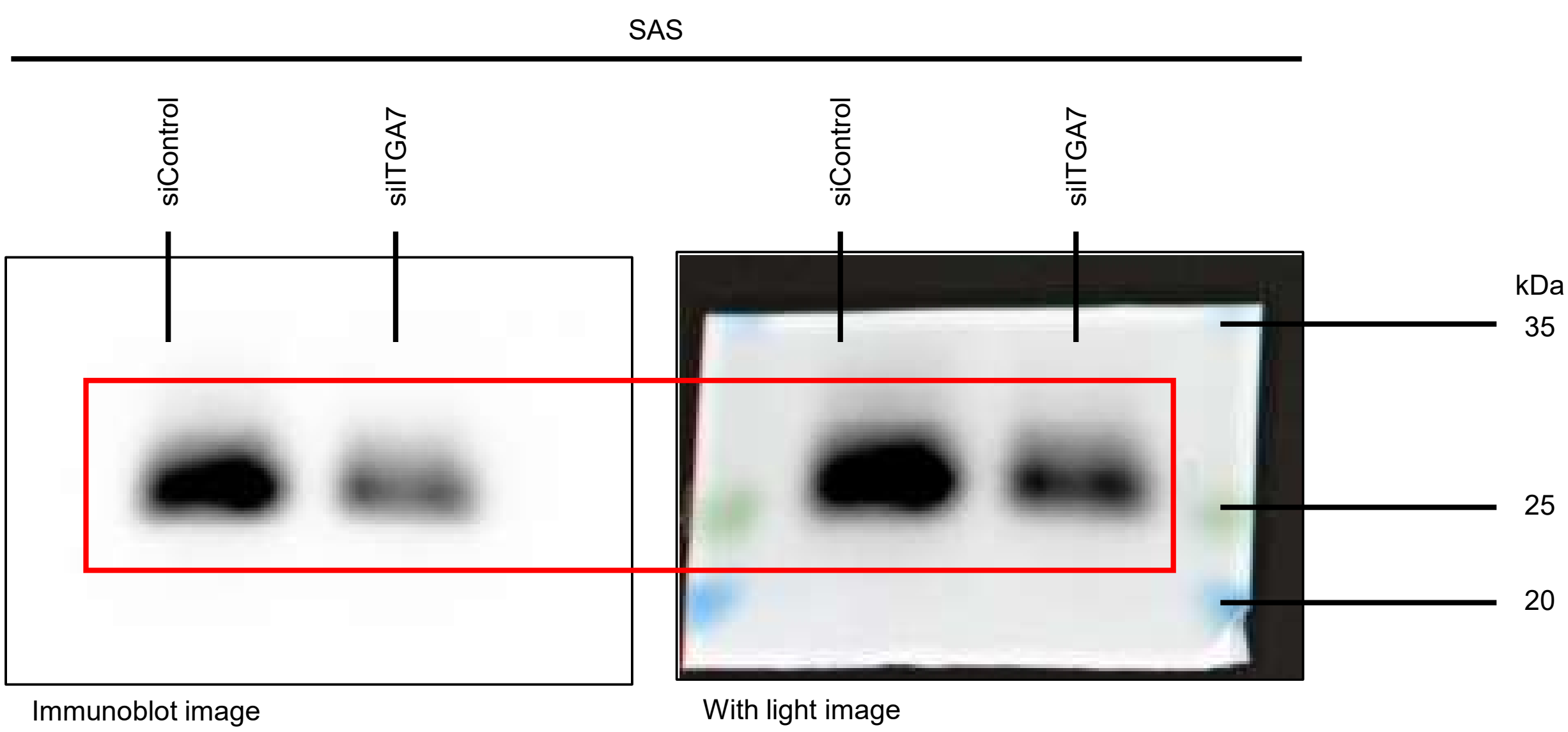

Area cropped on the article figure

Cyclin E

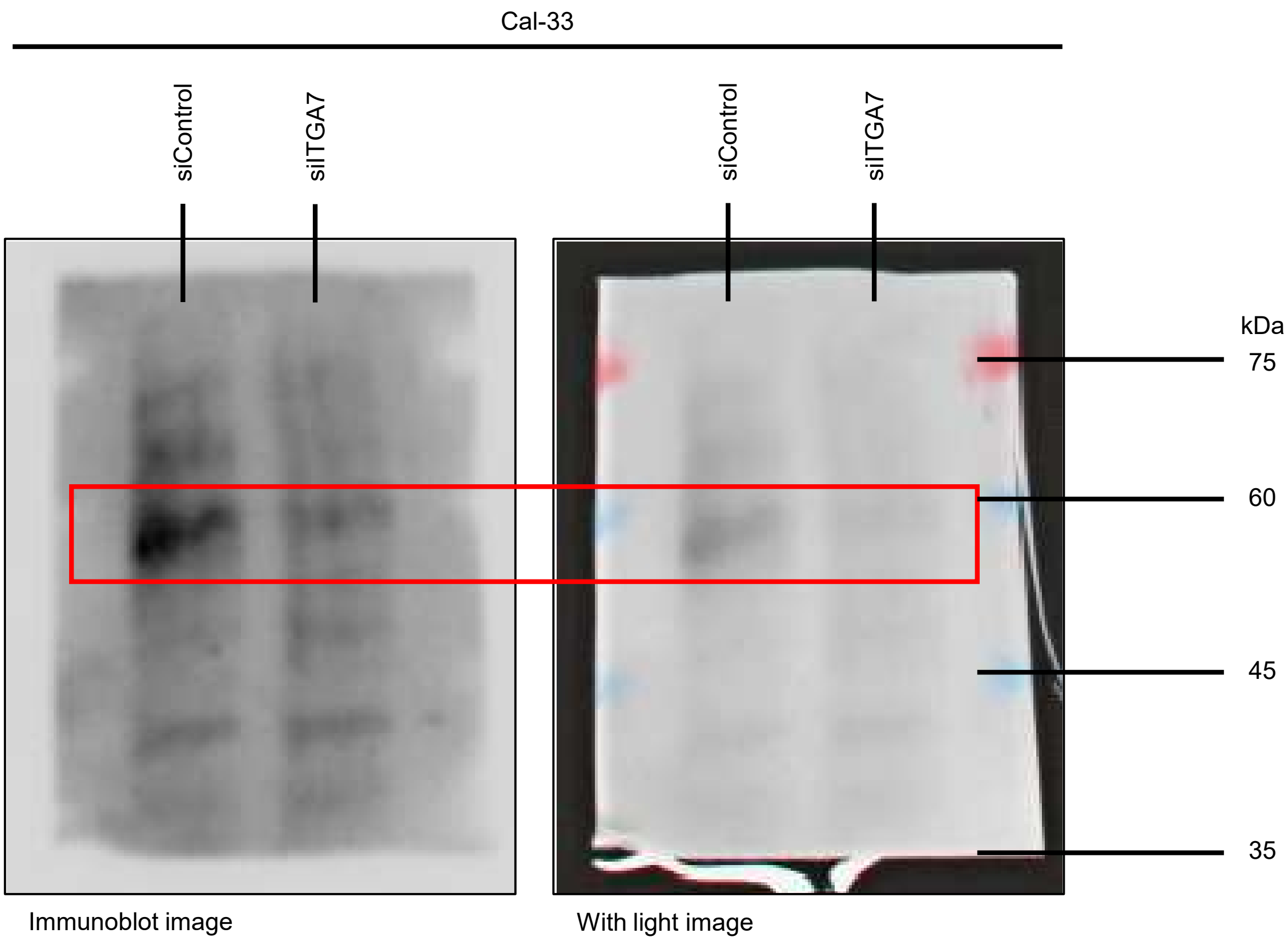

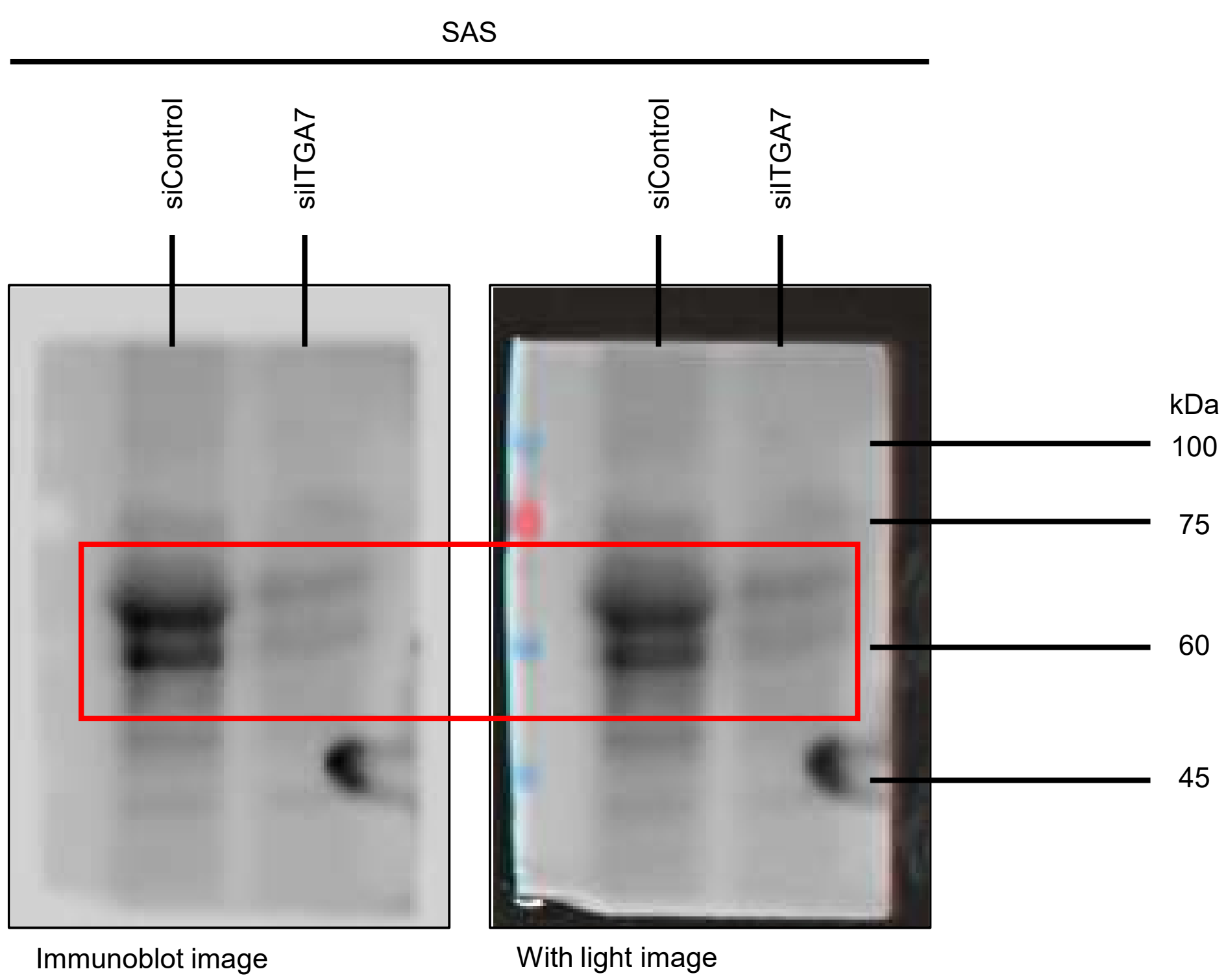

Area cropped on the article figure

Cyclin A

Immunoblot image

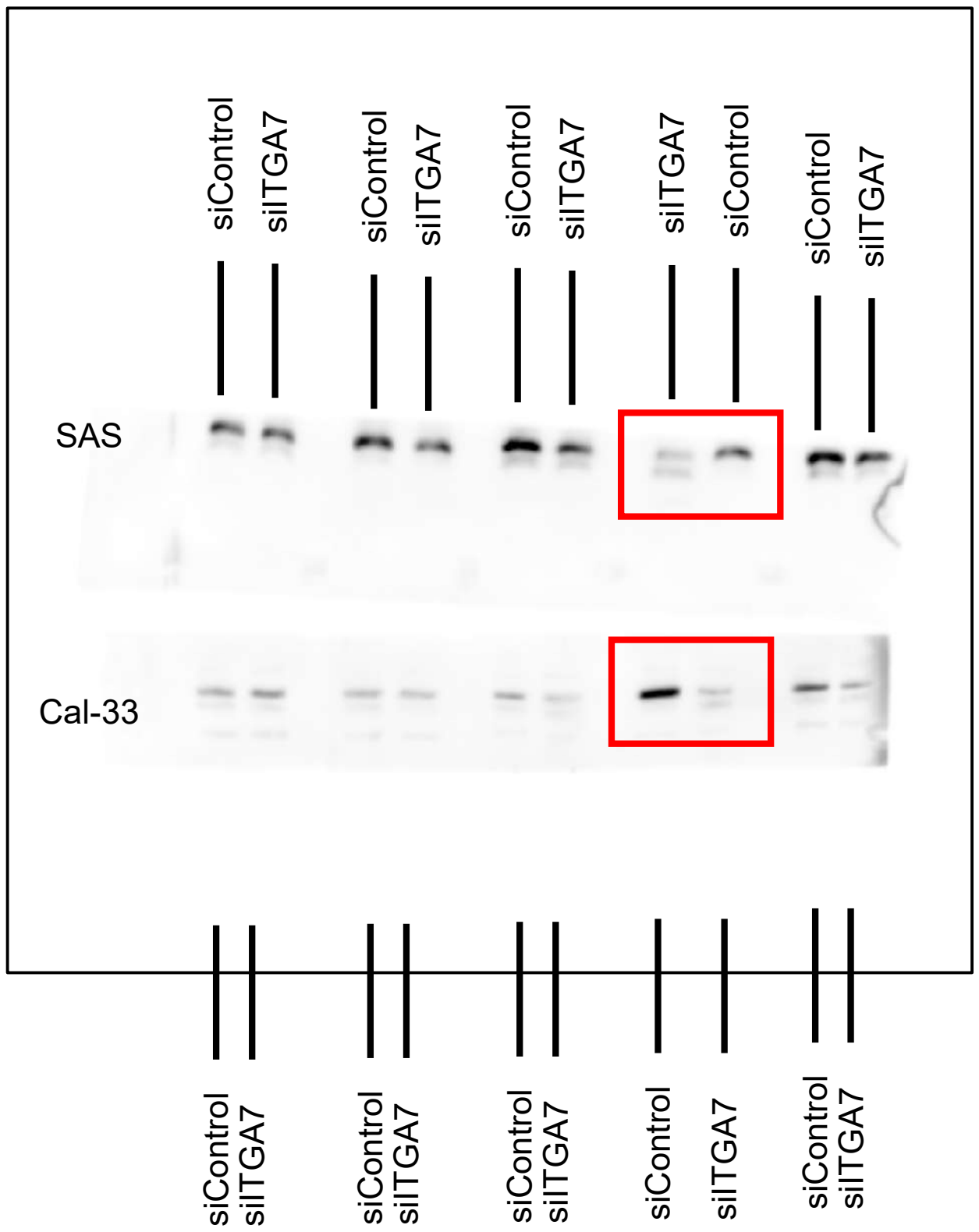

With light image

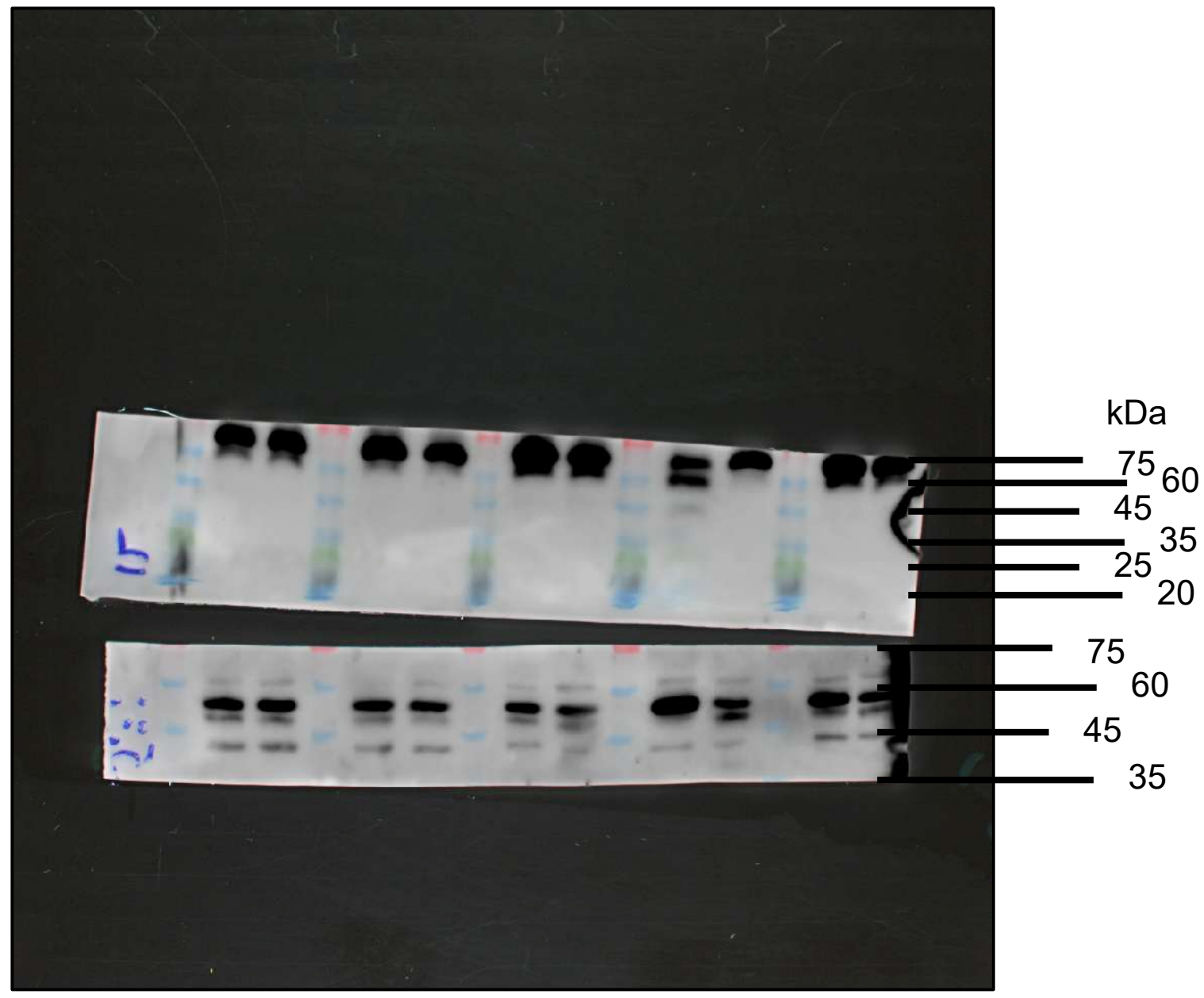

Area cropped on the article figure

Cyclin B1

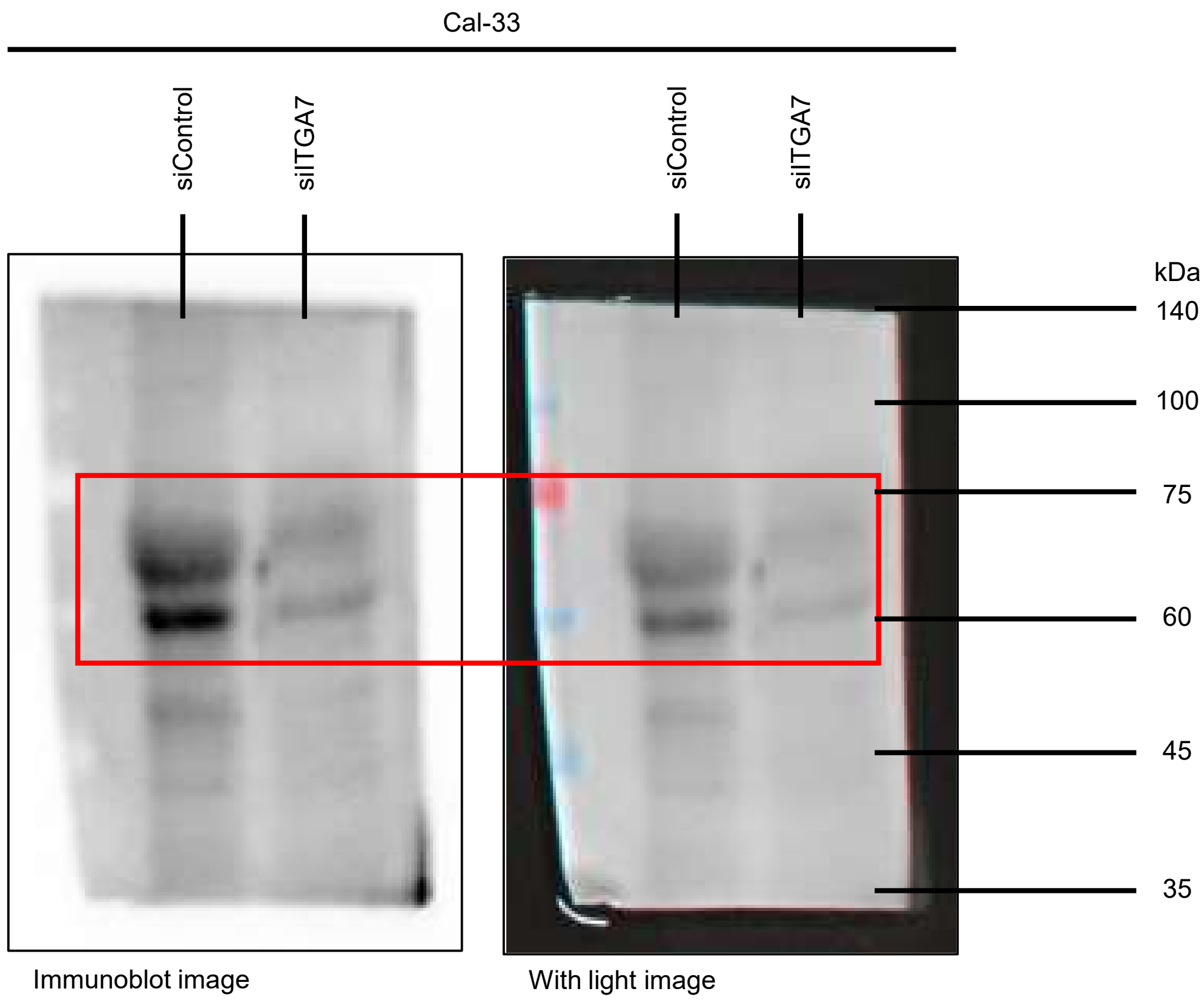

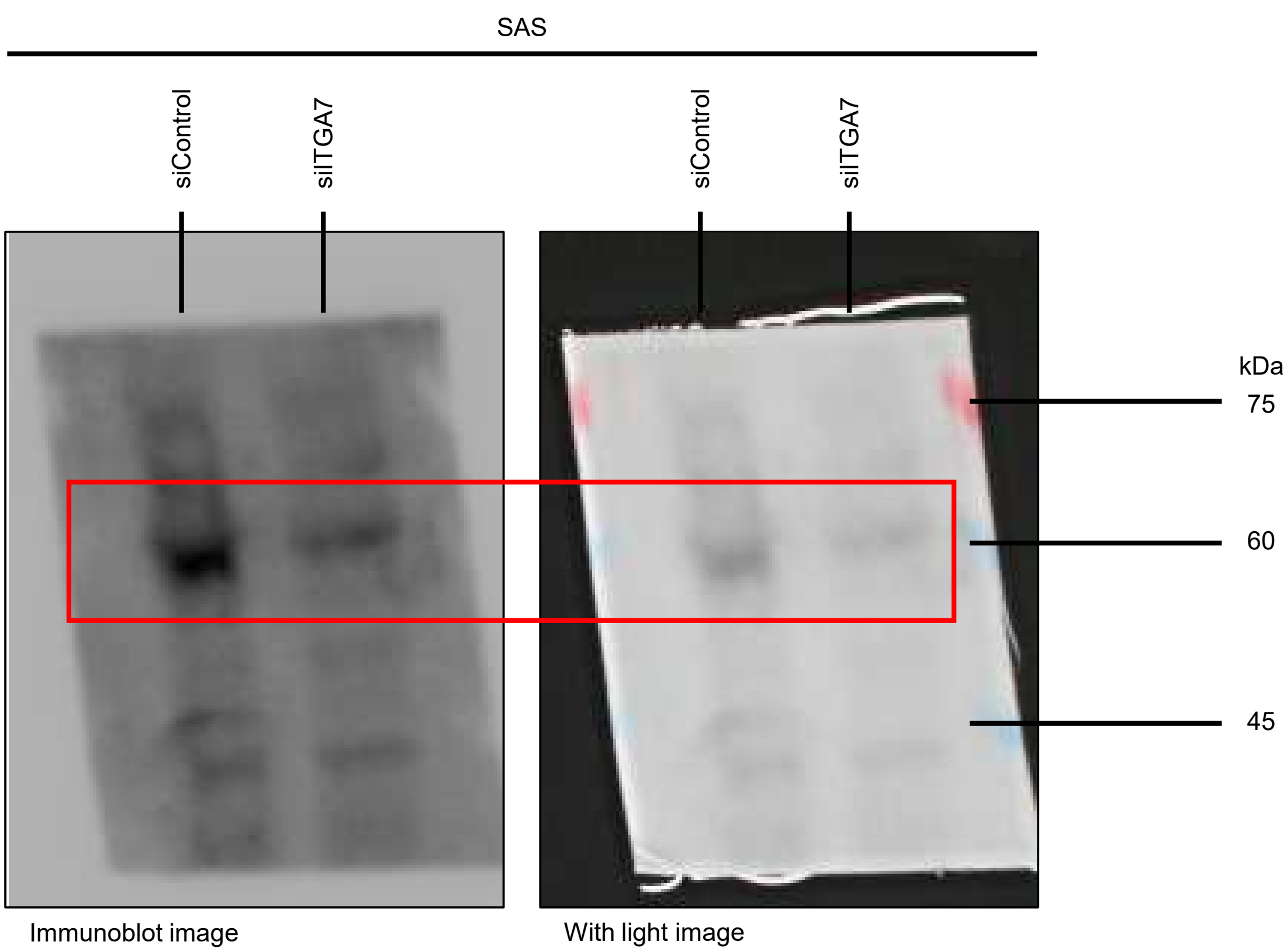

Area cropped on the article figure

CDC25A

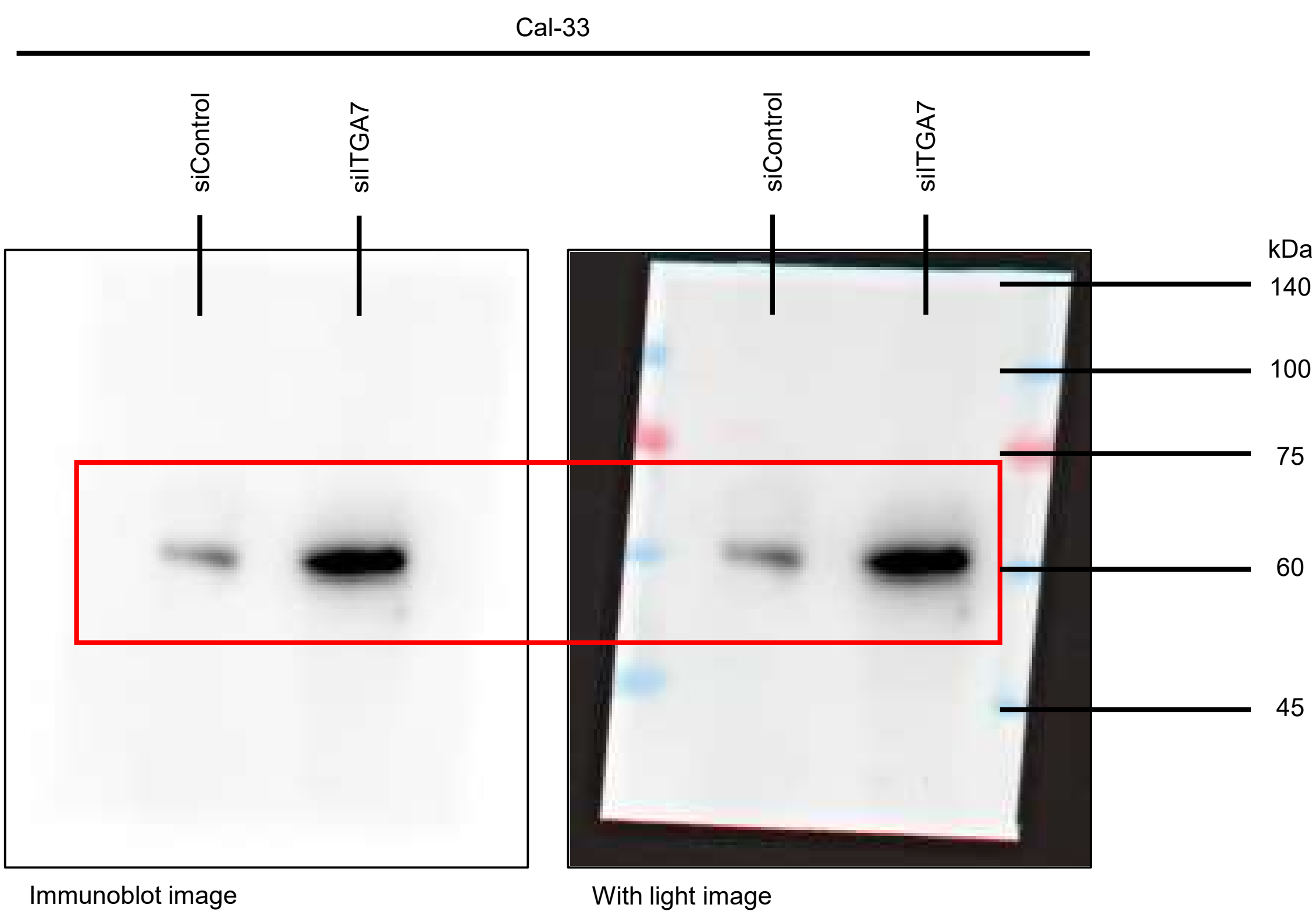

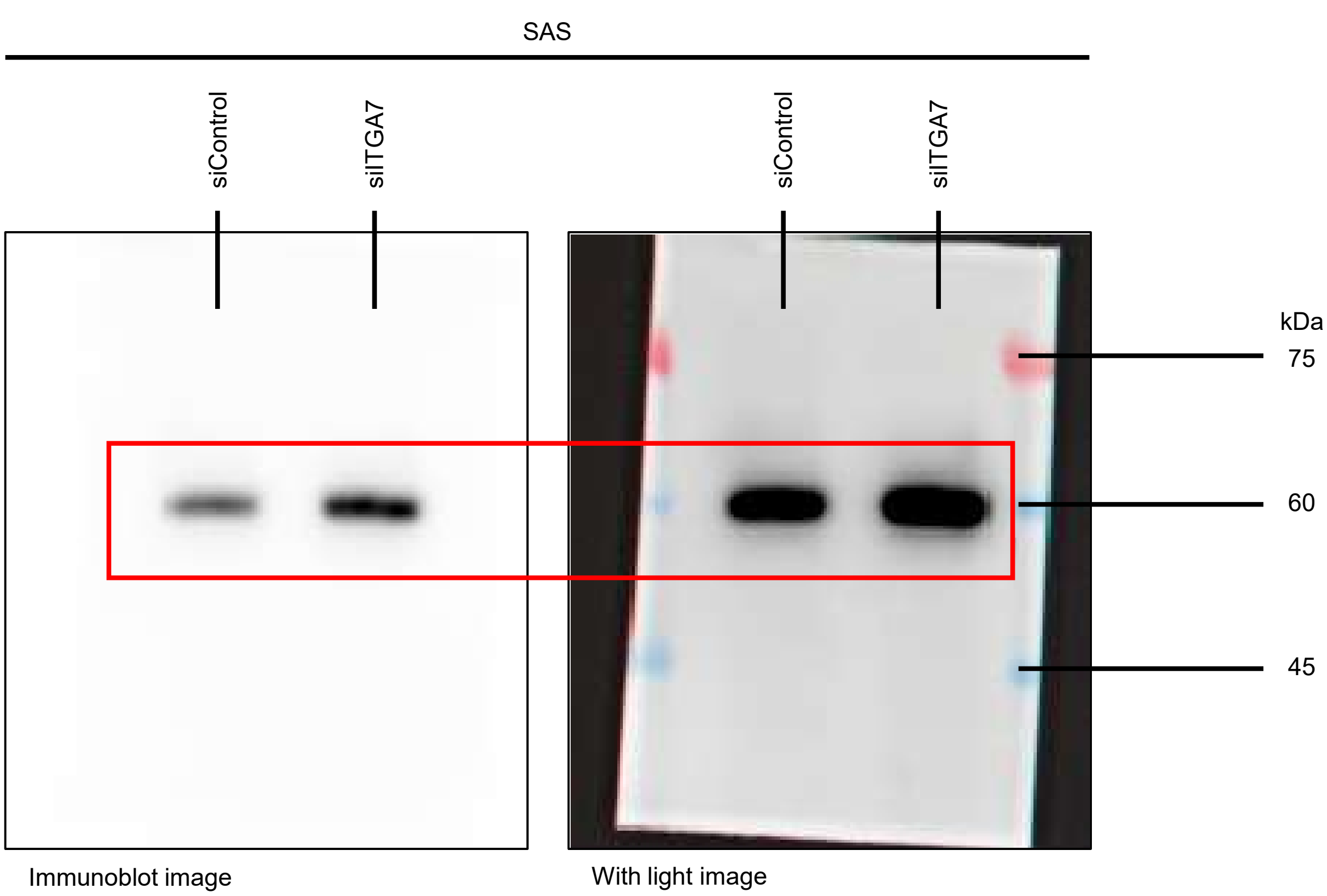

Area cropped on the article figure

p-CDC25A (Ser124)

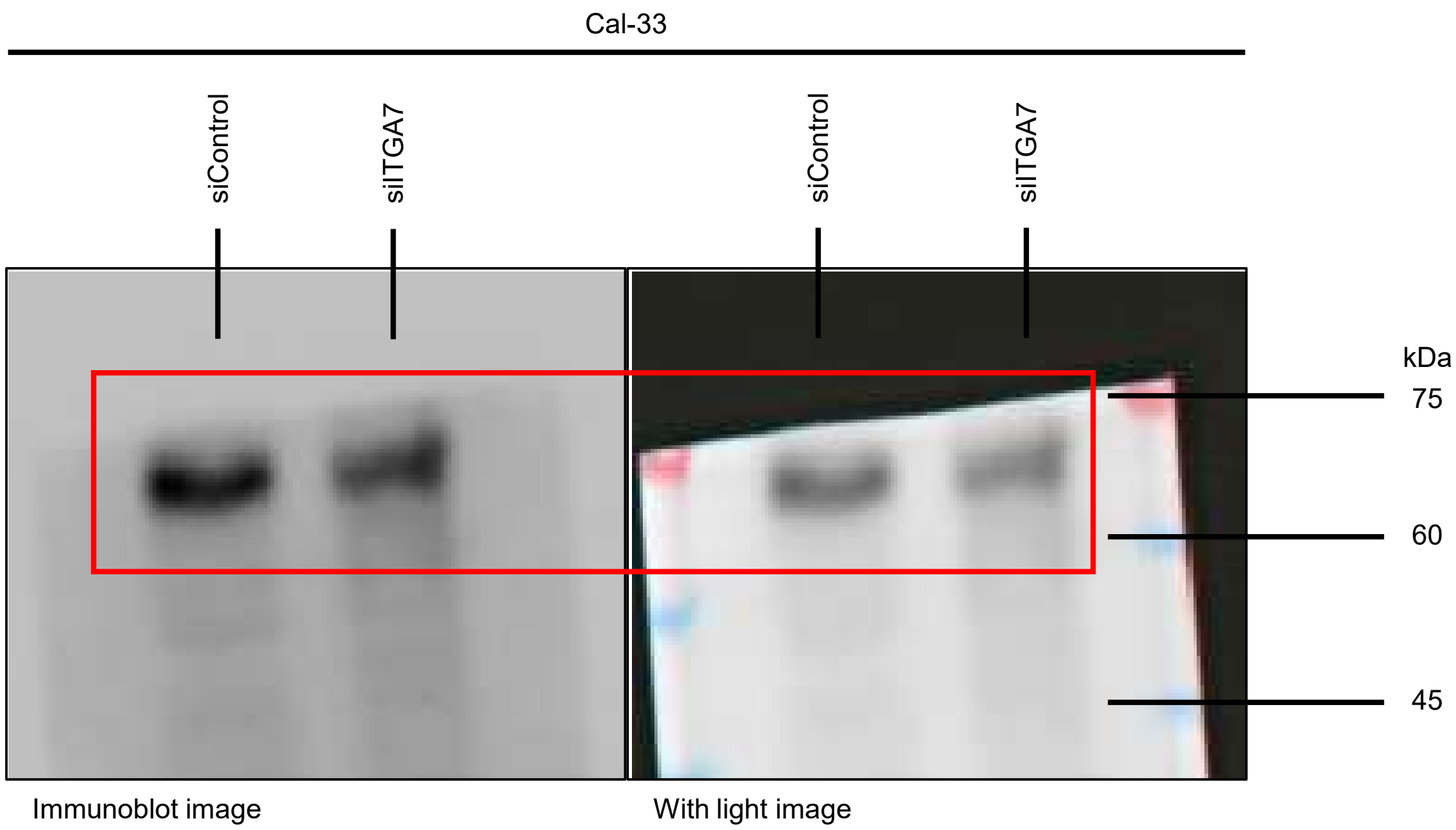

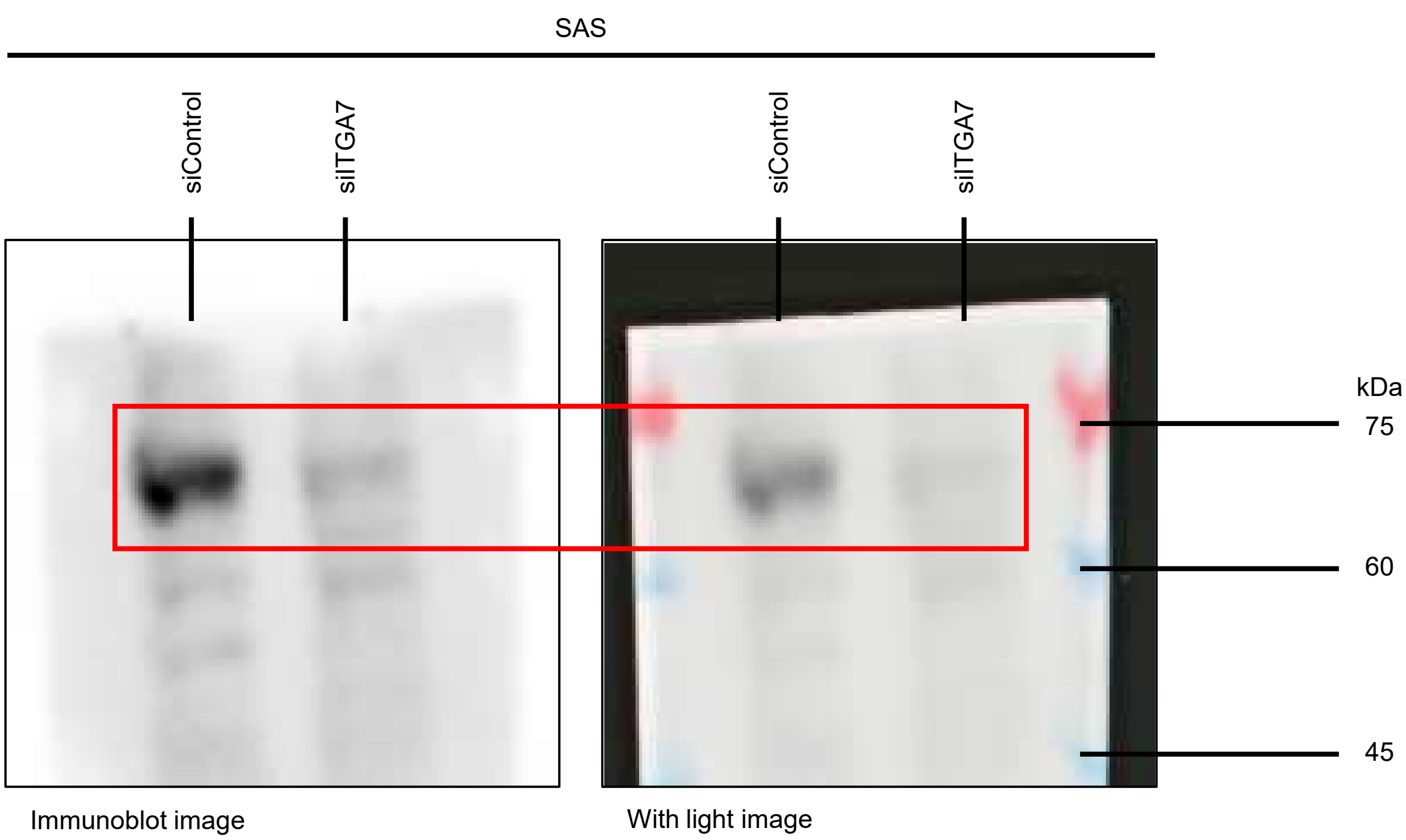

Area cropped on the article figure

CDC25C

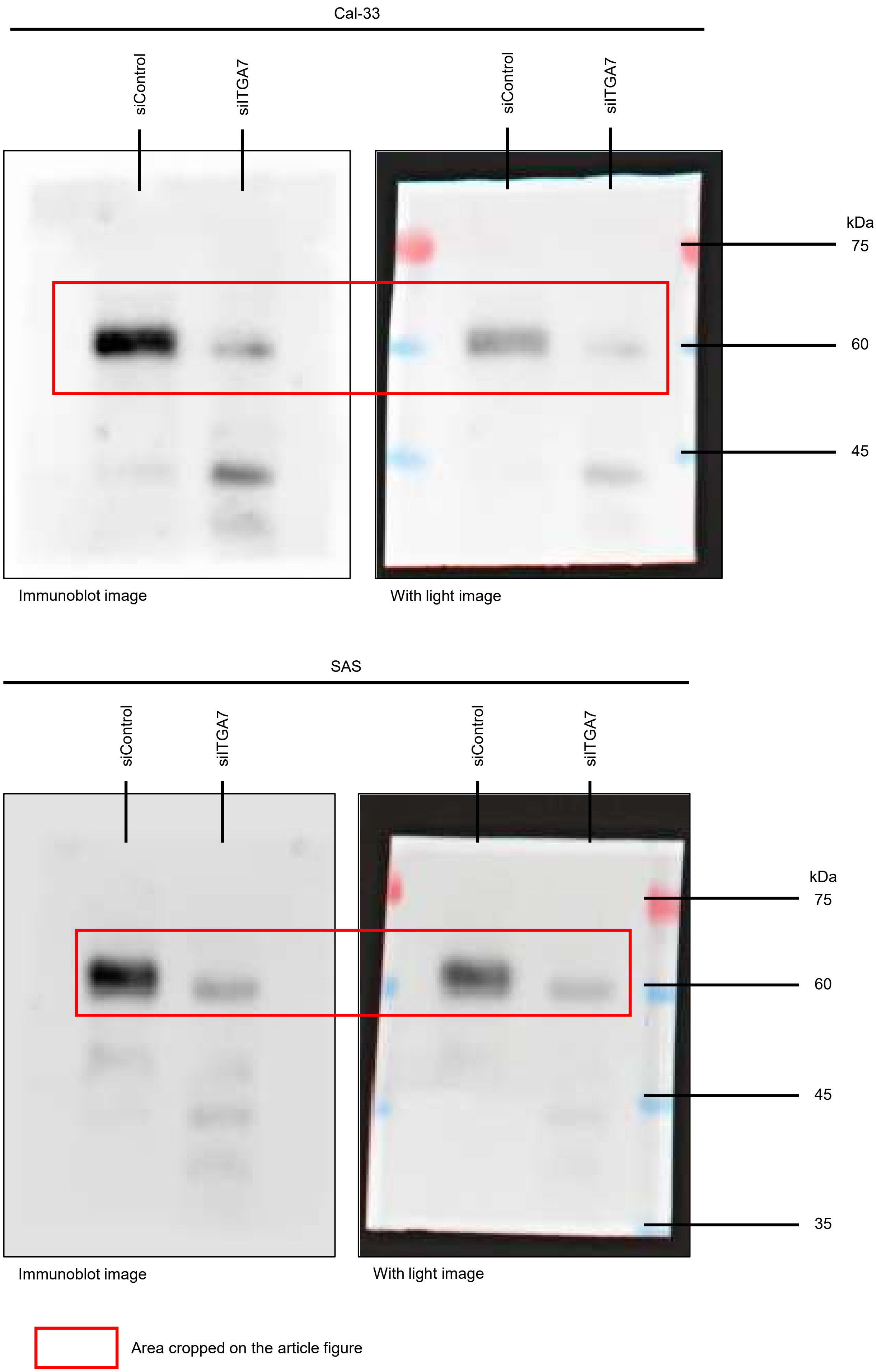
Cal-33
siControl
siITGA7
siControl
siITGA7
kDa
75
60
45
Immunoblot image
With light image
SAS
siControl
siITGA7
siControl
siITGA7
kDa
75
60
45
35
Immunoblot image
With light image
Area cropped on the article figure

Aurora A

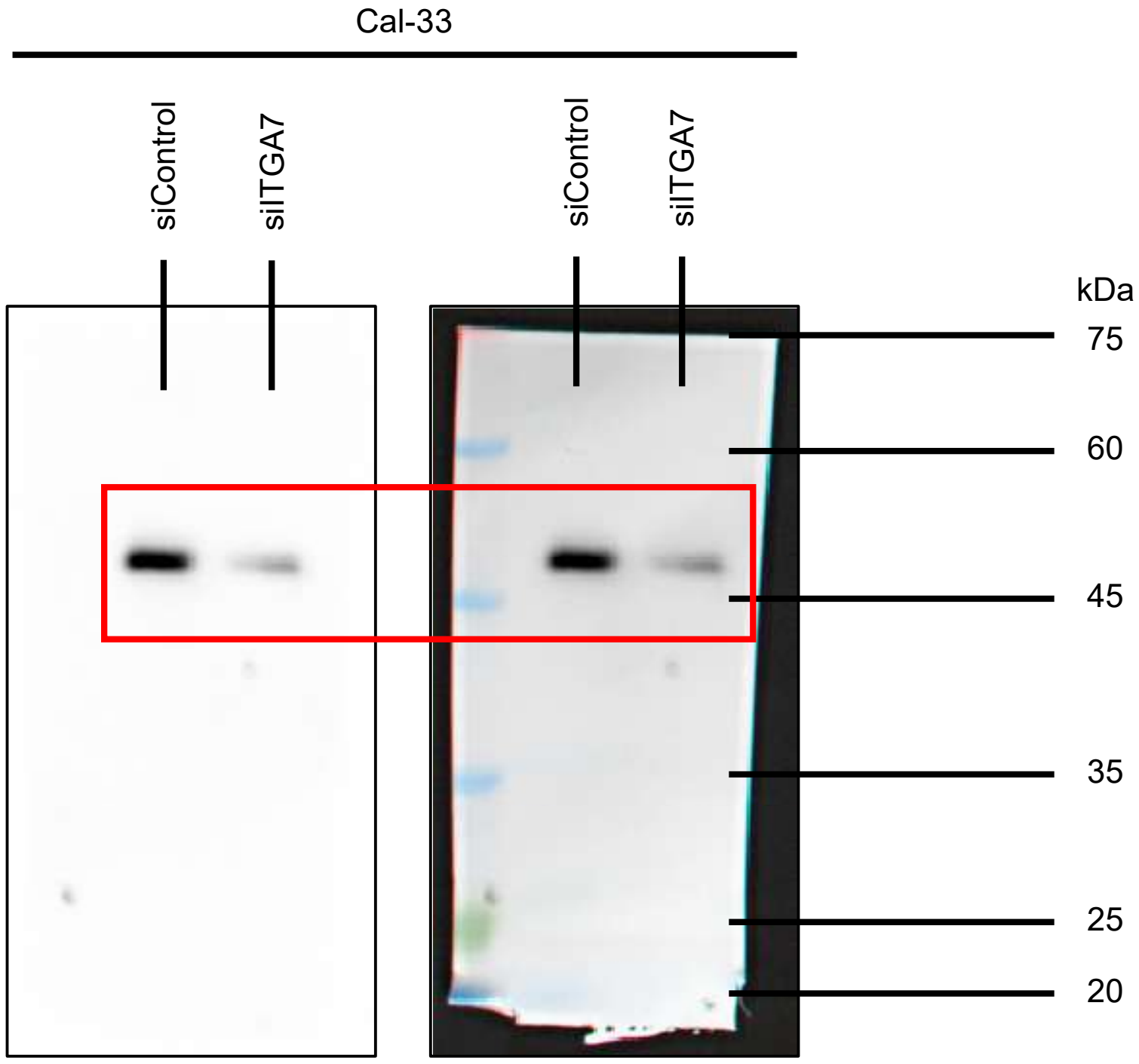

Immunoblot image With light image

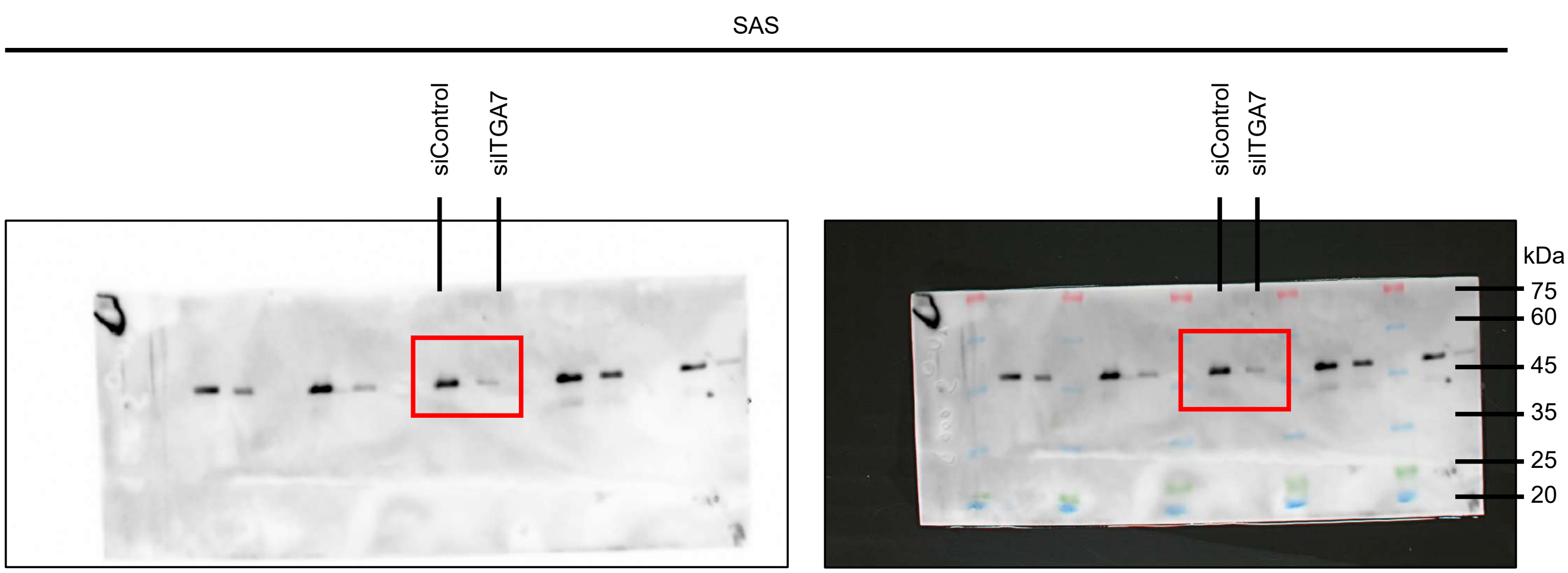

Immunoblot image With light image

Area cropped on the article figure

p-Aurora A (Thr288)

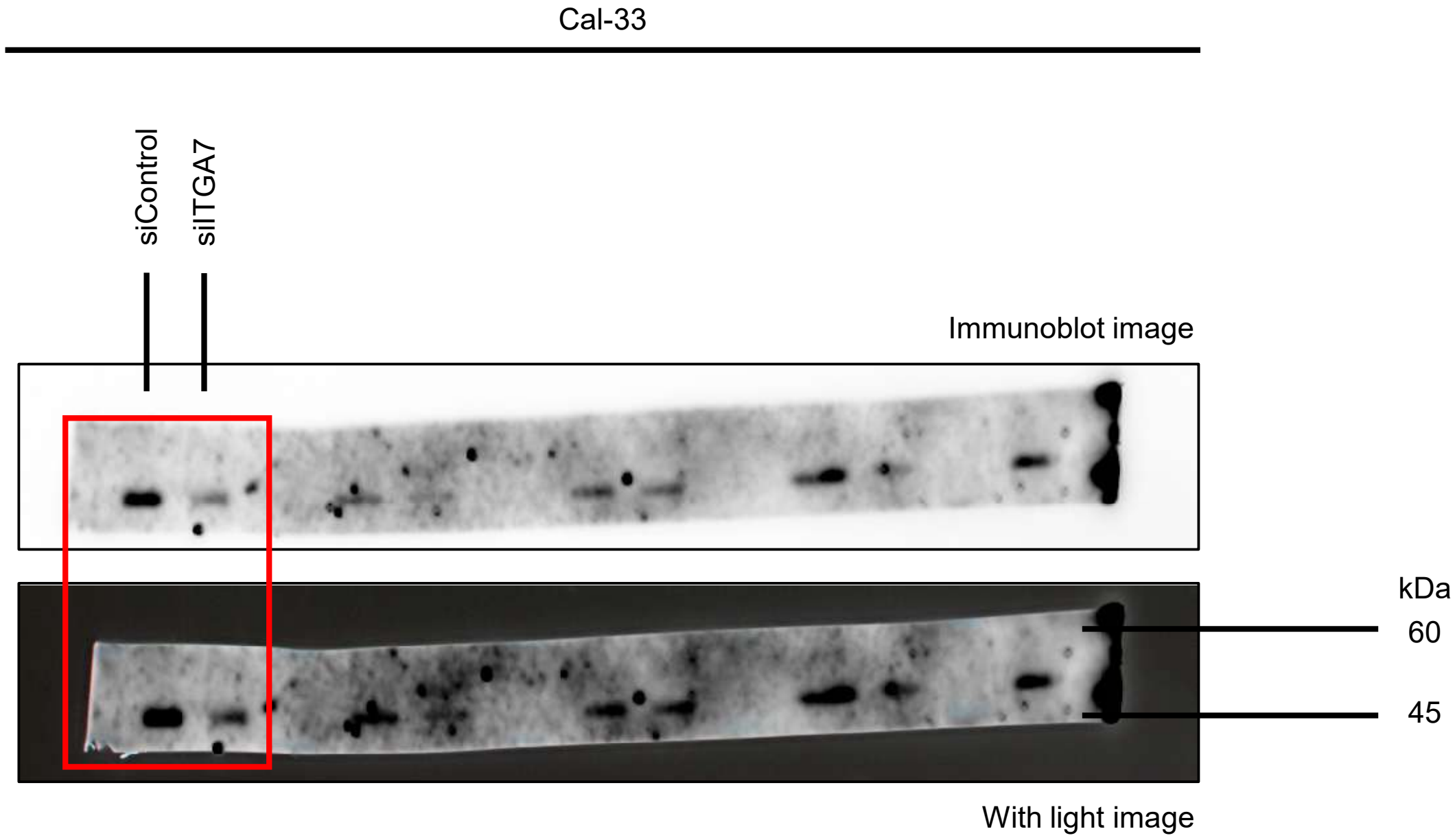

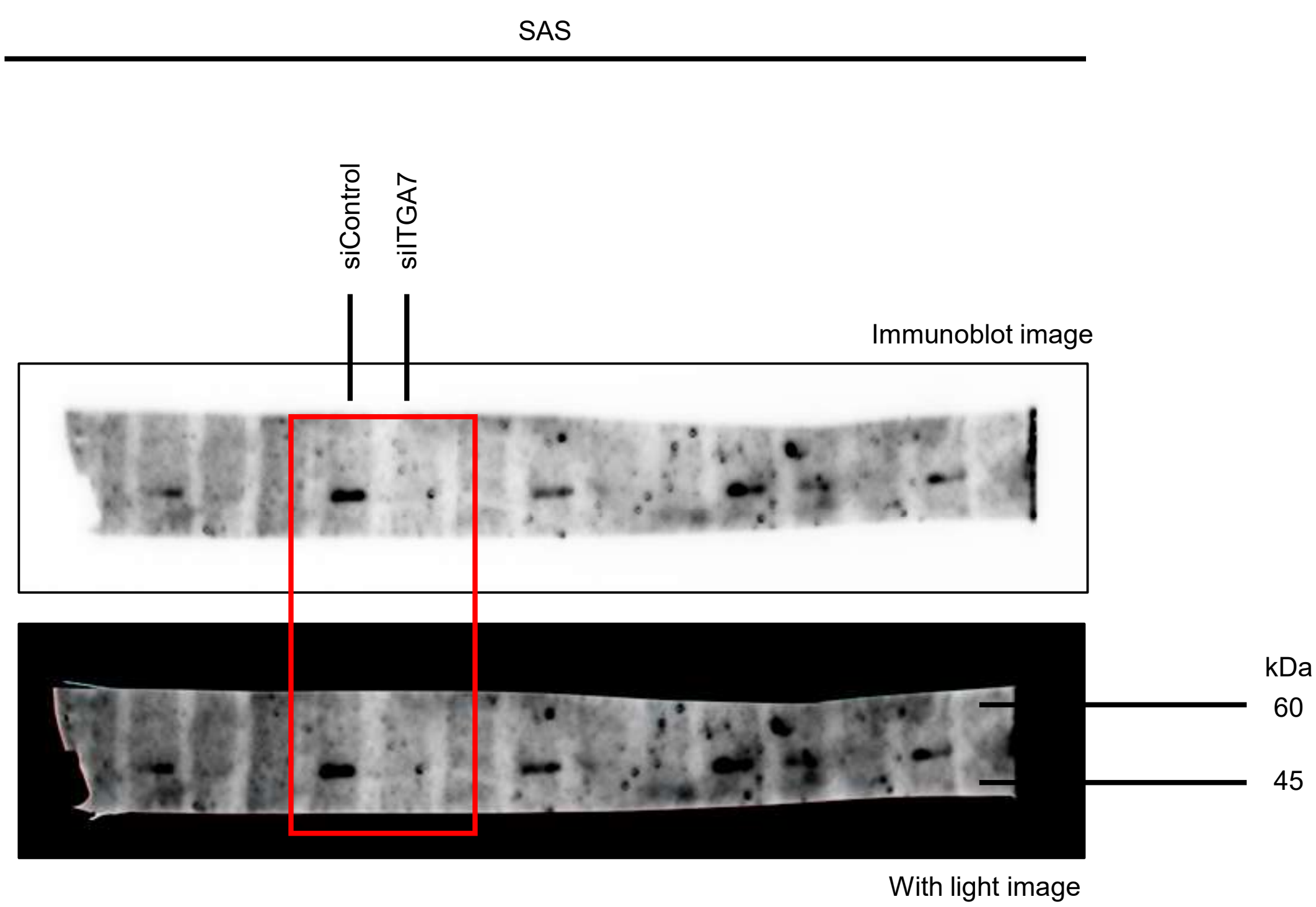

Area cropped on the article figure

Ki-67

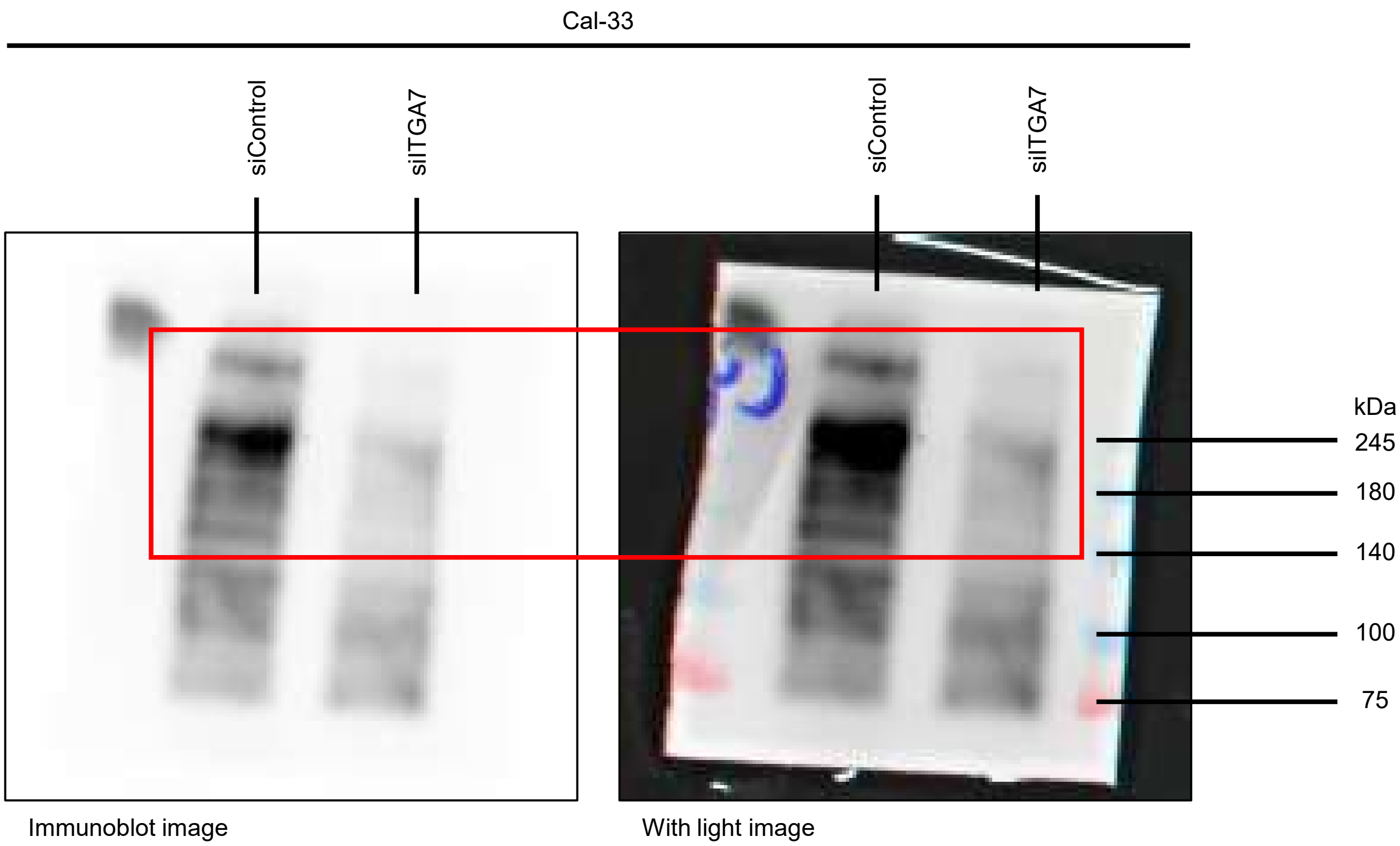

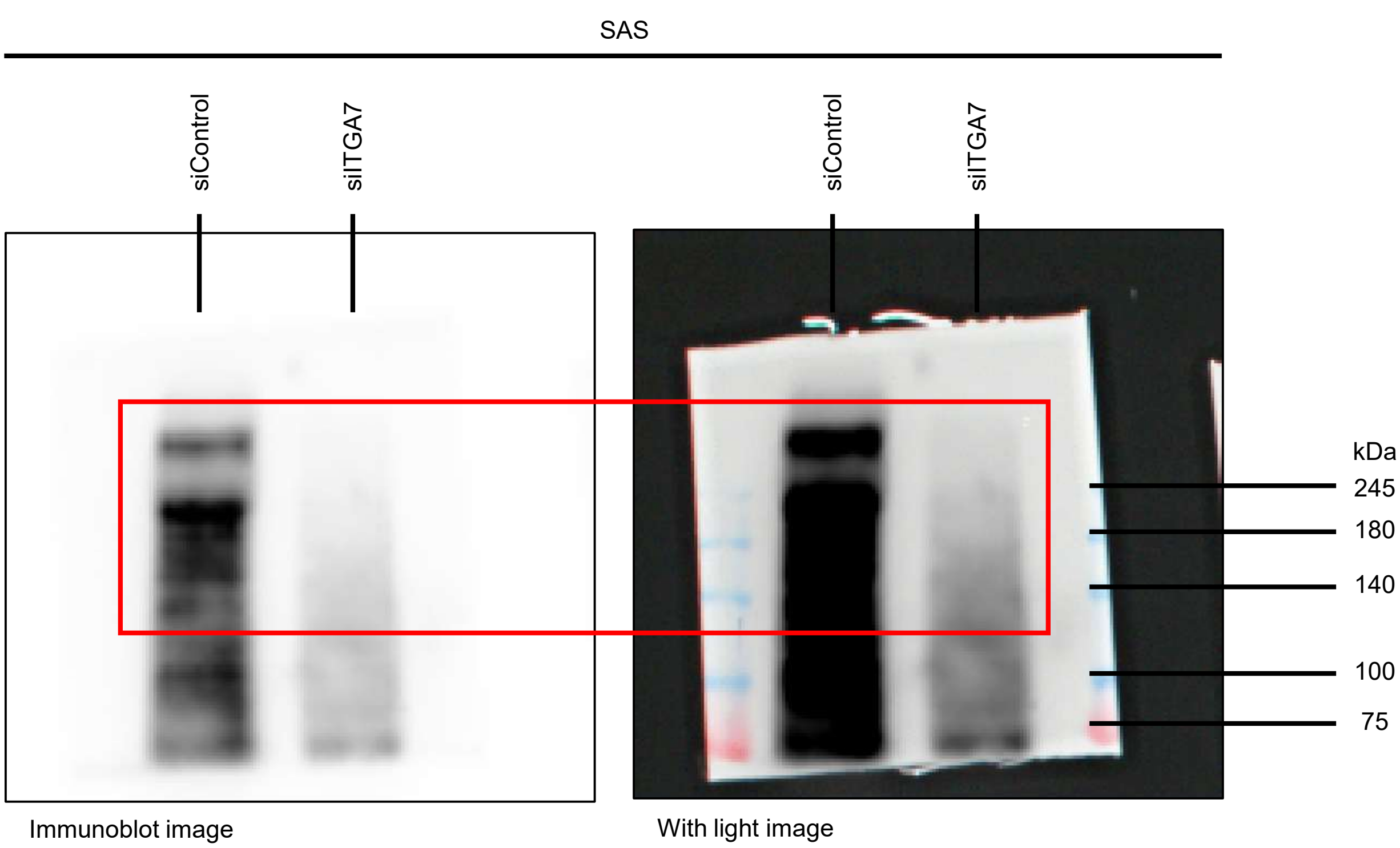

Area cropped on the article figure

ATF-1

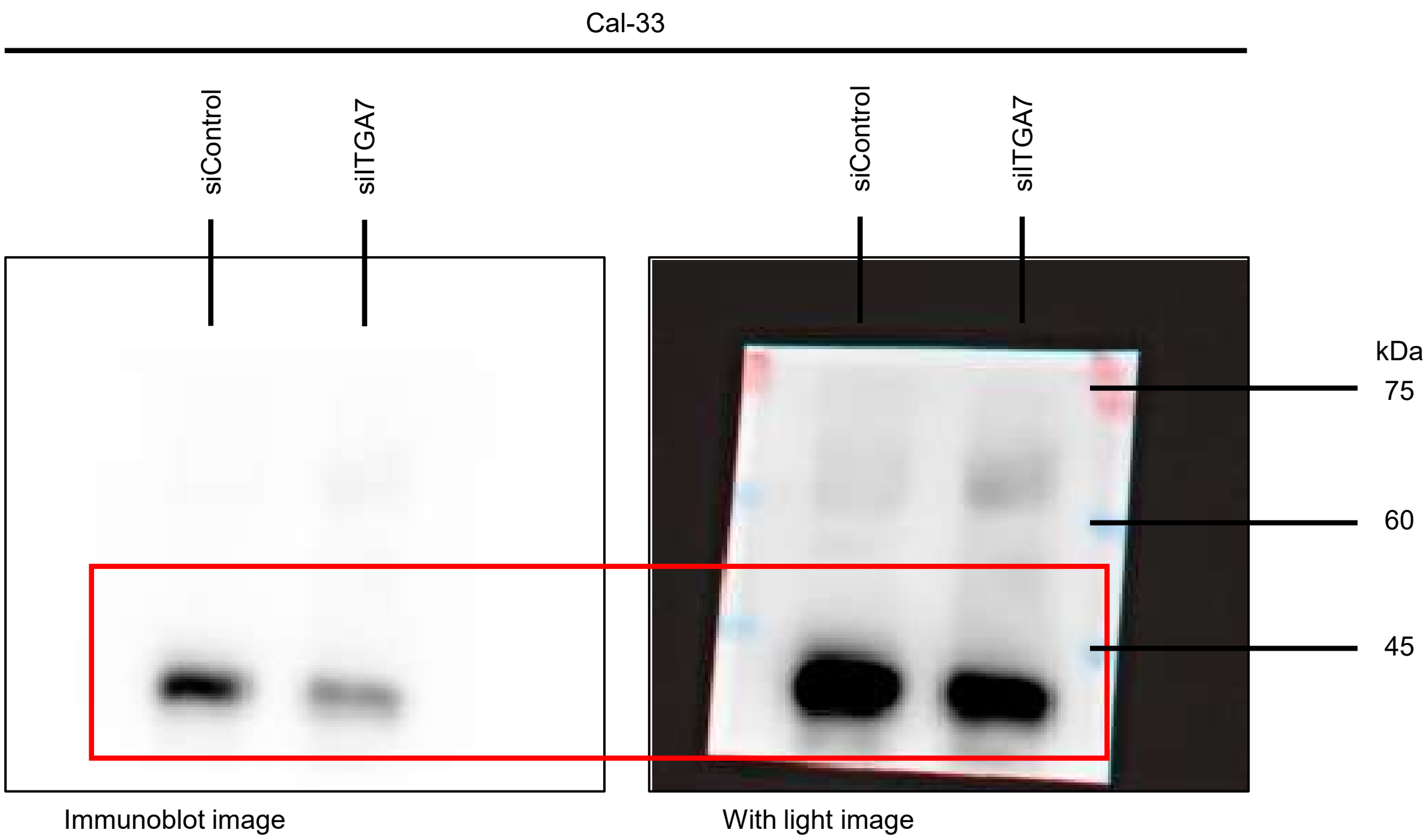

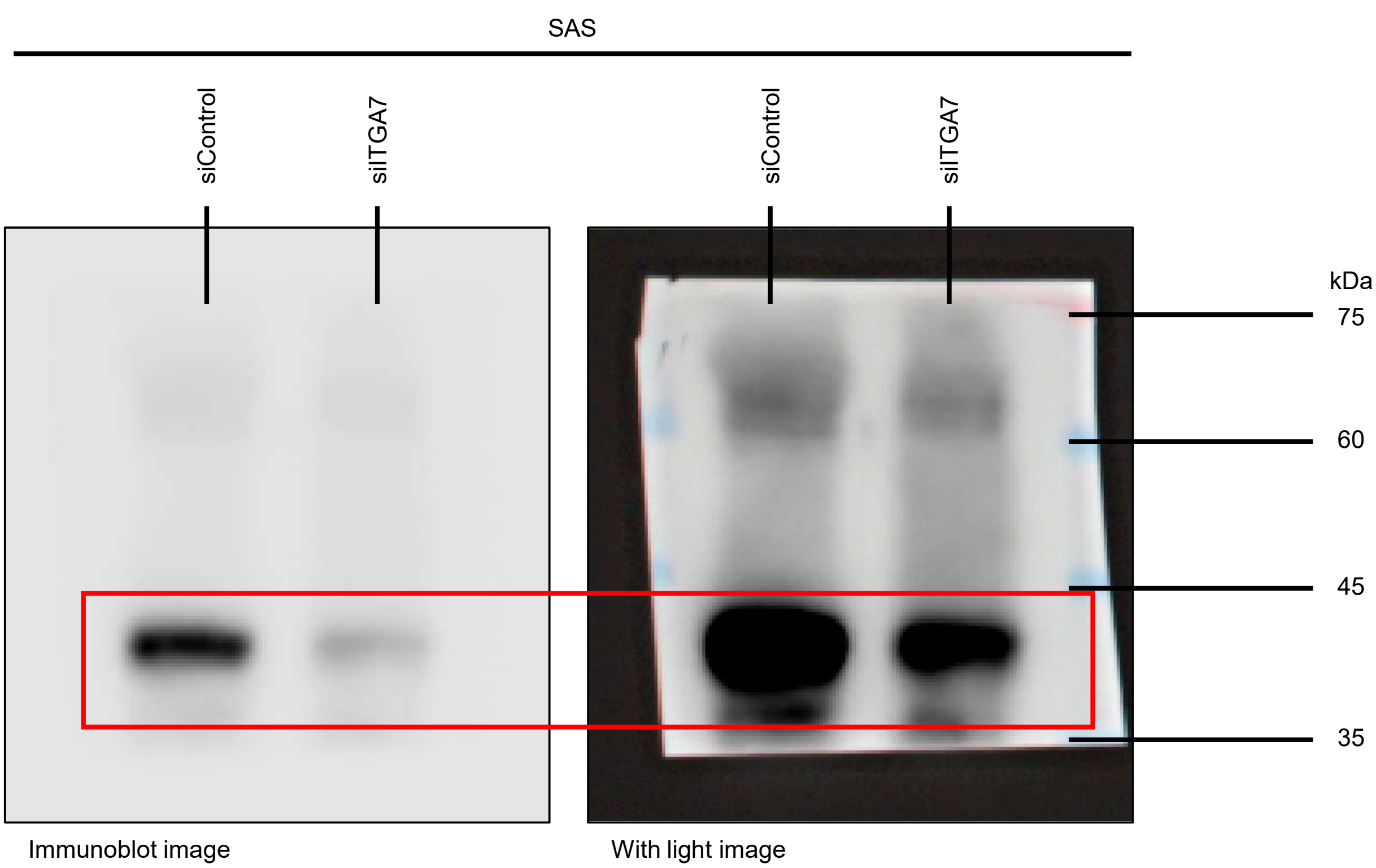

Area cropped on the article figure

p-ATF-2

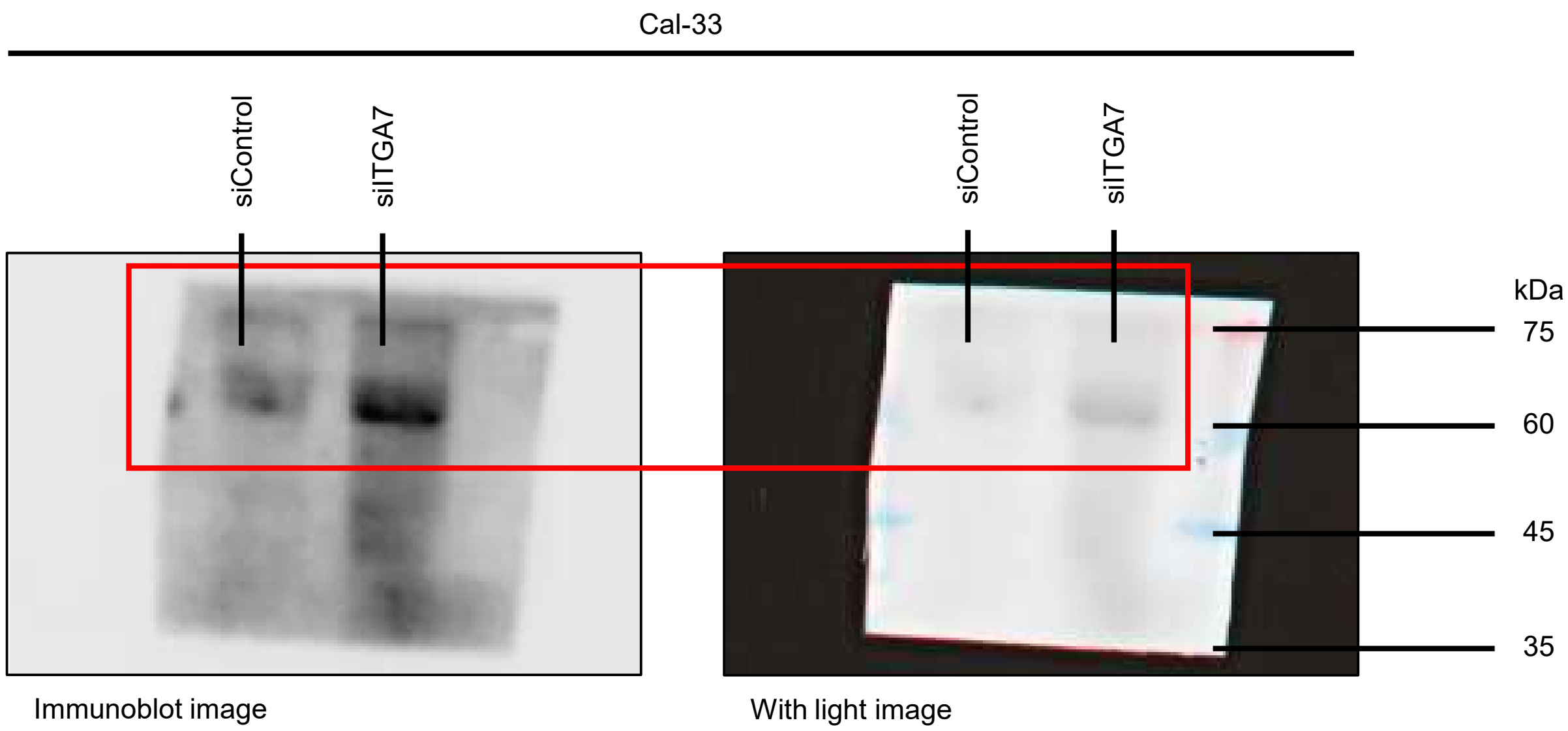

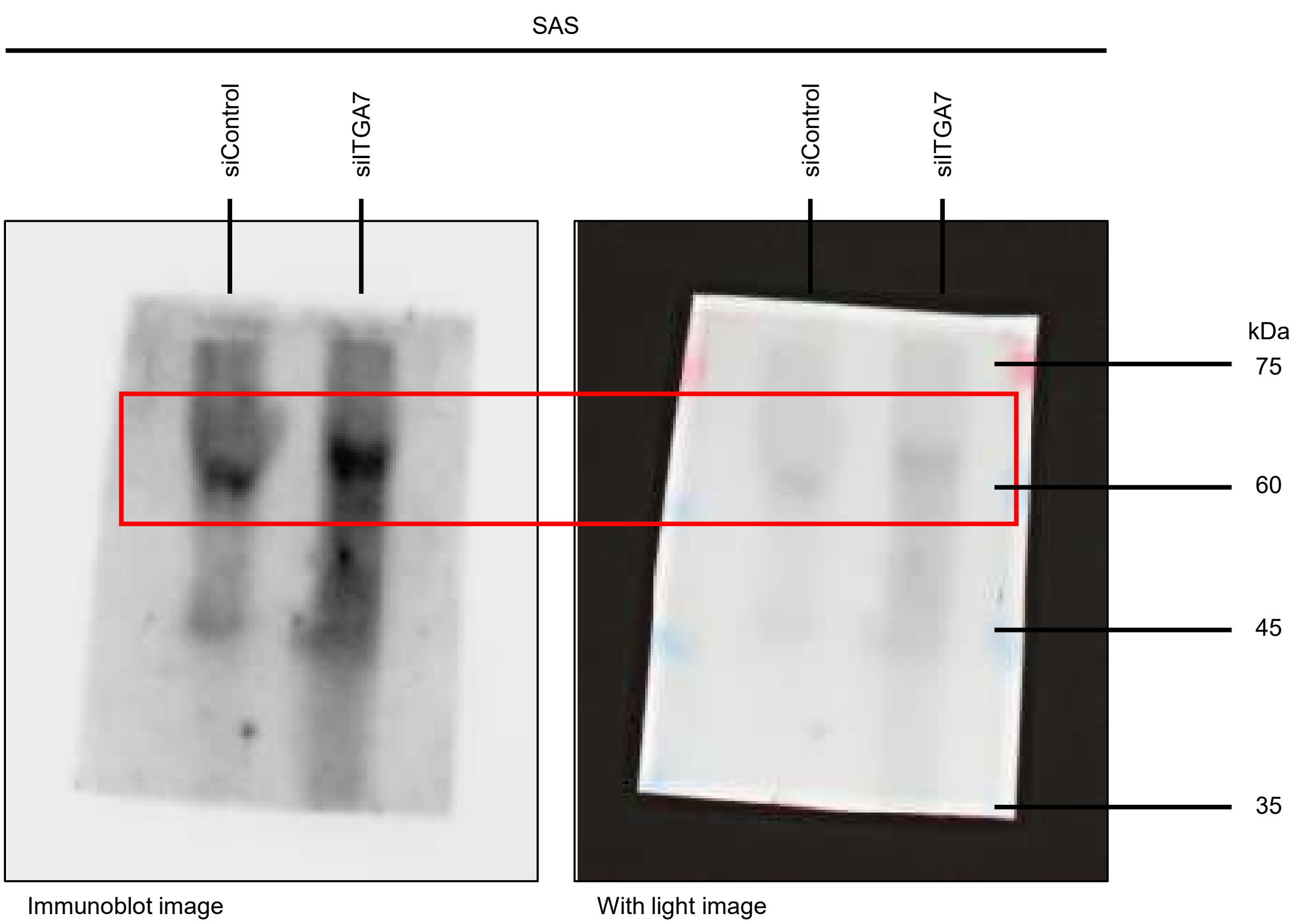

Area cropped on the article figure

CREB

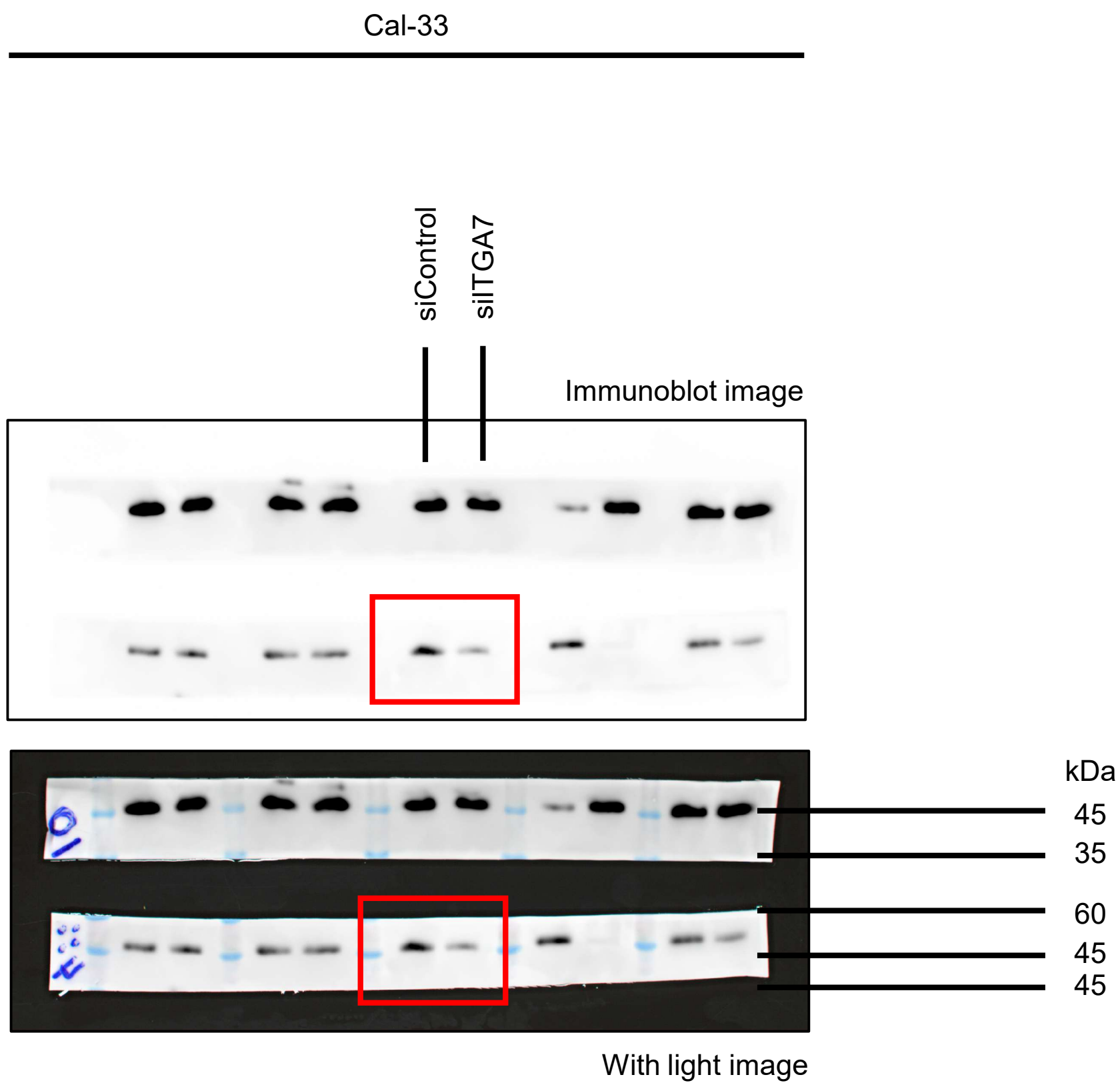

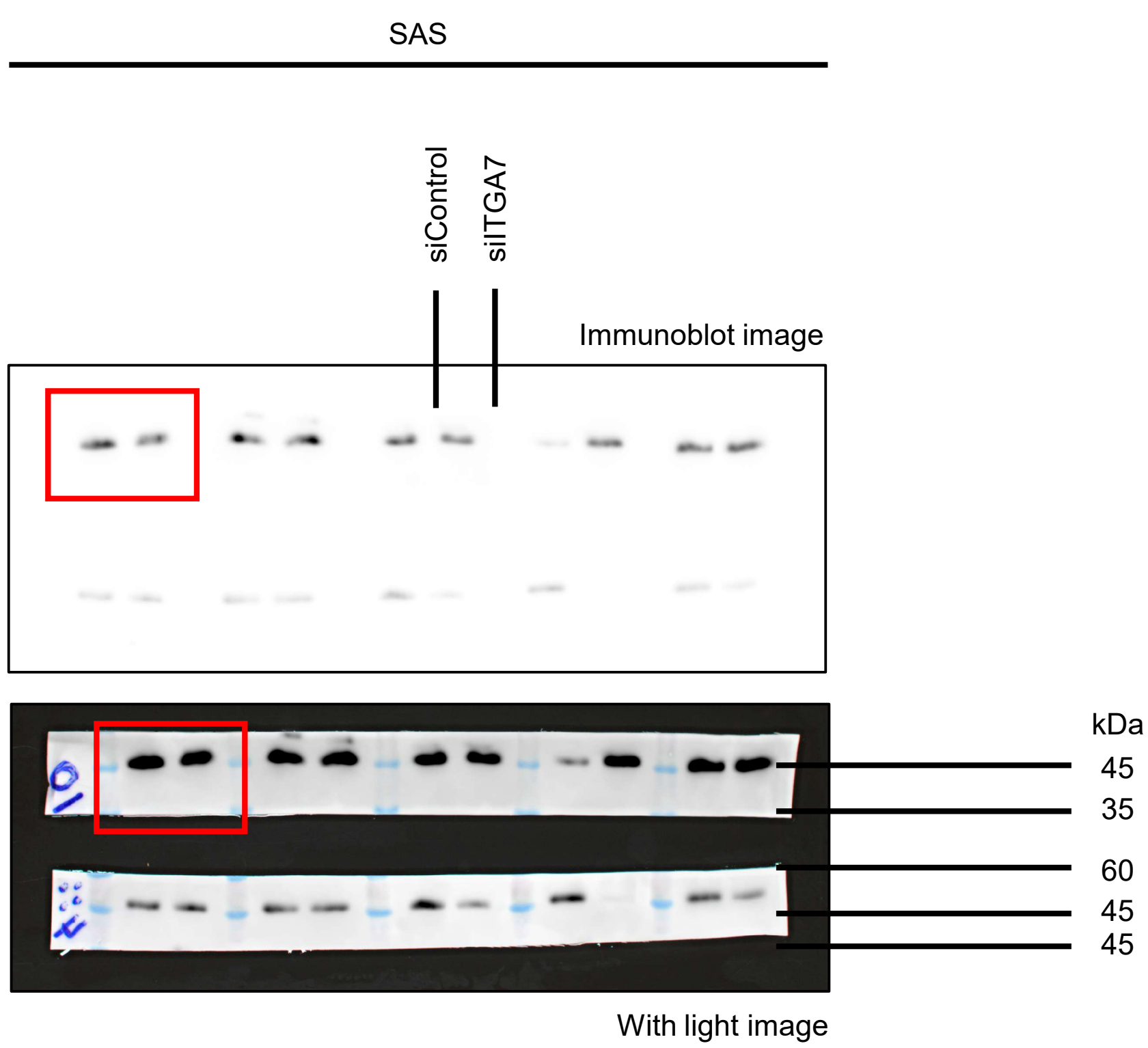

Area cropped on the article figure

p-c-JUN (Ser63)

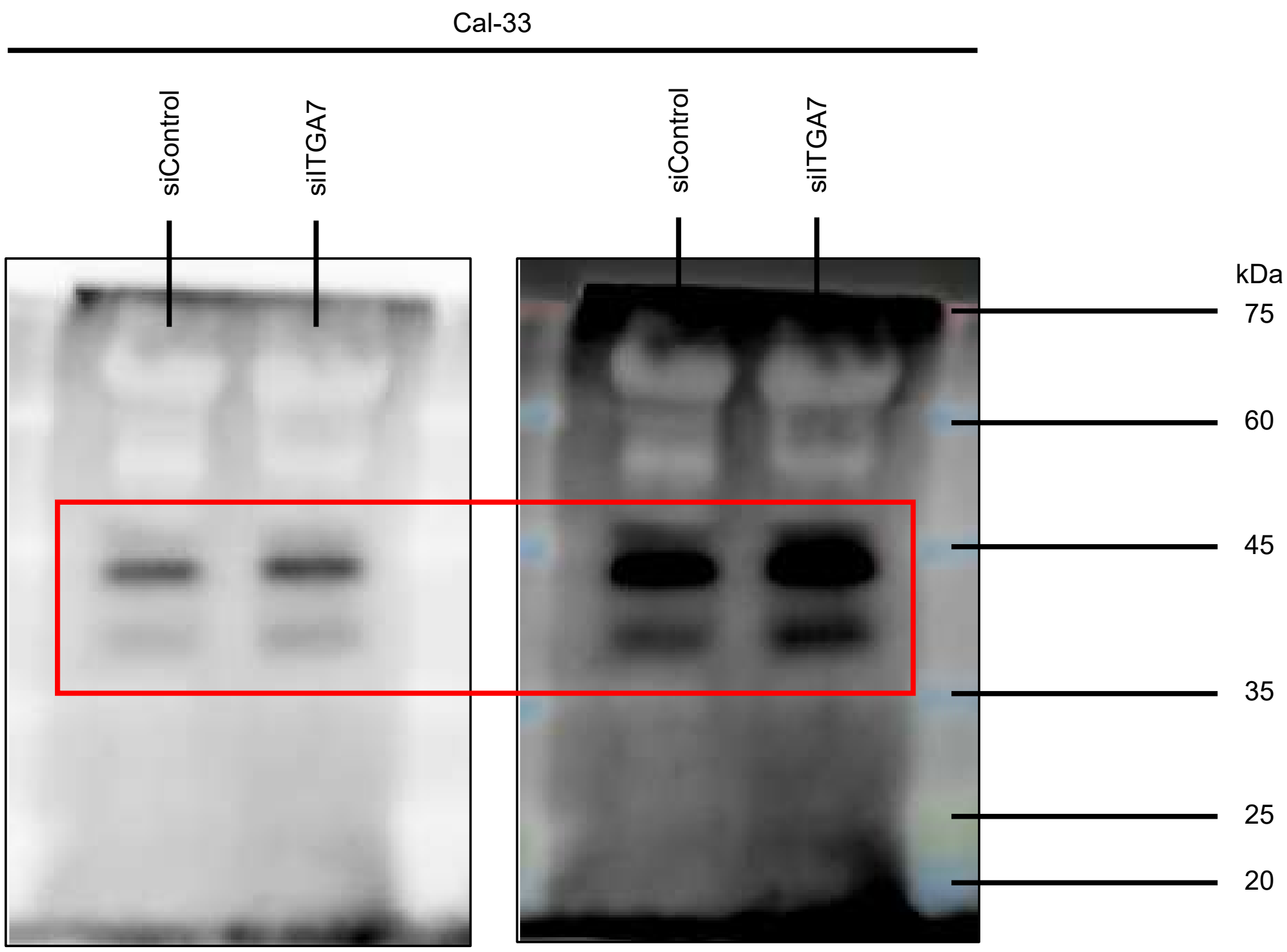

Immunoblot image

With light image

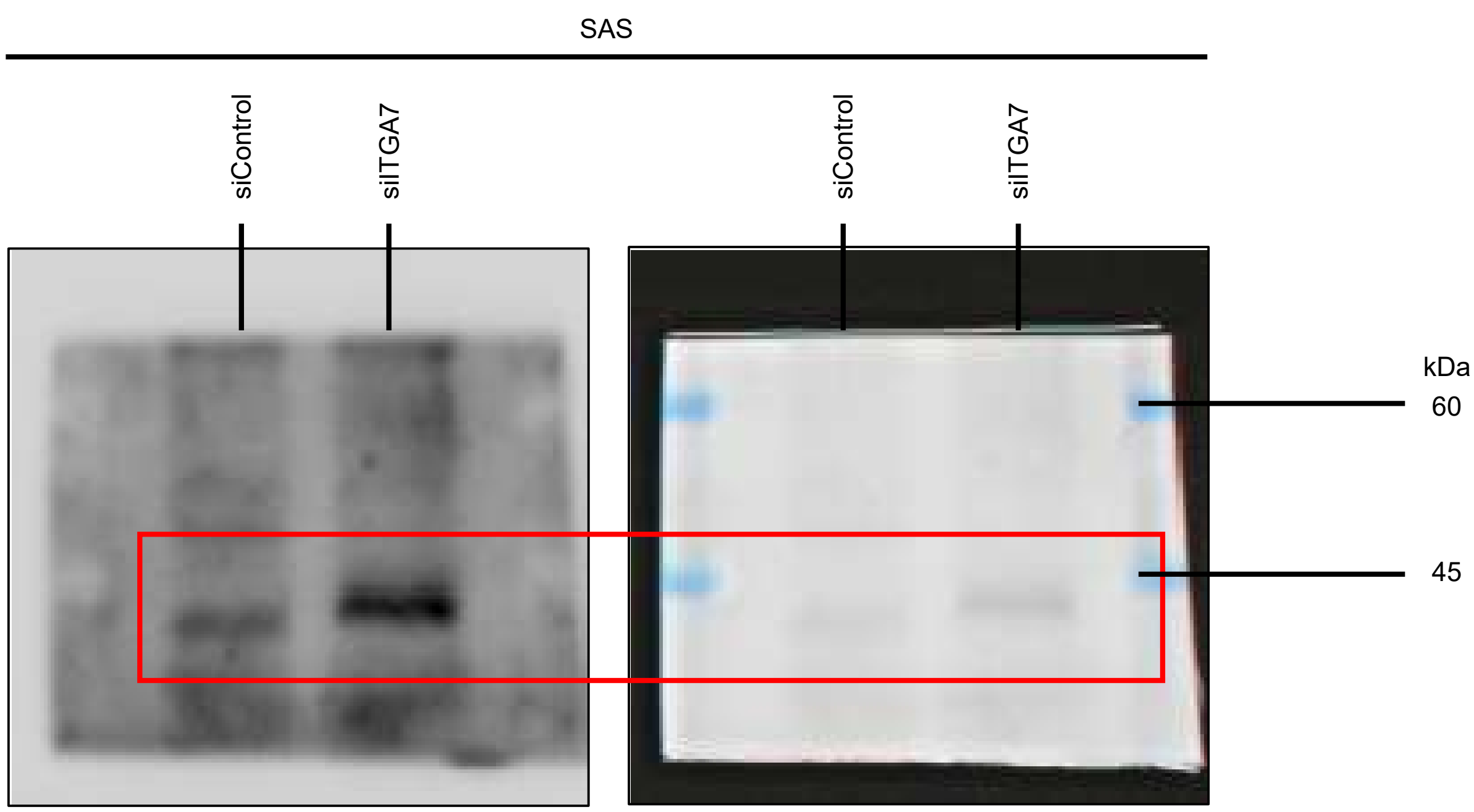

Immunoblot image

With light image

Area cropped on the article figure

c-Myc

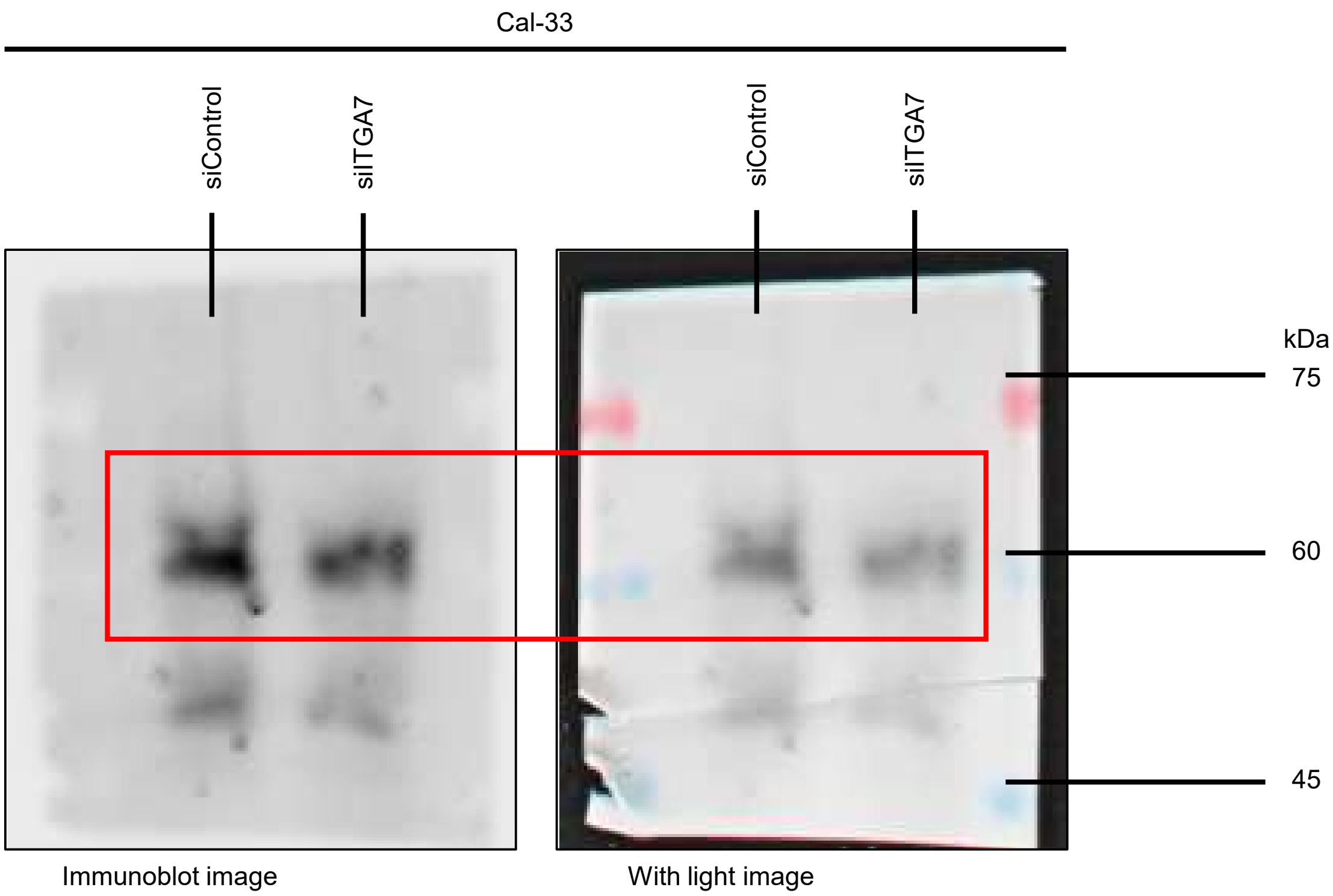

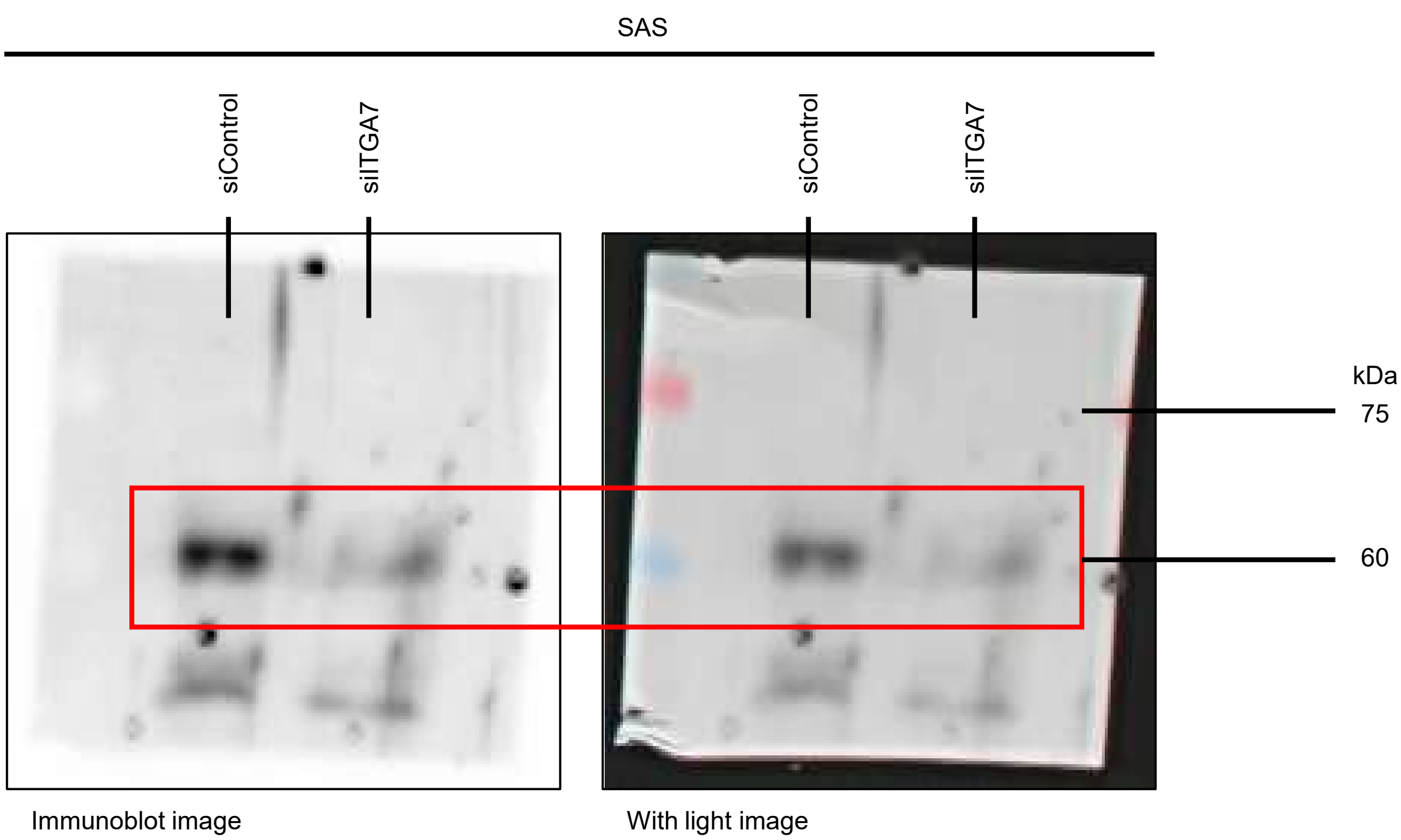

Area cropped on the article figure

NF-κB p65

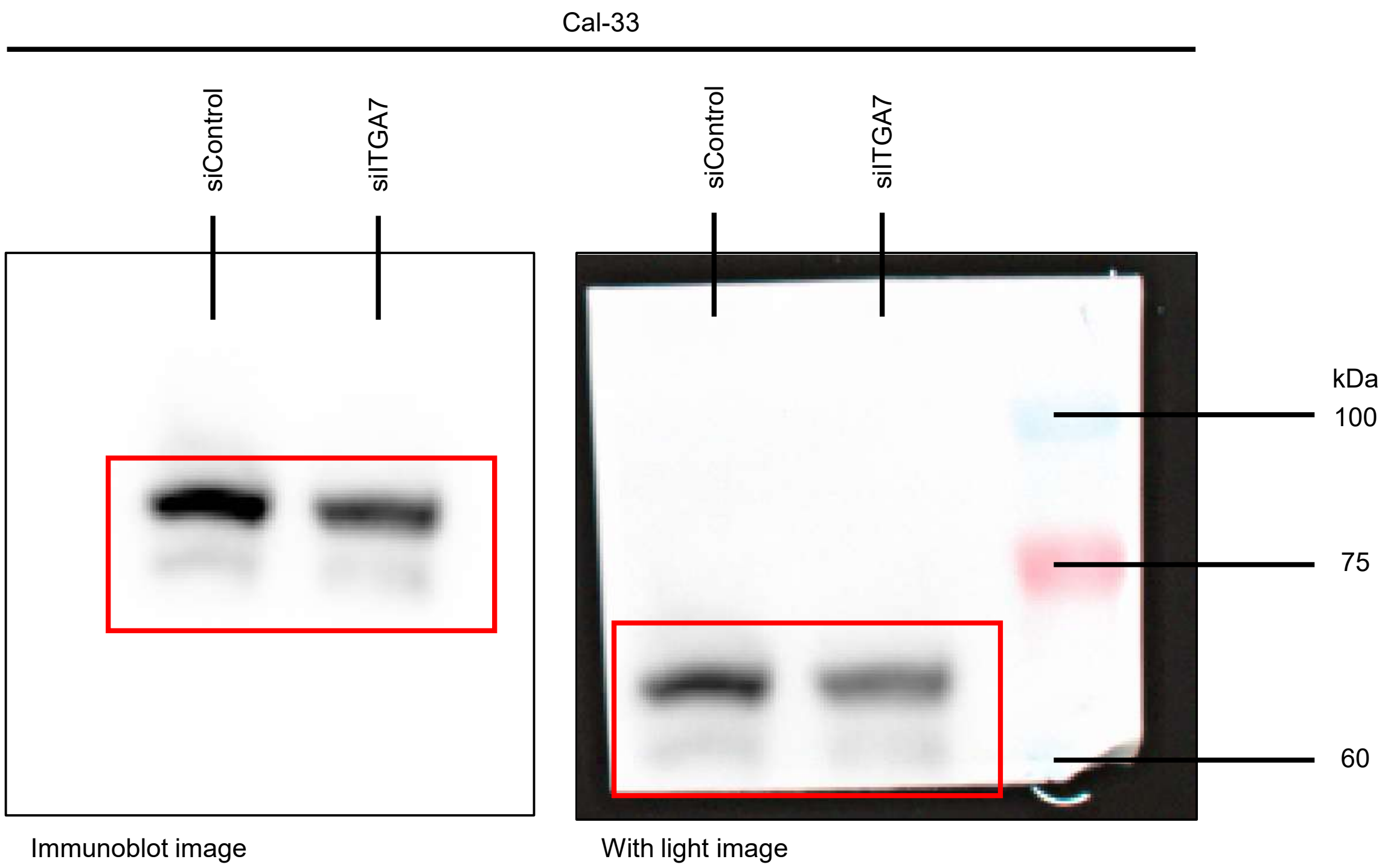

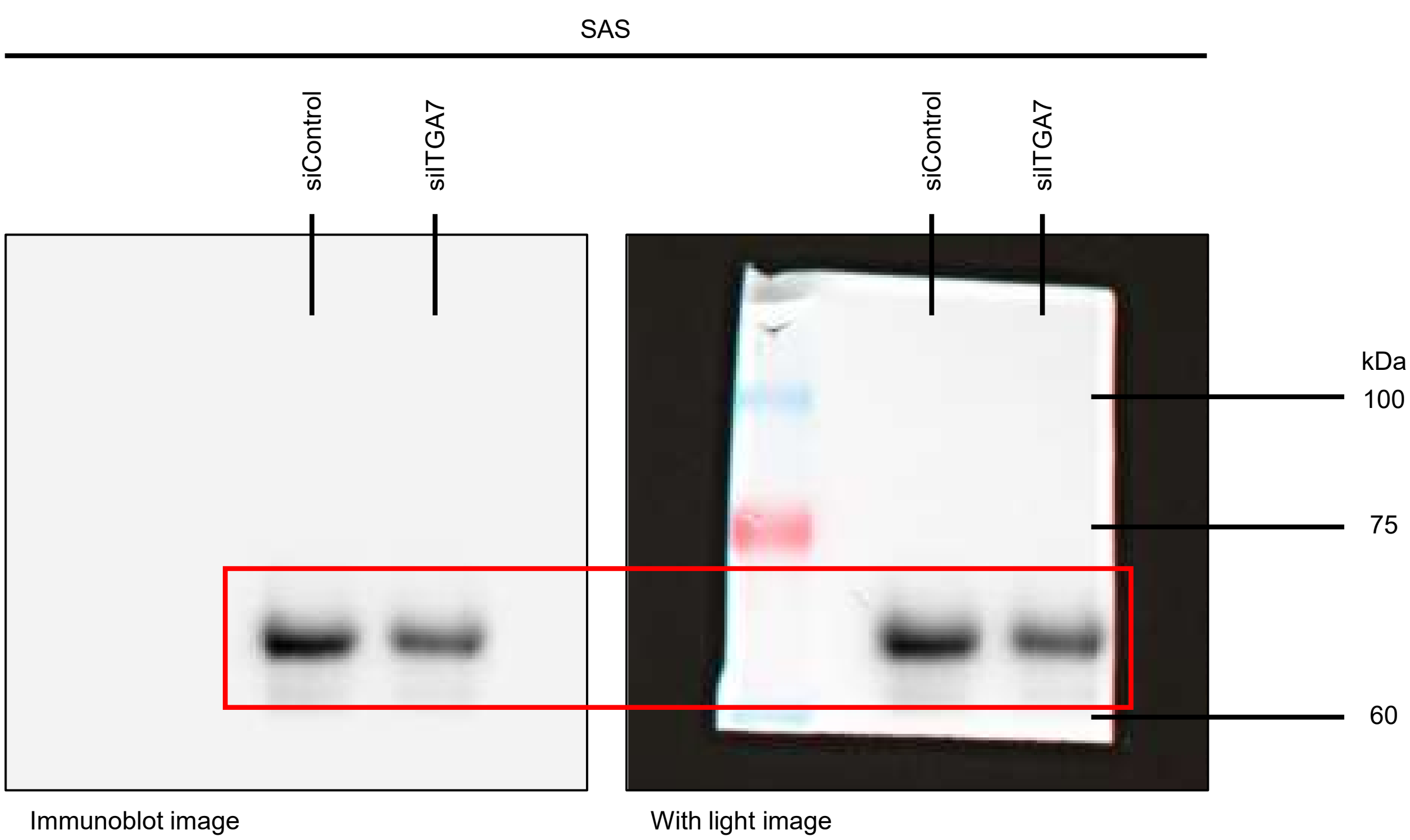

Area cropped on the article figure

p-NF-κB p65 (Ser468)

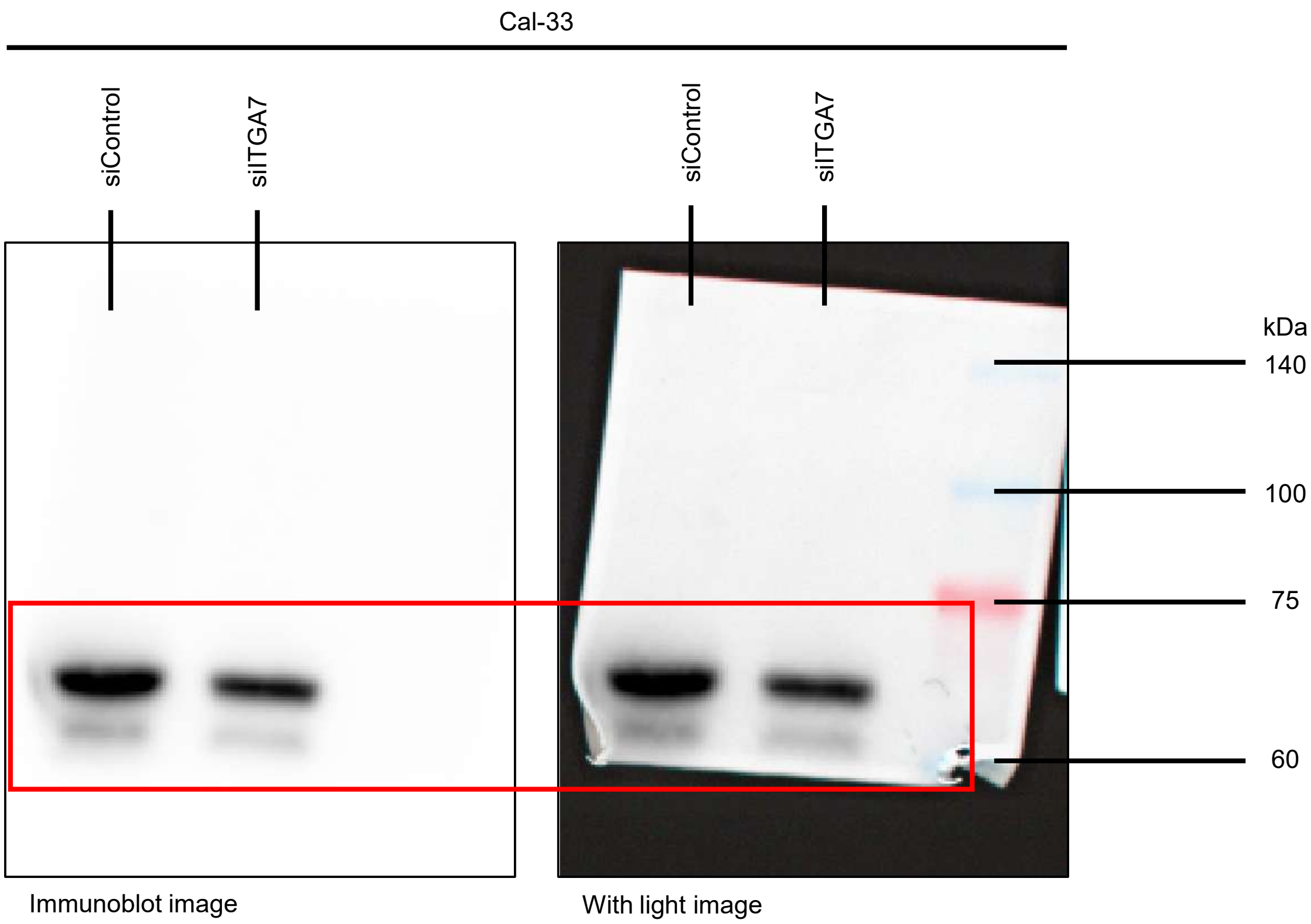

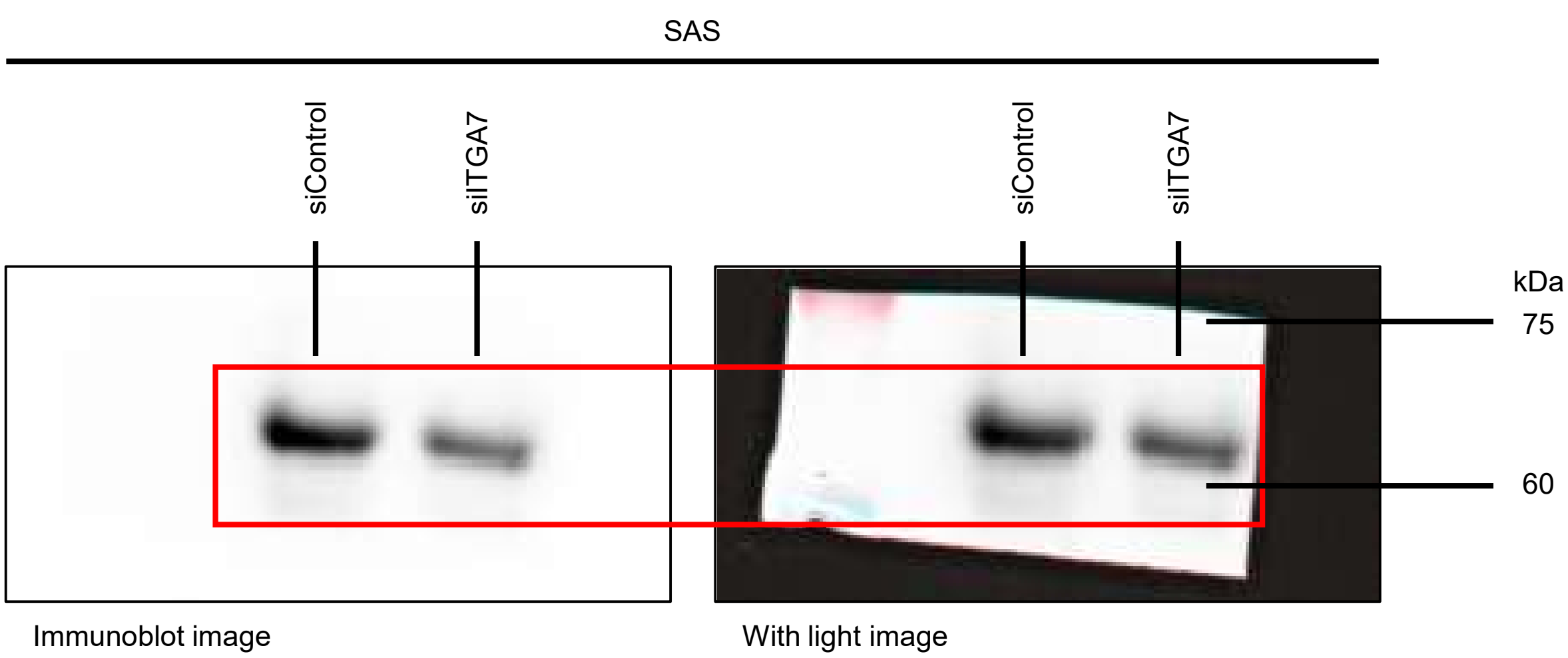

Area cropped on the article figure

Bcl-2

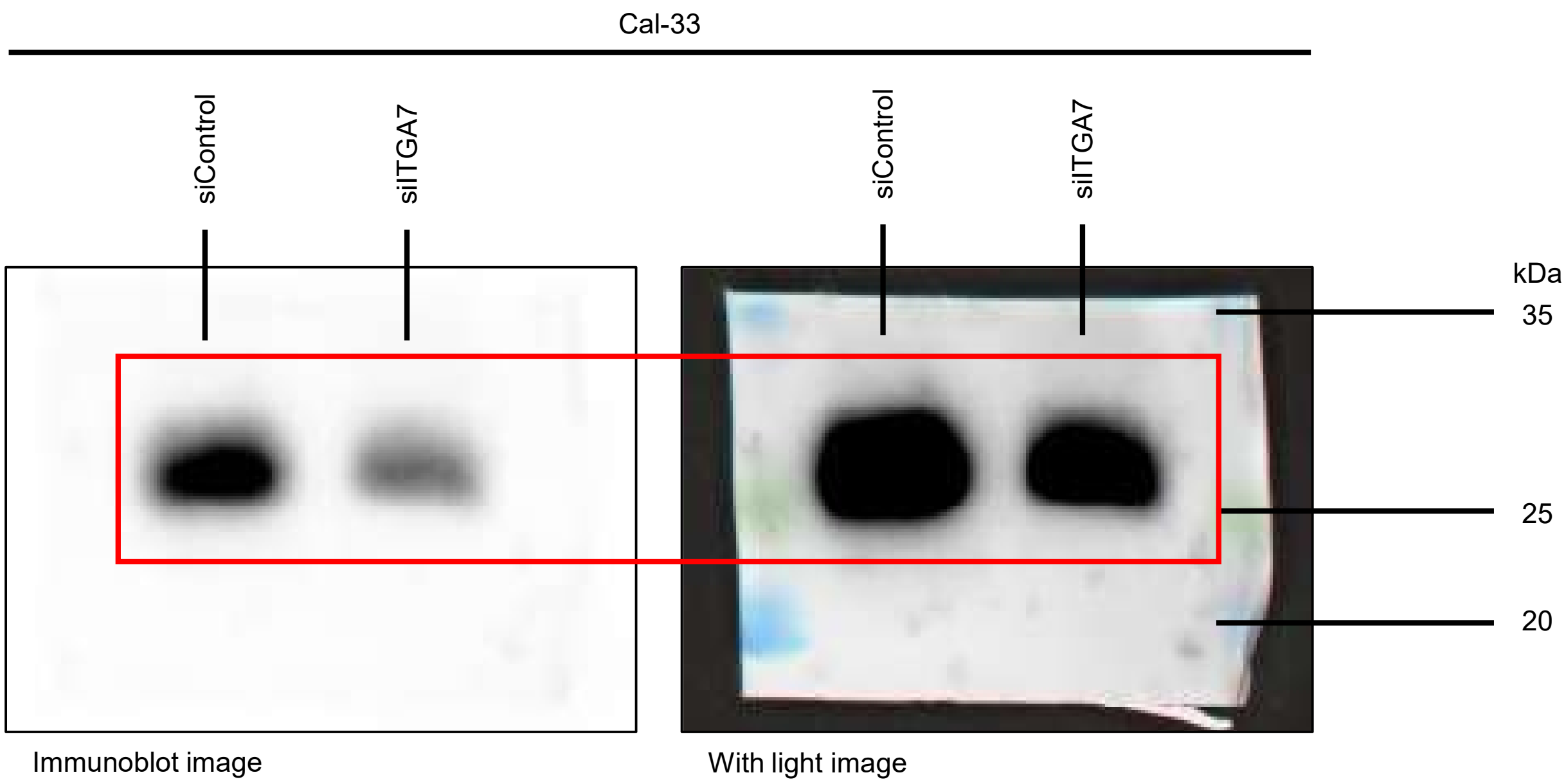

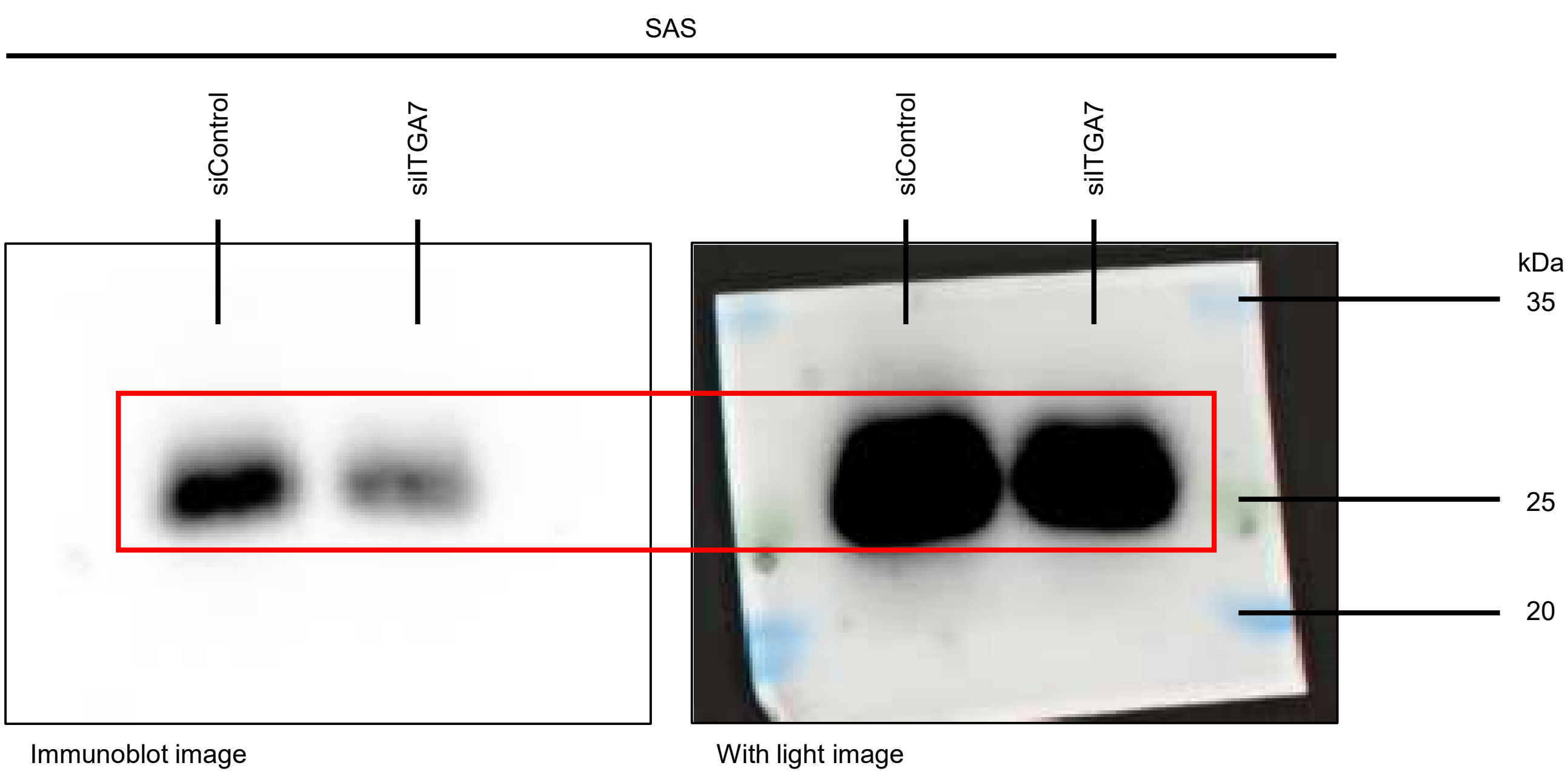

Area cropped on the article figure

14-3-3 (pan)

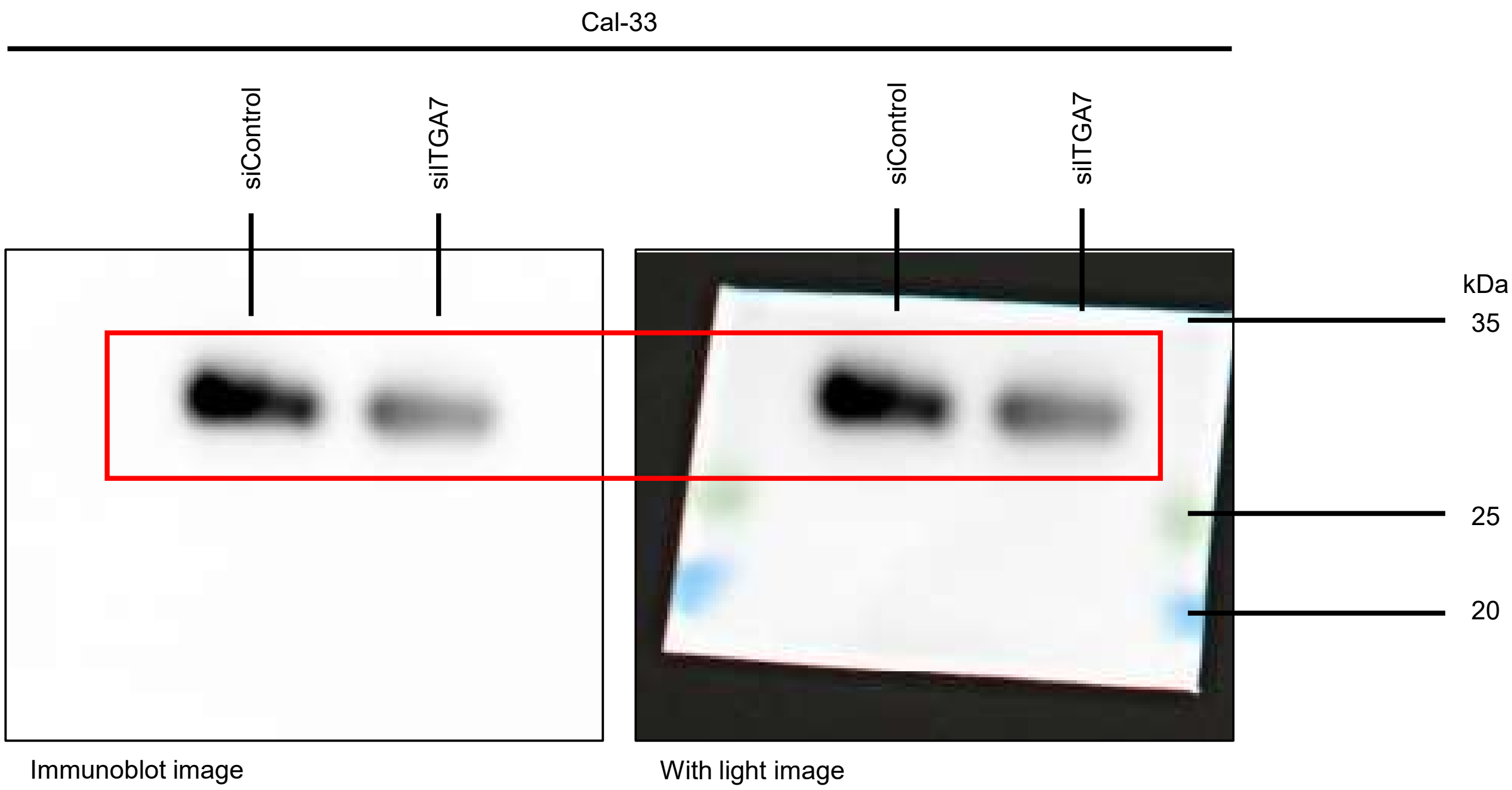

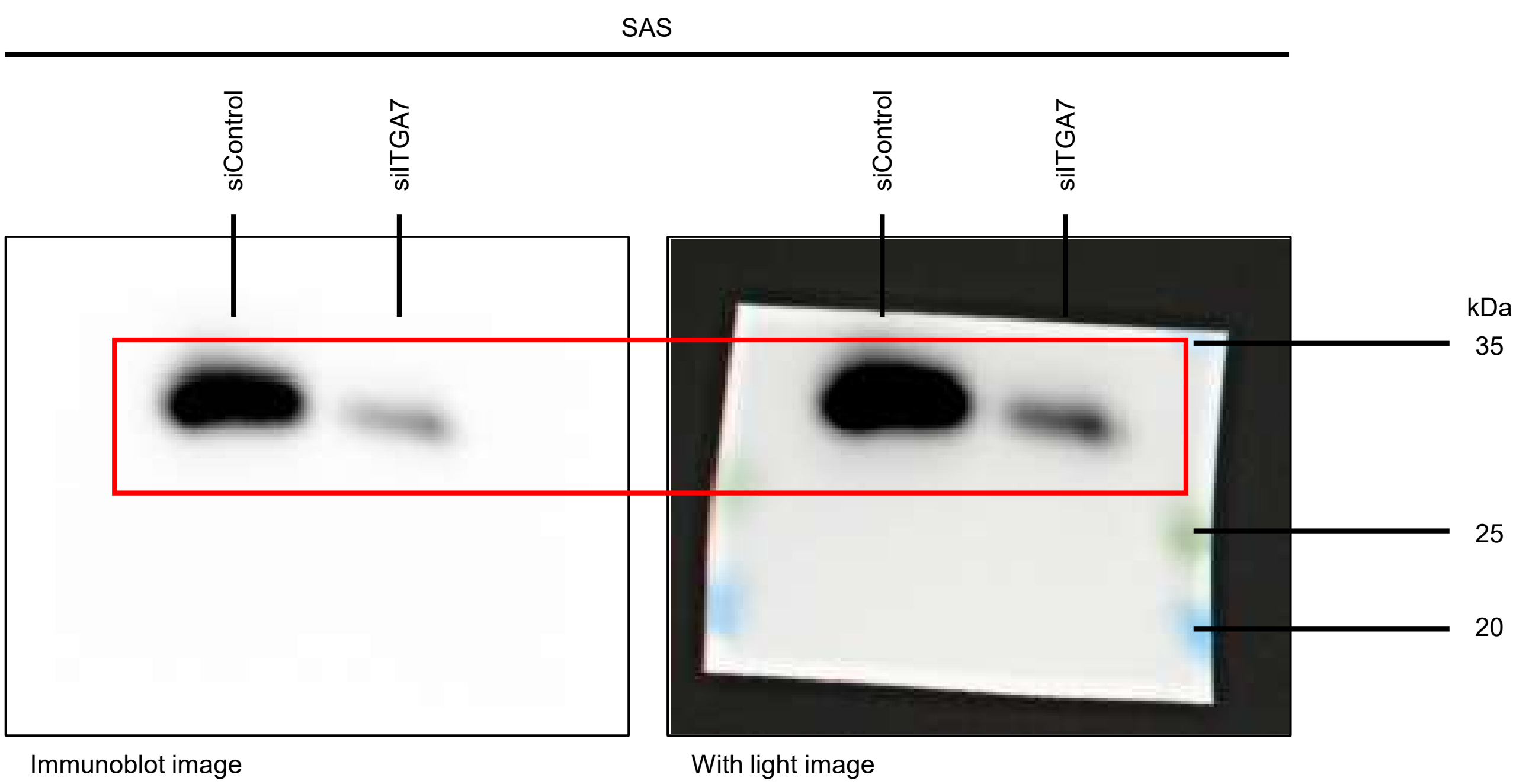

Area cropped on the article figure

14-3-3 ζ

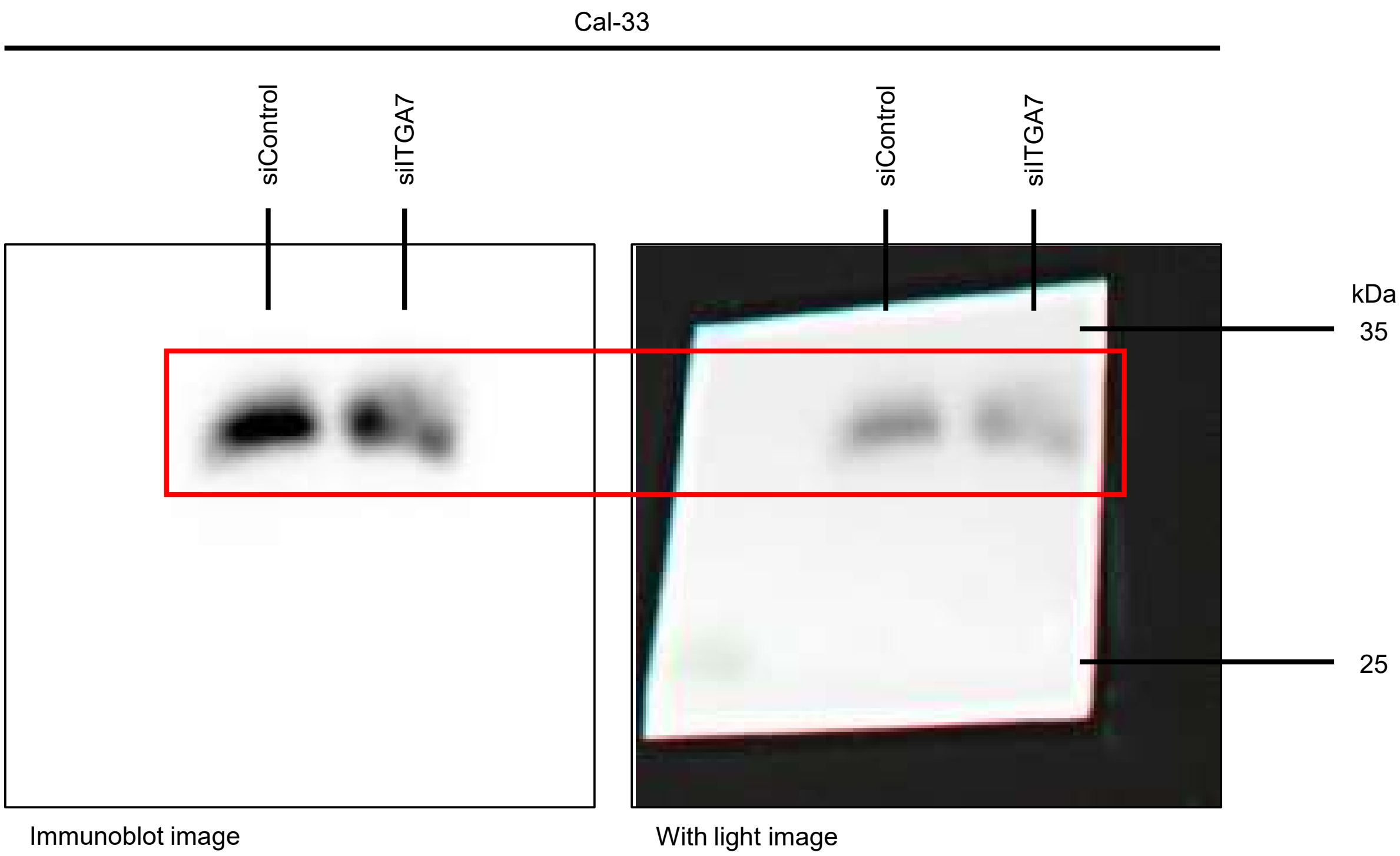

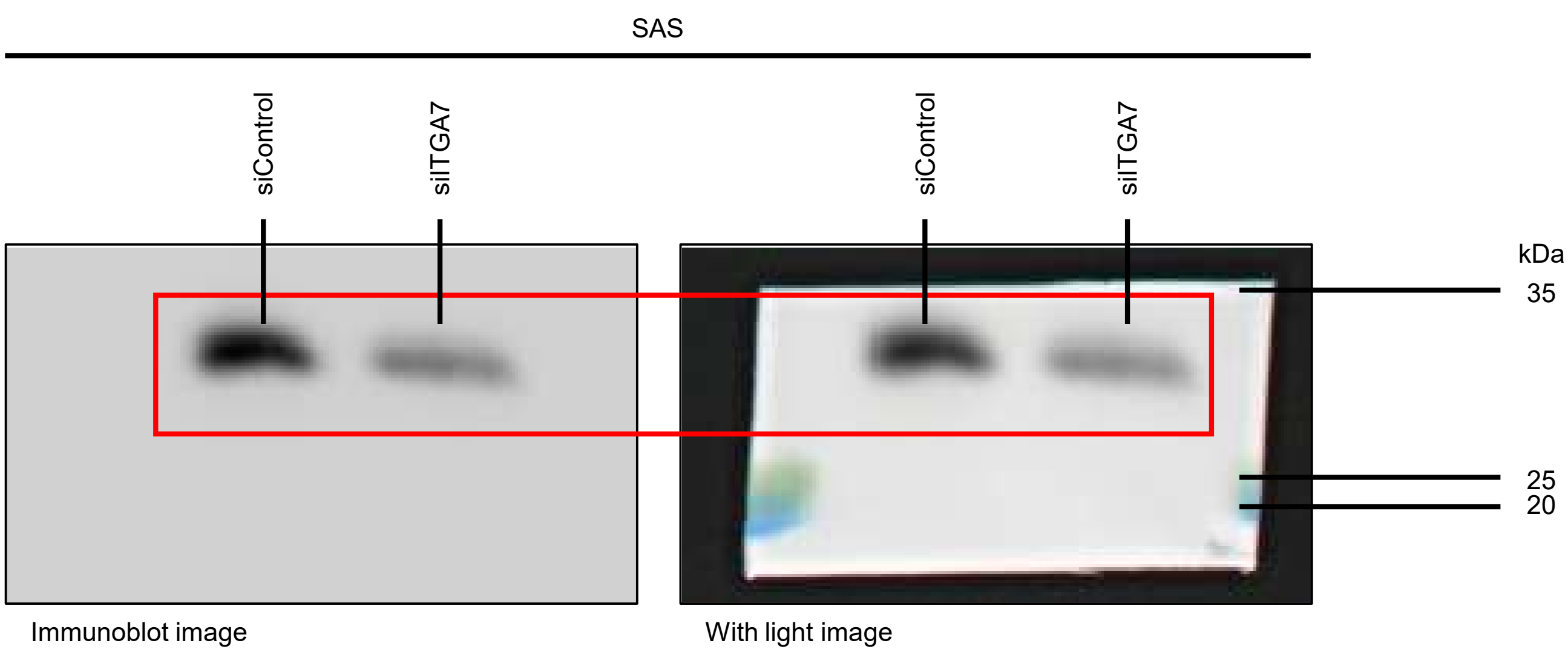

Area cropped on the article figure

14-3-3 ζ/Δ

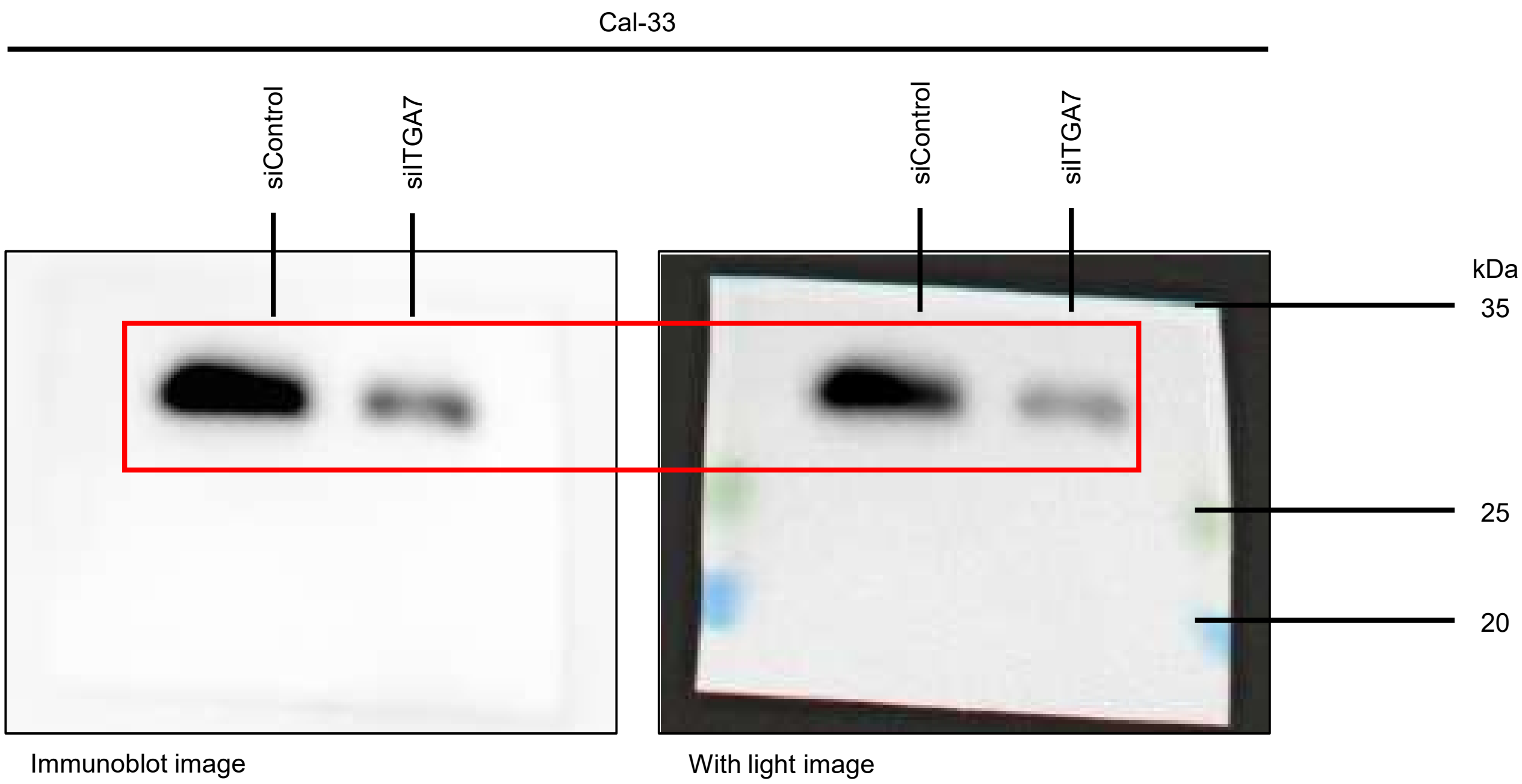

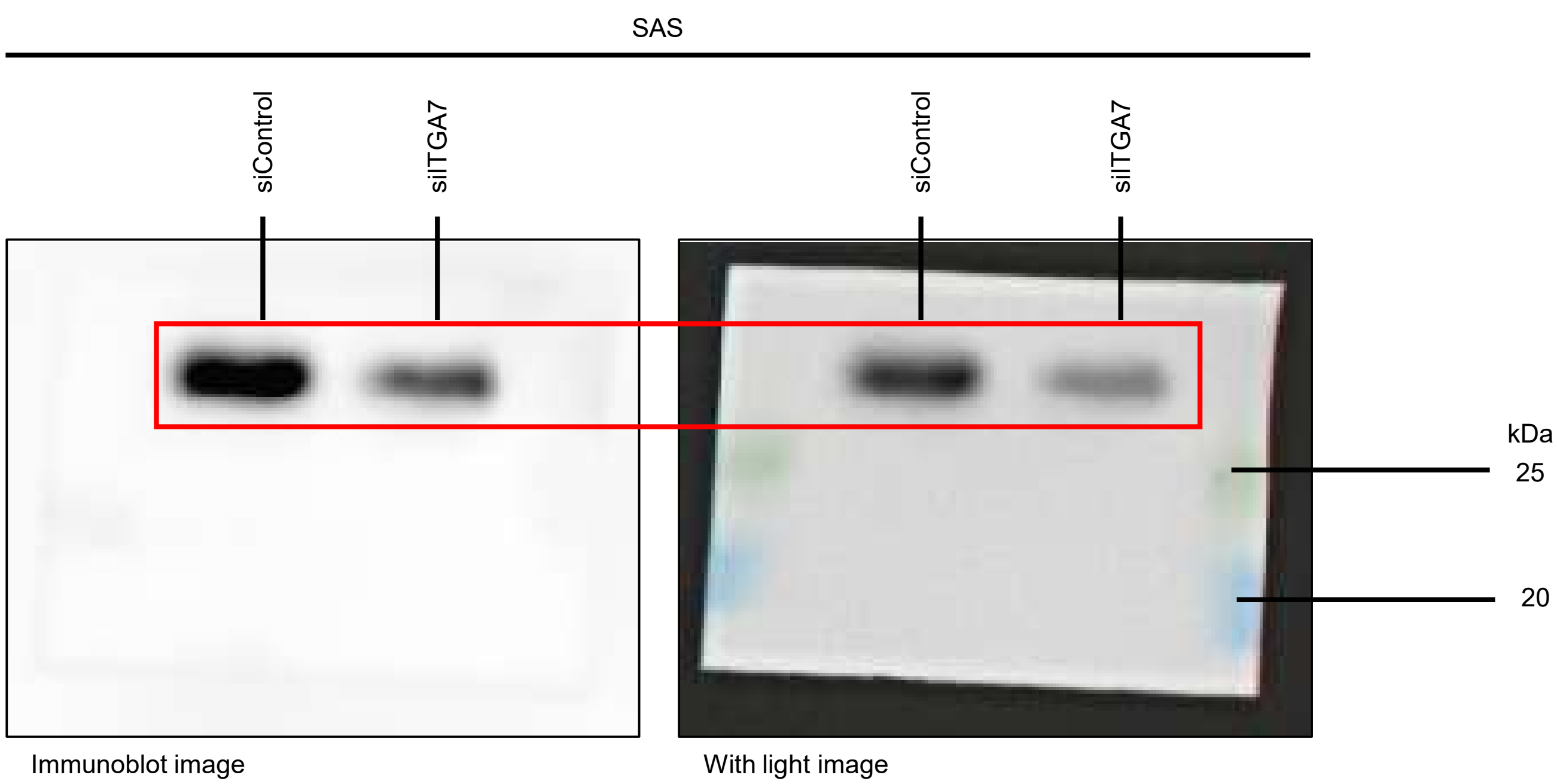

Area cropped on the article figure

14-3-3 θ

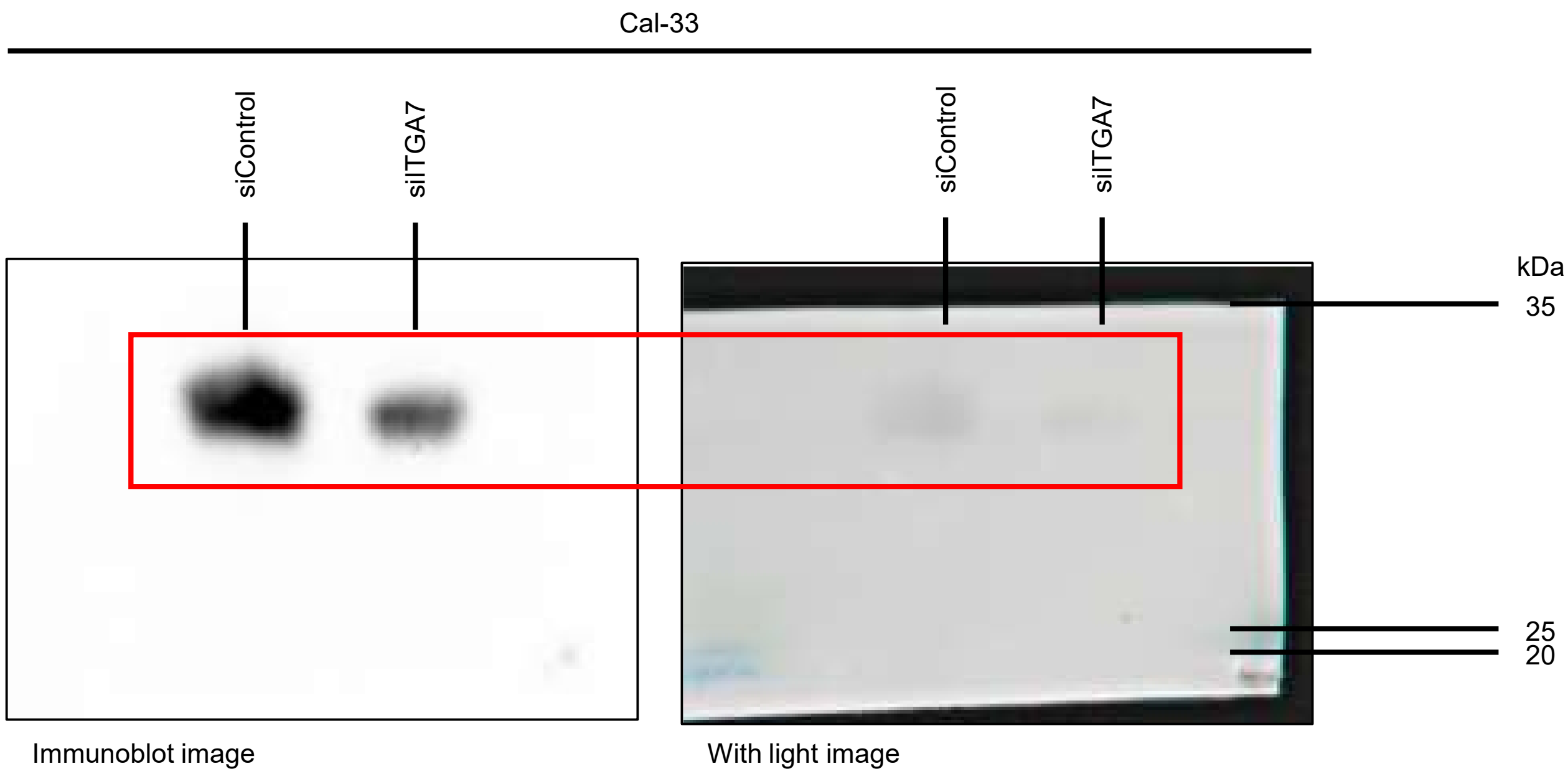

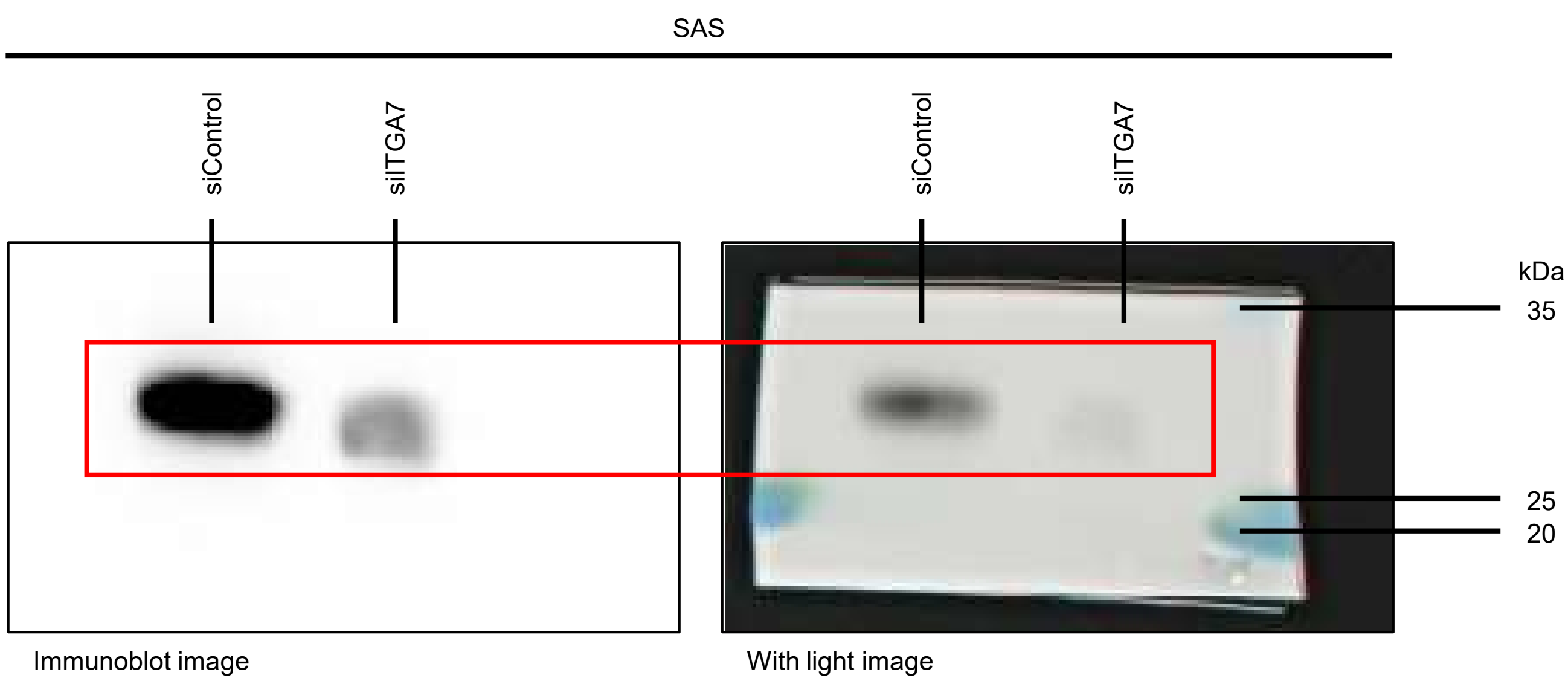

Area cropped on the article figure

p-14-3-3 θ (Ser232)

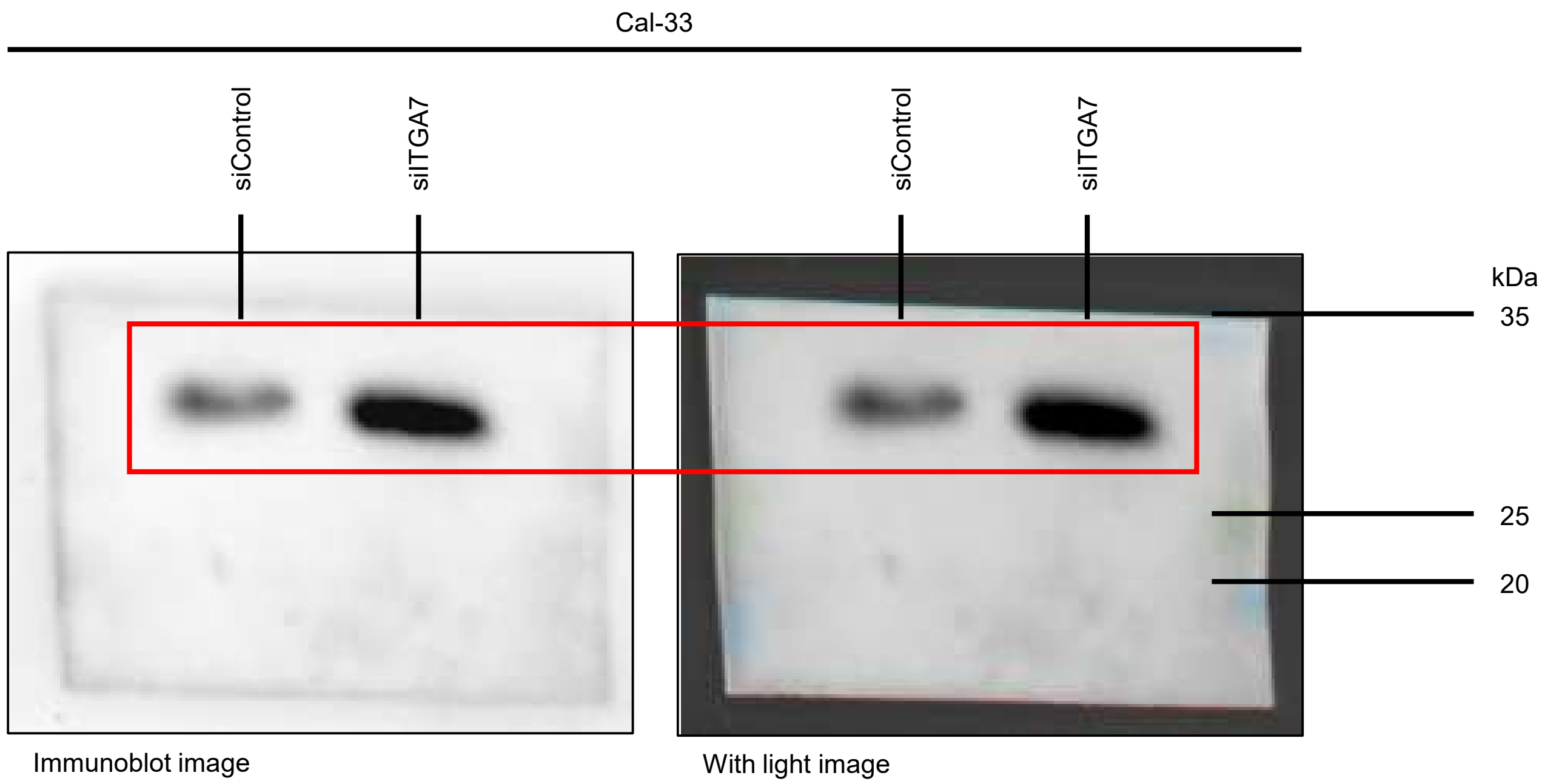

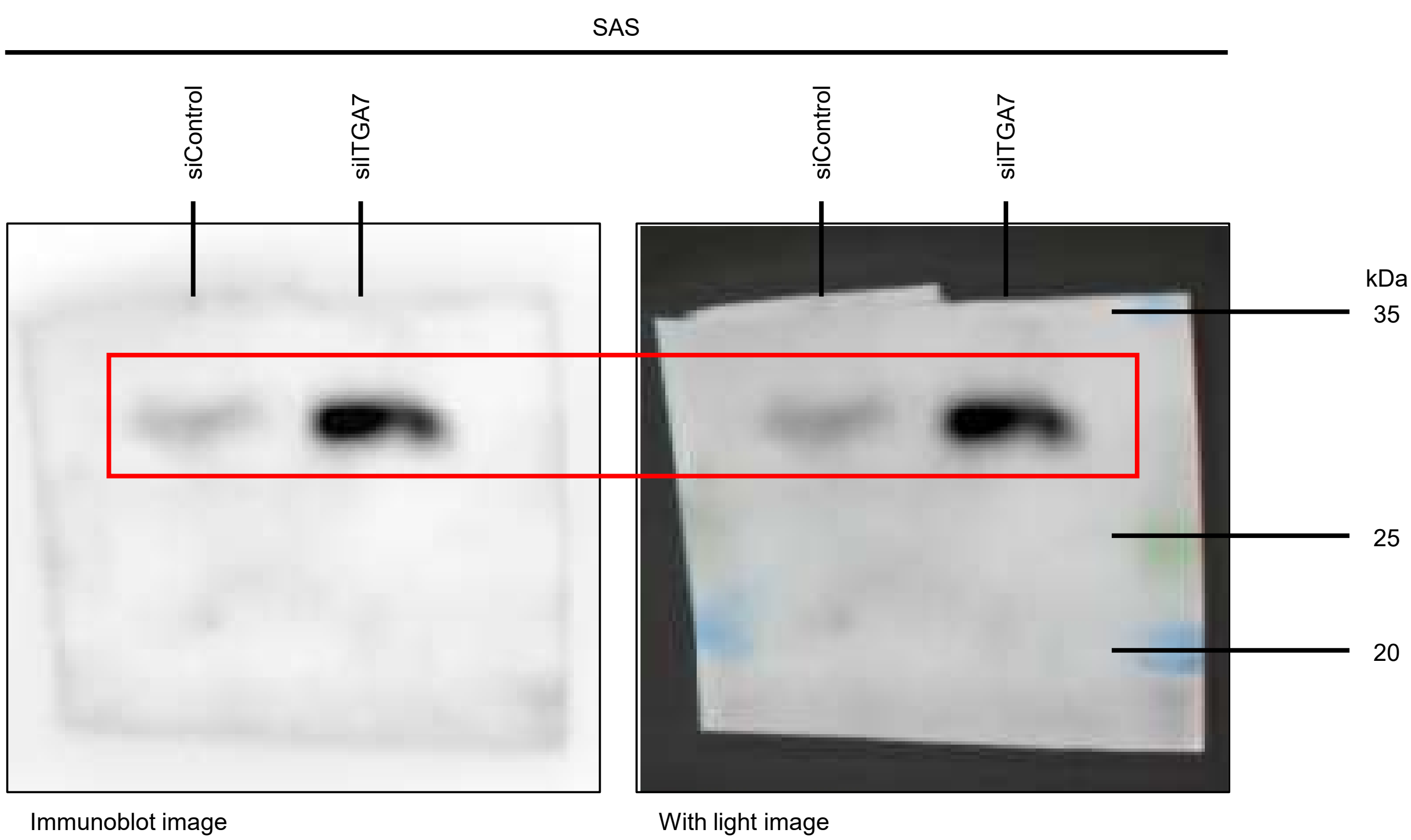

Area cropped on the article figure

H-Ras

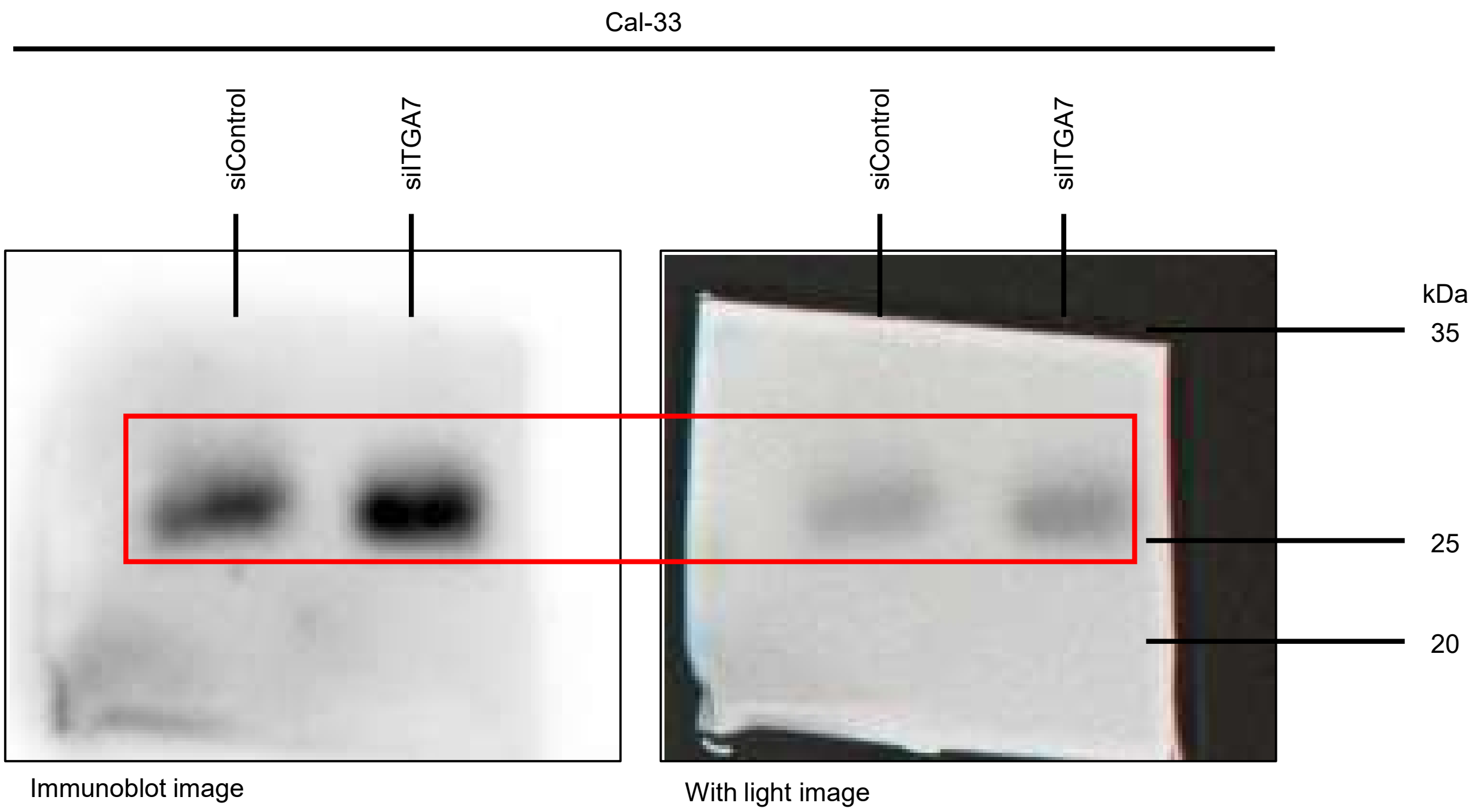

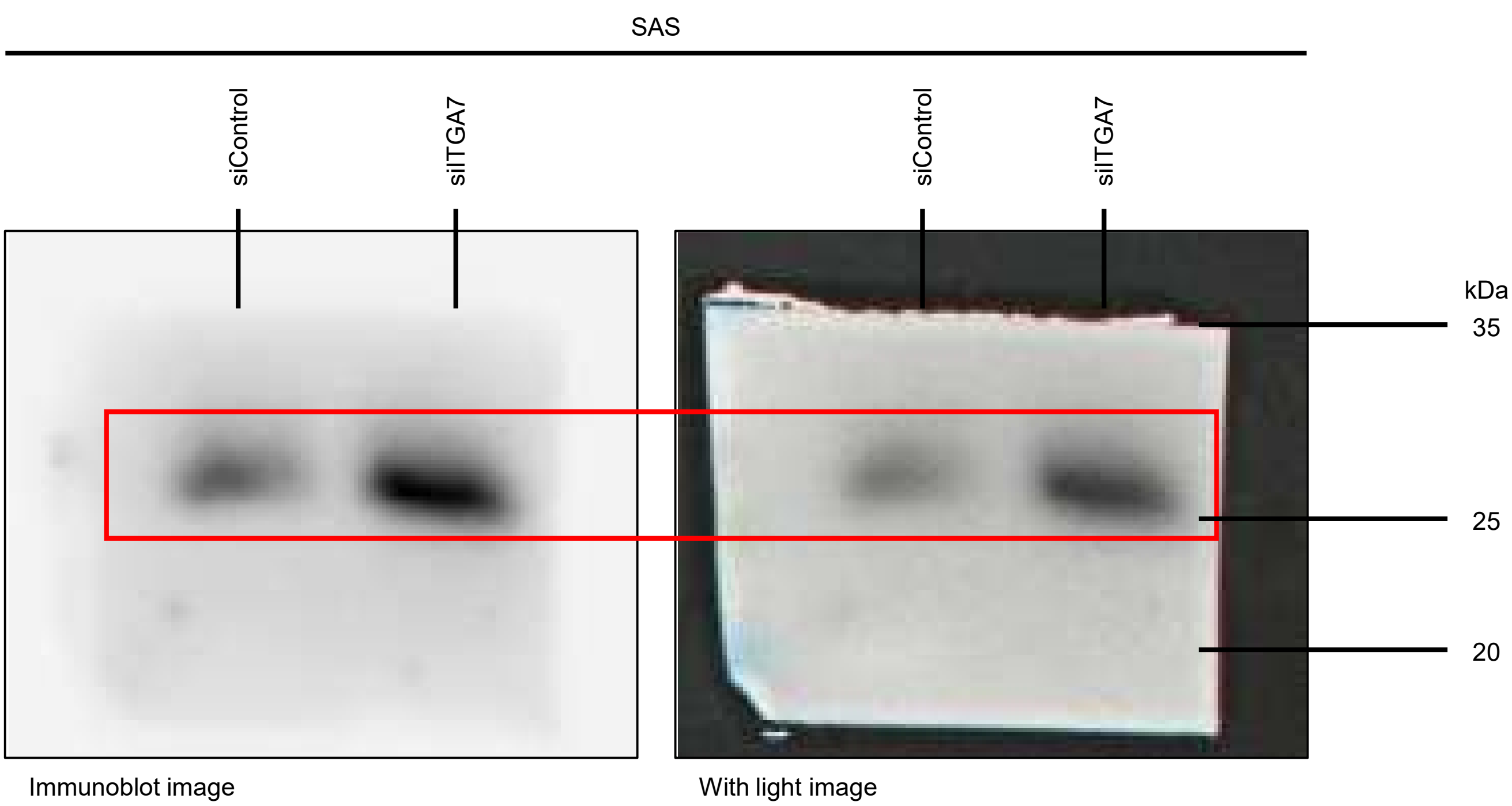

Area cropped on the article figure

β-Actin

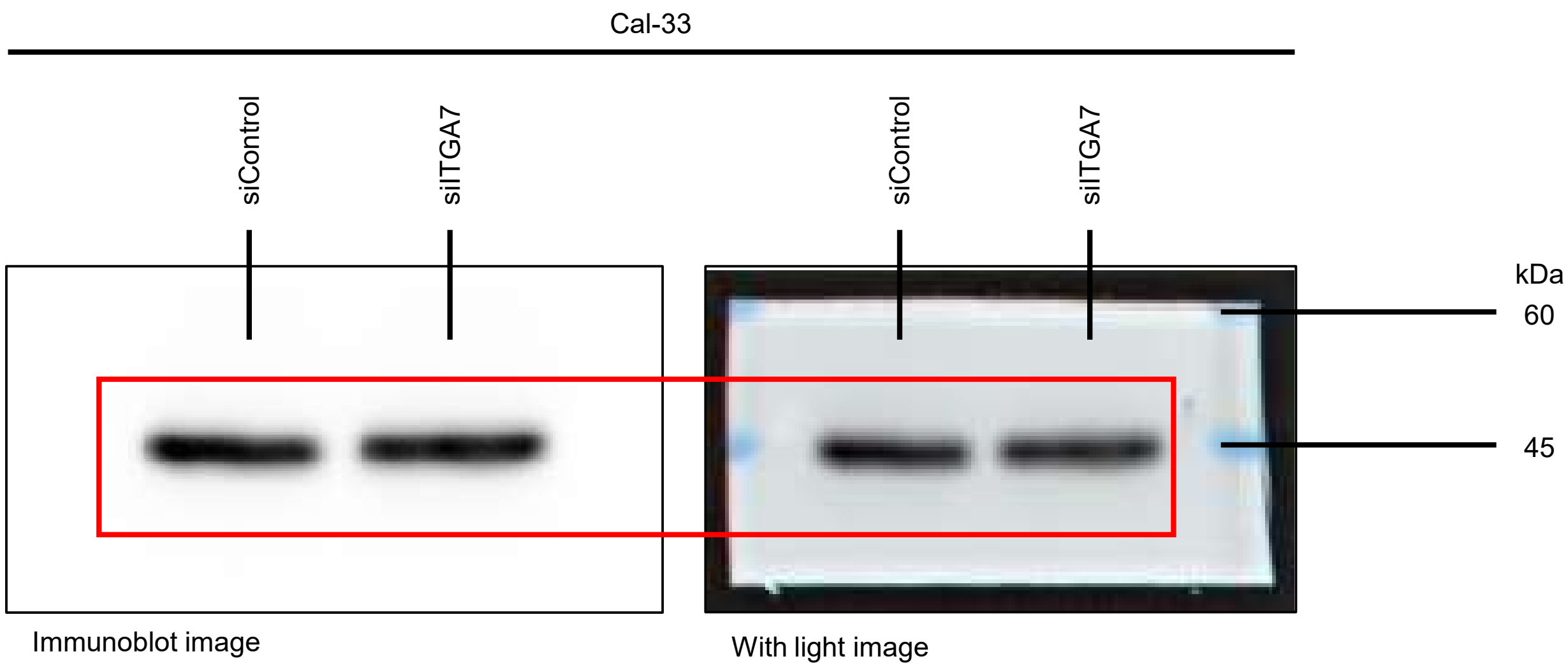
Cal-33
siControl
siITGA7
siControl
siITGA7
kDa
60
45
Immunoblot image
With light image

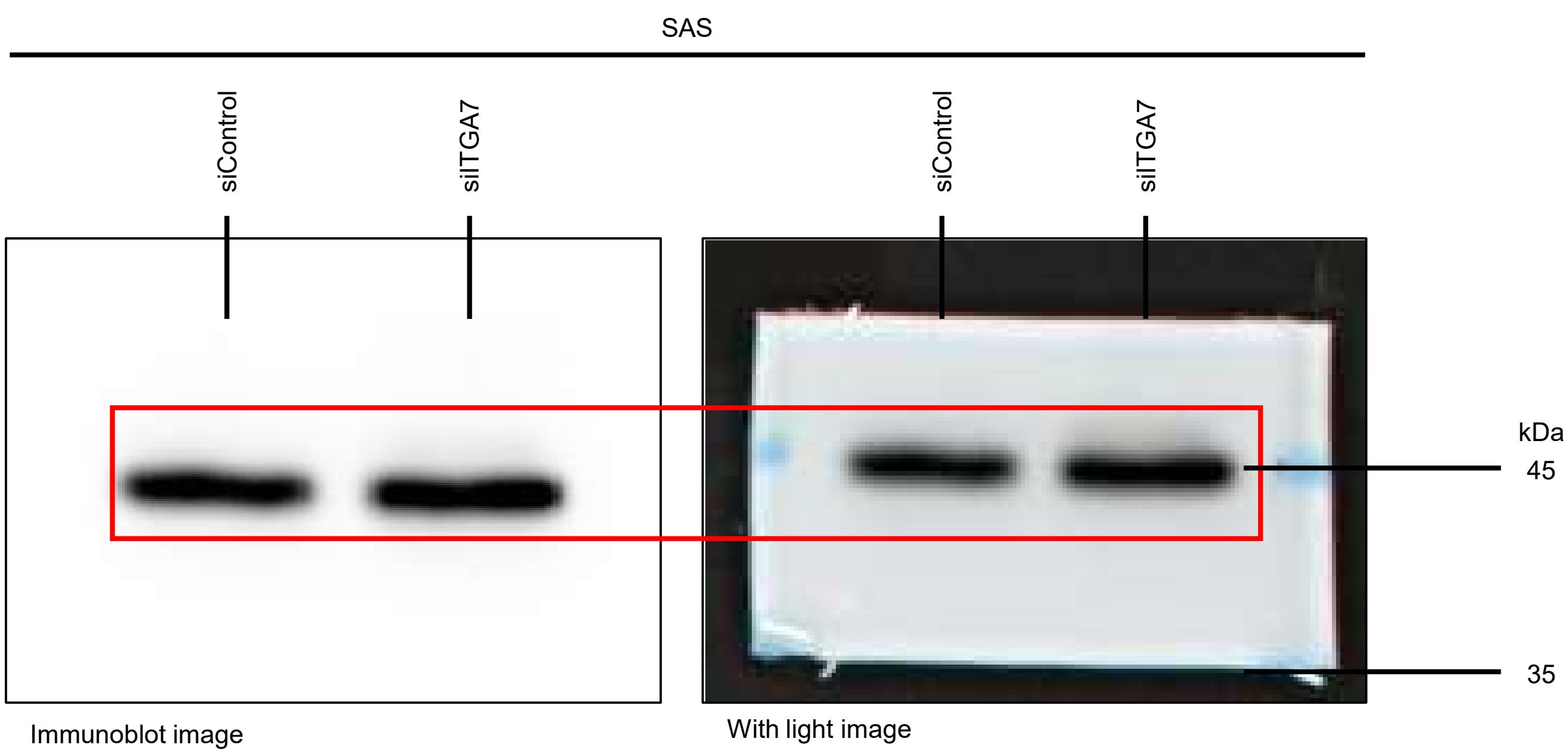
SAS
siControl
siITGA7
siControl
siITGA7
kDa
45
35
Immunoblot image
With light image

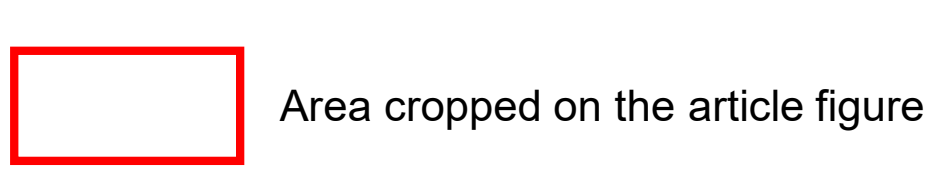
Area cropped on the article figure
