# Supplementary material for: α7 integrin co-regulates chromatin accessibility and gene expression in HPV-negative head and neck cancer
Source: Signal Transduct Target Ther. 2026 Oct 7;11:429. doi: 10.1038/s41392-026-02995-9 (PMC13642342; doi:10.1038/s41392-026-02995-9)
Supplement: Supplementary file 2 [file 41392_2026_2995_MOESM1_ESM.docx]

Supplementary Materials for

α7 integrin co-regulates chromatin accessibility and gene expression in HPV-negative head and neck cancer

Olegs Borodins ^1^, Anne Vehlow ^1^, Katrin Sameith ^2^, Andreas Dahl ^2^, Jennifer M. Spangle ^3^, Daniela E. Aust ^4^, Cornelia Brunner ^5^, Outi Monni ^6,7,8^, Inga Lange ^1,9^, Jovan Mircetic ^10^, Elisa Thomas ^1,10,11^, Nils Cordes ^1,9,10,11,^*

Correspondence to: Nils.Cordes@OncoRay.de

This PDF file includes:

Materials and Methods

Supplementary Figures 1 to 14

Other Supplementary Materials for this manuscript include the following:

Data S1 to S8

Supplementary Videos S1–S8

Tables S1

# Material and Methods

## **Public data collection and preprocessing**

Data were sourced from publicly available databases and repositories. RNA sequencing (RNA-seq) data for Head and Neck Squamous Cell Carcinoma (HNSCC) were obtained from The Cancer Genome Atlas (TCGA) (https://www.cancer.gov/tcga) and normalized to TPM + 1 values. Additional datasets were accessed from Gene Expression Omnibus (GEO) ^1^ with accession numbers GSE127072, GSE151420, GSE152088, GSE186947, GSE196688, and GSE181919. Supporting data were retrieved from GEPIA2 ^2^ and OncoDB ^3^ platforms, and ChIP-seq data were obtained from ChIP-Atlas ^4^. Cell line information was referenced from Cellosaurus (https://www.cellosaurus.org). Bioinformatic analyses were supported using resources from the bioinformatics portal at Henan University (https://bioinfo.henu.edu.cn) and Broad Institute’s Morpheus software (https://software.broadinstitute.org/morpheus/). Public data were accessed via R TCGAbiolinks ^5^ and GEOquery ^6^ packages.

## **Network analysis and reconstruction**

Network analysis was performed using Cytoscape’s STRING app ^7,8^ to integrate and reconstruct networks of genes of interest. A minimum confidence score of 75% was applied across all analyses. For interaction data reconstruction, BioGRID, NCBI, GeneMANIA, Reactome ^9^ and Kyoto Encyclopedia of Genes and Genomes (KEGG) ^10^ were used.

## **Methylation data analysis**

Methylation data for HNSCC were obtained from TCGA, and differential methylation analysis was performed to identify significantly altered methylation patterns between HNSCC and control groups. The analysis utilized several R packages: methylGSA was applied for gene set enrichment analysis; sesame and sesameData were used for processing and quality control of Illumina DNA methylation array data, including preprocessing raw intensities and extracting β-values for fold-change calculation; TCGAbiolinks was used for efficient retrieval, querying, and preprocessing of TCGA methylation data for both HNSCC and control samples.

## **Gene ontology and functional analysis**

Genes and functions were annotated using Gene Ontology (GO) terms via ShinyGO ^11^, G:Profiler ^12^, ClueGO ^13^, and Ingenuity Pathway Analysis (IPA) ^14^, with additional insights from KEGG ^10^, Wikipathways ^15^, Reactome ^9^, and Gene Set Enrichment Analysis (GSEA) ^16^ (*p* < 0.05; FDR < 0.05). Pathways affected by ITGA7 knockdown were identified by ShinyGO, highlighting enriched processes and systemic impacts. Data from multiple databases were integrated with ClueGO to reveal shared biological functions and pathway convergence. Transcriptomic, proteomic, and phosphoproteomic data were analyzed with Ingenuity Pathway Analysis (IPA; Qiagen), identifying key pathways associated with the affected genes data relative to control condition, upstream regulators (molecules whose known regulatory relationships with downstream genes are used to infer whether they may be responsible for the observed expression changes in a dataset), and master regulators (regulatory molecule that controls large gene networks or multiple downstream pathways and is predicted to drive major transcriptional programs in the dataset) influencing gene expression. Disease and function analyses linked changes to pathological conditions, providing a comprehensive view of the biological implications of ITGA7 knockdown and multi-omics data.

## **Patient survival analysis**

HNSCC patient data were obtained from TCGA, GSE65858, and GSE41613 datasets using the TCGAbiolinks and GEOquery R libraries. Clinical metadata, including survival time and event status, were extracted and processed. A survival object was created using the Surv function, with survival time and event status appropriately coded. Gene expression data for ITGA7 were analyzed to assess prognostic significance. Patients were categorized into high- and low-expression groups based on the top 10%, 35%, 50% and bottom 10%, 35%, 50% of integrin expression levels across the entire cohort. Kaplan-Meier survival curves were generated to compare survival outcomes between these groups. A log-rank test was performed using the survdiff function to evaluate statistical differences. A Cox proportional hazards model was fitted using the coxph function to associate integrins expression and survival outcomes. Hazard ratios (HR), overall survival (OS), 95% confidence intervals, and *p*-values were calculated to quantify the impact of integrin expression on overall survival. All analyses were conducted in R, with visualizations created using the survminer and ggplot2 libraries.

## **Cell models and 3D cell culture**

HNSCC cell models (Supplementary Table S1) were kindly provided by R. Grenman (Turku University Central Hospital, Turku, Finland), H. Bier (University of Düsseldorf, Germany) and T. E. Carey (University of Michigan, USA). Cells were asynchronously grown in Dulbecco’s modified Eagle’s medium (DMEM) containing glutamax-I (Sigma-Aldrich, # D5796) supplemented with 10% fetal calf serum (PAN Biotech, # P30-2206) and 1% non-essential amino acids (Gibco, # 11140-035) at 37°C in a humidified atmosphere containing 8.5% CO_2_ here after referred as cell culture medium or complete DMEM. For 3D cultures, cells were embedded in 1.0 mg/mL physiological 3D laminin-rich extracellular matrix (lrECM; Matrigel^R^ Matrix; Corning^R^, # 354248) and grown in 1% agarose-coated 24-well plates, as described previously ^17^. All cell models were authenticated using Short Tandem Repeat (STR) DNA profiling and tested negative for mycoplasma contamination. All cell experiments were performed on cell passage 3.

## **Small interfering RNA knockdown**

siRNA transfection was performed as previously described ^17^. Briefly, 24 hours after plating cells, ON-TARGETplus SMARTpool™ siRNA targeting specific genes or a non-targeting control siRNA (Horizon Discovery Dharmacon; Supplementary Table S1) were transfected using Lipofectamine™ RNAiMAX (Invitrogen, # 13778150) according to manufacturer’s instructions. Twenty-four hours post-transfection, cells were subjected to 3D culture or other assays as described.

## **Histone extraction and dot blot**

Twenty-four hours after seeding a total of 4 × 10⁵ cells in 3D lrECM, histones were extracted using the Histone Extraction Kit (Abcam, # ab113476). Extraction was performed as outlined by the manufacturer’s protocol. Dot blot was performed on equal amounts of histone extracts using the Bio-Dot microfiltration system (Bio-Rad). Prior to assembling the Bio-Dot system, nitrocellulose membranes (Cytiva, # 10600004) were pre-wetted in 1x tris-buffered saline (1x TBS) for 10 minutes. Membranes were sequentially washed with 100 µL of 20% ethanol, 100 µL ddH_2_O and 50 µL 1x TBS before and with 50 µL 1x TBS and 100 µL 1x TBS/0.05% tween-20 after sample loading. Membrane incubation with primary and secondary antibodies, as well as detection, was carried out as described previously ^17^. Primary antibodies were: anti-Histone H3 rabbit mAb (1:2000, Cell Signaling Technology, # 4499), anti-Acetyl-Histone H3 rabbit Ab (1:1000, Merck, # 06-599), anti-Mono-Methyl-Histone H3 (Lys4) rabbit mAb (1:1000, Cell Signaling Technology, # 5326), anti-Tri-Methyl-Histone H3 (Lys4) rabbit mAb (1:1000, Cell Signaling Technology, # 9751), anti-Tri-Methyl-Histone H3 (Lys36) (1:1000, Cell Signaling Technology, # 4909), anti-Tri-Methyl-Histone H3 (Lys27) rabbit mAb (1:1000, Cell Signaling Technology, # 9733), anti-Tri-Methyl-Histone H3 (Lys9) rabbit mAb (1:1000, Cell Signaling Technology, # 13969), and anti-β-actin mouse mAb (1:5000, Merck, # A5441). Secondary antibodies were: HRP-conjugated donkey-anti-rabbit Ab (1:2000, Cytiva, # NA934) and HRP-conjugated sheep-anti-mouse Ab (1:2000, Cytiva, # NXA931). Protein detection was performed using ECL Prime Western Blotting Detection Reagent (Cytiva, # RPN2236) and the Fusion FX imaging system (Vilber Lourmat GmbH). Data were processed and analyzed using Fiji (v1.53f).

## **K-mean clustering**

Prior to clustering, the dot blot data were scaled using StandardScaler to standardize feature values. K-means clustering was applied to identify patterns within the dataset using the scikit-learn library. The optimal number of clusters was determined using the elbow method and silhouette score analysis. Clusters were visualized using scatter plots with cluster-specific colors and centroid markers.

## **HNSCC population mutation analysis**

Population mutation analysis for HNSCC was conducted using the datasets published previously ^18^. Single nucleotide polymorphism (SNP) data were utilized to analyze mutation frequency and distribution through the SNP Nexus web tool (https://www.snp-nexus.org/v4/). The results were cross-validated with the Genome Aggregation Database (gnomAD) and Exome Aggregation Consortium (ExAC) to ensure accuracy and relevance to the HNSCC population.

## **Immunofluorescence staining and microscopy**

Twenty-four hours after seeding in lrECM, cells were fixed with 4% paraformaldehyde for 30 minutes and washed twice with 1x phosphate-buffered saline (1x PBS) for 15 minutes each. Permeabilization was performed with a 1x PBS/0.25% Triton X-100 for 20 minutes, followed by a 5-minute wash with 1x PBS. Cells were stained overnight at 4°C with 2 nM Hoechst 33342 (Sigma-Aldrich, # 63493) and primary anti-Acetyl-Histone H3 rabbit Ab (1:200, Merck, # 06-599), anti-Tri-Methyl-Histone H3 (Lys4) rabbit mAb (1:300, Cell Signaling Technology, # 9751), anti-Tri-Methyl-Histone H3 (Lys27) rabbit mAb (1:200, Cell Signaling Technology, # 9733), under gentle rotation. After staining, cells were washed three times with 1x PBS for 5 minutes each, and stained with Alexa Fluor 546-conjugated secondary goat anti-rabbit IgG Ab (1:200, Invitrogen, A-11055) for 1 hour at room temperature prior to embedment in fluorescence mounting medium (Dako; # S3023) and transfer to coverslips. Images were acquired on an LSM980 Airyscan 2 confocal microscope with Z-stack acquisition using 25× and 63× objectives.

## **Human studies**

HNSCC and normal tissue samples (Supplementary Table S1) were obtained from the tissue bank for tumor and normal tissues of the University Cancer Center (UCC) National Center for Tumor Diseases (NCT) Dresden. Patients gave informed consent as intended by the ethics committee of the Technische Universität Dresden (EK 59032007).

## **H&E, immunohistochemistry and immunofluorescence staining of patient material**

Parafﬁn sections of patient material were deparafﬁnized in xylene and rehydrated. Antigen retrieval was performed in 10 mmol/L citric acid, pH 6.0 at 98 C for 15 minutes, and sections were either stained with hematoxylin and eosin (H & E) or primary anti-α7 integrin rabbit Ab (1:100, Thermofisher, # PA5-49447) and secondary Alexa Fluor 546-conjugated goat anti-rabbit IgG Ab (1:200, Invitrogen, A-11010) for 1 hour at room temperature. Sections were mounted using fluorescence mounting medium (Dako; # S3023). Tissue sections were placed in ddH_2_O after imaging to remove mounting medium, followed by H & E staining. Images were acquired using an LSM980 Airyscan 2 confocal microscope (Carl Zeiss Microscopy GmbH) and Axio Imager M1 microscope (Carl Zeiss Microscopy GmbH) using 25× and 10× objectives.

## **Image analysis**

3D image analysis was conducted using Imaris (v9.1, Bitplane). Background signal was removed based on the default settings of the Imaris image processing. The "Surface" module was used for segmentation of nuclei. “Spot” module with sub setting volume was used to identify relative occupancy of the signal intensity in the nucleus. 3D reconstructions of segmented regions were rendered with color-coded overlays for different markers. Rotational animations and orthogonal views were generated to illustrate spatial relationships. 2D image analyses were done in Fiji (v1.53f) ^19^. Nuclei were segmented by StarDist ^20^ and intensity data were obtained. Videos were generated within Imaris, captured with Open Broadcaster Software (v30, OBS Project) and rendered with CapCut (v12.7.0., ByteDance).

## **Single-cell omics data analysis**

Publicly available single-cell RNA-seq data (GSE181919) were analyzed using Seurat, focusing on quality control, normalization, and clustering. Dimensionality reduction was performed using UMAP, and marker genes were identified using Wilcoxon rank-sum tests. Key outputs included UMAP plots, heatmaps, and gene correlation matrices, visualized using ggplot2, pheatmap, and SingleR.

## **RNA extraction**

Twenty-four hours after transfection of ITGA7 and control siRNA, cells were seeded in 3D lrECM. Samples for RNA extraction were prepared after another 24 hours using the NucleoSpin RNA kit (Machery-Nagel, # 740955.250). RNA integrity was assessed using the 4200 TapeStation (Agilent) according the manufacturers’ instructions and as described previously ^17^.

## **Reverse transcription-quantitative PCR (RT-qPCR)**

Complementary DNA (cDNA) synthesis was performed with 500 ng extracted RNA, 1 µL oligo(dT)_20_ (50µM; Life Tech Corp., # 58063), and 1 µL dNTP mix (10 mM each of dATP, dGTP, dCTP, and dTTP at neutral pH, Agilent, # 200415). The master mix was heated at 65°C for 5 minutes and incubated on ice for 1 minute. To this, 4 µL 5x First Strand Buffer (Invitrogen, # Y02321), 1 µL DTT (0.1 M, Sigma, # D0632), 1 µL RNaseOUT™ (Invitrogen, # 10777019), and 1 µL SuperScript™ III Reverse Transcriptase (200 U/µL; Invitrogen, # 100000840) were added. The reaction mixture was incubated at 50°C for 30-60 minutes, followed by enzyme inactivation at 70°C for 15 minutes. The synthesized cDNA was subjected to qPCR (QuantStudio 3; Thermo Fisher) using 10 µL TB Green Premix Ex Taq II (Tli RNaseH Plus, Takara; # RR820A), 0.8 µL each of forward and reverse primers (Supplementary Table S1, 10 µM), 0.4 µL ROX reference dye (50x, Tli RNaseH Plus, Takara; # RR820A), 4 µL cDNA product, and sterile nuclease-free water to a final volume of 16 µL per sample. Relative mRNA levels were calculated using the 2^-ΔΔC^T method. Pure water was used as a negative control, and housekeeping genes (Supplementary Table S1) were used for normalization.

## **RNA sequencing and analysis**

RNA sequencing was performed by the DRESDEN-concept Genome Center (Technische Universität Dresden, Germany) including quality control, library preparation, and mapping. Total RNA was prepared from two 3D lrECM HNSCC cell models (four biological replicates per cell line) using the RNeasy Kit with on-column genomic DNA digestion (Qiagen). Only RNA with an integrity number (RIN) of ≥ 9 was used for sequencing. mRNA was isolated from 500 ng of total RNA using the NEBNext rRNA depletion Kit (human, mouse, rat) from New England Biolabs (NEB, # E7400X) according to the manufacturer’s instructions. Final elution was done in 5 µL nuclease free water. Samples were then directly subjected to the workflow for strand specific RNA-Seq library preparation with the NEBNext Ultra II Directional RNA Library Prep assay (NEB, # E7760L). After ligation and user digest, adaptors were depleted using XP bead purification (Beckman Coulter) at a 0.9:1 bead-to-sample ratio. Unique dual indexing was done during the following PCR enrichment (12 cycles) using amplification primers carrying the same sequence for i7 and i5 index (i5: AAT GAT ACG GCG ACC ACC GAG ATC TAC AC NNNNNNNN ACA TCT TTC CCT ACA CGA CGC TCT TCC GAT CT, i7: CAA GCA GAA GAC GGC ATA CGA GAT NNNNNNNN GTG ACT GGA GTT CAG ACG TGT GCT CTT CCG ATC T). After two more XP bead purifications (0.9:1), libraries were quantified using the Fragment Analyzer (Agilent). Libraries were pooled equimolarly and sequenced on an NovaSeq 6000 (Illumina) in 100 bp paired-end modeI generating approximately on average 45 million read pairs per library. Raw sequencing data underwent basic quality control, normalization, and transcriptome analysis. Reads were aligned to the human reference genome hg38 using Ensembl 104 ^21^ splice sites and the aligner GSNAP (v2020-12-16) ^22^ with the custom settings “-B 5 -n1 -m 0.6 -N 0”. Gene counts per sample were obtained by overlapping uniquely mapped fragments with the Ensembl annotation using featureCounts (v2.0.1) ^23^ with settings “-s 2 -p -Q 1.” Normalization of raw fragments by library size and differential expression analysis between conditions were performed using the DESeq2 R package (v1.30.1) in R (v4.0.4) ^24^. Principal Component Analysis (PCA) of the 500 most variable genes was conducted to assess relationships between biological replicates and libraries. Differentially expressed genes were identified using the Wald test in DESeq2, with *p*-values adjusted for multiple testing using the Independent Hypothesis Weighting package (IHW v1.18.0) ^25^. Genes with *p* ≤ 0.05 and FDR < 0.05 were considered significantly differentially expressed.

## **Nuclei Isolation**

Cells were centrifuged at 300 × g for 5 minutes, and the supernatant was carefully removed. Twice the pellet volume of Corning® Cell Recovery Solution (Corning; # 354253) was added and the suspension was incubated on ice for 1 hour. After incubation, cells were centrifuged again at 300 × g for 5 minutes, and the resulting pellet was washed with ice-cold 1x PBS. The cell pellet was gently lysed with 45 µL of ice-cold lysis buffer (10 mM Tris-HCl [pH 7.4], 10 mM NaCl, 3 mM MgCl₂, 0.01% Digitonin, 0.1% Tween-20, and 0.3% BSA). The lysate was mixed by gentle pipetting, and nuclei were pelleted by centrifugation at 500 × g for 10 minutes at 4°C. The supernatant was removed, and the nuclei were washed twice with 45 µL of ice-cold wash buffer (10 mM Tris-HCl [pH 7.4], 10 mM NaCl, 3 mM MgCl₂, 0.1% Tween-20, and 0.3% BSA). Nuclei integrity was checked under a microscope. Nuclei were resuspended in 5 µL of a prepared transposition mix containing 2.5 µL of 2x Tagment DNA Buffer (Illumina, # 20034210), 0.5 µL of Tagment DNA Enzyme (Illumina, # 20034210), and 2 µL of wash buffer. The nuclei were incubated with the transposition mix at 37°C for 60 minutes in a thermocycler (Mastercycler epgradient, Eppendorf) with the lid set to 50°C. Following the transposition reaction, DNA was purified using the MinElute Reaction Cleanup Kit (QIAGEN, # 28206) according to the manufacturer’s instructions. The purified DNA was quantified with NanoDrop™ One^C^ spectrophotometer (Thermo Fisher, # AZY2019672).

## **ATAC sequencing and peak analysis**

Nuclei for Assay for Transposase-Accessible Chromatin using sequencing (ATAC-seq) were extracted forty-eight hours after transfection of ITGA7 and control siRNA, cells were seeded in 3D lrECM. After tagmentation and purification 10 µL of tagmented DNA was pre-amplified in a 24 µL PCR reaction containing 1X KAPA HiFi HotStart ReadyMix (Roche, # 7958935001), 0.08 µM unique dual Nextera indexing primers (i7: 5’-CAAGCAGAAGACGGCATACGAGAT XXXXXXXX GTCTCGTGGGCTCGG, i5: 5’-AATGATACGGCGACCACCGAGATCTACAC XXXXXXXX TCGTCGGCAGCGTC). Cycling conditions were 72 °C 5 min, 98 °C 30 s, 5 cycles [98 °C 10 s, 63 °C 30 s, 72 °C 1 min]. Final elongation was skipped and the sample was put on ice. To determine the final number of indexing 1 µL of the pre-amplified PCR product was used in a 10 µL qPCR containing 1X KAPA HiFi HotStart ReadyMix (Roche, # 7958935001), 0.2 µM universal amplification primers (P5: 5’-AATGATACGGCGACCACCGAG and P7: 5’-CAAGCAGAAGACGGCATACGAG), and 0.7 X SYBR Green (Biotium, # 41004). The qPCR cycling conditions on a LightCycler 480 (Roche) were 98 °C 1 min, 15 cycles [98 °C 20 s, 63 °C 30 s, 72 °C 1 min]. After baseline subtraction of the fluorescence data using the LightCycler Software (Roche), the optimal cycle number of additional 8 cycles was determined as the cycle reaching approx. 25% of the plateau fluorescence signal intensity. PCR conditions were as for the pre-amplification. After XP bead-based size selection (Beckman Coulter, # A63882) with 1^st^ 1.55 X / 2^nd^ 0.6 X bead to sample ratio, libraries were quantified using the Fragment Analyzer (Agilent) and sequenced with 2 x 100 bp paired end sequencing mode on an Illumina Novaseq 6000 (Illumina) with an average sequencing depth of 50 million read pairs per library. Sequencing data were processed following the ENCODE official pipeline specifications (https://github.com/kundajelab/atac_dnase_pipelines). Adapter sequences were removed, and reads were mapped to the hg38 reference genome in paired-end mode using Bowtie, an ultrafast, memory-efficient read aligner. Duplicate and mitochondrial reads were excluded. ATAC peaks were identified using Zinba (Zero Inflated Negative Binomial Algorithm), a statistical framework for genome-wide analysis. The density of ATAC peaks near Transcription Start Site (TSS) of unchanged versus down-regulated genes was determined by extending the TSS region 100 kb in both the 5′ and 3′ directions. The mean ATAC peak density in each window was plotted as a function of distance from the TSS, with signal density expressed in units of reads per million per base pair (rpm/bp). Bioinformatics analysis was conducted using the nf-core atacseq pipeline (v1.2.1) ^26^ with the singularity image “atacseq_latest.sif” (from 21.3.2022), human reference genome hg38, and Ensembl 104 annotation. Custom pipeline settings included “--mito-name ‘chrM’ --macs_gsize ‘2.7e9’“. This pipeline performed all required steps, including raw read quality control, adapter trimming, alignment, filtering, normalized coverage track generation, peak calling, and peak annotation based on the nearest gene. A consensus peak set was created across all libraries, and differential accessibility analysis was performed. Peaks were additionally visualized using the Integrated Genomics Viewer (IGV).

## **Transcription factor motif identification**

Motif analysis on the subset of peaks was conducted using HOMER ^27^. The findMotifsGenome.pl command was applied to a BED file containing differentially accessible regions. *De novo* motif discovery was performed, and the identified motifs were compared against known motifs in the database to identify the best matches together with predicted motif analysis from transcription data from IPA (Qiagen). This approach allowed for the detection of sequence motifs associated with the differentially accessible regions.

## **Kinex^TM^ antibody microarray**

3D lrECM grown ITGA7 and control siRNA-treated Cal-33 and SAS cultures were lysed using the Kinexus lysis buffer with protease inhibitors and dithiothreitol according to the manufacturer's guidelines, specifically the "suspended cell lysates" method. Suspended cells were centrifuged at 500 x g for 2 minutes at 4°C in a swinging bucket benchtop centrifuge and cell pellet was washed twice with ice-cold 1x PBS. Lysates were centrifuged at maximum speed in a benchtop microcentrifuge for 30 minutes at 25°C and the supernatant was shipped to Kinexus Bioinformatic Corporation (Canada), where it was processed for analysis using the KAM-2023 antibody microarray (Supplementary Table S1). In brief, 50 - 100 µg of lysates were biotin-labeled and incubated on the microarray. Following incubation and washing to remove unbound proteins, the microarray was probed with fluorescent dye-labeled anti-streptavidin antibodies. Fluorescent signals were scanned using a Perkin-Elmer ScanArray Reader, and signal quantification was performed using ImaGene 9.0 software from BioDiscovery (El Segundo; CA), employing predefined settings for spot segmentation and background correction. The data were globally normalized, and the percentage change from control (%CFC) was calculated to identify significant changes in protein expression or phosphorylation. Microarray output included the average normalized net signals, calculated as the mean of two normalized net signal values for each antibody. Standard error and percent standard deviation were derived from two independent measurements of globally normalized signal intensity for each antibody. Positive %CFC values indicated an increase in signal intensity due to treatment, with 100% corresponding to a two-fold increase, while negative values reflected a reduction relative to control. Both raw and background-normalized signals were reported. To standardize the signal readout further, the normalized median signal was divided by the dot area and the log fold changes between ITGA7 knockdown and corresponding controls were calculated and log_2_-transformed for statistical analyses.

## **Multi-omics clustering**

Multi-omics data (scRNA-seq; scATAC-seq; and related modalities) were processed using Python with libraries including Pandas for data manipulation, NumPy for numerical computations, Matplotlib and Seaborn for visualization, and SciPy for statistical operations such as correlation, clustering, and peak detection. Interaction data from multiple datasets were combined into a single dataframe, and unique gene IDs were extracted from the "Interaction Partners" column to construct a binary matrix, representing the presence or absence of interactions. Correlation and distance matrices were computed using Pandas and SciPy, and hierarchical clustering was performed with the linkage method from SciPy’s hierarchy module. Peaks and valleys in the data were detected automatically using a spline-smoothed representation of binned correlation sums, fitted with UnivariateSpline from SciPy, and the find_peaks function was used to identify prominent features based on parameters like height and prominence thresholds. The binning approach used constant-sized bins to calculate averages for smoothed plots, enabling automated and consistent peak detection. Clustered heatmaps were visualized using Seaborn’s clustermap, with custom colormaps created via Matplotlib's LinearSegmentedColormap. Heatmaps incorporated hierarchical clustering annotations to delineate correlation-based groupings.

## **Machine learning and feature selection**

A Random Forest (RF) classifier was trained using scikit-learn to classify experimental conditions, with hyperparameter tuning via RandomizedSearchCV. The hyperparameter grid included n_estimators (100-1000), max_depth (10; 20; None), min_samples_split, min_samples_leaf, max_features, and class_weight. Model evaluation was performed using stratified train-test splitting (train_test_split) with a 75%-25% split, metrics from classification_report and confusion_matrix, and cross-validation with StratifiedKFold (n_splits). Learning curves were generated with learning_curve to analyze performance trends. Feature importances were extracted from the trained Random Forest model and visualized using Matplotlib and Seaborn to identify the top contributing proteins. PCA (scikit-learn.decomposition.PCA) was applied to analyze protein contributions to principal components, and overlaps with Random Forest-derived features were identified. Nested cross-validation was implemented using cross_val_score with an outer StratifiedKFold loop to obtain unbiased performance estimates and mitigate overfitting.

## **Generation of patient-derived HNSCC organoids (PDO)**

HNSCC patient-derived organoids (PDOs; Supplementary Table S1) were established from fresh primary tumor biopsies obtained from HNSCC patients surgically treated at the University Hospital Dresden. The study was conducted in accordance with institutional review board guidelines and approved by the Landesdirektion Sachsen (April 16, 2025; approval no. BO-EK-241052022). Written informed consent was obtained from all patients.

Organoids were cultured similar as previously published ^17^. Shortly organoid droplets in Matrigel (Corning, # 356231). Basal medium consisted of Advanced DMEM/F12 (Thermo Fisher Scientific, # 12634010; pre-oxygenated) supplemented with 1% penicillin/streptomycin (Thermo Fisher Scientific, # 15140122; used only during organoid disruption and matrix removal), 1% GlutaMAX (Thermo Fisher Scientific, # 35050061), and 10 mM HEPES (Thermo Fisher Scientific, # 15630080).

The complete expansion medium additionally contained: 1× B27 (Gibco, # 17504044), 10 mM nicotinamide (Sigma-Aldrich, # N0636), 1.25 mM N-acetylcysteine (Sigma-Aldrich, # A9165), 0.5 µM A-83-01 (Tocris, # 2939), 1 µM prostaglandin E₂ (Tocris, # 2296), 0.3 µM CHIR99021 (Sigma-Aldrich, #SML1046), 1 µM forskolin (Tocris, # 1099), 50 ng/mL human EGF (PeproTech, # AF-100-15), 10 ng/mL human FGF10 (PeproTech, #AF-100-26), 5 ng/mL human FGF2 (PeproTech, #AF-100-18B), 4% (v/v) R-spondin3-Fc conditioned medium (# R001, AVS Bio Netherlands B.V.), and 4% (v/v) Noggin-Fc conditioned medium (# N002, AVS Bio Netherlands B.V.). Where indicated, puromycin (InvivoGen, # ant-pm-1) was added.

Organoids were embedded as 20 µL Matrigel droplets per well and overlaid with 250 µL expansion medium in pre-warmed plates. Droplets were allowed to polymerize for 15 min at 37°C before medium addition. Cultures were maintained at 37°C in a humidified incubator with 5% CO₂.

For single-cell seeding experiments, Matrigel-organoid droplets were collected by pipetting and pooled (up to four wells) into a 15 mL tube. Suspensions were adjusted to ~2 mL with basal medium and mechanically dissociated using flame-thinned glass pipettes for ~3 min to disrupt the matrix and disperse organoids. The volume was increased to 5 mL followed by centrifugation at 400 × g for 5 min. Pellets were incubated with TrypLE™ Express (Thermo Fisher Scientific, # 12604013) for 2 min at 37°C and mechanically dissociated by pipetting with a 1000 µL tip until a single-cell suspension was obtained. Enzymatic activity was stopped with basal medium followed by centrifugation at 400 × g for 5 min. Cells were resuspended in organoid expansion medium and reseeded in Matrigel droplets (20 µL per well) in pre-warmed culture plates. Where indicated, culture medium was supplemented with ROCK inhibitor Y-27632 (10 µM; Tocris, # 1254) and puromycin during reseeding.

For cryopreservation, Matrigel was dissolved using Cell Recovery Solution (Corning, # 354253) on ice for ≥30 min. Organoids were washed with PBS, centrifuged at 400 × g for 5 min, and resuspended in Recovery™ Cell Culture Freezing Medium (Thermo Fisher Scientific, # 12648010). Cryovials were frozen at -80°C using a controlled-rate freezing container and subsequently transferred to liquid nitrogen for long-term storage.

## **gRNA cloning**

ITGA7-targeting gRNAs were designed using the Synthego CRISPR design tool and cloned into the pL.CRISPR.puro vector (provided by J. Mircetic, Deutsches Konsortium für Translationale Krebsforschung (DKTK) and Deutsches Krebsforschungszentrum (DKFZ)). The following gRNA target sequences were used: gRNA1, 5’-CCAGGAAGAGCCAGGGCCTG-3’; gRNA2, 5’-GGAGACTGACTGCTACAGAG-3’; and gRNA3, 5’-AGAGTGGACATCGACCAGGG-3’. For oligonucleotide annealing and phosphorylation, 1 µL of 100 µM top oligo and 1 µL of 100 µM bottom oligo were mixed with 1 µL of 10× T4 ligation buffer (New England Biolabs (NEB), # M0202S) and 0.5 µL of T4 polynucleotide kinase (PNK; NEB, # M0201S) and the volume was adjusted with H_2_O to a final volume of 10 µL. The reaction was incubated in a PCR cycler with the following program: 37°C for 30 min, 95°C for 5 min, followed by cooling to 25°C at a ramp rate of 5°C per min, and final holding at 8°C.

Golden Gate ligation (modified NEB protocol) was performed using pL.CRISPR.puro vector (650 ng/µL; volume adjusted to provide 100 ng per reaction), 2 µL of 1:250 diluted annealed oligonucleotides, 2 µL of 10× Tango buffer (Thermo Fisher Scientific, # BY5), 1 µL DTT (Sigma-Aldrich, # D0632), and 1 µL Esp3I (BsmBI, 10 U/µL; LifeTech, # ER0451), with H_2_O added to a final volume of 20 µL. The reaction was incubated in a PCR cycler at 37°C for 5 min and 20°C for 5 min for 5 cycles, followed by heat inactivation at 65°C and holding at 8°C.

The ligation product was mixed with 40 µL electrocompetent XL1-Blue bacterial cells in glycerol (provided by J. Mircetic (DKTK, DKFZ)) and electroporated at 1,700 V (Eppendorf Eporator). Cells were immediately recovered in 1 mL SOC medium (Invitrogen, # 15544-034) and incubated for 1 h at 37°C with shaking at 900 rpm. After recovery, 80 µL of the bacterial suspension was plated on LB-ampicillin agar plates (Carl ROTH, # X964) and incubated at 37°C for 12 h. Three single colonies were selected, plasmid DNA was purified using a GeneJET Plasmid Miniprep kit (LifeTech, # K0503), and constructs were verified by Sanger sequencing. Confirmed plasmids were subsequently amplified by NucleoBond Xtra Maxi prep kit (Th. Geyer, # 11699290) and stored at -80°C.

## **Lentivirus production in HEK293T cells**

HEK293T cells (provided by J. Mircetic (DKFZ, Germany)) were seeded and incubated for 24 h at 37°C and 5% CO_2_. After incubation, the culture medium was removed and the cells were transfected with lentiviral plasmids carrying the ITGA7 gRNA plasmids (3,600 ng), psPAX2 packaging plasmid (21,600 ng; # 12260, Addgene, Watertown, MA, USA), and pMD2.G envelope plasmid (7,200 ng; # 12259, Addgene), both generously provided by D. Trono. The plasmids were mixed in 2 mL DMEM containing 2 mL polyethylenimine (PEI, 1 mg/mL diluted in DMEM; Sigma-Aldrich, # 408727) and subsequently combined with 17 mL complete DMEM. The transfection mixture was then added to the culture flask. After 24 h, the transfection medium was removed and replaced with 15 mL complete DMEM. Two days later, the supernatant containing lentiviral particles was collected and concentrated using Amicon Ultra centrifugal filters (Sigma-Aldrich, # UFC910024) by centrifugation for 47 min at 1,700 × g at room temperature to produce final virus suspension stored at -80°C.

## **Lentiviral transduction of organoids and cell models**

Organoids and cell models were prepared for transduction by first generating single-cell suspensions as described above. A total of 752 µL of the single-cell suspension was added to each well of a 48-well plate. Subsequently, 8 µL of diluted (1:10) protamine solution (5 mg/mL; Sigma-Aldrich, # P3369) was added to each well, followed by 40 µL of viral suspension. Protamine was used to enhance viral uptake. Immediately before plating, the cell suspension was homogenized by repeated pipetting to ensure equal cell distribution across wells.

To increase transduction efficiency, plates were subjected to spin-inoculation for 1 h at 37°C and 700 × g using centrifuge settings of acceleration 4 and deceleration 2. The 48-well plate was placed directly into the centrifuge according to the established protocol. After spin-inoculation, cells were incubated for 4-5 h under standard culture conditions to allow viral entry and initial recovery. At the end of the incubation period, cells were transferred into Falcon tubes containing 7 mL organoid medium and centrifuged at 300 × g for 5 min. The supernatant was removed, and the pellet was resuspended in Matrigel for reseeding.

gRNA cutting efficiency was estimated by PCR amplification of the genomic region surrounding the target site using the following primers: forward, 5’-ACTCACAACAATCTTGCCCC-3’; reverse, 5’-TTAGTACTGAGGGGTTGGGC-3’. Bulk PCR products were subjected to Sanger sequencing, and indel frequency as well as cutting score were determined using the Synthego ICE analysis tool.

## **Organoids viability**

Five days after single cell seeding, organoid viability was assessed using the CellTiter-Glo 3D assay (Promega, # G9681). When organoids reached the appropriate stage for analysis described in our previous study ^17^, the culture medium was carefully removed from each well of the 48-well plate to ensure that no residual liquid remained.

For each organoid-containing well, 130 µL of CellTiter-Glo 3D reagent was added directly to the well. To initiate lysis, Matrigel and organoids were gently disrupted using a pipette tip. The plate was placed on a rotator and shaken at 500 rpm for 5 minutes. Following rotation, the plate was transferred to a dark environment and each well was mixed thoroughly by pipetting to ensure complete lysis of the organoids. The plate was then incubated in the dark for 30 minutes at room temperature.

After incubation, wells were inspected to confirm complete organoid lysis. Subsequently, 100 µL of each lysate was transferred into a white 96-well plate for luminescence measurement.

Luminescence was quantified using a SpectraMax iD3 (Molecular Devices) according to the standard CTG-3D assay protocol.

## **3D cell viability assay**

Twenty-four hours post siRNA transfection, 6×10^3^ cells were embedded in 1.0 mg/mL lrECM in 96-well plates, as previously described ^17^. Cell viability was assessed using the 3D CellTiter-Glo^®^ 3D Assay (Promega, # G9683) following the manufacturer’s instructions. The results were normalized to 1 µM ATP and presented as a percentage ratio of treatment versus control conditions, calculated by dividing the average viability under the indicated treatment by the average viability of the respective control.

## **3D colony formation assay**

Following siRNA-mediated knockdown, cells were embedded in 1.0 mg/mL lrECM within 96-well plates, as detailed previously ^17^. After a cell model-dependent growth period, colonies were fixed using a 4% formaldehyde solution in 1x PBS. Microscopic focus-stacked image acquisition was performed using a Cytation 5 reader (BioTek). Colony counting and area analysis were conducted using Fiji software, with all colonies having a minimum diameter of 50 µm included in the analysis.

## **3D proliferation assay**

Cell proliferation was analyzed as published previously ^28^. Briefly, Cal-33 and SAS cells were treated with siRNAs and cultured in 3D lrECM. After 24 hours, the cells were prepared for imaging, and the proportion of BrdU- and Ki67-positive cells was determined by immunofluorescence staining. Cells were incubated with 10 µM BrdU (Invitrogen, # B23151) for 2 hours, followed by washing with 2x PBS. Subsequently, cells were trypsinized for 1 hour at 37°C, washed again with 1x PBS, and fixed with ice-cold 4% formaldehyde. DNA denaturation was achieved by incubating cells with 2 M HCl for 30 minutes at room temperature. Permeabilization was performed using 1x PBS/0.25% Triton-X100, and cells were blocked with 1x PBS/1% BSA. Primary antibodies anti-BrdU mouse mAb (1:100, BD, # 347580) and anti-Ki67 rabbit Ab (1:200, Abcam, # ab15580), were applied overnight at 4°C. Secondary antibodies were: Alexa Fluor 594-conjugated goat anti-rabbit IgG (1:500, Life Technology, # A11010) or Alexa Fluor 488-conjugated goat anit-mouse IgG (1:500, Life Technology, # A11029) along with 2 nM Hoechst 33342 (Sigma-Aldrich, # 63493). The cell-matrix complexes were embedded in fluorescence mounting buffer medium (Dako; # S3023) and transferred to coverslips. Imaging was performed as previously described.

## **ΔViability and ΔPathScore analyses**

To identify genes likely to act in the same signaling pathway as ITGA7, we performed a combinatorial knockdown screen targeting 63 candidate genes (Supplementary Table S1), both individually and in combination with ITGA7. Cell viability was measured for each condition. For each candidate gene, we calculated a ΔViability score, defined as the difference between the viability of the double knockdown (ITGA7 + Gene A) and the more severe of the two corresponding single knockdowns. Formally:

$$\Delta\text{Viability}=\text{Viab}_{ITGA7+A}-\min(\text{Viab}_{ITGA7},\text{Viab}_{A})$$

A ΔViability close to zero suggests that the candidate gene and ITGA7 act in the same pathway (i.e., no additional effect when both are silenced). To assess probability of belonging to same signaling axis, we transformed the absolute ΔViability values into a ΔPathScore, ranging from 0 to 1, where higher values indicate greater likelihood of pathway co-membership. This score was calculated using a logistic-like decay function:

$$\Delta\text{PathScore}=\frac{1}{1+{(\frac{\mid\Delta\text{Viability}\mid}{T})}^{k}}$$

with $T=0.05$ and $k=3$, empirically chosen to model biological tolerance to minor deviations. Genes with ΔPathScore ≥ 0.9 were considered candidates for acting in the same pathway as ITGA7

## **Clinical relevance**

TCGA HNSCC patient expression data were used to calculate ITGA7 tumor expression relative to the mean expression across normal samples. For each patient $i$, expression was calculated as

$$x_{i}\equiv\log_{2}\left( \frac{E_{i}^{\text{tumor}}}{\bar{E}^{\text{normal}}} \right)$$

where $E_{i}^{\text{tumor}}$ is the ITGA7 tumor expression in patient $i$, and $\bar{E}^{\text{normal}}$ is ITGA7 expression across the normal cohort. Survival clinical metadata were matched to expression profiles and included overall survival time $T_{i}$(days) and event status $\delta_{i}$. Survival variables were converted to numeric format, and patients lacking ITGA7 or survival information were excluded. Patients were then ordered by increasing ITGA7 log_2_ fold change, and all downstream analyses were performed along this ranked expression axis, enabling continuous evaluation of survival patterns without imposing arbitrary high / low thresholds. Survival along the ITGA7 expression axis was evaluated using overlapping sliding windows of fixed size $W=80$patients, advanced in steps of $S=10$ patients. For each window w, starting at rank $s_{w}$, the index set was defined as

$$\mathcal{I}_{w}=\left\{ s_{w},s_{w}+1,\ldots,s_{w}+W-1 \right\}$$

where $s_{w}\in\{1,1+S,1+2S,\ldots\text{ }\}$ and $s_{w}+W-1\leq N$. Each window therefore represents a local neighborhood of patients with similar ITGA7 fold change. For visualization, each window was positioned along the x-axis using the median ITGA7 expression within that window,

$$\tilde{x}_{w}=\text{median}\{x_{\left( i \right)}:i\in\mathcal{I}_{w}\}$$

thereby reducing the influence of outliers in overlapping windows. Specifically, within each window $w$, survival was estimated using the Kaplan-Meier (KM) method,

$$\hat{S}_{w}(t)=\prod_{t_{j}\leq t} \left( 1-\frac{d_{j}}{n_{j}} \right)$$

where $t_{j}$ are distinct event times, $d_{j}$ is the number of deaths at time $t_{j}$, and $n_{j}$ is the number at risk immediately prior to $t_{j}$, computed using survival data $\left( T_{i} , \delta_{i} \right)$ for patients $i\in\mathcal{I}_{w}$. Survival was summarized using the restricted mean survival time (RMST), defined as the area under the survival curve up to a restriction time $\tau$,

$$\text{RMST}_{w}(\tau)\equiv\int_{0}^{\tau} \hat{S}_{w}(t)\text{ }dt$$

Rather than applying a fixed global time horizon, a window-specific restriction time was defined as

$$\tau_{w}\equiv\max\{T_{i}:i\in\mathcal{I}_{w}\}$$

such that survival in each window was evaluated up to the maximum observed follow-up within that group. Survival was evaluated only over the observed follow-up within each window, without extrapolation beyond the maximum observed time. As the time range automatically adjusts to how long patients in that local group were followed, the survival estimate is strictly local and not influenced by data from outside that neighborhood. Because the Kaplan-Meier curve is a right-continuous step function, the RMST was computed exactly as the sum of rectangular areas between successive event times,

$$\text{RMST}_{w}^{\text{max}}=\sum_{k=0}^{m-1} \hat{S}_{w}(t_{k})(t_{k+1}-t_{k})$$

where $0=t_{0}<t_{1}<\cdots<t_{m}\leq\tau_{w}$ where $\tau_{w}$ is the window-specific maximum observed follow-up time. Between two adjacent time points $t_{k}$ and $t_{k+1}$, the survival probability, $\hat{S}_{w}(t)$ remains constant, therefore, each interval contributes a rectangle with height $\hat{S}_{w}(t_{k})$ and width $t_{k+1}-t_{k}$. The total RMST is obtained by summing these rectangular areas across all intervals. If $\tau_{w}$exceeds the last observed event time, the survival curve remains flat and is extended to $\tau_{w}$, ensuring full integration over observed follow-up. Because sliding windows overlap substantially, apparent survival structure along the ITGA7 axis may arise from random fluctuation. To distinguish expression-associated survival patterns from random variation, an empirical null distribution was constructed using outcome permutation. Under the null hypothesis, survival outcomes $\left( T_{i} , \delta_{i} \right)$ are independent of ITGA7 expression ranking (simply speaking the fold ranking is unchanged but the clinical data associate is changed). A total of $P=2000$ permutations were generated by randomly shuffling paired survival outcomes while preserving ITGA7 ordering,

$$(T_{i},\delta_{i})\leftarrow(T_{\pi_{p}(i)},\delta_{\pi_{p}(i)})$$

thereby maintaining the marginal survival-time distribution and censoring structure while removing any association with expression. For each permutation and each window $w$, RMST was recomputed using the same window membership and window-specific maximal restriction time, yielding a null RMST distribution $\left\{ \text{RMST}_{w,1}^{\text{max}},\ldots,\text{RMST}_{w,P}^{\text{max}} \right\}$. Central null bands were then defined using empirical quantiles of this distribution.

To visualize the expected range of RMST values under the null hypothesis, central null bands were computed at multiple coverage levels. For a given coverage $c\%$, the band limits were defined using empirical quantiles of the permutation-based RMST distribution as

$$\left[ Q\left( \frac{1-c/100}{2} \right) , \text{ }Q\left( 1 - \frac{1-c/100}{2} \right) \right]$$

where $Q(p)$ denotes the empirical $p$ the quantile of the null RMST distribution for window $w$. For example, the 95% null band corresponds to $\left[ Q(0.025),Q(0.975) \right]$. In addition, the null median curve was defined as

$$\text{median}_{p}\left( \text{RMST}_{w,p}^{\text{max}} \right)$$

and used as the baseline expectation under random association between ITGA7 expression and survival. Observed windows in which $\text{RMST}_{w}^{\text{max}}$ deviated beyond high coverage null bands were interpreted as exhibiting survival behavior unlikely under the null hypothesis, given the exact window overlap structure and censoring distribution preserved in the permutation framework.

To quantify the magnitude and direction of survival differences, each window was compared to the remainder of the cohort and defined by

$$\Delta_{w}=\text{RMST}^{\text{max}}(\mathcal{I}_{w})-\text{RMST}^{\text{max}}(\mathcal{R}_{w})$$

where $\mathcal{R}_{w}=\{1,\ldots,N\}\setminus\mathcal{I}_{w}$. Because RMST uses group-specific maximal follow-up, restriction times were defined separately for the window and the remainder as

$$\tau_{\mathcal{I}_{w}}=\max\{T_{i}:i\in\mathcal{I}_{w}\},\tau_{\mathcal{R}_{w}}=\max\{T_{i}:i\in\mathcal{R}_{w}\}$$

Thus, survival in both groups was evaluated strictly within the follow-up depth supported by each respective subset.

To estimate uncertainty and robustness of this contrast, nonparametric bootstrap resampling (2000 replicates) was performed independently within the window and within the remainder. For bootstrap replicate $b$, samples were drawn with replacement,

$$\mathcal{I}_{w}^{\left( b \right)}\sim\text{Bootstrap}(\mathcal{I}_{w}),\mathcal{R}_{w}^{\left( b \right)}\sim\text{Bootstrap}(\mathcal{R}_{w})$$

and the bootstrap contrast was computed as

$$\Delta_{w}^{\left( b \right)}=\text{RMST}^{\text{max}}(\mathcal{I}_{w}^{\left( b \right)})-\text{RMST}^{\text{max}}(\mathcal{R}_{w}^{\left( b \right)})$$

The dominance probability was defined as

$$P_{w}\equiv\Pr(\Delta_{w}>0)\approx\frac{1}{B}\sum_{b=1}^{B} \mathbf{1}[\Delta_{w}^{\left( b \right)}>0]$$

with $P_{w}\to1$ indicating a robust survival advantage, $P_{w}\to0$indicating disadvantage, and $P_{w}\approx0.5$ indicating no consistent difference.

To quantify effect magnitude and uncertainty, the bootstrap distribution of the window-versus-remainder contrast $\Delta_{w}$ was summarized. Specifically, the bootstrap median of $\Delta_{w}$ was used as a robust estimate of effect size, and the 2.5% and 97.5% quantiles defined a nonparametric 95% confidence interval. These statistics provide a reliable measure of survival advantage or disadvantage and its stability under resampling. An empirical two-sided permutation *p* - value was computed for each window by comparing the observed RMST to its permutation-derived null distribution. The *p* - value was defined as

$$p_{w}=2\min\left( \Pr(\text{RMST}_{w,p}^{\text{max}}\geq\text{RMST}_{w}^{\text{max}}),\Pr(\text{RMST}_{w,p}^{\text{max}}\leq\text{RMST}_{w}^{\text{max}}) \right)$$

where probabilities were estimated from the permutation distribution. To avoid zero *p* - values due to finite sampling, a standard +1 correction was applied. Specifically,

$$\Pr(\text{null}\geq\text{obs})\approx\frac{\#\{\text{null}\geq\text{obs}\}+1}{P+1}$$

where $P$ is the total number of permutations and $\#\{\text{null}\geq\text{obs}\}$ indicates the number of permuted RMST values greater than or equal to the observed value. This empirical *p* - value provides a window-wise statistical descriptor that is fully consistent with the permutation-derived null bands shown in the visualization.

ITGA7 expression was evaluated for its ability to stratify TCGA HNSCC patients into discrete survival-relevant subgroups. To avoid arbitrary threshold selection, all valid 4-bin partitions of the ranked cohort were exhaustively scanned, generating four contiguous groups: “Lowest” (G1), “Low” (G2), “High” (G3) and “Highest” (G4). Candidate splits were defined by three ordered cut indices $\left( i , j , k \right)$, which partitioned the cohort into four contiguous groups:

$$G_{1}=\{1,\ldots,i+1\},G_{2}=\{i+2,\ldots,j+1\},G_{3}=\{j+2,\ldots,k+1\},G_{4}=\{k+2,\ldots,N\}$$

To avoid unstable splits driven by small subsets, a minimum group size constraint was applied such that each bin contained at least $M$ patients (MIN_BIN_N = 50), yielding feasible bounds $i\in[M-1,N-3M-1]$, $j\in[i+M,N-2M-1]$, and $k\in[j+M,N-M-1]$. For each candidate split, survival separation was quantified using the multivariate log-rank test across all four groups, producing a global *p* - value $p_{\text{overall}}$ for the null hypothesis of identical survival functions. In addition, all six pairwise group comparisons were tested using two-sample log-rank tests.

Combinatorial subgroup analysis was performed incorporating ITGA7-associated signature genes derived from integrative feature-selection and survival analyses. Patients were stratified into high and low expression groups based on median expression within the analyzed sub-cohort. ITGA7-based subgrouping was defined either by rank-based extremes (Top100 vs Bottom100) or by sign relative to zero (log2 fold change > 0 vs < 0). Subgroups were constructed from combinations of ITGA7 and gene expression states, and Kaplan–Meier survival probabilities were calculated at 5 years. To account for subgroup size, a size-adjusted survival metric (weighting factor) was defined:

$$Patient\_KM\_surv(t)=KM\_surv(t)\times N_{\text{group}}\times1000$$

where $KM\_surv(t)$is the Kaplan-Meier survival probability at time $t$ and $N_{\text{group}}$ is the number of patients in the subgroup. It rescales survival by subgroup size to provide a stability-aware survival intensity score.

## **Chromatin accessibility assay**

After knockdown, cell models were harvested and gently lyzed to isolate intact nuclei (see nuclei isolation method). In short, nuclei were incubated with Tn5 transposase in tagmentation buffer at 37°C for one hour to fragment accessible chromatin. After the reaction, genomic DNA was purified and quantified spectrophotometrically. Promoter accessibility was assessed by quantitative PCR (see qPCR method). For this method, an additional step was to equalize the genomic DNA input across samples. We designed two primers: an AURKA promoter primer and a primer for a constitutively open chromatin region (positive control) identified by ATAC-seq analysis from both cell models (Supplementary **Table** S1). All data were normalized to the positive control instead of the housekeeping gene used in the qPCR method above. All resulting fold changes were transformed to negative log2 for simplicity, because Tn5 transposase digests open chromatin (regions with more cuts), which lowers the starting template and yields higher Ct values. In contrast, closed chromatin regions (fewer cuts) provide more intact template for qPCR. Therefore, when the difference between open and closed regions is positive, it indicates that the treated samples have reduced accessibility to the qPCR template, reflecting more closed chromatin. This method was additionally validated using the EpiQuik kit (EpigenTek, P-1047).

## **Programming and coding languages**

Bioinformatic data were analyzed using Python, data processing and visualization were done with R, for sequencing data analyses was employed and image data were acquired and processed using Java.

## **References**

1.	Edgar, R., Domrachev, M. & Lash, A. E. Gene Expression Omnibus: NCBI gene expression and hybridization array data repository. *Nucleic Acids Res* **30**, 207–210 (2002).

2.	Tang, Z., Kang, B., Li, C., Chen, T. & Zhang, Z. GEPIA2: an enhanced web server for large-scale expression profiling and interactive analysis. *Nucleic Acids Res* **47**, W556–W560 (2019).

3.	Tang, G., Cho, M. & Wang, X. OncoDB: an interactive online database for analysis of gene expression and viral infection in cancer. *Nucleic Acids Res* **50**, D1334–D1339 (2022).

4.	Zou, Z., Ohta, T. & Oki, S. ChIP-Atlas 3.0: a data-mining suite to explore chromosome architecture together with large-scale regulome data. *Nucleic Acids Res* **52**, W45–W53 (2024).

5.	Colaprico, A. *et al.* TCGAbiolinks: an R/Bioconductor package for integrative analysis of TCGA data. *Nucleic Acids Res* **44**, e71 (2016).

6.	Davis, S. & Meltzer, P. S. GEOquery: a bridge between the Gene Expression Omnibus (GEO) and BioConductor. *Bioinformatics* **23**, 1846–1847 (2007).

7.	Shannon, P. *et al.* Cytoscape: a software environment for integrated models of biomolecular interaction networks. *Genome Res* **13**, 2498–2504 (2003).

8.	Szklarczyk, D. *et al.* The STRING database in 2023: protein-protein association networks and functional enrichment analyses for any sequenced genome of interest. *Nucleic Acids Res* **51**, D638–D646 (2023).

9.	Milacic, M. *et al.* The Reactome Pathway Knowledgebase 2024. *Nucleic Acids Res* **52**, D672–D678 (2024).

10.	Kanehisa, M. & Goto, S. KEGG: kyoto encyclopedia of genes and genomes. *Nucleic Acids Res* **28**, 27–30 (2000).

11.	Ge, S. X., Jung, D. & Yao, R. ShinyGO: a graphical gene-set enrichment tool for animals and plants. *Bioinformatics* **36**, 2628–2629 (2020).

12.	Kolberg, L. *et al.* g:Profiler-interoperable web service for functional enrichment analysis and gene identifier mapping (2023 update). *Nucleic Acids Res* **51**, W207–W212 (2023).

13.	Bindea, G. *et al.* ClueGO: a Cytoscape plug-in to decipher functionally grouped gene ontology and pathway annotation networks. *Bioinformatics* **25**, 1091–1093 (2009).

14.	Krämer, A., Green, J., Pollard, J. & Tugendreich, S. Causal analysis approaches in Ingenuity Pathway Analysis. *Bioinformatics* **30**, 523–530 (2014).

15.	Agrawal, A. *et al.* WikiPathways 2024: next generation pathway database. *Nucleic Acids Res* **52**, D679–D689 (2024).

16.	Mootha, V. K. *et al.* PGC-1alpha-responsive genes involved in oxidative phosphorylation are coordinately downregulated in human diabetes. *Nat Genet* **34**, 267–273 (2003).

17.	Broghammer, F. *et al.* Resistance of HNSCC cell models to pan-FGFR inhibition depends on the EMT phenotype associating with clinical outcome. *Mol Cancer* **23**, 39 (2024).

18.	Klapproth, E. *et al.* Whole exome sequencing identifies mTOR and KEAP1 as potential targets for radiosensitization of HNSCC cells refractory to EGFR and β1 integrin inhibition. *Oncotarget* **9**, 18099–18114 (2018).

19.	Schindelin, J. *et al.* Fiji: an open-source platform for biological-image analysis. *Nat Methods* **9**, 676–682 (2012).

20.	Zhao, Y. *et al.* Automatic Segmentation of Cervical Cells Based on Star-Convex Polygons in Pap Smear Images. *Bioengineering (Basel)* **10**, 47 (2022).

21.	Harrison, P. W. *et al.* Ensembl 2024. *Nucleic Acids Res* **52**, D891–D899 (2024).

22.	Wu, T. D. & Nacu, S. Fast and SNP-tolerant detection of complex variants and splicing in short reads. *Bioinformatics* **26**, 873–881 (2010).

23.	Liao, Y., Smyth, G. K. & Shi, W. featureCounts: an efficient general purpose program for assigning sequence reads to genomic features. *Bioinformatics* **30**, 923–930 (2014).

24.	Love, M. I., Huber, W. & Anders, S. Moderated estimation of fold change and dispersion for RNA-seq data with DESeq2. *Genome Biol* **15**, 550 (2014).

25.	Ignatiadis, N., Klaus, B., Zaugg, J. B. & Huber, W. Data-driven hypothesis weighting increases detection power in genome-scale multiple testing. *Nat Methods* **13**, 577–580 (2016).

26.	Ewels, P. A. *et al.* The nf-core framework for community-curated bioinformatics pipelines. *Nat Biotechnol* **38**, 276–278 (2020).

27.	Heinz, S. *et al.* Simple combinations of lineage-determining transcription factors prime cis-regulatory elements required for macrophage and B cell identities. *Mol Cell* **38**, 576–589 (2010).

28.	Korovina, I., Vehlow, A., Temme, A. & Cordes, N. Targeting integrin α2 as potential strategy for radiochemosensitization of glioblastoma. *Neuro-oncology* **25**, 648–661 (2023).

# Supplementary Figures:

## **Supplementary Figure 1. Extended data related to Figure 1.**

**a** Focal adhesion genes among all differentially expressed genes (DEG) in the TCGA HNSCC dataset. **b** DEG found in HNSCC tissues (TCGA). All data represent significant findings (*p* < 0.05; FDR < 0.05). **c**-**g** Depiction of the hierarchical order of all focal adhesion proteins (FAP) in different networks, with integrins marked in green. Hierarchical ordering of genes in the nucleus (c), plasma membrane (d), cytoplasm (e), chromatin (f), and DEG in the HNSCC (g) nexuses used in Fig. 1b. Gene order is based on the number of first neighbor interactions within the nexus.

## **Supplementary Figure 2.** **Extended data related to Figure 1.**

Kaplan-Meier analyses of the overall survival data shown in Fig. 1e. Evaluation of OS of HNSCC patients, based on 10% upper and lower integrin expression levels. Analysis includes data from 3 independent studies as indicated.

## **Supplementary Figure 3. Continuing Supplementary Figure 2.**

Continuing Kaplan-Meier analyses of the overall survival data shown in Fig. 1e. Evaluation of OS of HNSCC patients, based on 10% upper and lower integrin expression levels. Analysis includes data from 3 independent studies as indicated.

## **Supplementary Figure 4. Extended data related to Figure 1.**

**a** 12 α/β1 integrin-associated HNSCC DEG. **b** Integrin-associated DEG related to epigenetic regulation. **c**-**d** IPA of upstream (c) and master (d) regulators for integrin-associated DEG. Depiction of DEG associated with chromatin regulation indicated green.

## **Supplementary Figure 5.** **Extended data related to Figure 2.**

**a** Qualitative analysis of nucleus morphology across 8 different HNSCC cell models grown in physiological 3D lrECM. LSM980 Airyscan 2-acquired high-resolution Z-stack nuclear images from Hoechst 33342-stained 3D lrECM cell cultures, ordered from most homogeneous (top, left) to most heterogeneous nuclei (bottom, right). **b** Relative occupancy of histone posttranslational modifications (HPMs) determined by dot blot analysis of whole cell lysates from cells cultured under 3D lrECM conditions. For each cell line, the signal intensity of each modification was quantified and normalized to the total signal of all measured modifications within the same sample, yielding the relative percentage distribution of HPMs. Data are presented as mean ± standard deviation (N = 6). **c** Comparative pie diagram of euchromatin and heterochromatin marker distribution based on (b). **d** DNA occupancy analyses of eu- and heterochromatic markers from publicly available ChIP-seq data obtained from HNSCC publicly available databases. **e** Whole-exome sequencing for mutational profiles of chromatin regulatory genes in the selected 8 3D lrECM HNSCC models. **f** Comparative analysis of the average mutation frequencies of chromatin regulatory genes, as depicted in (e), across diverse human populations (data from Genome Aggregation Database (gnomAD); Exome Aggregation Consortium (ExAC)); african/african-american populations (AFR); east asian populations (EAS); south asian populations (SAS); latino/admixed american populations (AMR); finnish populations (FIN); ashkenazi jewish populations (ASJ); non-finnish european populations (NFE); other populations (OTH); aggregate of all populations (ALL). **g** Clustering of shared biological processes and pathways, derived from Reactome, Wikipathways, GO, and KEGG data sources. The analysis was conducted using ClueGO for the genes presented in (e). All functional enrichments are considered statistically significant at *p <* 0.05, with a FDR < 0.05.

## **Supplementary Figure 6. Extended data related to Figure 2.**

**a** LSM980 Airyscan 2-based images of nuclei stained with Hoechst 33342, H3Ac, H3K4me3 and H3K27me3 upon ITGA7 knockdown. Images were 3D segmented and minimum >60% of the fluorescent signal was considered for qualitative analysis. **b** Staining with Hoechst 33342 and for α7 integrin in HNSCC and normal tissue samples. Heatmap shows α7 integrin staining intensity. **c** and **d** Kaplan-Meier analyses of overall survival data of HNSCC patients based on ITGA7 mRNA expression for 50% (c), 35% (d) upper and lower expression levels. Data acquired from GSE65858 data set. **e** Single cell sequencing UMAP clustering on cancer for ITGA7 expression. **f** ITGA7 expression in different cell types with corresponding cell number, data from single cell sequencing analysis. Data are presented as mean ± standard deviation with a sample size of N = 37.

## **Supplementary Figure 7. Extended data related to Figure 3.**

**a** Confirmation of ITGA7 knockdown before (with qPCR) and after (next generation sequencing) RNA-seq and ATAC-seq. Data are presented as mean ± standard deviation with a sample size of N = 4 (unpaired *t* test; *** *p* < 0.001; **** *p* < 0.0001). **b** Dynamic of ITGA7 knockdown from 0 to 70 hours post transfection. **c** and **d** Differentially-expressed genes (DEG; DESeq2; *p <* 0.05, FDR < 0.05) found in Cal-33 (c) and SAS (d) cell models after ITGA7 knockdown compare to controls. **e** IPA of shared canonical pathways changed associated with Cal-33 (c) and SAS (d) DEG.

## **Supplementary Figure 8. Continuing Supplementary Figure 7.**

**a** and **b** IPA of canonical pathways changed associated with Cal-33 (a) and SAS (b) DEG individually. **c**-**f** IPA of upstream (Cal-33 cell model, c; SAS cell model, d) and master (Cal-33 cell model, e; SAS cell model, f) regulators associated with DEG in cell models after ITGA7 knockdown.

## **Supplementary Figure 9. Extended data related to Figure 4.**

**a** and **b** Differentially-accessible chromatin regions (DAR; DESeq2 *p* < 0.05) identified in Cal-33 (a) and SAS (b) cell models following ITGA7 knockdown. **c** Distribution of all ATAC peaks across mapped genomic components in Cal-33 (left) and SAS (right) cells. **d** Comparison of all ATAC peaks numbers across mapped genomic components in ITGA7-depleted and control Cal-33 (left) and SAS (right) cells. Data are presented as mean ± standard deviation with a sample size of N = 4. **e** Distribution of all similarly changed DAR (scDAR) peaks across genomic components. **f** Hierarchical clustering of assigned GO terms, highlighting functional similarities based on ClueGO network analysis of shared epigenetic DAR from Fig. 4g. **g** Shared transcription factors (TF) common to both cell models in increased accessibility and decreased accessibility DAR including “HOMER *de novo* motif discovery” and “known motif scanning”. **h** Correlation of significance levels between TF identified in both cell models is shown.

## **Supplementary Figure 10. Extended data related to Figure 5.**

**a** Knockdown efficiency of ITGA7 for samples used for Kinexus antibody microarray. Data represents qPCR analysis. Data are presented as mean ± standard deviation with a sample size of N *=* 2 (unpaired *t* test, ** *p <* 0.01, *** *p <* 0.001). **b** and **c** Differentially phosphorylated proteins (DPP) in ITGA7-depleted Cal-33 (b) and SAS (c) cells. **d** IPA of canonical pathways associated with DPP in both cell models. **e** and **f** Corresponding shared upstream (e) and master (f) regulators from (d). **g-i** IPA of canonical pathways (g) associated with similarly changed shared DPP (scDPP) and their associated upstream (h) and master (i) regulators. **j** and **k** Differentially-expressed proteins (DEP) in ITGA7-depleted Cal-33 (j) and SAS (k) cells. **l** IPA of canonical pathways significantly altered in both Cal-33 (j) and SAS (k) associated with DEP. **m** and **n** IPA of shared upstream (m) and master (n) regulators associated with Cal-33 (j) and SAS (k) DEP. **o**-**q** IPA of canonical pathways (o) for similarly changed shared DEP (scDEP) and their associated upstream (p) and master (q) regulators. Data are presented as both cell models mean ± standard deviation for *p*-value and activation Z-score.

## **Supplementary Figure 11. Extended data related to Figure 6.**

**a**-**d** Learning curves showing model performance with varying training set sizes for RNA-seq (a), ATAC-seq (b), phosphoproteomic (c), and proteomic (d) datasets. **e**-**l** PCA of the sample datasets based on gene expression and their corresponding PCA loadings for each gene in positioned according to its loadings in Principal Component 1 (PC1) and Principal Component 2 (PC2) for RNA-seq (e and f), ATAC-seq (g and h), phosphoproteomic (i and j), and proteomic (k and l) datasets. The loadings reflect the extent and direction of each gene's contribution to the principal components, indicating how much each gene influences the variance captured by PC1 and PC2. Marked points indicate genes selected by the random forest as key features. **m**-**p** Key random forest features importances with the corresponding data fold changes (knockdown *vs*. control) in both cell models for RNA-seq (m), ATAC-seq (n), phosphoproteomic (o), and proteomic (p) datasets. **q** Representative images of clonogenic survival of 3D lrECM grown HNSCC models following ITGA7 depletion.

## **Supplementary Figure 12. Extended data related to Figure 6 and 7.**

**a** Representative segmented images of Ki-67/BrdU-positive cells upon ITGA7 silencing for data on Fig. 6i. **b** Representative images of PDOs grown in 3D lrECM following ITGA7 knockout with 3 different guiding RNAs. **c** ITGA7 based linear regression model comparing mean fold change in viability after single and double gene knockdown. Each point represents the mean effect across biological replicates. The shaded region represents a ±15% deviation band around the regression-predicted values. **d** Statistical evaluation of the ITGA7 expression in HNSCC patients in the order from low to high expression. **e** Boundary accumulation plot across the rank-ordered ITGA7 axis. Each point represents the frequency with which a specific rank boundary was used among significant 4-bin splits (log-rank *p* < 0.05). Two prominent accumulation peaks indicate stable transition zones in ITGA7 expression associated with survival stratification. **f** Hexagonal density plot of bin median ITGA7 expression evaluated 4-bin splits. Color intensity indicates density of bin edges.

## **Supplementary Figure 13. Extended data related to Figure 7.**

**a** Western blot images from whole cell lysates of Cal-33 and SAS cell models showing phosphorylation and total expression levels of the indicated proteins (see raw images in Supplementary Data S8). **b** Western blot densitometry quantifications from whole cell lysates of Cal-33 and SAS cell models. β-actin normalized phosphorylation after ITGA7 knockdown relative to controls. Data are presented as mean ± standard deviation with a minimum sample size of N ≥ 4 (unpaired t-test; *ns* > 0.05 *p <* 0.5 - 0.0001).

## **Supplementary Figure 14. Extended data related to Figure 7.**

Hand constructed integrin α7 signaling via the AKT/MAPK/AURKA pathway. Color code indicates the acquired experimental evidence for similarly changed differentially expressed proteins, genes, chromatin regions (scDEP, scDEG, scDAR).

# Supplementary Tables:

Supplementary Table S1.

# Supplementary Data:

Supplementary Data S1 (Supporting file for Figure 1).

Supplementary Data S2 (Supporting file for Figure 2).

Supplementary Data S3 (Supporting file for Figure 3).

Supplementary Data S4 (Supporting file for Figure 4).

Supplementary Data S5 (Supporting file for Figure 5).

Supplementary Data S6 (Supporting file for Figure 6).

Supplementary Data S7 (Supporting file for Figure 7).

Supplementary Data S8 (Raw western blot images).

# Supplementary Videos:

Supplementary Video S1 (3D nuclear architecture in 3D lrECM Cal-33 cultures).

Supplementary Video S2 (3D nuclear architecture in 3D lrECM for UT-SCC-8 cultures).

Supplementary Video S3 (3D nuclear architecture in 3D lrECM for UT-SCC-15 cultures).

Supplementary Video S4 (3D nuclear architecture in 3D lrECM for UT-SCC-5 cultures).

Supplementary Video S5 (3D nuclear architecture in 3D lrECM for FaDu cultures).

Supplementary Video S6 (3D nuclear architecture in 3D lrECM for HSC4 cultures).

Supplementary Video S7 (3D nuclear architecture in 3D lrECM for UT-SCC-14 cultures).

Supplementary Video S8 (3D nuclear architecture in 3D lrECM for SAS cultures).
